# Nonpharmacological Treatment of Persistent Postconcussion Symptoms in Adults: A Systematic Review and Meta-analysis and Guideline Recommendation

**DOI:** 10.1001/jamanetworkopen.2021.32221

**Published:** 2021-11-09

**Authors:** Hana Malá Rytter, Heidi J. Graff, Henriette K. Henriksen, Nicolai Aaen, Jan Hartvigsen, Morten Hoegh, Ivan Nisted, Erhard Trillingsgaard Næss-Schmidt, Lisbeth Lund Pedersen, Henrik Winther Schytz, Mille Møller Thastum, Bente Zerlang, Henriette Edemann Callesen

**Affiliations:** 1 Danish Concussion Center, Copenhagen, Denmark; 2 Department of Psychology, University of Copenhagen, Copenhagen, Denmark; 3 Department of Neurology, Bispebjerg-Frederiksberg University Hospital, Copenhagen, Denmark; 4 Center for Rehabilitation of Brain Injury, Copenhagen, Denmark; 5 The Danish Concussion Association, Copenhagen, Denmark; 6 Department of Sports Science and Clinical Biomechanics, University of Southern Denmark, Odense, Denmark; 7 Chiropractic Knowledge Hub, Odense, Denmark; 8 Musculoskeletal Health and Implementation, Department of Medicine, Aalborg University, Aalborg, Denmark; 9 Pain Science Educator, Aarhus, Denmark; 10 Danish College of Optometry, Dania Academy, Randers, Denmark; 11 Department of Clinical Medicine, Aarhus University, Aarhus, Denmark; 12 Hammel Neurorehabilitation Centre–University Clinic for Neurorehabilitation, Aarhus University, Aarhus, Denmark; 13 Alleens Physiotherapy and Sports Clinic, Odense, Denmark; 14 Danish Headache Center, Rigshospitalet-Glostrup, University of Copenhagen, Copenhagen, Denmark; 15 Exercise and Health Training Center, Roskilde Municipality, Roskilde, Denmark; 16 Callesen Method Consulting, Copenhagen, Denmark

## Abstract

**Importance:**

Persistent (>4 weeks) postconcussion symptoms (PPCS) are challenging for both patients and clinicians. There is uncertainty about the effect of commonly applied nonpharmacological treatments for the management of PPCS.

**Objective:**

To systematically assess and summarize evidence for outcomes related to 7 nonpharmacological interventions for PPCS in adults (aged >18 years) and provide recommendations for clinical practice.

**Data Sources:**

Systematic literature searches were performed via Embase, MEDLINE, PsycINFO, CINAHL, PEDro, OTseeker, and Cochrane Reviews (via MEDLINE and Embase) from earliest possible publication year to March 3, 2020. The literature was searched for prior systematic reviews and primary studies. To be included, studies had to be intervention studies with a control group and focus on PPCS.

**Study Selection:**

A multidisciplinary guideline panel selected interventions based on frequency of use and need for decision support among clinicians, including early information and advice, graded physical exercise, vestibular rehabilitation, manual treatment of neck and back, oculomotor vision treatment, psychological treatment, and interdisciplinary coordinated rehabilitative treatment. To be included, studies had to be intervention studies within the areas of the predefined clinical questions, include a control group, and focus on symptoms after concussion or mild traumatic brain injury.

**Data Extraction and Synthesis:**

Extraction was performed independently by multiple observers. The Preferred Reporting Items for Systematic Reviews and Meta-Analyses (PRISMA) guidelines were used for data abstraction and data quality assessment. Included studies were assessed using the Assessment of Multiple Systematic Reviews (AMSTAR) tool and the Cochrane Risk of Bias (randomized clinical trials) tool. Meta-analysis was performed for all interventions where possible. Random-effects models were used to calculate pooled estimates of effects. The level and certainty of evidence was rated and recommendations formulated according to the Grading of Recommendations, Assessment, Development, and Evaluations (GRADE) framework.

**Main Outcomes and Measures:**

All outcomes were planned before data collection began according to a specified protocol. The primary outcomes were the collective burden of PPCS and another outcome reflecting the focus of a particular intervention (eg, physical functioning after graded exercise intervention).

**Results:**

Eleven systematic reviews were identified but did not contribute any primary studies; 19 randomized clinical trials comprising 2007 participants (1064 women [53.0%]) were separately identified and included. Evidence for the 7 interventions ranged from no evidence meeting the inclusion criteria to very low and low levels of evidence. Recommendations were weak for early information and advice, graded physical exercise, vestibular rehabilitation, manual treatment of the neck and back, psychological treatment, and interdisciplinary coordinated rehabilitative treatment. No relevant evidence was identified for oculomotor vision treatment, so the panel provided a good clinical practice recommendation based on consensus.

**Conclusions and Relevance:**

Based on very low to low certainty of evidence or based on consensus, the guideline panel found weak scientific support for commonly applied nonpharmacological interventions to treat PPCS. Results align with recommendations in international guidelines. Intensified research into all types of intervention for PPCS is needed.

## Key Points

**Question:**

What is the evidence for nonpharmacological interventions to treat persistent postconcussion symptoms?

**Findings:**

Following a systematic review and meta-analysis of 19 randomized clinical trials comprising 2007 participants, using the Grading of Recommendations, Assessment, Development, and Evaluations method, weak recommendations for the following were assigned: systematic provision of early information and advice, use of graded physical exercise, vestibular rehabilitation, manual treatment of neck and back, psychological treatment, and interdisciplinary rehabilitation. No studies were identified regarding oculomotor vision treatment, resulting in a consensus-based statement.

**Meaning:**

Based on very low to low certainty of evidence or on consensus, individually tailored nonpharmacological treatment of persistent symptoms was recommended, both through specific disciplines and interdisciplinary rehabilitation.

## Teaser

This systematic review and meta-analysis assesses the outcomes related to 7 nonpharmacological interventions for persistent postconcussion symptoms in adults and provides recommendations for clinical practice.

## Introduction

Concussion or mild traumatic brain injury (mTBI) accounts for up to 90% of all TBIs.^1^ The yearly incidence in Denmark is 457 per 100 000 inhabitants^2^; however, these numbers do not include those who consult general practitioners or do not seek care. The true incidence is estimated to be around 600 per 100 000.^3^

Concussion or mTBI is defined as a transmission of mechanical energy due to a blow to the head, neck, or body that results in disruption of brain function.^4,5^ Consensus regarding diagnostic criteria emphasizes at least 1 of the following signs: (1) any alteration of mental state immediately after injury, (2) posttraumatic amnesia for less than 24 hours, (3) loss of consciousness for less than 30 minutes, or (4) other signs of focal and transient neurologic dysfunction.^5,6,7^

Most people who sustain a concussion or mTBI recover quickly, but a significant proportion experience long-term symptoms.^8,9,10^ According to the *International Statistical Classification of Diseases and Related Health Problems, Tenth Revision* (*ICD-10*),^11^ recovery from concussion is expected within the first 2 to 3 weeks, and persistent symptoms are defined as those lasting for more than 4 weeks after injury.^12^ Studies indicate that up to 34% to 44% of patients with concussion or mTBI experience symptoms at 3 to 6 months after injury, and between 5% and 20% experience symptoms at 12 months after injury.^8,13,14,15^

Persistent postconcussion symptoms (PPCS) comprise a combination of physical (headache, dizziness, blurred vision, sleep disturbance, neck pain, and fatigue), cognitive (slowed thinking, difficulties with attention, concentration, memory, or executive functions), and emotional or behavioral symptoms (changed emotional responsivity, irritability, quickness to anger, disinhibition, or emotional lability),^16^ and might be associated with changes in personality and difficulties regarding personal and professional identity.^17^ There is an uncertainty regarding the effectiveness of commonly applied nonpharmacological interventions to treat PPCS, and to our knowledge, there is a scarcity of meta-analyses dedicated to this topic. Furthermore, although systematic approaches are in general applied, the Grades of Recommendation, Assessment, Development, and Evaluation (GRADE)^18^ approach has only been used sporadically.

The Danish Health Authority commissioned a set of National Clinical Guidelines with the objective to evaluate and summarize the evidence for the effectiveness of nonpharmacological interventions in adults experiencing PPCS and to provide recommendations for clinical practice. The selected areas of interest were (1) early information and advice, (2) graded physical exercise, (3) vestibular rehabilitation, (4) manual treatment of neck and spine, (5) oculomotor vision treatment, (6) psychological treatment, and (7) interdisciplinary coordinated rehabilitative treatment. This article reports the methodology and results of this guideline based on meta-analyses and the GRADE approach.

## Methods

The content of this guideline is structured around selected clinical questions in accordance with the Population, Intervention, Comparison, and Outcome (PICO) framework.^19^ Making recommendations for diagnostic procedures, care pathways, or providing estimates of associated costs were beyond the scope of this guideline. Recommendations are based on systematic reviews and follow the principles described in GRADE.^18^ This guideline followed the AGREE reporting checklist. The complete clinical guideline is available in Danish online.^20^ A protocol for this systematic review and meta-analysis has been registered in the PROSPERO database.^21^ The protocol for this study was reviewed and approved by the Danish Health Authority. This study followed the Preferred Reporting Items for Systematic Reviews and Meta-analyses (PRISMA) reporting guideline.

### Organization

The work was performed by a multidisciplinary group forming the guideline panel. This included a chairman, search specialist, methodologist, lead reviewer, and members appointed by professional societies and a patient organization. Members had clinical and academic expertise from neurology, physical therapy, neuropsychology, occupational therapy, optometry, chiropractic medicine, pain science, and epidemiology. The guideline panel was involved in every major step of the process. The work was coordinated by the chairman (H.M.R.) assisted by the lead reviewer (H.J.G.) and the methodologist (H.E.C.). If an included study was authored by a member of the guideline panel, the study was appraised by other members of the group. In addition, a reference group including stakeholders from the Danish health care system (ie, municipalities, hospitals, rehabilitation institutions) gave feedback on the PICO questions and the final recommendations. Any potential conflicts of interests were declared before initiating the work and can be accessed online in Danish.^22^ The draft of the clinical guideline was peer reviewed by ^2^ external reviewers as well as in a public hearing.

### Clinical Questions

Seven clinical questions regarding the outcomes of the following nonpharmacological interventions were selected: (1) early information and advice to prevent PPCS, (2) graded physical exercise, (3) vestibular rehabilitation to treat persistent vestibular dysfunction, (4) manual treatment of the neck and back, (5) oculomotor vision treatment to treat persistent visual symptoms, (6) psychological treatment and (7) interdisciplinary coordinated rehabilitative treatment. For early information and advice, such information had to be provided systematically and start within 4 weeks after injury. In the remaining clinical questions, patients had to be diagnosed with concussion or mTBI and experience symptoms that persisted for more than 4 weeks after injury. The interventions were compared with either no intervention or treatment as usual (eTable 4 in the Supplement).

### Eligibility Criteria

To be included, studies had to (1) be intervention studies within the areas of the predefined clinical questions, (2) include a control group, and (3) focus on symptoms after concussion or mTBI. Studies including both concussion or mTBI and moderate to severe TBI were only included if it was possible to extract separate data for the concussion/mTBI population. Participants had to be aged 18 years or older and be diagnosed with concussion or mTBI. Consent (written or oral) from the participants was not obtained, as the study only used data from previously published studies. Studies including adolescents were included only if adolescent participants represented a minority of the study sample. Data on patient race and ethnicity were not collected because we did not assume that these variables would have a significant effect on the outcomes in the included studies. Collection of data on race and ethnicity was not required by the funding agency.

### Outcomes

For each clinical question, 2 primary outcomes were chosen alongside a number of secondary outcomes. The primary outcome for the majority of clinical questions was the collective burden of postconcussion symptoms supplemented by another primary outcome closely reflecting the focus of the respective interventions (eg, physical functioning after graded physical exercise, pain after manual treatment of neck and back, emotional symptoms after psychological treatment). The secondary outcomes also varied across the clinical questions and included pain, physical functioning, emotional symptoms, behavioral response, quality of life, negative effect on prognosis, and return to daily activities. A time frame for each outcome was chosen a priori. A complete overview of outcomes and time frames is available in eTable 2 in the Supplement.

### Literature Searches and Study Selection

A systematic literature search was conducted from November 22, 2019, to March 3, 2020, in Embase, MEDLINE, PsycINFO, CINAHL, PEDro, OTseeker, and Cochrane Reviews (via MEDLINE and Embase) from the earliest possible publication year and up to March 3, 2020. The search strategy was subdivided into searches for relevant randomized clinical trials (RCTs) or observational studies with a control group included in either the guidelines or the systematic reviews. Finally, a search was performed to identify individual studies not included in existing guidelines or systematic reviews and for interventions for which no reviews were available. When relevant, the search date for individual studies was limited to the dates of the latest search in the included reviews (eTable 1 in the Supplement).

Titles and abstracts of potential studies were screened by the lead reviewer (H.J.G.). Subsequently, the full text of potential studies was screened by the lead reviewer (H.J.G.) and members of the working group (J.H., M.H., I.N., E.T.N-S., L.L.P., H.W.S., M.M.T., and B.Z.) for eligibility. Disagreement was discussed by consultation with a third reviewer (H.M.R.) and/or the methodologist (H.E.C.). The selection process was aided by using Covidence software, a web-based tool.^23^ The review authors were not blinded to the journal titles, study authors, study institutions, or the year of publication. A PRISMA flowchart was created to document the number of included and excluded studies identified during the search process (eFigure 1 in the Supplement).

### Data Extraction and Quality Assessment

Information extracted included the population demographic and baseline characteristics, details on intervention and control treatments, study design, outcomes, and time of measurement. The lead reviewer and methodologist independently extracted the data and assessed the risk of bias for all RCTs using the Cochrane risk of bias tool.^24^ If information was missing or unclear, this was noted without making any assumptions regarding the cause. If clinical guidelines or systematic reviews were identified, their quality was evaluated using the Appraisal of Guidelines for Research and Evaluation (AGREE-II)^25^ or Assessment of Multiple Systematic Reviews (AMSTAR) tools.^26^ Any discrepancies were identified and resolved through discussion with a third reviewer.

### Statistical Analysis

Meta-analyses were performed to give a summary measure of the outcome if data across trials for a given outcome were comparable. Analyses were performed in the program RevMan5, version 5.3 (Cochrane Collaboration). If data were inadequate for inclusion in a meta-analysis, the study results were narratively described. For dichotomous outcomes, the relative risk including the 95% CI was calculated. For continuous outcomes, effect size was calculated using standardized mean difference including the 95% CI if different measurement scales were used. If the continuous outcomes across studies were measured using the same measurement scale, effect size was assessed as mean difference. Random-effect models were used to calculate pooled estimates of effects. Statistical heterogeneity was quantified using the *I*^2^ statistic,^27^ with an *I*^2^ value greater than 50% considered to be substantial heterogeneity. A forest plot was provided for each meta-analysis (eFigure 2 in the Supplement).

#### Certainty of Evidence

The outcome measures were combined into a single summary of findings table. The certainty of evidence was then determined according to the GRADE,^28^ resulting in 4 possible ratings: high, moderate, low, and very low (Table 1). If applicable, downgrading was done for each outcome by evaluating the extent of risk of bias, inconsistency, indirectness, imprecision, and publication bias.^28,29^ The overall quality of evidence for each clinical question was based on the lowest rating for the primary outcome.

**Table 1.** Definitions of the Certainty of Evidence Based on the Grading of Recommendations, Assessment, Development, and Evaluations (GRADE) Approach^a^

Certainty of evidence	Definition
High	We are confident that the estimated effect lies close to the true effect.
Moderate	We are moderately confident that the estimated effect is likely to be close to the true effect. However, there is a possibility that it is substantially different.
Low	We have limited confidence in the estimated effect as it may be substantially different from the true effect.
Very low	We have very limited confidence in the estimated effect, as it is likely to be substantially different from the true effect.

^a^ This table was adapted from Balshem et al.^28^

#### From Evidence to Recommendation

Either a strong or weak recommendation in favor of or against an intervention was made based on a combined assessment of the strength of the available evidence, assumed patient preferences, and weighting of benefits and harms. A good clinical practice recommendation was made in case there was no available evidence, ie, RCTs or observational studies with a control group (Table 2).^30^

**Table 2.** Definitions of Recommendations Based on the Grading of Recommendations, Assessment, Development, and Evaluations (GRADE) Approach and by the Danish Health Authority^a^

Recommendation	Definition
Strong recommendation for	A strong recommendation in favor of an intervention is given when there is high-quality evidence showing that the overall benefits of the intervention are clearly greater than the disadvantages. The majority of the patients would want the intervention.
Weak recommendation for	A weak recommendation in favor of an intervention is given when it is assessed that the advantages of the intervention outweigh the disadvantages or if the available evidence cannot rule out a significant benefit of the intervention while at the same time the harmful effects are few or absent. This recommendation is also given when there are substantial variations in patient preferences.
Strong recommendation against	A strong recommendation against an intervention is given when there is high-quality evidence showing that the overall disadvantages of the intervention are clearly greater than the benefits. The majority of the patients would not want the intervention.
Weak recommendation against	A weak recommendation against an intervention is given when it is assessed that the disadvantages of the intervention outweigh the advantages but where it is not substantiated by high-quality evidence. This recommendation is also used where there is high-quality evidence for both beneficial and harmful effects but where the balance between them is difficult to determine. This recommendation is also given when there are substantial variations in patient preferences.
Good clinical practice statement	A good clinical practice statement is used when there is no relevant evidence to answer the clinical questions and thus the recommendation is based on professional consensus among the members of the working group that drafted the guideline. The recommendation can be either for or against the intervention. Because this is based on professional consensus, this type of recommendation is weaker than any evidence-based recommendation.

^a^ This table was adapted from the Danish Health Authority.

## Results

A total of 13 097 articles were identified; of those, 109 were excluded as duplicates. Among the remaining 12 988 articles, titles and abstracts were screened for inclusion and exclusion criteria. A total of 297 articles received a full-text review, and 11 relevant systematic reviews^31,32,33,34,35,36,37,38,39,40,41^ were identified for 5 of the clinical questions. Only 1 systematic review^33^ matched our clinical question (graded physical exercise) and was of sufficient methodological quality(eTable 3 in the Supplement); however, it did not contribute any primary study matching our inclusion and exclusion criteria. The search date for individual studies for this question was then limited to the date of the latest search in the included systematic review,^33^ and here we identified 2 relevant RCTs.^42,43^ Furthermore, 1 relevant RCT^44^ was identified in a systematic review for manual treatment of neck and back. A total of 19 individual RCTs^42,43,44,45,46,47,48,49,50,51,52,53,54,55,56,57,58,59,60^ comprising 2007 participants (1064 women [53.0%]; 943 men [47.0%]) were identified for 6 of the clinical questions. Nine^43,45,46,47,49,52,53,55,60^ of these included a minority of adolescents in the sample. No evidence was identified for the effect of oculomotor vision treatment. Thus, all recommendations were based exclusively on primary studies. For characteristics of the included studies, see eTable 5 in the Supplement. For flowcharts of included literature, see eFigure 1 in the Supplement.

The available evidence was limited for all interventions, ranging from low to very low mainly owing to risk of bias, imprecision (few studies identified or few patients included), and indirectness (patients aged <18 years included) (Table 3). For results of meta-analyses, see eFigure 2 in the Supplement. For summary of findings table, including the rating of the evidence and risk of bias assessment, see eTable 4 in the Supplement. For recommendations including the supporting evidence, see Table 4.

**Table 3.** Overview of Recommendations in the Guideline and the Certainty of Evidence^a^

PICO	Intervention	Certainty of evidence	Recommendation
PICO 1	Systematically offered information and advice	Very low	Weak recommendation for
PICO 2	Graded physical exercise	Very low	Weak recommendation for
PICO 3	Vestibular rehabilitation	Very low	Weak recommendation for
PICO 4	Spinal manual therapy	Very low	Weak recommendation for
PICO 5	Oculomotor vision treatment	No relevant evidence identified	Good clinical practice statement
PICO 6	Psychological treatment	Low	Weak recommendation for
PICO 7	Interdisciplinary coordinated rehabilitative treatment	Low	Weak recommendation for

Abbreviation: PICO, Population, Intervention, Comparison, and Outcome.

^a^ Definitions of certainty of evidence and recommendations are noted in Tables 1 and 2.

**Table 4.** Overall Population, Intervention, Comparison, and Outcome (PICO) Questions, Recommendations, Definitions of Interventions, and Primary Outcomes, Supporting Evidence, and Rationale

Recommendation	**Definition of intervention**	**Primary outcomes**	**Included studies**	**Rationale**
**PICO 1. Should patients, early after concussion, be systematically offered early information and advice on preventing persistent postconcussive symptoms?**				
Consider systematically offering early information and advice to patients within the first 4 wk after concussion.	Definition of intervention: Systematic education, instructions, advice and guidance regarding postconcussion symptoms, symptom management, restitution, and self-care provided individually or in groups, either in person or as telephone guidance by a health care professional, using oral and/or written information. The intervention must be initiated within the first 4 wk after injury and should be provided by relevant health professionals.	The collective burden of postconcussion symptoms assessed a minimum of 2 wk after completed intervention Emotional symptoms assessed a minimum of 1 mo after completed intervention	Bell et al,^45^ Heskestad et al,^46^ Matuseviciene et al,^47^ Mittenberg et al,^48^ Ponsford et al,^49^ Suffoletto et al,^50^ and Varner et al^51^	The intervention was associated with a positive effect on the overall symptom burden 2 wk after completion. Furthermore, intervention reduced the number of patients who subsequently experienced memory problems and the number of patients in which leisure and working life was affected. There were no reported serious adverse effects; however, this was not systematically assessed. The certainty of evidence was very low due to risk of bias, indirectness, and imprecision. It was assumed that there was no substantial variability in terms of patient preferences and that the majority of patients would want the intervention. Based on a collective assessment of these findings, a weak recommendation was given for the use of systematic information and advice.
**PICO 2. Should patients with persistent postconcussive symptoms be offered graded physical exercise?**				
Consider offering graded physical exercise in addition to other treatment to patients with persistent postconcussion symptoms.	Definition of intervention: Graded physical exercise, ie, physical exercise with a gradual increase in intensity and/or complexity over time, such as general physiotherapy, general physical activity, sensorimotor training, aerobic and anaerobic training, performed minimally 1 time/wk for 4 wk.	The collective burden of postconcussion symptoms assessed at the end of completed intervention Physical functioning assessed at the end of completed intervention	Rytter et al^42^ and Thastum et al^43^	The intervention in addition to other treatment was associated with a positive effect on both the overall burden of symptoms, the level of physical functioning, behavioral reactions, emotional symptoms, quality of life, and the general satisfaction with the current work situation. There were no reported serious adverse events; however, this was not systematically assessed. The certainty of the evidence was very low due to serious risk of bias, indirectness, and imprecision. It was assumed that there was no substantial variability in terms of patient preferences and that the majority of patients would want the intervention. Based on a collective assessment of these findings, a weak recommendation was given for the use of graded physical exercise.
**PICO 3. Should patients with persistent vestibular dysfunction after concussion be offered vestibular rehabilitation?**				
Consider offering vestibular rehabilitation in addition to other treatments to patients who experience persistent vestibular dysfunction after concussion.	Vestibular rehabilitation, including the otolith manipulating procedures, habituation and adaptation exercises, substitution training, and balance training administered minimally 1 time/wk for a period of 4 wk.	The collective burden of postconcussion symptoms assessed at the end of completed intervention Vestibular dysfunction assessed at the end of completed intervention	Kleffelgaard et al^53^ and Schneider et al^52^	The intervention was associated with a positive effect on the level of physical functioning as well as the number of patients considered ready to return to sport after completed intervention. No serious adverse events were reported; however, this was not systematically assessed. The certainty of the evidence was very low due to risk of bias, indirectness, and imprecision. It was assumed that there was no substantial variability in terms of patient preferences and that the majority of patients would want the intervention. Based on a collective assessment of these findings, a weak recommendation was given for the use of vestibular rehabilitation.
**PICO 4. Should patients with persistent postconcussive symptoms be offered spinal manual therapy, ie, mobilization and manipulation of the neck and spine?**				
Consider offering manual treatment of neck and spine in addition to other treatments to patients with persistent symptoms after concussion.	Manual therapy in the form of hands-on mobilization and/or manipulation of the spine or other joints, typically performed by physiotherapists or chiropractors.	Physical functioning assessed at the end of completed intervention Pain assessed at the end of completed intervention	Schneider et al^52^ and Jensen et al^44^	The intervention was associated with a positive effect on pain as well as the number of patients considered ready to return to sport after completed intervention. No serious adverse events were reported; however, this was not systematically assessed. The certainty of the evidence was very low due to risk of bias, indirectness, and imprecision. It is expected that the intervention will include differences in patient preferences, as some patient would want the treatment whereas others would not. Based on a collective assessment of these findings, a weak recommendation was given for the use of spinal manual therapy.
**PICO 5. Should patients with persistent visual symptoms after concussion be offered oculomotor vision treatment?**				
It is good clinical practice to consider offering oculomotor vision treatment to patients who experience persistent visual symptoms after concussion.	Oculomotor vision treatment, ie, oculomotor training to treat vergence, accommodative, or eye movement dysfunction after concussion, including computer-based training and optometric instrumental training administered as an optometric session minimally 1 time/wk during a period of 4 wk.	Oculomotor dysfunction assessed at the end of completed intervention Visual functioning assessed at the end of completed intervention	No relevant trials identified	Clinical experience shows that oculomotor visual treatment improves the visual symptoms as well as reduces other symptoms such as headache and tiredness in patients with persistent symptoms after concussion. In addition, there is clinical consensus that oculomotor visual therapy has a positive effect on the number of patients returning to work. Because there were no relevant trials identified, this recommendation is largely based on this clinical experience. There are, however, peer-reviewed studies without a control group, showing a positive effect of oculomotor visual therapy in patients with concussion (Gallaway et al^61^; Thiagarajan and Cuiffreda^62^; Thiagarajan et al^63^; Thiagarajan and Cuiffreda^64^). Based on a collective assessment of these findings, a good clinical practice statement was given for the use of visual therapy.
**PICO 6. Should patients with persistent post-concussive symptoms be offered psychological treatment?**				
Consider offering psychological treatment in addition to other treatment to patients with persistent symptoms after concussion.	Psychological treatment by psychologists or clinicians with similar professional background administered minimally 1 h/wk as either individual or group therapy for a period of minimally 4 wk.	The collective burden of postconcussion symptoms assessed after completed intervention Emotional symptoms assessed a minimum of 3 mo after completed intervention	Caplain et al,^54^ Elgmark-Andersson et a,l^55^ Kjeldgaard et a,l^56^ Potter et al,^57^ Rytter et al,^42^ Silverberg et al,^58^ Thastum et al,^43^ Tiersky et al,^59^ and Vikane et al^60^	The intervention was associated with a positive effect on the overall burden of symptoms after the completion of the intervention as well as at longest follow-up. A positive effect was also seen with respect to emotional symptoms and quality of life at the longest follow-up. No serious adverse events were reported; however, this was not systematically assessed. The overall certainty of evidence was low due to risk of bias and indirectness. It is expected that the intervention will include differences in patient preferences, as some patient would want the treatment whereas others would not. Based on a collective assessment of these findings, a weak recommendation was given for the use of psychological treatment.
**PICO 7. Should patients with persistent postconcussive symptoms be offered an interdisciplinary coordinated rehabilitative treatment?**				
Consider offering interdisciplinary coordinated rehabilitative treatment to patients with persistent symptoms after concussion.	An interdisciplinary coordinated rehabilitative treatment is a treatment provided by health professionals from at least 2 different disciplines, who collaborate on the rehabilitation of the patient. The treatment includes at least 2 interventions, eg, vestibular rehabilitation, graded physical exercise, oculomotor vision therapy, manual treatment, (neuro)psychological and psychotherapeutic intervention, advice on managing everyday activities, and vocational rehabilitation and appears as a comprehensive interdisciplinary approach. The treatment is administered minimally 1 time/wk during a period of 4 wk by clinicians with relevant background, eg, physiotherapists, occupational therapist, nurses, rehabilitation therapists, (neuro)psychologists, neurologists.	The collective burden of postconcussion symptoms assessed after completed intervention Return to daily activities assessed a minimum of 3 month after completed intervention	Rytter et a,l^42^ Thastum et al,^43^ and Vikane et al^60^	The intervention was associated with a positive effect on the overall burden of symptoms, the level of physical functioning, emotional symptoms, as well as on quality of life and the general satisfaction with work life. No serious adverse events were reported; however, this was not systematically assessed. The overall certainty of evidence was low due to risk of bias and indirectness. It was assumed that there was no substantial variability in terms of patient preferences and that the majority of patients would want the intervention. Based on a collective assessment of these findings, a weak recommendation was given for the use of interdisciplinary coordinated rehabilitative treatment

### Recommendations

A weak recommendation was given for systematically providing early information and advice, the use of graded physical exercise, the use of vestibular rehabilitation, the use of manual treatment of neck and back, psychological treatment, and interdisciplinary rehabilitative treatment. No relevant evidence was identified for oculomotor vision treatment, so the panel provided a good clinical practice recommendation based on consensus (Tables 3 and 4).

## Discussion

This guideline considered 7 clinical questions regarding nonpharmacological management of PPCS. Based on very low to low certainty of evidence, the guideline panel provided weak recommendations for early information and advice, graded physical exercise, vestibular rehabilitation for persistent vestibular dysfunction, manual treatment of the neck and back, psychological treatment, and interdisciplinary coordinated rehabilitative treatment. A consensus-based recommendation was provided for oculomotor vision treatment to treat persistent visual symptoms. The guideline panel concluded that intensified research into all intervention types is needed.

Given that concussion or mTBI can be associated with persistent symptoms and disability,^65,66,67,68^ there has been a shift from passively awaiting symptom remission to recommending active management when symptoms persist. Thus, our recommendations align with the Synthesis of Practice Guidelines from the American Congress of Rehabilitation Medicine’s Mild TBI task force^4^; the American Physical Therapy Association’s (APTA) guideline for physical therapy evaluation and treatment after concussion or mild TBI^69^; the third edition of the Ontario Neurotrauma Foundation’s (ONF) Guideline for concussion or mild TBI and persistent symptoms^5^; and the latest update on the consensus statement published by the Concussion in Sports Group (CISG).^70^ However, compared with our guideline, these guidelines have different scopes and include pediatric and adolescent populations. Furthermore, although these guidelines apply a systematic approach to guideline generation, they do not apply the GRADE approach.

Early information and advice was recommended based on 7 RCTs,^45,46,47,48,49,50,51^ of which 6 were included in the meta-analysis. The study by Ponsford et al^49^ was not included because it was presented as a short report. Collectively, the studies concluded that information should be tailored to individual patient needs and that information and advice are particularly beneficial if provided over a longer period of time. This recommendation aligns with the Synthesis of Practice Guidelines^4^ as well as with the ONF^5^ and APTA^69^ guidelines that give a grade A, or strong, recommendation. The CISG guideline^70^ does not address this question. Our guideline provides a weak recommendation based on GRADE owing to possible risks of bias in the included studies as well as indirectness.

Graded physical exercise was recommended based on 2 RCTs.^42,43^ In both, the intervention was part of interdisciplinary rehabilitation programs with several interventions applied in parallel. Critical outcomes included physical functioning, which presumably reflected the effect of graded physical exercise. Both the APTA^69^ and the CISG guideline^70^ recommend graded physical exercise as a treatment of autonomic instability or physical deconditioning, with the APTA^69^ indicating level A, or strong evidence. The ONF guideline^5^ recommends exercise below symptom threshold in management of posttraumatic fatigue (grade C). The Synthesis of Practice Guidelines^4^ advocates exercise in general. Our guideline arrived at a weak recommendation based on GRADE.

Vestibular rehabilitation was recommended based on 2 RCTs.^52,53^ In both, participants also received exercise and manual treatment of the cervical and thoracic spine. The ONF^5^ and APTA guidelines^69^ give a grade A, or strong, recommendation for dealing with benign paroxysmal positional vertigo. Furthermore, the ONF guideline gives a grade A recommendation for treatment of unilateral peripheral vestibular dysfunction, whereas the APTA guideline indicates level B, or moderate evidence, for vestibular rehabilitation in conjunction with oculomotor rehabilitation. The ONF guideline gives a grade C consensus-based recommendation, and APTA indicates level C, or weak evidence, for treatment of functional balance impairment. The CISG guideline^70^ recommends a targeted physical therapy program for patients with vestibular dysfunction without further specification.

Manual treatment of the neck and back, ie, mobilization and/or manipulation, is recommended based on 2 small RCTs,^44,52^ of which only one^52^ could be included in the meta-analysis owing to poor reporting in the other.^44^ The 2 studies indicate possible beneficial outcomes regarding pain and return to sport, resulting in a weak recommendation. The Synthesis of Practice Guidelines^4^ and the ONF guideline^5^ do not include manual treatment of the neck and back. The APTA guideline recommends that practitioners address cervical and thoracic spine dysfunction, including manual treatment based on level B, or moderate, evidence.^69^ The CISG guideline recommends targeted physical therapy in patients with cervical dysfunction without further details.^70^

Oculomotor vision treatment is recommended based on consensus in the guideline panel. Low-quality studies without appropriate control groups, however, do exist and report positive effects.^61,62,63,64^ The ONF guideline^5^ gives grade C, or a consensus-based recommendation, for vision assessment and treatment, whereas the APTA guideline^69^ indicates level B, or moderate, evidence. However, this guideline deals with oculomotor rehabilitation in combination with vestibular treatment, which may explain the discrepancy. The CISG guideline^70^ does not address this question.

Psychological treatment is recommended based on 9 RCTs,^42,43,54,55,56,57,58,59,60^ of which 8 were included in the meta-analysis.^42,43,54,55,56,57,58,59,60^ Tiersky et al^59^ was not included owing to insufficient reporting. Psychological interventions may be applied for multiple reasons, eg, to treat mental health disorders (primarily anxiety and depression), to address cognitive symptoms, and/or to change maladaptive behavioral strategies. Accordingly, the RCTs applied a combination of different approaches, such as psychoeducation, counseling, cognitive behavioral therapy, computer-based cognitive remediation training, and energy management. The Synthesis of Practice Guidelines^4^ emphasizes primarily an active approach to mental health disorders, with fast initiation of cognitive behavioral therapy and/or pharmacotherapy. The ONF guideline^5^ gives a grade B recommendation regarding a routine screen for depression and anxiety followed by a specialist treatment, and a grade B recommendation is given for cognitive behavioral therapy as a supplementary intervention for patients with psychological risk factors. The ONF guideline^5^ gives a grade A recommendation for neuropsychological assessment, grade B recommendation for treatment of persistent cognitive difficulties, and grade C recommendation for stress management techniques. The CISG guideline^70^ recommends cognitive behavioral therapy for persistent mood disorders or behavioral issues.

Interdisciplinary coordinated rehabilitation was recommended based on 3 RCTs.^42,43,60^ Their results indicate its usefulness regarding the primary outcome (collective burden of symptoms) as well as several secondary outcomes (Table 4). The Synthesis of Practice Guidelines^4^ highlights the need for individually tailored interdisciplinary treatment. The ONF guideline^5^ gives a grade A recommendation for evidence-based neurorehabilitation for patients with cognitive impairment and a grade B recommendation for interdisciplinary vocational assessment for those who do not resume preinjury work duties. The CISG guideline^70^ recommends a psychological, cervical, and vestibular rehabilitation performed in a collaborative manner based on detailed assessment of physical and psychosocial factors.

Even though the development of the guideline followed a rigorous methodology, our confidence in the recommendations is low or very low. This low confidence is due to scarcity and inadequacies of the underlying literature wherein studies have risk of bias due to investigating the effects of experiential interventions with difficulties related to blinding of participants and therapists. Moreover, in clinic, interventions were often used in combination as part of a multimodal approach with multiple interventions given in parallel.^10,42^

The weak recommendations call for shared decision-making, involving a discussion with the patient. People with PPCS represent a heterogeneous group regarding both their trauma-induced consequences and their premorbid functioning levels, coping strategies, and life situation in general.^71^

Expert groups have used the lack of evidence for benefit or harm for a particular intervention as an argument for not putting forward a recommendation.^72^ Such positions have, however, been met with frustration by health care professionals who look to expert groups for guidance.^73^ Fortunately, the GRADE approach accommodates these circumstances because it provides interpretations for patients, clinicians, and policy makers.^30^ Faced with either no or weak evidence, it is important that patients know that their particular preference among the various treatments should guide choice of intervention; clinicians must recognize that different interventions may be appropriate for different patients and help each patient to arrive at a management decision consistent with their values. Policy makers must involve relevant professional groups and stakeholders when determining design-of-care pathways.^30^ Importantly, guideline panels should not refrain from making recommendations because individual patients and clinicians will make different choices when faced with a weak recommendation. In fact, this is to be expected. Consequently, the GRADE Working Group encourages panels to make recommendations whenever possible whether they are based on solid evidence or not.^30^

### Strengths and Limitations

Strengths of this guideline include the broad scope informed by a need for direction in clinical practice. The guideline panel adhered rigorously to the GRADE methodology. The group was composed of experienced academics and clinicians within this research area, an expert librarian, and a skilled methodologist. The process was overseen by the Danish Health Authority, and the final product was reviewed by ^2^ external experts who provided valuable input to the final draft. Finally, the interdisciplinary reference group and the public hearing strengthened the outreach to clinical practice and health administration.

Study limitations include the fact that the guideline panel itself selected the interventions in focus. Also, we focused on adults, so the recommendations do not cover adolescents, leading to exclusion of several studies.^74,75^ Last, RCTs are not well suited to address potential harms associated with treatments; however, the guideline panel considered the included interventions to be associated with low frequency of harmful consequences.

## Conclusions

There is an urgent need for more methodologically robust research evaluating the outcomes of nonpharmacological treatments for persistent symptoms after concussion or mTBI. Given the best available evidence to date, and based on the findings of this systematic review and meta-analysis, active management and treatment of PPCS is recommended, both through individual disciplines targeting specific problems and through interdisciplinary rehabilitation. There was agreement on this recommendation across the available guidelines, including the one presented here, regardless of their applied methodology.

## Notes

Supplement. **eTable 1.** Reproduction of Search Strings by Search Engine for PICO 1-7 **eTable 2.** Clinical Questions in PICO Form, Including Specified Outcome Measures **eFigure 1.** Flow Charts With Results From the Systematic Review Process for PICO 1-7 **eTable 3.** Evaluation of Systematic Reviews Based on AMSTAR Tool **eFigure 2.** Meta-Analyses for PICO 1, 2, 3, 4, 6, 7 per Specified Outcome Measures **eTable 4.** Summary of Findings Table, Including the Rating of the Evidence and Risk of Bias Assessment for PICO 1-7 **eTable 5.** Characteristics of the Included Studies in the Systematic Review
