# Supplementary material for: Nonpharmacological Treatment of Persistent Postconcussion Symptoms in Adults: A Systematic Review and Meta-analysis and Guideline Recommendation
Source: JAMA Netw Open. 2021 Nov 9;4(11):e2132221. doi: 10.1001/jamanetworkopen.2021.32221 (PMC8579233; doi:10.1001/jamanetworkopen.2021.32221)
Supplement: Supplement. — eTable 1. Reproduction of Search Strings by Search Engine for PICO 1-7 eTable 2. Clinical Questions in PICO Form, Including Specified Outcome Measures eFigure 1. Flow Charts With Results From the Systematic Review Process for PICO 1-7 eTable 3. Evaluation of Systematic Reviews Based on AMSTAR Tool eFigure 2. Meta-Analyses for PICO 1, 2, 3, 4, 6, 7 per Specified Outcome Measures eTable 4. Summary of Findings Table, Including the Rating of the Evidence and Risk of Bias Assessment for PICO 1-7 eTable 5. Characteristics of the Included Studies in the Systematic Review [file jamanetwopen-e2132221-s001.pdf]

Supplementary Online Content

Rytter HM, Graff HJ, Henriksen HK, et al. Nonpharmacological treatment of persistent postconcussion symptoms in adults: a systematic review and meta-analysis and guideline recommendation. *JAMA Netw Open*. 2021;4(11):e2132221. doi:10.1001/jamanetworkopen.2021.32221

eTable 1. Reproduction of Search Strings by Search Engine for PICO 1-7

eTable 2. Clinical Questions in PICO Form, Including Specified Outcome Measures

eFigure 1. Flow Charts With Results From the Systematic Review Process for PICO 1-7

eTable 3. Evaluation of Systematic Reviews Based on AMSTAR Tool

eFigure 2. Meta-Analyses for PICO 1, 2, 3, 4, 6, 7 per Specified Outcome Measures

eTable 4. Summary of Findings Table, Including the Rating of the Evidence and Risk of Bias Assessment for PICO 1-7

eTable 5. Characteristics of the Included Studies in the Systematic Review

This supplementary material has been provided by the authors to give readers additional information about their work.

© 2021 Rytter HM et al. *JAMA Network Open.*

eTable 1. Reproduction of Search Strings by Search Engine for PICO 1-7

Project title	Danish National Clinical Guidelines for non-pharmacological treatment of persistent post-concussion symptoms: A systematic review and meta-analyses with recommendations for clinical practice.
Project manager	Hana Malá Rytter
Lead	Heidi Graff
Methodologist	Henriette Callesen
Search specialist	Kirsten Birkefoss, Danish Health Authority, StepChange
Last updated	20.03.2020

SEARCH STRATEGY

	**PICO 1**: Should patients, early after concussion, be offered systematically applied information and advice on preventing persistent post-concussive symptoms?<br><br>**PICO 2**: Should patients with persistent post-concussive symptoms be offered graded physical exercise?<br><br>**PICO 3**: Should patients with persistent vestibular dysfunction after concussion be offered vestibular rehabilitation?<br><br>**PICO 4**: Should patients with persistent post-concussive symptoms be offered spinal manual therapy, i.e. mobilization

© 2021 Rytter HM et al. *JAMA Network Open.*

	and manipulation of the neck and spine?<br><br>**PICO 5**: Should patients with persistent visual symptoms after concussion be offered oculomotor vision therapy?<br><br>**PICO 6:** Should patients with persistent post-concussive symptoms be offered psychological treatment?<br><br>**PICO 7:** Should patients with persistent post-concussive symptoms be offered an interdisciplinary coordinated rehabilitative treatment?
**Search terms**	See search terms in the search protocol below
**Inclusion and exclusion criterias**	Language: English, Danish, Norwegian, Swedish<br><br>Pub Year: 2000-2020<br><br>Population: Adults 18 years and older<br><br>Publ.types: Systematic reviews, meta analyses, randomized controlled trials, observational studies, intervention studies

© 2021 Rytter HM et al. *JAMA Network Open.*

Information sources

DATABASE	INTERFACE	SEARCH DATE<br>Systematic reviews<br>Meta analyses	SEARCH DATE<br>RCT<br>Observational/<br>Intervent.studies
**Medline (incl. Cochrane Reviews)**	OVID	22.11.2019–09.12.2019	03.01.2020-09.03.2020
**EMBASE (incl. Cochrane Reviews)**	OVID	22.11.2019–09.12.2019	03.01.2020-18.02.2020
**PsycINFO** (PICO 1,6,7)	OVID	22.11.2019–09.12.2019	03.01.2020-18.02.2020
**CINAHL**	EBSCO	22.11.2019–09.12.2019	03.01.2020-09.03.2020
**Cochrane Central (RCT)**	Internet		03.01.2020-18.02.2020
**PEDRO** (PICO 1,2,3,4,6,7)	Internet	22.11.2019–09.12.2019	03.01.2020-18.02.2020
**OT Seeker (**PICO 1,3,6,7)	Internet	22.11.2019–09.12.2019	03.01.2020-18.02.2020

Note

- Search terms and inclusion/exclusion criterias are adapted to the different database languages.
- Duplicates have as thoroughly as possible been removed by the help of RefWorks.
- References found has been transferred to Covidence

© 2021 Rytter HM et al. *JAMA Network Open.*

Search for systematic reviews and meta analyses

Filter used to identify systematic reviews/meta analyses

Medline

1	limit x[1)] to (systematic reviews or meta analysis)
2	(((systematic or rapid or integrative or umbrella) adj3 (review* or overview* or study or studies or search* or approach* or analys*)) or meta analy* or meta-analy* or metaanaly*).ti,bt,ab,kf.
3	(pool* adj1 (data or analys*)).ti,ab.
4	(pubmed or medline or embase or Cochrane or "web of science" or psycinfo or psychinfo or scopus).ti,ab.
5	Cochrane.jw.
6	Network meta-analysis/
7	(((multiple or mixed) adj1 (treatment* or therap*) adj1 comparison*) or (indirect adj1 comparison*)).ti,bt,ab,kf.
8	or/2-7
9	x[1)] and 8
10	1 or 9

1) x means search line for result of subject search

Embase

1	limit x[1)] to (systematic reviews or meta analysis)
2	(((systematic or rapid or integrative or umbrella) adj3 (review* or overview* or study or studies or search* or approach* or analys*)) or meta analy* or meta-analy* or metaanaly*).ti,ab,kw.
3	(pool* adj1 (data or analys*)).ti,ab.
4	(pubmed or medline or embase or Cochrane or "web of science" or psycinfo or psychinfo or scopus).ti,ab.
5	Cochrane.jx.
6	Network meta-analysis/
7	(((multiple or mixed) adj1 (treatment* or therap*) adj1 comparison*) or (indirect adj1 comparison*)).ti,ab,kw.

© 2021 Rytter HM et al. *JAMA Network Open.*

8	or/2-7
9	x[1)] and 8
10	1 or 9

1) x means search line for result of subject search

PsycINFO

1	limit x[1)] to (systematic review or meta analysis)
2	(((systematic or rapid or integrative or umbrella) adj3 (review* or overview* or study or studies or search* or approach* or analys*)) or meta analy* or meta-analy* or metaanaly*).ti,ab,id.
3	(pool* adj1 (data or analys*)).ti,ab.
4	(((multiple or mixed) adj1 (treatment* or therap*) adj1 comparison*) or (indirect adj1 comparison*)).ti,ab,id.
5	or/2-4
6	x and 5
7	1 or 6

1) x means search line for result of subject search

Cinahl

S5	S1 OR S2 OR S3 OR S4
S4	(((multiple or mixed) adj1 (treatment* or therap*) N1 comparison*) or (indirect N1 comparison*))
S3	(pool* N1 (data or analys*))
S2	(((systematic or rapid or integrative or umbrella) N3 (review* or overview* or study or studies or search* or approach*)) or meta analy* or meta-analy* or metaanaly*)
S1	PT (Systematic Review or Meta Analysis) OR MH (Systematic Review or Meta Analysis)

© 2021 Rytter HM et al. *JAMA Network Open.*

PICO 1: Should patients, early after concussion, be offered systematically applied information and advice on preventing persistent post-concussive symptoms?

Search date:

2009-2019: 22.11.2019

2008-2019: 27.11.2019

Medline

#	Searches
1	exp brain injuries, traumatic/
2	exp Brain concussion/
3	((traumatic or mild or moderat* or minor) adj3 (brain or head) adj3 (injur* or trauma)).ti,bt,ab,kw,kf.
4	(concussion* or post-concussion* or postconcussion* or post concussion).ti,bt,ab,kw,kf.
5	(concussive or post-concussive or postconcussive or post concussive or sport?-concuss* or contusion*).ti,bt,ab,kw,kf.
6	(TBI or PCS).ti,bt,ab,kw,kf.
7	or/1-6
8	Health education/
9	exp Patient Education as Topic/
10	(psychoeducat* or psycho-educat*).ti,bt,ab,kw,kf.
11	((information or educat* or guid* or counsel* or councel* or Course* or Coach* or session* or consult* or guid* or advic* or advis* or handout* or hand-out* or pamphlet* or leaflet* or booklet* or brochure*) adj10 (treatment* or therapy or intervention or management or patient* or tailor* or individual* or Patient-centred or Patient-centered or Patient-oriented or Patient-focused or Patient-based or Patient-tailored or client-cent* or client cent* or client-focus* or client focus* or Client-oriented or Client-based or Client-tailored or person-cent* or person cent* or person-focus* or person focus* or Person-oriented or Person-based or Person-tailored or Individual-centred or Individual-centered or Individual-focused or Individual-oriented or Individual-based or Individual-tailored)).ti,bt,ab,kw,kf.
12	(self-management or self-instruction* or selfmanagement or selfinstruction* or self management or self

© 2021 Rytter HM et al. *JAMA Network Open.*

	instruction* or self-guid* or self guid* or selfguid* or guidance).ti,bt,ab,kw,kf.
13	((information* or instruction* or educat*) adj3 (material or advic* or advis* or handout* or hand-out* or pamphlet* or leaflet* or booklet* or brochure* or guid*)).ti,bt,ab,kw,kf.
14	((non-pharmacological or nonpharmacological or non-drug* or psychological) adj6 (therap* or treat* or intervention* or action* or program* or strateg* or protocol* or support* or approach*)).ti,bt,kw,kf.
15	or/8-14
16	7 and 15

Embase

#	Searches
1	exp traumatic brain injury/
2	exp brain concussion/
3	exp brain contusion/
4	exp postconcussion syndrome/
5	((traumatic or mild or moderat* or minor) adj3 (brain or head) adj3 (injur* or trauma)).ti,ab,kw.
6	(concussion* or post-concussion* or postconcussion* or post concussion*).ti,ab,kw.
7	(concussive or post-concussive or postconcussive or post concussive or sport?-concuss* or contusion*).ti,ab,kw.
8	(TBI or PCS).ti,ab,kw.
9	or/1-8
10	Health education/
11	exp Patient Education/
12	exp Psychoeducation/
13	(psychoeducat* or psycho-educat*).ti,ab,kw.
14	((information or educat* or guid* or counsel* or councel* or Course* or Coach* or session* or consult* or guid* or advic* or advis* or handout* or hand-out* or pamphlet* or leaflet* or booklet* or brochure*) adj10 (treatment* or therapy or intervention or management or patient* or tailor* or individual* or Patient-centred or Patient-centered or Patient-oriented or Patient-focused or Patient-based or Patient-tailored or client-cent* or client cent* or client-focus* or client focus* or Client-oriented or Client-based or Client-tailored or person-cent* or

© 2021 Rytter HM et al. *JAMA Network Open.*

	person cent* or person-focus* or person focus* or Person-oriented or Person-based or Person-tailored or Individual-centred or Individual-centered or Individual-focused or Individual-oriented or Individual-based or Individual-tailored)).ti,ab,kw.
15	((information* or instruction* or educat*) adj3 (material or advic* or advis* or handout* or hand-out* or pamphlet* or leaflet* or booklet* or brochure* or guid*)).ti,ab,kw.
16	(self-management or self-instruction* or selfmanagement or selfinstruction* or self management or self instruction* or self-guid* or self guid* or selfguid* or guidance).ti,ab,kw.
17	((non-pharmacological or nonpharmacological or non-drug* or psychological) adj6 (therap* or treat* or intervention* or action* or program* or strateg* or protocol* or support* or approach*)).ti,kw.
18	or/10-17
19	9 and 18

PsycINFO

#	Searches
1	exp traumatic brain injury/
2	exp brain concussion/
3	((traumatic or mild or moderat* or minor) adj3 (brain or head) adj3 (injur* or trauma)).ti,ab,id.
4	(concussion* or post-concussion* or postconcussion* or post concussion*).ti,ab,id.
5	(concussive or post-concussive or postconcussive or post concussive or sport?-concuss* or contusion*).ti,ab,id.
6	(TBI or PCS).ti,ab,id.
7	or/1-6
8	Health education/
9	exp Client Education/
10	exp Psychoeducation/
11	(psychoeducat* or psycho-educat*).ti,ab,id.
12	((information or educat* or guid* or counsel* or councel* or Course* or Coach* or session* or consult* or guid* or advic* or advis* or handout* or hand-out* or pamphlet* or leaflet* or booklet* or brochure*) adj10 (treatment* or therapy or intervention or management or patient* or tailor* or individual* or Patient-centred or Patient-centered or Patient-oriented or Patient-focused or Patient-based or Patient-tailored or client-cent* or client cent* or client-focus* or client focus* or Client-oriented or Client-based or Client-tailored or person-cent* or

© 2021 Rytter HM et al. *JAMA Network Open.*

	person cent* or person-focus* or person focus* or Person-oriented or Person-based or Person-tailored or Individual-centred or Individual-centered or Individual-focused or Individual-oriented or Individual-based or Individual-tailored)).ti,ab,id.
13	((information* or instruction* or educat*) adj3 (material or advic* or advis* or handout* or hand-out* or pamphlet* or leaflet* or booklet* or brochure* or guid* or support*)).ti,ab,id.
14	(self-management or self-instruction* or selfmanagement or selfinstruction* or self management or self instruction* or self-guid* or self guid* or selfguid* or guidance).ti,ab,id.
15	((non-pharmacological or nonpharmacological or non-drug* or psychological) adj6 (therap* or treat* or intervention* or action* or program* or strateg* or protocol* or support* or approach*)).ti,id.
16	or/9-15
17	7 and 16

Cinahl

#	Query
S19	S8 AND S18
S18	S9 OR S10 OR S11 OR S12 OR S13 OR S14 OR S15 OR S16 OR S17
S17	((non-pharma* or nonpharma* or non pharma* or non-drug* or psychological) N6 (therap* or treat* or intervention* or action* or program* or strateg* or protocol* or support* or approach*))
S16	((information* or instruction* or educat*) N3 (material or advic* or advis* or handout* or hand-out* or pamphlet* or leaflet* or booklet* or brochure* or guid*))
S15	(self-management or self-instruction* or selfmanagement or selfinstruction* or self management or self instruction* or self-guid* or self guid* or selfguid* or guidance)
S14	((information or educat* or guid* or counsel* or councel* or Course* or Coach* or session* or consult* or guid* or advic* or advis* or handout* or hand-out* or pamphlet* or leaflet* or booklet* or brochure*) N10 (treatment* or therapy or intervention or management or patient* or tailor* or individual* or Patient-centred or Patient-centered or Patient-oriented or Patient-focused or Patient-based or Patient-tailored or client-cent* or client cent* or client-focus* or client focus* or Client-oriented or Client-based or Client-tailored or person-cent* or person cent* or person-focus* or person focus* or Person-oriented or Person-based or Person-tailored or Individual-centred or Individual-centered or Individual-focused or Individual-oriented or Individual-based or Individual-tailored))
S13	(psychoeducat* or psycho-educat*)
S12	(MH "Psychoeducation")

© 2021 Rytter HM et al. *JAMA Network Open.*

S11	(MH "Patient Discharge Education")
S10	(MH "Patient Education")
S9	MH "Health education"
S8	S1 OR S2 OR S3 OR S4 OR S5 OR S6 OR S7
S7	TBI or PCS
S6	(concussive or post-concussive or postconcussive or post concussive or sport?-concuss* or contusion*)
S5	(concussion* or post-concussion* or postconcussion* or post concussion)
S4	((traumatic or mild or moderat* or minor) N3 (brain or head) N3 (injur* or trauma))
S3	MH "Brain Contusions"
S2	(MH "Brain Injuries")
S1	(MH "Brain Concussion+")

PEDRO

PICOs searched combined

Abstract & Title: Concussi* OR post-concussi* OR postconcussi* OR "traumatic brain injury" OR "traumatic head injury"

Method: Systematic review

Published since: 2000

OT SEEKER

PICOs searched combined

© 2021 Rytter HM et al. *JAMA Network Open.*

Any Field ("traumatic brain injur*" OR "traumatic head injur*" OR concussi* OR post-concussi* OR postconcussi* or "post concussi*" or sports-concuss* or contusion*)

© 2021 Rytter HM et al. *JAMA Network Open.*

PICO 2: Should patients with persistent post-concussive symptoms be offered graded physical exercise?

Search date: 29.11.2019

Medline

#	Searches
1	exp brain injuries, traumatic/
2	exp Brain concussion/
3	((traumatic or mild or moderat* or minor) adj3 (brain or head) adj3 (injur* or trauma)).ti,bt,ab,kw,kf.
4	(concussion* or post-concussion* or postconcussion* or post concussion).ti,bt,ab,kw,kf.
5	(concussive or post-concussive or postconcussive or post concussive or sport?-concuss* or contusion*).ti,bt,ab,kw,kf.
6	(TBI or PCS).ti,bt,ab,kw,kf.
7	or/1-6
8	exp exercise/
9	exp exercise therapy/
10	exp Sports/
11	exp Physical Exertion/
12	exp Muscle strength/
13	exp Exercise Movement Techniques/
14	exp "Physical Education and Training"/
15	exp Hydrotherapy/
16	Weight-Bearing/
17	Tai Ji/
18	Yoga/
19	physical therapy modalities/

© 2021 Rytter HM et al. *JAMA Network Open.*

20	(exercis* or train* or stepped care or physical therap* or physiotherap* or kinesiotherapy).ti,bt,kw,kf.
21	((muscl* or resistance or physical or endurance or strength* or balance or aquatic* or trunk or graded or gradual or controlled or incremented or symptom-based or symptombased or symptom based or guided or supervis* or stepped care) adj3 (train* or retrain* or re-train* or exercise* or strength* or fit* or condition* or exposure or activit* or load* or physical therap* or physiotherap*)).ti,bt,ab,kw,kf.
22	(aerobic* or anaerobic* or movement therapy or watergym* or water gym* or aquagym or gym* or hydrotherap* or (warm water adj4 (exercis* or train* or rehab))).ti,bt,ab,kw,kf.
23	(running or Skate* or skating or skiing or nordic walking or Jog* or zumba or kettlebelt or Swim* or Bicycl* or cycling or bicycling or spinning or walk* or rowing or pacing or paced or titreted or soccer or ball game* or handball or basketball or athletic* or (play* adj2 ball) or (weight* adj3 (lift* or train* or exercis*))).ti,bt,ab,kw,kf.
24	(yoga or tai ji or tai chi or qigong or qi-gong or pilates).ti,bt,ab,kw,kf.
25	((mckenzie or mechanical or Alexander or William or Feldenkrais) adj2 (technique* or model* or tool* or exercise* or therapy)).ti,bt,ab,kw,kf.
26	((non-pharmacological or nonpharmacological or non-drug or nondrug) adj6 (therap* or treat* or intervention* or action* or program* or strateg* or protocol* or support* or approach*)).ti,bt,kw,kf.
27	or/8-26
28	7 and 27

Embase

#	Searches
1	exp traumatic brain injury/
2	exp Brain concussion/
3	exp brain contusion/
4	exp postconcussion syndrome/
5	((traumatic or mild or moderat* or minor) adj3 (brain or head) adj3 (injur* or trauma)).ti,ab,kw.
6	(concussion* or post-concussion* or postconcussion* or post concussion).ti,ab,kw.
7	(concussive or post-concussive or postconcussive or post concussive or sport?-concuss* or contusion*).ti,ab,kw.
8	(TBI or PCS).ti,ab,kw.

© 2021 Rytter HM et al. *JAMA Network Open.*

9	or/1-8
10	exp exercise/
11	exp kinesiotherapy/
12	exp Sports/
13	exp Physical Exertion/
14	exp Muscle strength/
15	exp Exercise Movement Techniques/
16	exp "Physical Education and Training"/
17	exp Hydrotherapy/
18	Weight-Bearing/
19	Tai Ji/
20	Yoga/
21	physical therapy modalities/
22	(exercis* or train* or stepped care or physical therap* or physiotherap* or kinesiotherapy).ti,kw.
23	((muscl* or resistance or physical or endurance or strength* or balance or aquatic* or trunk or graded or gradual or controlled or incremented or symptom-based or symptombased or symptom based or guided or supervis*) adj3 (train* or retrain* or re-train* or exercise* or strength* or fit* or condition* or exposure or activit* or load* or physical therap* or physiotherap*)).ti,ab,kw.
24	(aerobic* or anaerobic* or movement therapy or watergym* or water gym* or aquagym or gym* or hydrotherap* or (warm water adj4 (exercis* or train* or rehab))).ti,ab,kw.
25	(running or Skate* or skating or skiing or nordic walking or Jog* or zumba or kettlebelt or Swim* or Bicycl* or cycling or bicycling or spinning or walk* or rowing or pacing or paced or titreted or soccer or ball game* or handball or basketball or athletic* or (play* adj2 ball) or (weight* adj3 (lift* or train* or exercis*))).ti,ab,kw.
26	(yoga or tai ji or tai chi or qigong or qi-gong or pilates).ti,ab,kw.
27	((mckenzie or mechanical or Alexander or William or Feldenkrais) adj2 (technique* or model* or tool* or exercise* or therapy)).ti,ab,kw.
28	((non-pharmacological or nonpharmacological or non-drug or nondrug) adj6 (therap* or treat* or intervention* or action* or program* or strateg* or protocol* or support* or approach*)).ti,kw.
29	or/10-28

© 2021 Rytter HM et al. *JAMA Network Open.*

30	9 and 29

Cinahl

#	Query
S19	S8 AND S18
S18	S9 OR S10 OR S11 OR S12 OR S13 OR S14 OR S15 OR S16 OR S17
S17	(running or Skate* or skating or skiing or nordic walking or Jog* or zumba or kettlebelt or Swim* or Bicycl* or cycling or bicycling or spinning or walk* or rowing or pacing or paced or titreted or soccer or ball game* or handball or basketball or athletic* or weight-bearing or (play* N2 ball) or (weight* N3 (lift* or train* or exercis* or bearing)))
S16	(aerobic* or anaerobic* or movement therapy or watergym* or water gym* or aquagym or gym* or hydrotherap* or (warm water N4 (exercis* or train* or rehab)) or yoga or tai ji or tai chi or qigong or qi-gong or pilates)
S15	((muscl* or resistance or physical or endurance or strength* or balance or aquatic* or trunk or graded or gradual or controlled or incremented or symptom-based or symptombased or symptom based or guided or supervis* or stepped care) N3 (train* or retrain* or re-train* or exercise* or strength* or fit* or condition* or exposure or activit* or load* or physical therap* or physiotherap*))
S14	(exercis* or train* or stepped care or physical therap* or physiotherap* or kinesiotherapy)
S13	(non-pharmacological or nonpharmacological or non-drug or nondrug) N6 (therap* or treat* or intervention* or action* or program* or strateg* or protocol* or support* or approach*))
S12	(mckenzie or mechanical or Alexander or William or Feldenkrais) N2 (technique* or model* or tool* or exercise* or therapy)
S11	MH Sports+ or MH "tai chi" or MH Yoga or MH "Weight-bearing"
S10	(MH "Physical Therapy") OR (MH "Constraint-Induced Therapy") OR (MH "Functional Training") OR (MH "Gait Training+") OR (MH "Home Physical Therapy") OR (MH "Hydrotherapy+") OR (MH "Therapeutic Exercise+")
S9	MH Exercise+
S8	S1 OR S2 OR S3 OR S4 OR S5 OR S6 OR S7
S7	TBI or PCS

© 2021 Rytter HM et al. *JAMA Network Open.*

S6	(concussive or post-concussive or postconcussive or post concussive or sport#-concuss* or contusion*)
S5	(concussion* or post-concussion* or postconcussion* or post concussion)
S4	((traumatic or mild or moderat* or minor) N3 (brain or head) N3 (injur* or trauma))
S3	MH "Brain Contusions"
S2	(MH "Brain Injuries")
S1	(MH "Brain Concussion+")

PEDRO

PICOs searched combined

Abstract & Title: Concussi* OR post-concussi* OR postconcussi* OR "traumatic brain injury" OR "traumatic head injury"

Method: Systematic review

Published since: 2000

OT Seeker

PICOs searched combined

Any Field ("traumatic brain injur*" OR "traumatic head injur*" OR concussi* OR post-concussi* OR postconcussi* or "post concussi*" or sports-concuss* or contusion*)

PICO 3: Should patients with persistent vestibular dysfunction after concussion be offered vestibular rehabilitation?

Search date: 29.11.2019

© 2021 Rytter HM et al. *JAMA Network Open.*

Medline

#	Searches
1	exp brain injuries, traumatic/
2	exp brain concussion/
3	((traumatic or mild or moderat* or minor) adj3 (brain or head) adj3 (injur* or trauma)).ti,bt,ab,kw,kf.
4	(concussion* or post-concussion* or postconcussion* or post concussion*).ti,bt,ab,kw,kf.
5	(concussive or post-concussive or postconcussive or post concussive or sport?-concuss* or contusion*).ti,bt,ab,kw,kf.
6	(TBI or PCS).ti,bt,ab,kw,kf.
7	or/1-6
8	exp Vestibular Diseases/
9	exp vertigo/
10	dizziness/ or perilymph/ and fistula/
11	(vertigo or dizzyness or dizziness or vestibulopath* or ((vestibular or balance*) adj6 (disorder or hypofunction* or dysfunction* or impair* or disability* or pathology* or disturbance*))).ti,bt,ab,kw,kf.
12	(vestibular adj6 (rehabilitation or adaptation or habituation)).ti,bt,ab,kw,kf.
13	(neurolabyrinthitides or neurolabyrinthitis or (vestibular and (neuritis or neuronitis or neuritides))).ti,bt,ab,kw,kf.
14	((acoustic adj neuroma) or (acoustic adj neurinoma) or (acoustic adj neurilemoma) or (acoustic adj neurilemmoma) or (vestibular adj schwannoma) or (acoustic adj schwannoma) or (motion adj sensitivity) or (vestibular and peripheral) or (perilymphatic and fistula) or meniere* or (endolymphatic and hydrops) or (labyrinth* and hydrops) or (labyrinth* and syndrome) or bpv or bppv or antbppv).ti,bt,ab,kw,kf.
15	((non-pharmacological or nonpharmacological or non-drug or nondrug) adj6 (therap* or treat* or intervention* or action* or program* or strateg* or protocol* or support* or approach*)).ti,kw.
16	or/8-15
17	Rehabilitation, Vocational/
18	exp Exercise therapy/
19	exp exercise/

© 2021 Rytter HM et al. *JAMA Network Open.*

20	exp head movement/
21	exp physical therapy modalities/
22	(rehabilitation or physiotherap* or (physical and therap*) or exercis* or habituat* or epley or canalith or semont or manoeuvre* or maneuver* or (reconditioning adj activit*) or posturography or ((posture or postural or sensory) adj6 (control* or learn* or relearn* or train* or retrain* or exercise)) or pfpp).ti,bt,ab,kw,kf.
23	((position* and procedure*) or (reposition* and procedure*) or (reposition* and particle*) or (visual and vestibular) or (functional and retrain*) or (occupational and retrain*) or (occupational and adaptation) or (cooksey and cawthorne)).ti,bt,ab,kw,kf.
24	or/17-23
25	7 and 16 and 24

Embase

#	Searches
1	exp traumatic brain injury/
2	exp brain concussion/
3	exp brain contusion/
4	exp postconcussion syndrome/
5	((traumatic or mild or moderat* or minor) adj3 (brain or head) adj3 (injur* or trauma)).ti,ab,kw.
6	(concussion* or post-concussion* or postconcussion* or post concussion*).ti,ab,kw.
7	(concussive or post-concussive or postconcussive or post concussive or sport?-concuss* or contusion*).ti,ab,kw.
8	(TBI or PCS).ti,ab,kw.
9	or/1-8
10	exp vestibular disorder/
11	exp vertigo/
12	dizziness/
13	perilymph/ and fistula/
14	(vertigo or dizzyness or dizziness or vestibulopath* or ((vestibular or balance*) adj6 (disorder or hypofunction* or dysfunction* or impair* or disability* or pathology* or disturbance*))).ti,ab,kw.

© 2021 Rytter HM et al. *JAMA Network Open.*

15	(vestibular adj6 (rehabilitation or adaptation or habituation)).ti,ab,kw.
16	(neurolabyrinthitides or neurolabyrinthitis or (vestibular and (neuritis or neuronitis or neuritides))).ti,ab,kw.
17	((acoustic adj neuroma) or (acoustic adj neurinoma) or (acoustic adj neurilemoma) or (acoustic adj neurilemmoma) or (vestibular adj schwannoma) or (acoustic adj schwannoma) or (motion adj sensitivity) or (vestibular and peripheral) or (perilymphatic and fistula) or meniere* or (endolymphatic and hydrops) or (labyrinth* and hydrops) or (labyrinth* and syndrome) or bpv or bppv or antbppv).ti,ab,kw.
18	or/10-17
19	vocational rehabilitation/
20	exp kinesiotherapy/
21	exp exercise/
22	exp head movement/
23	exp physical therapy modalities/
24	(rehabilitation or physiotherap* or (physical and therap*) or exercis* or habituat* or epley or canalith or semont or manoeuvre* or maneuver* or (reconditioning adj activit*) or posturography or ((posture or postural or sensory) adj6 (control* or learn* or relearn* or train* or retrain* or exercise)) or pfpp).ti,ab,kw.
25	((position* and procedure*) or (reposition* and procedure*) or (reposition* and particle*) or (visual and vestibular) or (functional and retrain*) or (occupational and retrain*) or (occupational and adaptation) or (cooksey and cawthorne)).ti,ab,kw.
26	or/19-25
27	9 and 18 and 26

Cinahl

#	Query
S19	S8 AND S18
S18	S9 OR S10 OR S11 OR S12 OR S13 OR S14 OR S15 OR S16 OR S17
S17	((non-pharma* or nonpharma* or non pharma* or non-drug* or psychological) N6 (therap* or treat* or intervention* or action* or program* or strateg* or protocol* or support* or approach*))
S16	((acoustic N1 neuroma) or (acoustic N1 neurinoma) or (acoustic N1 neurilemoma) or (acoustic N1 neurilemmoma) or (vestibular N1 schwannoma) or (acoustic N1 schwannoma) or (motion N1 sensitivity) or (vestibular and peripheral) or (perilymphatic and fistula) or meniere* or (endolymphatic and hydrops) or

© 2021 Rytter HM et al. *JAMA Network Open.*

	(labyrinth* and hydrops) or (labyrinth* and syndrome) or bpv or bppv or antbppv)
S15	(neurolabyrinthitides or neurolabyrinthitis or (vestibular and (neuritis or neuronitis or neuritides)))
S14	(vestibular N6 (rehabilitation or adaptation or habituation))
S13	(vertigo or dizzyness or dizziness or vestibulopath* or ((vestibular or balance*) N6 (disorder or hypofunction* or dysfunction* or impair* or disability* or pathology* or disturbance*)))
S12	(MH "perilymph") AND (MH "fistula")
S11	MH dizziness
S10	(MH "Vertigo")
S9	MH "Vestibular Diseases"
S8	S1 OR S2 OR S3 OR S4 OR S5 OR S6 OR S7
S7	TBI or PCS
S6	(concussive or post-concussive or postconcussive or post concussive or sport#-concuss* or contusion*)
S5	(concussion* or post-concussion* or postconcussion* or post concussion)
S4	((traumatic or mild or moderat* or minor) N3 (brain or head) N3 (injur* or trauma))
S3	MH "Brain Contusions"
S2	(MH "Brain Injuries")
S1	(MH "Brain Concussion+")

PEDRO

PICOs searched combined

<u>Abstract & Title</u>: Concussi* OR post-concussi* OR postconcussi* OR "traumatic brain injury" OR "traumatic head injury"

© 2021 Rytter HM et al. *JAMA Network Open.*

Method: Systematic review

Published since: 2000

OT Seeker

PICOs searched combined

Any Field ("traumatic brain injur*" OR "traumatic head injur*" OR concussi* OR post-concussi* OR postconcussi* or "post concussi*" or sports-concuss* or contusion*)

© 2021 Rytter HM et al. *JAMA Network Open.*

PICO 4: Should patients with persistent post-concussive symptoms be offered spinal manual therapy, i.e. mobilization and manipulation of the neck and spine?

Search date: 02.12.2019

Medline

#	Searches
1	exp brain injuries, traumatic/
2	exp Brain concussion/
3	((traumatic or mild or moderat* or minor) adj3 (brain or head) adj3 (injur* or trauma)).ti,bt,ab,kw,kf.
4	(concussion* or post-concussion* or postconcussion* or post concussion).ti,bt,ab,kw,kf.
5	(concussive or post-concussive or postconcussive or post concussive or sports-concuss* or contusion*).ti,bt,ab,kw,kf.
6	(TBI or PCS).ti,bt,ab,kw,kf.
7	or/1-6
8	Musculoskeletal Manipulations/
9	Manipulation, Chiropractic/
10	Manipulation, Orthopedic/
11	Manipulation, Osteopathic/
12	Manipulation, Spinal/
13	(adjust* adj6 (chiropract* or spinal or lumbar or cervical or thoracic or instrument* or tool* or electric)).ti,bt,ab,kf.
14	(HVLA or high velocity low amplitude).ti,bt,ab,kw.
15	(manipulat* and (chiropract* or naprapath* or osteopath* or orthopedic* or orthopaedic*)).ti,bt,ab,kf.
16	(manipulat* and (spinal or spine or low* back or joint* or lumbar or neck or thoracic or cervical or MSK or musculoskeletal or vertebr*)).ti,bt,ab,kf.
17	(manipulat* and (physiotherap* or physical therap*)).ti,bt,ab,kf.
18	(manipulat* and technique*).ti,bt,ab,kw,kf.

© 2021 Rytter HM et al. *JAMA Network Open.*

19	(mobili?at* and (chiropract* or naprapath* or osteopath* or orthopedic* or orthopaedic*)).ti,bt,ab,kf.
20	(mobili?at* and (spinal or spine or low* back or joint* or lumbar or neck or thoracic or cervical or MSK or musculoskeletal or vertebr*)).ti,bt,ab,kf.
21	((therap* or treat* or intervention* or manag*) and (manual or manipulat* or mobili?at* or MSK or musculoskeletal)).ti,bt,ab,kf.
22	(traction adj3 (manual or passive or mechanical or non-surgical or nonsurgical)).ti,bt,ab,kf.
23	(flexion-distraction or flexion distraction).ti,bt,ab,kf.
24	(activator adj3 (method* or technique* or instrument*)).ti,bt,ab,kf.
25	((mechanical or Alexander or Atlas Orthogonal or Atlas Specific or Bilateral Nasal Specific or Blair Upper Cervical or Chiropractic Manipulative Reflex or Cox or Derifield-Thompson or Directional Non-Force or Diversified or Endo-Nasal or feldenkrais or William or Flexion-Distraction or Full Spine or Gonstead or Grostic or Harrison or Koren Specific or Logan Basic or McTimoney or Maitland or Mulligan or muligan or Neuro Emotional or NUCCA or Palmer or Pierce-Stillwagon or Sacro Occipital or Thompson Terminal Point or Toftness or Toggle Recoil or Torque Release or Webster or muscle energy) adj1 (Technique* or model* or tool* or exercise* or therapy)).ti,bt,ab,kw,kf.
26	(reflexolog* or cyriax or High velocity or low amplitude or Subluxation or Joint dysfunction or hypomobility or muscle energy technique* or therapeutic touch or stretching).ti,bt,ab,kw,kf.
27	(vibration adj5 (therap* or treatment*)).ti,bt,ab,kw,kf.
28	((non-pharmacological or nonpharmacological or non-drug or nondrug or non-surgical or nonsurgical or non surgical or non-surgery or nonsurgery or non surgery) adj6 (therap* or treat* or intervention* or action* or program* or strateg* or protocol* or support* or approach*)).ti,bt,kw,kf.
29	or/8-28
30	7 and 29

Embase

#	Searches
1	exp traumatic brain injury/
2	exp Brain concussion/
3	exp brain contusion/
4	exp postconcussion syndrome/
5	((traumatic or mild or moderat* or minor) adj3 (brain or head) adj3 (injur* or trauma)).ti,ab,kw.

© 2021 Rytter HM et al. *JAMA Network Open.*

6	(concussion* or post-concussion* or postconcussion* or post concussion).ti,ab,kw.
7	(concussive or post-concussive or postconcussive or post concussive or sports-concuss* or contusion*).ti,ab,kw.
8	(TBI or PCS).ti,ab,kw.
9	or/1-8
10	exp musculoskeletal manipulation/
11	(adjust* adj6 (chiropract* or spinal or lumbar or cervical or thoracic or instrument* or tool* or electric)).ti,ab,kw.
12	(HVLA or high velocity low amplitude).ti,ab,kw.
13	(manipulat* and (chiropract* or naprapath* or oesteopath* or osteopath* or orthopedic* or orthopaedic*)).ti,ab,kw.
14	(manipulat* and (spinal or spine or low* back or joint* or lumbar or neck or thoracic or cervical or MSK or musculoskeletal or vertebr*)).ti,ab,kw.
15	(manipulat* and (physiotherap* or physical therap*)).ti,ab,kw.
16	(manipulat* and technique*).ti,ab,kw.
17	(mobili?at* and (chiropract* or naprapath* or osteopath* or orthopedic* or orthopaedic*)).ti,ab,kw.
18	(mobili?at* and (spinal or spine or low* back or joint* or lumbar or neck or thoracic or cervical or MSK or musculoskeletal or vertebr*)).ti,ab,kw.
19	((therap* or treat* or intervention* or manag*) and (manual or manipulat* or mobili?at* or MSK or musculoskeletal)).ti,ab,kw.
20	(traction adj3 (manual or passive or mechanical or non-surgical or nonsurgical)).ti,ab,kw.
21	(flexion-distraction or flexion distraction).ti,ab,kw.
22	(activator adj3 (method* or technique* or instrument*)).ti,ab,kw.
23	((mechanical or Alexander or Atlas Orthogonal or Atlas Specific or Bilateral Nasal Specific or Blair Upper Cervical or Chiropractic Manipulative Reflex or Cox or Derifield-Thompson or Directional Non-Force or Diversified or Endo-Nasal or feldenkrais or William or Flexion-Distraction or Full Spine or Gonstead or Grostic or Harrison or Koren Specific or Logan Basic or McTimoney or Maitland or Mulligan or muligan or Neuro Emotional or NUCCA or Palmer or Pierce-Stillwagon or Sacro Occipital or Thompson Terminal Point or Toftness or Toggle Recoil or Torque Release or Webster or muscle energy) adj1 (Technique* or model* or tool* or exercise* or therapy)).ti,ab,kw.
24	(reflexolog* or cyriax or High velocity or low amplitude or Subluxation or Joint dysfunction or hypomobility or muscle energy technique* or therapeutic touch or stretching).ti,ab,kw.
25	(vibration adj5 (therap* or treatment*)).ti,ab,kw.

© 2021 Rytter HM et al. *JAMA Network Open.*

26	((non-pharmacological or nonpharmacological or non-drug or nondrug or non-surgical or nonsurgical or non surgical or non-surgery or nonsurgery or non surgery) adj6 (therap* or treat* or intervention* or action* or program* or strateg* or protocol* or support* or approach*)).ti,kw.
27	or/10-26
28	9 and 27

© 2021 Rytter HM et al. *JAMA Network Open.*

Cinahl

#	Query
S19	S8 AND S18
S18	S9 OR S10 OR S11 OR S12 OR S13 OR S14 OR S15 OR S16 OR S17
S17	((non-pharmacological or nonpharmacological or non-drug or nondrug or non-surgical or nonsurgical or non surgical or non-surgery or nonsurgery or non surgery) N6 (therap* or treat* or intervention* or action* or program* or strateg* or protocol* or support* or approach*))
S16	((reflexolog* or cyriax or High velocity or low amplitude or Subluxation or Joint dysfunction or hypomobility or muscle energy technique* or therapeutic touch or stretching)) or (vibration N5 (therap* or treatment*))
S15	((mechanical or Alexander or Atlas Orthogonal or Atlas Specific or Bilateral Nasal Specific or Blair Upper Cervical or Chiropractic Manipulative Reflex or Cox or Derifield-Thompson or Directional Non-Force or Diversified or Endo-Nasal or feldenkrais or William or Flexion-Distraction or Full Spine or Gonstead or Grostic or Harrison or Koren Specific or Logan Basic or McTimoney or Maitland or Mulligan or muligan or Neuro Emotional or NUCCA or Palmer or Pierce-Stillwagon or Sacro Occipital or Thompson Terminal Point or Toftness or Toggle Recoil or Torque Release or Webster or muscle energy) N1 (Technique* or model* or tool* or exercise* or therapy))
S14	((therap* or treat* or intervention* or manag*) and (manual or manipulat* or mobili?at* or MSK or musculoskeletal)) or ((traction N3 (manual or passive or mechanical or non-surgical or nonsurgical))) or (activator N3 (method* or technique* or instrument*))
S13	(mobili#at* and (chiropract* or naprapath* or osteopath* or oesteopath* or orthopedic* or orthopaedic* or spinal or spine or low* back or joint* or lumbar or neck or thoracic or cervical or MSK or musculoskeletal or vertebr*))
S12	(manipulat* and (chiropract* or naprapath* or osteopath* or oesteopath* or orthopedic* or orthopaedic* or spinal or spine or low* back or joint* or lumbar or neck or thoracic or cervical or MSK or musculoskeletal or vertebr* or physiotherap* or physical therap* or technique*))
S11	(HVLA or high velocity low amplitude or flexion-distraction or flexion distraction)
S10	(adjust* N6 (chiropract* or spinal or lumbar or cervical or neck* or thoracic or instrument* or tool* or electric))
S9	MH "Manual therapy+"
S8	S1 OR S2 OR S3 OR S4 OR S5 OR S6 OR S7
S7	TBI or PCS

© 2021 Rytter HM et al. *JAMA Network Open.*

S6	(concussive or post-concussive or postconcussive or post concussive or sport#-concuss* or contusion*)
S5	(concussion* or post-concussion* or postconcussion* or post concussion)
S4	((traumatic or mild or moderat* or minor) N3 (brain or head) N3 (injur* or trauma))
S3	MH "Brain Contusions"
S2	(MH "Brain Injuries")
S1	(MH "Brain Concussion+")

PEDRO

PICOs searched combined

Abstract & Title: Concussi* OR post-concussi* OR postconcussi* OR "traumatic brain injury" OR "traumatic head injury"

Method: Systematic review

Published since: 2000

OT Seeker

PICOs searched combined

Any Field ("traumatic brain injur*" OR "traumatic head injur*" OR concussi* OR post-concussi* OR postconcussi* or "post concussi*" or sports-concuss* or contusion*)

PICO 5: Should patients with persistent visual symptoms after concussion be offered oculomotor vision therapy?

Search date: 04.12.2019

Medline

#	Searches
1	exp brain injuries, traumatic/
2	exp brain concussion/
3	((traumatic or mild or moderat* or minor) adj3 (brain or head) adj3 (injur* or trauma)).ti,bt,ab,kw,kf.
4	(concussion* or post-concussion* or postconcussion* or post concussion*).ti,bt,ab,kw,kf.
5	(concussive or post-concussive or postconcussive or post concussive or sport?-concuss* or contusion*).ti,bt,ab,kw,kf.
6	(TBI or PCS).ti,bt,ab,kw,kf.
7	or/1-6
8	exp Visual Perception/
9	exp Eye Movements/
10	Visual cortex/
11	Evoked Potentials, Visual/
12	exp Eye Manifestations/
13	exp Visual pathways/
14	Oculomotor Nerve Injuries/
15	Reflex, Vestibulo-Ocular/
16	Orthoptics/
17	((vision or visual or visual-evoked or visual-related or vision-evoked or vision-related or optical or binocular or convergence or vergence or saccadic or oculomotor* or oculo-motor* or accommodative or eye movement* or reading) adj6 (therap* or treat* or exercise* or train* or learn* or relearn* or rehabilitat* or neuro-rehabilitation or neurorehabilitation or coordination or attention or potential* or active* or dysfunction* or insufficien* or disorder* or defici* or symptom* or impairment* or injur*)).ti,bt,ab,kw,kf.

© 2021 Rytter HM et al. *JAMA Network Open.*

18	(Orthoptic* or Vergence or accommodation or eye movement* or Eye Manifestations or visual pathway* or visual nervous system or visual perception or visual cortex or "V1" or Striate Cortex or Extrastriate Cortex or oculomotor system or oculo-motor system or Vestibo-Ocular reflex* or Vestibuloocular or "Near point of convergence" or Convergence amplitude* or Fusional vergence or fusional reserve* or Vergence facility or Vergence flexibility or Stereopsis or Stereoacuity or "Amplitude of accommodation" or Accommodative amplitude* or "facility of accommodation" or Accommodative facility or Developmental Eye Movement test or "Visual search and Attention Test" or Convergence insufficiency symptom survey or Vergence latency or Vergence peek velocity or Vergence accuracy or Accommodative latency or Accommodative peek velocity or Accommodative accuracy or Eye tracking or Versional peek velocity or Fixation stability or Versional latency or VEP).ti,bt,ab,kw,kf.
19	((non-pharmacological or nonpharmacological or non-drug or nondrug or non-surgical or nonsurgical or non surgical or non-surgery or nonsurgery or non surgery) adj6 (therap* or treat* or intervention* or action* or program* or strateg* or protocol* or support* or approach*)).ti,bt,kw,kf.
20	or/8-19
21	7 and 20

Embase

#	Searches
1	exp traumatic brain injury/
2	exp Brain concussion/
3	exp brain contusion/
4	exp postconcussion syndrome/
5	((traumatic or mild or moderat* or minor) adj3 (brain or head) adj3 (injur* or trauma)).ti,ab,kw.
6	(concussion* or post-concussion* or postconcussion* or post concussion).ti,ab,kw.
7	(concussive or post-concussive or postconcussive or post concussive or sports-concuss* or contusion*).ti,ab,kw.
8	(TBI or PCS).ti,ab,kw.
9	or/1-8
10	exp Binocular vision/ or exp Eye Movements/
11	Visual cortex/
12	Visual Evoked Potential/
13	exp Eye Manifestations/

© 2021 Rytter HM et al. *JAMA Network Open.*

14	exp Oculomotor Nerve Injury/
15	exp visual nervous system/
16	Vestibuloocular Reflex/
17	Orthoptics/
18	((vision or visual or visual-evoked or visual-related or vision-evoked or vision-related or optical or binocular or convergence or vergence or saccadic or oculomotor* or oculo-motor* or accommodative or eye movement* or reading) adj6 (therap* or treat* or exercise* or train* or learn* or relearn* or rehabilitat* or neuro-rehabilitation or neurorehabilitation or coordinat* or attention or potential* or active* or dysfunction* or insufficien* or disorder* or defici* or symptom* or impairment* or injur*)).ti,ab,kw.
19	(Orthoptic* or Vergence or accommodation or eye movement* or Eye Manifestations or visual pathway* or visual nervous system or vestibular ocular response or visual cortex or "V1" or Striate Cortex or Extrastriate Cortex or oculomotor system or oculo-motor system or Vestibo-Ocular reflex* or Vestibuloocular or "Near point of convergence" or Convergence amplitude* or Fusional vergence or fusional reserve* or Vergence facility or Vergence flexibility or Stereopsis or Stereoacuity or "Amplitude of accommodation" or Accommodative amplitude* or "facility of accommodation" or Accommodative facility or Developmental Eye Movement test or "Visual search and Attention Test" or Convergence insufficiency symptom survey or Vergence latency or Vergence peek velocity or Vergence accuracy or Accommodative latency or Accommodative peek velocity or Accommodative accuracy or Eye tracking or Versional peek velocity or Fixation stability or Versional latency or VEP).ti,ab,kw.
20	((non-pharmacological or nonpharmacological or non-drug or nondrug or non-surgical or nonsurgical or non surgical or non-surgery or nonsurgery or non surgery) adj6 (therap* or treat* or intervention* or action* or program* or strateg* or protocol* or support* or approach*)).ti,kw.
21	or/10-20
22	9 and 21

Cinahl

#	Query
S16	S8 AND S15
S15	S9 OR S10 OR S11 OR S12 OR S13 OR S14
S14	((non-pharmacological or nonpharmacological or non-drug or nondrug or non-surgical or nonsurgical or non surgical or non-surgery or nonsurgery or non surgery) N6 (therap* or treat* or intervention* or action* or program* or strateg* or protocol* or support* or approach*))

© 2021 Rytter HM et al. *JAMA Network Open.*

S13	(Orthoptic* or Vergence or accommodation or eye movement* or Eye Manifestations or visual pathway* or visual nervous system or visual perception or visual cortex or "V1" or Striate Cortex or Extrastriate Cortex or oculomotor system or oculo-motor system or Vestibo-Ocular reflex* or Vestibuloocular or "Near point of convergence" or Convergence amplitude* or Fusional vergence or fusional reserve* or Vergence facility or Vergence flexibility or Stereopsis or Stereoacuity or "Amplitude of accommodation" or Accommodative amplitude* or "facility of accommodation" or Accommodative facility or Developmental Eye Movement test or "Visual search and Attention Test" or Convergence insufficiency symptom survey or Vergence latency or Vergence peek velocity or Vergence accuracy or Accommodative latency or Accommodative peek velocity or Accommodative accuracy or Eye tracking or Versional peek velocity or Fixation stability or Versional latency or VEP)
S12	((vision or visual or visual-evoked or visual-related or vision-evoked or vision-related or optical or binocular or convergence or vergence or saccadic or oculomotor* or oculo-motor* or accommodative or eye movement* or reading) N6 (therap* or treat* or exercise* or train* or learn* or relearn* or rehabilitat* or neuro-rehabilitation or neurorehabilitation or coordination or attention or potential* or active* or dysfunction* or insufficien* or disorder* or defici* or symptom* or impairment* or injur*))
S11	MH Orthoptics
S10	MH ("Visual cortex" or "Evoked Potentials, Visual" or "Eye Manifestations" or "Reflex, Vestibulo-Ocular")
S9	MH ("Vision perception+" or "Eye Movements+" or "Vision tests")
S8	S1 OR S2 OR S3 OR S4 OR S5 OR S6 OR S7
S7	TBI or PCS
S6	(concussive or post-concussive or postconcussive or post concussive or sport#-concuss* or contusion*)
S5	(concussion* or post-concussion* or postconcussion* or post concussion)
S4	((traumatic or mild or moderat* or minor) N3 (brain or head) N3 (injur* or trauma))
S3	MH "Brain Contusions"
S2	(MH "Brain Injuries")
S1	(MH "Brain Concussion+")

PEDRO

PICOs searched combined

© 2021 Rytter HM et al. *JAMA Network Open.*

Abstract & Title: Concussi* OR post-concussi* OR postconcussi* OR "traumatic brain injury" OR "traumatic head injury"

Method: Systematic review

Published since: 2000

OT Seeker

PICOs searched combined

Any Field ("traumatic brain injur*" OR "traumatic head injur*" OR concussi* OR post-concussi* OR postconcussi* or "post concussi*" or sports-concuss* or contusion*)

PICO 6: Should patients with persistent post-concussive symptoms be offered psychological treatment?

Search date: 27.11.2019

Medline

#	Searches
1	exp brain injuries, traumatic/
2	exp Brain concussion/
3	((traumatic or mild or moderat* or minor) adj3 (brain or head) adj3 (injur* or trauma)).ti,bt,ab,kw,kf.
4	(concussion* or post-concussion* or postconcussion* or post concussion).ti,bt,ab,kw,kf.
5	(concussive or post-concussive or postconcussive or post concussive or sport?-concuss* or contusion*).ti,bt,ab,kw,kf.
6	(TBI or PCS).ti,bt,ab,kw,kf.
7	or/1-6

© 2021 Rytter HM et al. *JAMA Network Open.*

8	exp Psychotherapy/ or exp Mindfulness/
9	((individual or group or Schema* or asserti* or network or communit* or family or carer* or parent* or imagery or marital or spous* or Transference* or cognitive or behavio?r* or mentali#ation* or dynamic or cris#s or emotion* or problem solving or psychodynamic* or Interpersonal or Conversational or psychiatric or Aversive or Biofeedback or Desensiti#ation or Relaxation or Feedback or Gestalt or Hypnosis or Music or Narrative or Psychoanalytic or Psychotherapeutic or Association or Reality or Neurolinguistic Programming or NPL or mindful* or multimodal* or multi-modal*) adj6 (therap* or treatment* or intervention* or management* or model* or program* or modal* or treat* or care or interview* or training or rehabilitation or support* or counseling or counselling or remediation)).ti,bt,ab,kw,kf.
10	(psychotherap* or psycho-therap*).ti,bt,ab,kw,kf.
11	((Psychosocial or psycho-social or psychologic* or neuro-psychologic* or neuropsychologic* or non-pharmacologic* or nonpharmacologic* or non pharmacolog*) adj6 (therap* or treat* or intervention* or program* or plan* or approach* or action* or care or model* or modal* or multimodal* or multi-modal* or interview* or management or training or rehabilitation or support* or counseling or counselling or remediation)).ti,bt,ab,kw,kf.
12	((integrated or integrative or individualized or holistic or relational or tailor* or Multi-modal* or multimodal* or multi-component or multicomponent or multidisciplinary or multi-disciplinary or interdisciplinary or inter-disciplinary or complimentary or multiple) adj6 (therap* or treatment* or intervention* or management* or model* or program* or plan or modal* or multimodal* or multi-modal* or treat* or care or approach* or action* or interview* or training or rehabilitation or support* or counseling or counselling or remediation)).ti,bt,ab,kw,kf.
13	((personalis* or personaliz* or Person-centred or Person-centered or Person-oriented or Person-focused or Person-based or Person-tailored or Patient-centred or Patient-centered or Patient-oriented or Patient-focused or Patient-based or Patient-tailored or Personally-centred or Personally-centered or Personally-oriented or Personally-focused or Personally-based or Personally-tailor* or Client-centred or Client-centered or Client-oriented or Client-focused or Client-based or Client-tailored or individuali#ed or Individual-centred or Individual-centered or Individual-focused or Individual-oriented or Individual-based or Individual-tailored or individually-centred or individually-centered or Individually-focused or Individually-oriented or Individually-based or Individually-tailored or relationship-centred or relationship-centered) adj6 (therap* or treatment* or intervention* or management* or model* or program* or plan or modal* or multimodal* or multi-modal* or treat* or care or approach* or action* or interview* or training or rehabilitation or support* or counseling or counselling or remediation)).ti,bt,ab,kw,kf.
14	((Person* or patient* or client* or individual*) adj2 (centred or center* or orient* or focus* or based or tailor*) adj6 (therap* or treatment* or intervention or management* or model* or program* or plan or modal* or multimodal* or multi-modal* or treat* or care or approach* or action* or interview* or training or rehabilitation or support* or counseling or counselling or remediation)).ti,bt,ab,kw,kf.
15	((tele* or tele-based or tele-support* or telehealth or tele-health or telemedicine or tele-medicine or internet* or internet-based or internetbased or internet-delivered or internet-support* or internet-assisted or web or

© 2021 Rytter HM et al. *JAMA Network Open.*

	webbased or web-based or web based or web-support* or web-assisted or WWW or email* or e-mail or telephone or phone or computer* or computer-based or computerbased or computer-support* or computer-assisted or PC or PCs or PC-based or pc-support* or pc-assisted or skype or skype-based or skype-delivered or skype-assisted or app*1 or app-based or app based or app-assisted or smartphone* or ipad* or tablet* or IOS or android* or online* or on-line or mobile or ehealth or e-health or e-therap* or distance or remote or video-conferenc* or videoconference* or chatroom* or electronic or digital* or technology or technology-based or technology-delivered or technology-supported or technology-assisted) adj3 (counsel* or support* or interview*)).ti,bt,ab,kw,kf.
16	or/8-15
17	7 and 16

© 2021 Rytter HM et al. *JAMA Network Open.*

Embase

#	Searches
1	exp traumatic brain injury/
2	exp Brain concussion/
3	exp brain contusion/
4	exp postconcussion syndrome/
5	((traumatic or mild or moderat* or minor) adj3 (brain or head) adj3 (injur* or trauma)).ti,ab,kw.
6	(concussion* or post-concussion* or postconcussion* or post concussion).ti,ab,kw.
7	(concussive or post-concussive or postconcussive or post concussive or sport?-concuss* or contusion*).ti,ab,kw.
8	(TBI or PCS).ti,ab,kw.
9	or/1-8
10	exp Psychotherapy/ or exp Mindfulness/
11	((individual or group or Schema* or asserti* or network or communit* or family or carer* or parent* or imagery or marital or spous* or Transference* or cognitive or behavio?r* or mentali#ation* or dynamic or cris#s or emotion* or problem solving or psychodynamic* or Interpersonal or Conversational or psychiatric or Aversive or Biofeedback or Desensiti#ation or Relaxation or Feedback or Gestalt or Hypnosis or Music or Narrative or Psychoanalytic or Psychotherapeutic or Association or Reality or Neurolinguistic Programming or NPL or mindful* or multimodal* or multi-modal*) adj6 (therap* or treatment* or intervention* or management* or model* or program* or modal* or treat* or care or interview* or training or rehabilitation or support* or counseling or counselling or remediation)).ti,ab,kw.
12	(psychotherap* or psycho-therap*).ti,ab,kw.
13	exp Psychosocial care/
14	((Psychosocial or psycho-social or psychologic* or neuro-psychologic* or neuropsychologic* or non-pharmacologic* or nonpharmacologic* or non pharmacolog*) adj6 (therap* or treat* or intervention* or program* or plan* or approach* or action* or care or model* or modal* or multimodal* or multi-modal* or interview* or management or training or rehabilitation or support* or counseling or counselling or remediation)).ti,ab,kw.
15	((integrated or integrative or individualized or holistic or relational or tailor* or Multi-modal* or multimodal* or multi-component or multicomponent or multidisciplinary or multi-disciplinary or interdisciplinary or inter-

© 2021 Rytter HM et al. *JAMA Network Open.*

	disciplinary or complimentary or multiple) adj6 (therap* or treatment* or intervention* or management* or model* or program* or plan or modal* or multimodal* or multi-modal* or treat* or care or approach* or action* or interview* or training or rehabilitation or support* or counseling or counselling or remediation)).ti,ab,kw.
16	((personalis* or personaliz* or Person-centred or Person-centered or Person-oriented or Person-focused or Person-based or Person-tailored or Patient-centred or Patient-centered or Patient-oriented or Patient-focused or Patient-based or Patient-tailored or Personally-centred or Personally-centered or Personally-oriented or Personally-focused or Personally-based or Personally-tailor* or Client-centred or Client-centered or Client-oriented or Client-focused or Client-based or Client-tailored or individuali#ed or Individual-centred or Individual-centered or Individual-focused or Individual-oriented or Individual-based or Individual-tailored or individually-centred or individually-centered or Individually-focused or Individually-oriented or Individually-based or Individually-tailored or relationship-centred or relationship-centered) adj6 (therap* or treatment* or intervention* or management* or model* or program* or plan or modal* or multimodal* or multi-modal* or treat* or care or approach* or action* or interview* or training or rehabilitation or support* or counseling or counselling or remediation)).ti,ab,kw.
17	((Person* or patient* or client* or individual*) adj2 (centred or center* or orient* or focus* or based or tailor*) adj6 (therap* or treatment* or intervention or management* or model* or program* or plan or modal* or multimodal* or multi-modal* or treat* or care or approach* or action* or interview* or training or rehabilitation or support* or counseling or counselling or remediation)).ti,ab,kw.
18	((tele* or tele-based or tele-support* or telehealth or tele-health or telemedicine or tele-medicine or internet* or internet-based or internetbased or internet-delivered or internet-support* or internet-assisted or web or webbased or web-based or web based or web-support* or web-assisted or WWW or email* or e-mail or telephone or phone or computer* or computer-based or computerbased or computer-support* or computer-assisted or PC or PCs or PC-based or pc-support* or pc-assisted or skype or skype-based or skype-delivered or skype-assisted or app*1 or app-based or app based or app-assisted or smartphone* or ipad* or tablet* or IOS or android* or online* or on-line or mobile or ehealth or e-health or e-therap* or distance or remote or video-conferenc* or videoconference* or chatroom* or electronic or digital* or technology or technology-based or technology-delivered or technology-supported or technology-assisted) adj3 (counsel* or support* or interview*)).ti,ab,kw.
19	or/10-18
20	9 and 19

PsycINFO

#	Searches
1	exp traumatic brain injury/

© 2021 Rytter HM et al. *JAMA Network Open.*

2	exp Brain concussion/
3	((traumatic or mild or moderat* or minor) adj3 (brain or head) adj3 (injur* or trauma)).ti,ab,id.
4	(concussion* or post-concussion* or postconcussion* or post concussion).ti,ab,id.
5	(concussive or post-concussive or postconcussive or post concussive or sport?-concuss* or contusion*).ti,ab,id.
6	(TBI or PCS).ti,ab,id.
7	or/1-6
8	exp Psychotherapy/
9	exp cognitive therapy/
10	exp recreation therapy/
11	exp counseling/
12	exp mindfulness/
13	mindfulness-based interventions/
14	((individual or group or Schema* or asserti* or recreation* or network or communit* or family or carer* or parent* or imagery or marital or spous* or Transference* or cognitive or behavio?r* or mentali#ation* or dynamic or cris#s or emotion* or problem solving or psychodynamic* or Interpersonal or Conversational or psychiatric or Aversive or Biofeedback or Desensiti#ation or Relaxation or Feedback or Gestalt or Hypnosis or Music or Narrative or Psychoanalytic or Psychotherapeutic or Association or Reality or Neurolinguistic Programming or NPL or mindful* or multimodal* or multi-modal*) adj6 (therap* or treatment* or intervention* or management* or model* or program* or modal* or treat* or care or interview* or training or rehabilitation or support* or counseling or counselling or remediation)).ti,ab,id.
15	(psychotherap* or psycho-therap* or counsel*).ti,ab,id.
16	exp Psychosocial rehabilitation/
17	((Psychosocial or psycho-social or psychologic* or neuro-psychologic* or neuropsychologic* or non-pharmacologic* or nonpharmacologic* or non pharmacolog*) adj6 (therap* or treat* or intervention* or program* or plan* or approach* or action* or care or model* or modal* or multimodal* or multi-modal* or interview* or management or training or rehabilitation or support* or counseling or counselling or remediation)).ti,ab,id.
18	((integrated or integrative or individualized or holistic or relational or tailor* or Multi-modal* or multimodal* or multi-component or multicomponent or multidisciplinary or multi-disciplinary or interdisciplinary or inter-disciplinary or complimentary or multiple) adj6 (therap* or treatment* or intervention* or management* or model* or program* or plan or modal* or multimodal* or multi-modal* or treat* or care or approach* or action*

© 2021 Rytter HM et al. *JAMA Network Open.*

	or interview* or training or rehabilitation or support* or counseling or counselling or remediation)).ti,ab,id.
19	((personalis* or personaliz* or Person-centred or Person-centered or Person-oriented or Person-focused or Person-based or Person-tailored or Patient-centred or Patient-centered or Patient-oriented or Patient-focused or Patient-based or Patient-tailored or Personally-centred or Personally-centered or Personally-oriented or Personally-focused or Personally-based or Personally-tailor* or Client-centred or Client-centered or Client-oriented or Client-focused or Client-based or Client-tailored or individuali#ed or Individual-centred or Individual-centered or Individual-focused or Individual-oriented or Individual-based or Individual-tailored or individually-centred or individually-centered or Individually-focused or Individually-oriented or Individually-based or Individually-tailored or relationship-centred or relationship-centered) adj6 (therap* or treatment* or intervention* or management* or model* or program* or plan or modal* or multimodal* or multi-modal* or treat* or care or approach* or action* or interview* or training or rehabilitation or support* or counseling or counselling or remediation)).ti,ab,id.
20	((Person* or patient* or client* or individual*) adj2 (centred or center* or orient* or focus* or based or tailor*) adj6 (therap* or treatment* or intervention or management* or model* or program* or plan or modal* or multimodal* or multi-modal* or treat* or care or approach* or action* or interview* or training or rehabilitation or support* or counseling or counselling or remediation)).ti,ab,id.
21	((tele* or tele-based or tele-support* or telehealth or tele-health or telemedicine or tele-medicine or internet* or internet-based or internetbased or internet-delivered or internet-support* or internet-assisted or web or webbased or web-based or web based or web-support* or web-assisted or WWW or email* or e-mail or telephone or phone or computer* or computer-based or computerbased or computer-support* or computer-assisted or PC or PCs or PC-based or pc-support* or pc-assisted or skype or skype-based or skype-delivered or skype-assisted or app*1 or app-based or app based or app-assisted or smartphone* or ipad* or tablet* or IOS or android* or online* or on-line or mobile or ehealth or e-health or e-therap* or distance or remote or video-conferenc* or videoconference* or chatroom* or electronic or digital* or technology or technology-based or technology-delivered or technology-supported or technology-assisted) adj3 (counsel* or support* or interview*)).ti,ab,id.
22	or/8-21
23	7 and 22

Cinahl

#	Query
S19	S8 AND S18
S18	S9 OR S10 OR S11 OR S12 OR S13 OR S14 OR S15 OR S16 OR S17

© 2021 Rytter HM et al. *JAMA Network Open.*

S17	((tele* or tele-based or tele-support* or telehealth or tele-health or telemedicine or tele-medicine or internet* or internet-based or internetbased or internet-delivered or internet-support* or internet-assisted or web or webbased or web-based or web based or web-support* or web-assisted or WWW or email* or e-mail or telephone or phone or computer* or computer-based or computerbased or computer-support* or computer-assisted or PC or PCs or PC-based or pc-support* or pc-assisted or skype or skype-based or skype-delivered or skype-assisted or app*1 or app-based or app based or app-assisted or smartphone* or ipad* or tablet* or IOS or android* or online* or on-line or mobile or ehealth or e-health or e-therap* or distance or remote or video-conferenc* or videoconference* or chatroom* or electronic or digital* or technology or technology-based or technology-delivered or technology-supported or technology-assisted) N3 (counsel* or support* or interview*))
S16	((Person* or patient* or client* or individual*) N2 (centred or center* or orient* or focus* or based or tailor*) N6 (therap* or treatment* or intervention or management* or model* or program* or plan or modal* or multimodal* or multi-modal* or treat* or care or approach* or action* or interview* or training or rehabilitation or support* or counseling or counselling or remediation))
S15	((personalis* or personaliz* or Person-centred or Person-centered or Person-oriented or Person-focused or Person-based or Person-tailored or Patient-centred or Patient-centered or Patient-oriented or Patient-focused or Patient-based or Patient-tailored or Personally-centred or Personally-centered or Personally-oriented or Personally-focused or Personally-based or Personally-tailor* or Client-centred or Client-centered or Client-oriented or Client-focused or Client-based or Client-tailored or individuali#ed or Individual-centred or Individual-centered or Individual-focused or Individual-oriented or Individual-based or Individual-tailored or individually-centred or individually-centered or Individually-focused or Individually-oriented or Individually-based or Individually-tailored or relationship-centred or relationship-centered) N6 (therap* or treatment* or intervention* or management* or model* or program* or plan or modal* or multimodal* or multi-modal* or treat* or care or approach* or action* or interview* or training or rehabilitation or support* or counseling or counselling or remediation))
S14	((integrated or integrative or individualized or holistic or relational or tailor* or Multi-modal* or multimodal* or multi-component or multicomponent or multidisciplinary or multi-disciplinary or interdisciplinary or inter-disciplinary or complimentary or multiple) N6 (therap* or treatment* or intervention* or management* or model* or program* or plan or modal* or multimodal* or multi-modal* or treat* or care or approach* or action* or interview* or training or rehabilitation or support* or counseling or counselling or remediation))
S13	((Psychosocial or psycho-social or psychologic* or neuro-psychologic* or neuropsychologic* or non-pharmacologic* or nonpharmacologic* or non pharmacolog*) N6 (therap* or treat* or intervention* or program* or plan* or approach* or action* or care or model* or modal* or multimodal* or multi-modal* or interview* or management or training or rehabilitation or support* or counseling or counselling or remediation))
S12	(psychotherap* or psycho-therap*)

S11	((individual or group* or group-based or Schema* or asserti* or network or communit* or family or carer* or parent* or imagery or marital or spous* or Transference* or cognitive or behavio#r* or mentali#ation* or dynamic or cris#s or emotion* or problem solving or psychodynamic* or Interpersonal or Conversational or psychiatric or Aversive or Biofeedback or Desensiti#ation or Relaxation or Feedback or Gestalt or Hypnosis or Music or Narrative or Psychoanalytic or Psychotherapeutic or Association or Reality or Neurolinguistic Programming or NPL or mindful* or multimodal* or multi-modal*) N6 (therap* or treatment* or intervention* or management* or model* or program* or modal* or treat* or care or interview* or training

	or rehabilitation or support* or counseling or counselling or remediation))
S10	(MH "Support, Psychosocial+") OR (MH "Socioenvironmental Therapy+" or MH "Recreational therapy") or (MH "Rehabilitation, Psychosocial+")
S9	MH Psychotherapy+ or MH Mindfulness+
S8	S1 OR S2 OR S3 OR S4 OR S5 OR S6 OR S7
S7	TBI or PCS
S6	(concussive or post-concussive or postconcussive or post concussive or sport#-concuss* or contusion*)
S5	(concussion* or post-concussion* or postconcussion* or post concussion)
S4	((traumatic or mild or moderat* or minor) N3 (brain or head) N3 (injur* or trauma))
S3	MH "Brain Contusions"
S2	(MH "Brain Injuries")
S1	(MH "Brain Concussion+")

PEDRO

PICOs searched combined

Abstract & Title: Concussi* OR post-concussi* OR postconcussi* OR "traumatic brain injury" OR "traumatic head injury"

Method: Systematic review

Published since: 2000

OT Seeker

PICOs searched combined

Any Field ("traumatic brain injur*" OR "traumatic head injur*" OR concussi* OR post-concussi* OR postconcussi* or "post concussi*" or sports-concuss* or contusion*)

PICO 7: Should patients with persistent post-concussive symptoms be offered an interdisciplinary coordinated rehabilitative treatment?

Search date: 06 - 9.12.2019

Medline

#	Searches
1	exp brain injuries, traumatic/
2	exp Brain concussion/
3	((traumatic or mild or moderat* or minor) adj3 (brain or head) adj3 (injur* or trauma)).ti,bt,ab,kw,kf.
4	(concussion* or post-concussion* or postconcussion* or post concussion).ti,bt,ab,kw,kf.
5	(concussive or post-concussive or postconcussive or post concussive or sport?-concuss* or sport?-related concussion or contusion*).ti,bt,ab,kw,kf.
6	(TBI or PCS).ti,bt,ab,kw,kf.
7	or/1-6
8	Rehabilitation/
9	Activities of Daily Living/
10	Neurological rehabilitation/

© 2021 Rytter HM et al. *JAMA Network Open.*

11	Telerehabilitation/
12	exp Interdisciplinary Studies/
13	exp Interdisciplinary Research/
14	exp Patient-Centered Care/
15	((Multidisciplinary or Multi-disciplinary or Interdisciplinary or Inter-disciplinary or complex or multimodal* or multi-modal* or cross-disciplinary or crossdisciplinary or biopsychosocial or bio-psycho-social or multi-facet* or multifacet* or complimentary or outpatient* or out-patient* or different or multiple or addition*) adj6 (therap* or management* or intervention* or model* or program* or plan or treat* or care or approach* or action* or interview* or train* or retrain* or re-train* or learn* or relearn* or re-learn* or rehabilitation or neurorehabilitation or neuro-rehabilitation or support* or counseling or counselling or remediation)).ti,bt,ab,kw,kf.
16	((vocational rehabilitation or return-to-work or return to work or return-to-activit* or return to activit* or return-to-play or return to play or return-to-sport* or return to sport or return-to-learn* or return to learn* or return-to-academic* or return to academic* or return-to-study or return to study or return-to-studies or return to studies) and (therap* or management* or intervention* or model* or program* or plan or treat* or care or approach* or action* or interview* or train* or retrain* or re-train* or learn* or relearn* or re-learn* or rehabilitation or neurorehabilitation or neuro-rehabilitation or support* or counseling or counselling or remediation)).ti,bt,ab,kw,kf.
17	((integrated or integrative or individualized or holistic or relational or tailor*) adj6 (therap* or treatment* or intervention* or management* or model* or program* or plan or modal* or multimodal* or multi-modal* or treat* or care or approach* or action* or interview* or training or rehabilitation or neurorehabilitation or neuro-rehabilitation or support* or counseling or counselling or remediation)).ti,bt,ab,kw,kf.
18	((personalis* or personaliz* or Person-centred or Person-centered or Person-oriented or Person-focused or Person-based or Person-tailored or Patient-centred or Patient-centered or Patient-oriented or Patient-focused or Patient-based or Patient-tailored or Personally-centred or Personally-centered or Personally-oriented or Personally-focused or Personally-based or Personally-tailor* or Client-centred or Client-centered or Client-oriented or Client-focused or Client-based or Client-tailored or individuali#ed or Individual-centred or Individual-centered or Individual-focused or Individual-oriented or Individual-based or Individual-tailored or individually-centred or individually-centered or Individually-focused or Individually-oriented or Individually-based or Individually-tailored or relationship-centred or relationship-centered) adj6 (therap* or treatment* or intervention* or management* or model* or program* or plan or modal* or multimodal* or multi-modal* or treat* or care or approach* or action* or interview* or training or rehabilitation or neurorehabilitation or neuro-rehabilitation or support* or counseling or counselling or remediation)).ti,bt,ab,kw,kf.
19	((Person* or patient* or client* or individual*) adj2 (centred or center* or orient* or focus* or based or tailor*) adj6 (therap* or treatment* or intervention or management* or model* or program* or plan or modal* or multimodal* or multi-modal* or treat* or care or approach* or action* or interview* or training or rehabilitation

© 2021 Rytter HM et al. *JAMA Network Open.*

	or neurorehabilitation or neuro-rehabilitation or support* or counseling or counselling or remediation)).ti,bt,ab,kw,kf.
20	((non-pharmacological or nonpharmacological or non-drug or nondrug or non-surgical or nonsurgical or non surgical or non-surgery or nonsurgery or non surgery) adj6 (therap* or treat* or intervention* or action* or program* or strateg* or protocol* or support* or approach*)).ti,kw.
21	or/8-20
22	7 and 21

Embase

#	Searches
1	exp traumatic brain injury/
2	exp Brain concussion/
3	exp brain contusion/
4	exp postconcussion syndrome/
5	((traumatic or mild or moderat* or minor) adj3 (brain or head) adj3 (injur* or trauma)).ti,ab,kw.
6	(concussion* or post-concussion* or postconcussion* or post concussion).ti,ab,kw.
7	(concussive or post-concussive or postconcussive or post concussive or sports-concuss* or contusion*).ti,ab,kw.
8	(TBI or PCS).ti,ab,kw.
9	or/1-8
10	Rehabilitation/
11	Activities of Daily Living/
12	Neurorehabilitation/
13	Telerehabilitation/
14	exp Interdisciplinary Research/
15	rehabilitation medicine/
16	multidisciplinary team/
17	community based rehabilitation/

© 2021 Rytter HM et al. *JAMA Network Open.*

18	geriatric rehabilitation/
19	home rehabilitation/
20	daily life activity/
21	rehabilitation care/
22	holistic care/
23	vocational rehabilitation/
24	((Multidisciplinary or Multi-disciplinary or Interdisciplinary or Inter-disciplinary or complex or multimodal* or multi-modal* or cross-disciplinary or crossdisciplinary or biopsychosocial or bio-psycho-social or multi-facet* or multifacet* or complimentary or outpatient* or out-patient* or different or multiple or addition*) adj6 (therap* or management* or intervention* or model* or program* or plan or treat* or care or approach* or action* or interview* or train* or retrain* or re-train* or learn* or relearn* or re-learn* or rehabilitation or neurorehabilitation or neuro-rehabilitation or support* or counseling or counselling or remediation)).ti,ab,kw.
25	((vocational rehabilitation or return-to-work or return to work or return-to-activit* or return to activit* or return-to-play or return to play or return-to-sport* or return to sport or return-to-learn* or return to learn* or return-to-academic* or return to academic* or return-to-study or return to study or return-to-studies or return to studies) and (therap* or management* or intervention* or model* or program* or plan or treat* or care or approach* or action* or interview* or train* or retrain* or re-train* or learn* or relearn* or re-learn* or rehabilitation or neurorehabilitation or neuro-rehabilitation or support* or counseling or counselling or remediation)).ti,ab,kw.
26	((integrated or integrative or holistic or tailor* or personalis* or personaliz* or Person-centred or Person-centered or Person-oriented or Person-focused or Person-based or Person-tailored or Patient-centred or Patient-centered or Patient-oriented or Patient-focused or Patient-based or Patient-tailored or Personally-centred or Personally-centered or Personally-oriented or Personally-focused or Personally-based or Personally-tailor* or Client-centred or Client-centered or Client-oriented or Client-focused or Client-based or Client-tailored or individuali#ed or Individual-centred or Individual-centered or Individual-focused or Individual-oriented or Individual-based or Individual-tailored or individually-centred or individually-centered or Individually-focused or Individually-oriented or Individually-based or Individually-tailored or relationship-centred or relationship-centered) adj6 (therap* or treatment* or intervention* or management* or model* or program* or plan or modal* or multimodal* or multi-modal* or treat* or care or approach* or action* or interview* or training or rehabilitation or neurorehabilitation or neuro-rehabilitation or support* or counseling or counselling or remediation)).ti,ab,kw.
27	((Person* or patient* or client* or individual*) adj2 (centred or center* or orient* or focus* or based or tailor*) adj6 (therap* or treatment* or intervention or management* or model* or program* or plan or modal* or multimodal* or multi-modal* or treat* or care or approach* or action* or interview* or training or rehabilitation or neurorehabilitation or neuro-rehabilitation or support* or counseling or counselling or remediation)).ti,ab,kw.
28	((non-pharmacological or nonpharmacological or non-drug or nondrug or non-surgical or nonsurgical or non surgical or non-surgery or nonsurgery or non surgery) adj6 (therap* or treat* or intervention* or action* or

© 2021 Rytter HM et al. *JAMA Network Open.*

	program* or strateg* or protocol* or support* or approach*)).ti,kw.
29	or/10-28
30	9 and 29

PsycINFO

#	Searches
1	exp traumatic brain injury/
2	exp Brain concussion/
3	((traumatic or mild or moderat* or minor) adj3 (brain or head) adj3 (injur* or trauma)).ti,ab,id.
4	(concussion* or post-concussion* or postconcussion* or post concussion).ti,ab,id.
5	(concussive or post-concussive or postconcussive or post concussive or sport?-concuss* or sport?-related concussion or contusion*).ti,ab,id.
6	(TBI or PCS).ti,ab,id.
7	or/1-6
8	Interdisciplinary Treatment Approach/
9	rehabilitation/
10	rehabilitation counseling/
11	psychosocial rehabilitation/
12	telerehabilitation/
13	Vocational rehabilitation/
14	Activities of Daily Living/
15	Independent living programs/
16	Biopsychosocial Approach/
17	holistic health/
18	((Multidisciplinary or Multi-disciplinary or Interdisciplinary or Inter-disciplinary or complex or multimodal* or multi-modal* or cross-disciplinary or crossdisciplinary or biopsychosocial or bio-psycho-social or multi-facet* or multifacet* or complimentary or outpatient* or out-patient* or different or multiple or addition*) adj6 (therap* or

© 2021 Rytter HM et al. *JAMA Network Open.*

	management* or intervention* or model* or program* or plan or treat* or care or approach* or action* or interview* or train* or retrain* or re-train* or learn* or relearn* or re-learn* or rehabilitation or neurorehabilitation or neuro-rehabilitation or support* or counseling or counselling or remediation)).ti,ab,id.
19	((vocational rehabilitation or return-to-work or return to work or return-to-activit* or return to activit* or return-to-play or return to play or return-to-sport* or return to sport or return-to-learn* or return to learn* or return-to-academic* or return to academic* or return-to-study or return to study or return-to-studies or return to studies) and (therap* or management* or intervention* or model* or program* or plan or treat* or care or approach* or action* or interview* or train* or retrain* or re-train* or learn* or relearn* or re-learn* or rehabilitation or neurorehabilitation or neuro-rehabilitation or support* or counseling or counselling or remediation)).ti,ab,id.
20	exp Psychosocial rehabilitation/
21	((Psychosocial or psycho-social or neuro-psychologic* or neuropsychologic* or non-pharmacologic* or nonpharmacologic* or non pharmacolog* or non-drug or nondrug or non drug or rehabilitative) adj6 (therap* or treat* or intervention* or program* or plan* or approach* or action* or care or model* or modal* or multimodal* or multi-modal* or interview* or management or training or rehabilitation or support* or counseling or counselling or remediation)).ti,ab,id.
22	((integrated or integrative or individualized or holistic or relational or tailor*) adj6 (therap* or treatment* or intervention* or management* or model* or program* or plan or modal* or multimodal* or multi-modal* or treat* or care or approach* or action* or interview* or training or rehabilitation or neurorehabilitation or neuro-rehabilitation or support* or counseling or counselling or remediation)).ti,ab,id.
23	((personalis* or personaliz* or Person-centred or Person-centered or Person-oriented or Person-focused or Person-based or Person-tailored or Patient-centred or Patient-centered or Patient-oriented or Patient-focused or Patient-based or Patient-tailored or Personally-centred or Personally-centered or Personally-oriented or Personally-focused or Personally-based or Personally-tailor* or Client-centred or Client-centered or Client-oriented or Client-focused or Client-based or Client-tailored or individuali#ed or Individual-centred or Individual-centered or Individual-focused or Individual-oriented or Individual-based or Individual-tailored or individually-centred or individually-centered or Individually-focused or Individually-oriented or Individually-based or Individually-tailored or relationship-centred or relationship-centered) adj6 (therap* or treatment* or intervention* or management* or model* or program* or plan or modal* or multimodal* or multi-modal* or treat* or care or approach* or action* or interview* or training or rehabilitation or neurorehabilitation or neuro-rehabilitation or support* or counseling or counselling or remediation)).ti,ab,id.
24	((Person* or patient* or client* or individual*) adj2 (centred or center* or orient* or focus* or based or tailor*) adj6 (therap* or treatment* or intervention or management* or model* or program* or plan or modal* or multimodal* or multi-modal* or treat* or care or approach* or action* or interview* or training or rehabilitation or neurorehabilitation or neuro-rehabilitation or support* or counseling or counselling or remediation)).ti,ab,id.
25	or/8-24
26	7 and 25

© 2021 Rytter HM et al. *JAMA Network Open.*

Cinahl

#	Query
S19	S8 AND S18
S18	S9 OR S10 OR S11 OR S12 OR S13 OR S14 OR S15 OR S16 OR S17
S17	((Person* or patient* or client* or individual*) N2 (centred or center* or orient* or focus* or based or tailor*) N6 (therap* or treatment* or intervention or management* or model* or program* or plan or modal* or multimodal* or multi-modal* or treat* or care or approach* or action* or interview* or training or rehabilitation or neurorehabilitation or neuro-rehabilitation or support* or counseling or counselling or remediation))
S16	((personalis* or personaliz* or Person-centred or Person-centered or Person-oriented or Person-focused or Person-based or Person-tailored or Patient-centred or Patient-centered or Patient-oriented or Patient-focused or Patient-based or Patient-tailored or Personally-centred or Personally-centered or Personally-oriented or Personally-focused or Personally-based or Personally-tailor* or Client-centred or Client-centered or Client-oriented or Client-focused or Client-based or Client-tailored or individuali#ed or Individual-centred or Individual-centered or Individual-focused or Individual-oriented or Individual-based or Individual-tailored or individually-centred or individually-centered or Individually-focused or Individually-oriented or Individually-based or Individually-tailored or relationship-centred or relationship-centered) N6 (therap* or treatment* or intervention* or management* or model* or program* or plan or modal* or multimodal* or multi-modal* or treat* or care or approach* or action* or interview* or training or rehabilitation or neurorehabilitation or neuro-rehabilitation or support* or counseling or counselling or remediation))
S15	((integrated or integrative or individualized or holistic or relational or tailor*) N6 (therap* or treatment* or intervention* or management* or model* or program* or plan or modal* or multimodal* or multi-modal* or treat* or care or approach* or action* or interview* or training or rehabilitation or neurorehabilitation or neuro-rehabilitation or support* or counseling or counselling or remediation))
S14	((Psychosocial or psycho-social or neuro-psychologic* or neuropsychologic* or non-pharmacologic* or nonpharmacologic* or non pharmacolog* or non-drug or nondrug or non drug or rehabilitative) N6 (therap* or treat* or intervention* or program* or plan* or approach* or action* or care or model* or modal* or multimodal* or multi-modal* or interview* or management or training or rehabilitation or support* or counseling or counselling or remediation))

© 2021 Rytter HM et al. *JAMA Network Open.*

S13	((vocational rehabilitation or return-to-work or return to work or return-to-activit* or return to activit* or return-to-play or return to play or return-to-sport* or return to sport or return-to-learn* or return to learn* or return-to-academic* or return to academic* or return-to-study or return to study or return-to-studies or return to studies) and (therap* or management* or intervention* or model* or program* or plan or treat* or care or approach* or action* or interview* or train* or retrain* or re-train* or learn* or relearn* or re-learn* or rehabilitation or neurorehabilitation or

© 2021 Rytter HM et al. *JAMA Network Open.*

	neuro-rehabilitation or support* or counseling or counselling or remediation))
S12	((Multidisciplinary or Multi-disciplinary or Interdisciplinary or Inter-disciplinary or complex or multimodal* or multi-modal* or cross-disciplinary or crossdisciplinary or biopsychosocial or bio-psycho-social or multi-facet* or multifacet* or complimentary or outpatient* or out-patient* or different or multiple or addition*) N6 (therap* or management* or intervention* or model* or program* or plan or treat* or care or approach* or action* or interview* or train* or retrain* or re-train* or learn* or relearn* or re-learn* or rehabilitation or neurorehabilitation or neuro-rehabilitation or support* or counseling or counselling or remediation))
S11	MH ("Rehabilitation, athletic" or "Home rehabilitation" or "Rehabilitation, geriatric" or "Rehabilitation research" or "Rehabilitation, vocational" or "Rehabilitation, community-based" or "Rehabilitation nursing" or "Research, interdisciplinary" or "Telerehabilitation" or "Activities of Daily Living")
S10	(MH "Support, Psychosocial+") or (MH "Rehabilitation, Psychosocial+")
S9	MH ("Rehabilitation" or "Multidisciplinary Care Team" or "Holistic Health")
S8	S1 OR S2 OR S3 OR S4 OR S5 OR S6 OR S7
S7	TBI or PCS
S6	(concussive or post-concussive or postconcussive or post concussive or sport#-concuss* or contusion*)
S5	(concussion* or post-concussion* or postconcussion* or post concussion)
S4	((traumatic or mild or moderat* or minor) N3 (brain or head) N3 (injur* or trauma))
S3	MH "Brain Contusions"
S2	(MH "Brain Injuries")
S1	(MH "Brain Concussion+")

© 2021 Rytter HM et al. *JAMA Network Open.*

PEDRO

PICOs searched combined

Abstract & Title: Concussi* OR post-concussi* OR postconcussi* OR "traumatic brain injury" OR "traumatic head injury"

Method: Systematic review

Published since: 2000

OT Seeker

PICOs searched combined

Any Field ("traumatic brain injur*" OR "traumatic head injur*" OR concussi* OR post-concussi* OR postconcussi* or "post concussi*" or sports-concuss* or contusion*)

Search for randomized controlled trials/controlled trials, observational studies, intervention studies

Filter used to identify randomized/controlled trials and observational/ intervention studies

Medline

1	limit x[1)] to (randomized controlled trial or controlled clinical trial)
2	(((random* or cluster-random* or quasi-random* or control?ed or crossover or cross-over or blind* or mask*) adj4 (trial*1 or study or studies or analy*)) or rct).ti,bt,ab,kw,kf.
3	(placebo* or single-blind* or double-blind* or triple-blind*).ti,bt,kw,kf.

© 2021 Rytter HM et al. *JAMA Network Open.*

4	((single or double or triple) adj2 (blind* or mask*)).ti,bt,kw,kf.
5	((patient* or person* or participant* or population* or allocat* or assign*) adj3 random*).ti,bt,ab,kw,kf.
6	or/2-5
7	x[1)] and 28
8	1 or 7
9	exp epidemiologic studies/
10	exp observational study/
11	exp comparative studies/
12	exp evaluation studies/
13	exp multicenter study/
14	exp twin study/
15	exp validation studies/
16	((Epidemiologic or cohort* or perspective or prospective or longitud* or follow-up or follow up or followup or retro-spective or retrospective or case-control* or case control* or observational or cross-section* or cross section* or multi-cent* or multicent* or evaluation or comparative or intervention or provoca* or validation) adj3 (study or studies or trial*1 or analys*)).ti,bt,kw,kf.
17	or/9-16

1) x means search line for result of subject search

Embase

1	limit x[1)] to (randomized controlled trial or controlled clinical trial)
2	(((random* or cluster-random* or quasi-random* or control?ed or crossover or cross-over or blind* or mask*) adj4 (trial*1 or study or studies or analy*)) or rct).ti,ab,kw.
3	(placebo* or single-blind* or double-blind* or triple-blind*).ti,kw.
4	((single or double or triple) adj2 (blind* or mask*)).ti,kw.
5	((patient* or person* or participant* or population* or allocat* or assign*) adj3 random*).ti,ab,kw.
6	or/2-5

© 2021 Rytter HM et al. *JAMA Network Open.*

7	x[1)] and 6
8	1 or 7
9	Observational study/ or Multicenter study/ or Evaluation study/ or exp comparative study/ or exp case control study/ or intervention study/ or exp longitudinal study/ or prospective study/ or retrospective study/ or Follow up/ or validation study/ or cohort analysis/ or cross-sectional study/ or crossover procedure/ or double blind procedure/ or single blind procedure/
10	((Epidemiologic or cohort* or perspective or prospective or longitud* or follow-up or follow up or followup or retro-spective or retrospective or case-control* or case control* or observational or cross-section* or cross section* or multi-cent* or multicent* or evaluation or comparative or intervention or provoca* or validation) adj1 (study or studies or trial*1 or analys*)).ti,kw.
11	or/9-10
12	x[1)] and 11

1) x means search line for result of subject search

PsycINFO

1	(((random* or cluster-random* or quasi-random* or control?ed or crossover or cross-over) adj4 (trial*1 or study or studies or analy*)) or rct).ti,ab,id.
2	((patient* or person* or participant* or population* or allocat* or assign*) adj3 random*).ti,ab,id.
3	exp Longitudinal Studies/ or exp Followup Studies/ or exp Retrospective Studies/ or exp Prospective Studies/
4	((Epidemiologic or cohort* or perspective or prospective or longitud* or follow-up or follow up or followup or retro-spective or retrospective or case-control* or case control* or observational or cross-section* or cross section* or multi-cent* or multicent* or evaluation or comparative or intervention or provoca* or validation) adj1 (study or studies or trial*1 or analys*)).ti,id.
5	or/1-4

© 2021 Rytter HM et al. *JAMA Network Open.*

Cinahl

S6	S1 OR S2 OR S3 OR S4 OR S5
S5	TI ((Epidemiologic or cohort* or perspective or prospective or longitud* or follow-up or follow up or followup or retro-spective or retrospective or case-control* or case control* or observational or cross-section* or cross section* or multi-cent* or multicent* or evaluation or comparative or intervention or provoca* or validation) N3 (study or studies or trial* or analys*)) OR SU ((Epidemiologic or cohort* or perspective or prospective or longitud* or follow-up or follow up or followup or retro-spective or retrospective or case-control* or case control* or observational or cross-section* or cross section* or multi-cent* or multicent* or evaluation or comparative or intervention or provoca* or validation) N3 (study or studies or trial* or analys*))
S4	((patient* or person* or participant* or population* or allocat* or assign*) N3 (random* or blind* or mask*))
S3	TI (placebo* or single-blind* or double-blind* or triple-blind* or ((single or double or triple) N1 (blind* or mask*)) OR SU (placebo* or single-blind* or double-blind* or triple-blind* or ((single or double or triple) N1 (blind* or mask*))
S2	TI (((random* or cluster-random* or quasi-random* or control#ed or crossover or cross-over or blind* or mask*) N4 (trial* or study or studies or analy*)) or rct) OR SU (((random* or cluster-random* or quasi-random* or control#ed or crossover or cross-over or blind* or mask*) N4 (trial* or study or studies or analy*)) or rct)
S1	PT Randomized Controlled Trial OR MH "Randomized Controlled Trial+" OR MH "Controlled Clinical Trial+" OR MH "Nonexperimental Studies+"

PICO 1: Should patients, early after concussion, be offered systematically applied information and advice on preventing persistent post-concussive symptoms?

PICO 6: Should patients with persistent post-concussive symptoms be offered psychological treatment?

PICO 1 and PICO 6 searched combined.

Search date: 17-18.02.2020

After search and sorting of duplicates, additional references are removed containing:

© 2021 Rytter HM et al. *JAMA Network Open.*

TITLE: child* (but not ”child* and adolescen*), pediatric*, paediatric*, case study, protocol, conference, congress, surg*, severe traumatic brain injury, severe TBI, rat*, cancer

Medline

#	Searches
1	brain injuries, traumatic/
2	exp Brain concussion/
3	((traumatic or mild or moderat* or minor) adj3 (brain or head) adj3 (injur* or trauma)).ti,bt,kw,kf.
4	(concussion* or post-concussion* or postconcussion* or post concussion).ti,bt,kw,kf.
5	(concussive or post-concussive or postconcussive or post concussive or sport?-concuss* or contusion*).ti,bt,kw,kf.
6	(TBI or mTBI).ti,bt,kw,kf.
7	or/1-6
8	Health education/
9	exp Patient Education as Topic/
10	(psychoeducat* or psycho-educat*).ti,bt,ab,kw,kf.
11	((information or educat* or guid* or counsel* or councel* or Course* or Coach* or guide or guidance or advic* or advis* or handout* or hand-out* or pamphlet* or leaflet* or booklet* or brochure*) adj6 (health or concussi* or post-concuss* or postconcussi* or treatment* or therapy or intervention or management or patient* or tailor* or individual* or Patient-centred or Patient-centered or Patient-oriented or Patient-focused or Patient-based or Patient-tailored or client-cent* or client cent* or client-focus* or client focus* or Client-oriented or Client-based or Client-tailored or person-centered or person-centred or person centered or person centred or person-focus* or person focus* or Person-oriented or Person-based or Person-tailored or Individual-centred or Individual-centered or Individual-focused or Individual-oriented or Individual-based or Individual-tailored)).ti,bt,ab,kw,kf.
12	(self-management or self-instruction* or selfmanagement or selfinstruction* or self management or self instruction* or self-guid* or self guid* or selfguid* or guidance).ti,bt,ab,kw,kf.
13	((information* or instruction* or educat*) adj3 (material or advic* or advis* or handout* or hand-out* or pamphlet* or leaflet* or booklet* or brochure* or guid*)).ti,bt,ab,kw,kf.
14	exp Psychotherapy/ or exp Mindfulness/
15	exp Cognitive Behavioral Therapy/

16	(cbt or act or (acceptance adj2 therapy)).ti,bt,ab,kw,kf.
17	(cognitive-behavio?r-therapy or cognitive-behavio?ral-therapy).ti,bt,ab,kw,kf.
18	((cognitive or cognitive-behavio?r* or individual or group or Schema* or asserti* or network or communit* or family or carer* or parent* or imagery or marital or spous* or Transference* or cognitive or behavio?r* or mentali#ation* or dynamic or cris#s or emotion* or problem solving or problem-solving or psychodynamic* or Interpersonal or Conversational or psychiatric or Aversive or Biofeedback or Desensiti#ation or Relaxation or Feedback or Gestalt or Hypnosis or Music or Narrative or Psychoanalytic or Psychotherapeutic or Association or Reality or Neurolinguistic Programming or NPL or mindful* or meditation) adj3 (therap* or treatment* or intervention* or management* or model* or program* or modal* or treat* or interview* or training or rehabilitation or neuro-rehabilitation or neurorehabilitation or support* or counseling or counselling or remediation or session*)).ti,bt,ab,kw,kf.
19	(psychotherap* or psycho-therap*).ti,bt,ab,kw,kf.
20	((Psychosocial or psycho-social or psychologic* or neuro-psycholog* or neuropsycholog*) adj3 (therap* or treat* or intervention* or program* or plan* or approach* or action* or model* or modal* or multimodal* or multi-modal* or interview* or management or training or rehabilitation or neuro-rehabilitation or neurorehabilitation or support* or counseling or counselling or remediation or session*)).ti,bt,ab,kw,kf.
21	or/8-20
22	7 and 21

Embase

#	Searches
1	traumatic brain injury/
2	exp brain concussion/
3	exp brain contusion/
4	exp postconcussion syndrome/
5	((traumatic or mild or moderat* or minor) adj3 (brain or head) adj3 (injur* or trauma)).ti,kw.
6	(concussion* or post-concussion* or postconcussion* or post concussion*).ti,kw.
7	(concussive or post-concussive or postconcussive or post concussive or sport?-concuss* or contusion*).ti,kw.
8	(TBI or mTBI).ti,kw.
9	or/1-8
10	Health education/

© 2021 Rytter HM et al. *JAMA Network Open.*

11	exp Patient Education/
12	exp Psychoeducation/
13	(psychoeducat* or psycho-educat*).ti,ab,kw.
14	((information or educat* or guid* or counsel* or councel* or Course* or Coach* or guide or guidance or advic* or advis* or handout* or hand-out* or pamphlet* or leaflet* or booklet* or brochure*) adj3 (health or concussi* or post-concuss* or postconcussi* or treatment* or therapy or intervention or management or patient* or tailor* or individual* or Patient-centred or Patient-centered or Patient-oriented or Patient-focused or Patient-based or Patient-tailored or client-cent* or client cent* or client-focus* or client focus* or Client-oriented or Client-based or Client-tailored or person-centered or person-centred or person centered or person centred or person-focus* or person focus* or Person-oriented or Person-based or Person-tailored or Individual-centred or Individual-centered or Individual-focused or Individual-oriented or Individual-based or Individual-tailored)).ti,ab,kw.
15	(self-management or self-instruction* or selfmanagement or selfinstruction* or self management or self instruction* or self-guid* or self guid* or selfguid* or guidance).ti,ab,kw.
16	((information* or instruction* or educat*) adj3 (material or advic* or advis* or handout* or hand-out* or pamphlet* or leaflet* or booklet* or brochure* or guid*)).ti,ab,kw.
17	exp Psychotherapy/ or exp Mindfulness/
18	Psychosocial rehabilitation/
19	exp Cognitive Behavior Therapy/
20	(cbt or act or (acceptance adj2 therapy)).ti,ab,kw.
21	(cognitive-behavio?r-therapy or cognitive-behavio?ral-therapy).ti,ab,kw.
22	((cognitive or cognitive-behavio?r* or individual or group or Schema* or asserti* or network or communit* or family or carer* or parent* or imagery or marital or spous* or Transference* or cognitive or behavio?r* or mentali#ation* or dynamic or cris#s or emotion* or problem solving or problem-solving or psychodynamic* or Interpersonal or Conversational or psychiatric or Aversive or Biofeedback or Desensiti#ation or Relaxation or Feedback or Gestalt or Hypnosis or Music or Narrative or Psychoanalytic or Psychotherapeutic or Association or Reality or Neurolinguistic Programming or NPL or mindful* or meditation) adj3 (therap* or treatment* or intervention* or management* or model* or program* or modal* or treat* or interview* or training or rehabilitation or neuro-rehabilitation or neurorehabilitation or support* or counseling or counselling or remediation or session*)).ti,ab,kw.
23	(psychotherap* or psycho-therap*).ti,ab,kw.
24	((Psychosocial or psycho-social or psychologic* or neuro-psycholog* or neuropsycholog*) adj3 (therap* or treat* or intervention* or program* or plan* or approach* or action* or model* or modal* or multimodal* or multi-modal* or interview* or management or training or rehabilitation or neuro-rehabilitation or neurorehabilitation or support* or counseling or counselling or remediation or session*)).ti,ab,kw.
25	or/10-24
26	9 and 25

PsycINFO

© 2021 Rytter HM et al. *JAMA Network Open.*

#	Searches
1	traumatic brain injury/
2	exp Brain concussion/
3	((traumatic or mild or moderat* or minor) adj3 (brain or head) adj3 (injur* or trauma)).ti,ab,id.
4	(concussion* or post-concussion* or postconcussion* or post concussion).ti,ab,id.
5	(concussive or post-concussive or postconcussive or post concussive or sport?-concuss* or contusion*).ti,ab,id.
6	(TBI or mTBI).ti,ab,id.
7	or/1-6
8	Health education/
9	exp Client Education/
10	exp Psychoeducation/
11	(psychoeducat* or psycho-educat*).ti,ab,id.
12	((information or educat* or guid* or counsel* or councel* or Course* or Coach* or guide or guidance or advic* or advis* or handout* or hand-out* or pamphlet* or leaflet* or booklet* or brochure*) adj6 (health or concussi* or post-concuss* or postconcussi* or treatment* or therapy or intervention or management or patient* or tailor* or individual* or Patient-centred or Patient-centered or Patient-oriented or Patient-focused or Patient-based or Patient-tailored or client-cent* or client cent* or client-focus* or client focus* or Client-oriented or Client-based or Client-tailored or person-centered or person-centred or person centered or person centred or person-focus* or person focus* or Person-oriented or Person-based or Person-tailored or Individual-centred or Individual-centered or Individual-focused or Individual-oriented or Individual-based or Individual-tailored)).ti,ab,id.
13	(self-management or self-instruction* or selfmanagement or selfinstruction* or self management or self instruction* or self-guid* or self guid* or selfguid* or guidance).ti,ab,id.
14	((information* or instruction* or educat*) adj3 (material or advic* or advis* or handout* or hand-out* or pamphlet* or leaflet* or booklet* or brochure* or guid*)).ti,ab,id.
15	exp Psychotherapy/
16	exp cognitive therapy/
17	exp recreation therapy/
18	Neuropsychology/ and (therap* or treatment* or intervention* or management* or model* or program* or modal* or treat* or interview* or training or rehabilitation or support* or counseling or counselling or remediation or session*).ti,ab,id.
19	exp counseling/
20	psychosocial rehabilitation/
21	exp mindfulness/

© 2021 Rytter HM et al. *JAMA Network Open.*

22	mindfulness-based interventions/
23	Biopsychosocial Approach/
24	(cbt or act or (acceptance adj2 therapy)).ti,ab,id.
25	(cognitive-behavio?r-therapy or cognitive-behavio?ral-therapy).ti,ab,id.
26	((cognitive or cognitive-behavio?r* or individual or group or Schema* or asserti* or network or communit* or family or carer* or parent* or imagery or marital or spous* or Transference* or cognitive or behavio?r* or mentali#ation* or dynamic or cris#s or emotion* or problem solving or problem-solving or psychodynamic* or Interpersonal or Conversational or psychiatric or Aversive or Biofeedback or Desensiti#ation or Relaxation or Feedback or Gestalt or Hypnosis or Music or Narrative or Psychoanalytic or Psychotherapeutic or Association or Reality or Neurolinguistic Programming or NPL or mindful* or meditation) adj3 (therap* or treatment* or intervention* or management* or model* or program* or modal* or treat* or interview* or training or rehabilitation or neuro-rehabilitation or neurorehabilitation or support* or counseling or counselling or remediation or session*)).ti,ab,id.
27	(psychotherap* or psycho-therap*).ti,ab,id.
28	((Psychosocial or psycho-social or psychologic* or neuro-psycholog* or neuropsycholog*) adj3 (therap* or treat* or intervention* or program* or plan* or approach* or action* or model* or modal* or multimodal* or multi-modal* or interview* or management or training or rehabilitation or neuro-rehabilitation or neurorehabilitation or support* or counseling or counselling or remediation or session*)).ti,ab,id.
29	or/8-28
30	7 and 29

Cochrane Central

ID	Search Hits
#1	MeSH descriptor: [Brain Injuries, Traumatic] this term only
#2	MeSH descriptor: [Brain Concussion] explode all trees
#3	MeSH descriptor: [Post-Concussion Syndrome] explode all trees
#4	((traumatic or mild or moderat* or minor) NEAR/3 (brain or head) NEAR/3 (injur* or trauma)):ti,kw
#5	(concussion* or post-concussion* or postconcussion* or post concussion*):ti,kw
#6	(concussive or post-concussive or postconcussive or "post concussive" or sport-concuss* or contusion*):ti,kw
#7	(TBI or mTBI):ti,kw
#8	#1 or #2 or #3 or #4 or #5 or #6 or #7
#9	MeSH descriptor: [Health Education] explode all trees
#10	MeSH descriptor: [Patient Education as Topic] explode all trees

© 2021 Rytter HM et al. *JAMA Network Open.*

#11 (psychoeducat* or psycho-educat*):ti,ab,kw

#12 ((information or educat* or guid* or counsel* or councel* or Course* or Coach* or guide or guidance or advic* or advis* or handout* or hand-out* or pamphlet* or leaflet* or booklet* or brochure*) NEAR/6 (health or concussi* or post-concuss* or postconcussi* or treatment* or therapy or intervention or management or patient* or tailor* or individual* or Patient-centred or Patient-centered or Patient-oriented or Patient-focused or Patient-based or Patient-tailored or client-cent* or client cent* or client-focus* or client focus* or Client-oriented or Client-based or Client-tailored or person-centered or person-centred or person centered or person centred or person-focus* or person focus* or Person-oriented or Person-based or Person-tailored or Individual-centred or Individual-centered or Individual-focused or Individual-oriented or Individual-based or Individual-tailored)):ti,ab,kw

#13 (self-management or self-instruction* or selfmanagement or selfinstruction* or self management or self instruction* or self-guid* or self guid* or selfguid* or guidance):ti,ab,kw

#14 ((information* or instruction* or educat*) NEAR/3 (material or advic* or advis* or handout* or hand-out* or pamphlet* or leaflet* or booklet* or brochure* or guid*)):ti,ab,kw

#15 MeSH descriptor: [Psychotherapy] explode all trees

#16 MeSH descriptor: [Mindfulness] explode all trees

#17 MeSH descriptor: [Cognitive Behavioral Therapy] explode all trees

#18 (cbt or act or (acceptance NEAR/2 therapy)):ti,ab,kw

#19 (cognitive-behavio?r-therapy or cognitive-behavio?ral-therapy):ti,ab,kw

#20 ((cognitive or cognitive-behavio*r* or cognitive-behavio*r* individual or Schema* or asserti* or network or communit* or family or imagery or marital or spous* or Transference* or cognitive or behavio*r* or mentali?ation* or dynamic or cris?s or emotion* or problem solving or problem-solving or psychodynamic* or Interpersonal or Conversational or psychiatric or Aversive or Biofeedback or Desensiti?ation or Relaxation or Feedback or Gestalt or Hypnosis or Music or Narrative or Psychoanalytic or Psychotherapeutic or Association or Reality or Neurolinguistic Programming or NPL or mindful* or meditation) NEAR/3 (therap* or treatment* or intervention* or management* or model* or program* or treat* or training or rehabilitation or neuro-rehabilitation or neurorehabilitation or counseling or counselling or remediation or session*)):ti,ab,kw

#21 (psychotherap* or psycho-therap*):ti,ab,kw

#22 ((Psychosocial or psycho-social or psychologic* or neuro-psycholog* or neuropsycholog*) NEAR/3 (therap* or treatment* or intervention* or management* or model* or program* or treat* or training or rehabilitation or neuro-rehabilitation or neurorehabilitation or support* or counseling or counselling or remediation or session*)):ti,ab,kw

#23 #9 or #10 or #11 or #12 or #13 or #14 or #15 or #16 or #17 or #18 or #19 or #20 or #21 or #22

#24 #8 AND #23 in Trials

Cinahl

© 2021 Rytter HM et al. *JAMA Network Open.*

#	Query
S23	S8 AND S22
S22	S9 OR S10 OR S11 OR S12 OR S13 OR S14 OR S15 OR S16 OR S17 OR S18 OR S19 OR S20 OR S21
S21	TI ((Psychosocial or psycho-social or psychologic* or neuro-psycholog* or neuropsycholog*) N3 (therap* or treat* or intervention* or program* or plan* or approach* or action* or model* or management or training or rehabilitation or neuro-rehabilitation or neurorehabilitation or support* or counseling or counselling or remediation or session*)) OR SU ((Psychosocial or psycho-social or psychologic* or neuro-psycholog* or neuropsycholog*) N3 (therap* or treat* or intervention* or program* or plan* or approach* or action* or model* or management or training or rehabilitation or neuro-rehabilitation or neurorehabilitation or support* or counseling or counselling or remediation or session*))
S20	TI (psychotherap* or psycho-therap*) OR SU (psychotherap* or psycho-therap*)
S19	TI ((cognitive or cognitive-behavio#r* or individual or Schema* or asserti* or network or communit* or family or imagery or marital or spous* or Transference* or cognitive or behavio#r* or mentali?ation* or dynamic or cris?s or emotion* or problem solving or problem-solving or psychodynamic* or Interpersonal or Conversational or psychiatric or Aversive or Biofeedback or Desensiti?ation or Relaxation or Feedback or Gestalt or Hypnosis or Music or Narrative or Psychoanalytic or Psychotherapeutic or Association or Reality or Neurolinguistic Programming or NPL or mindful* or meditation) N3 (therap* or treatment* or intervention* or management* or model* or program* or treat* or training or rehabilitation or neuro-rehabilitation or neurorehabilitation or support* or counseling or counselling or remediation or session*)) OR SU ((cognitive or cognitive-behavio#r* or individual or Schema* or asserti* or network or communit* or family or imagery or marital or spous* or Transference* or cognitive or behavio#r* or mentali?ation* or dynamic or cris?s or emotion* or problem solving or problem-solving or psychodynamic* or Interpersonal or Conversational or psychiatric or Aversive or Biofeedback or Desensiti?ation or Relaxation or Feedback or Gestalt or Hypnosis or Music or Narrative or Psychoanalytic or Psychotherapeutic or Association or Reality or Neurolinguistic Programming or NPL or mindful* or meditation) N3 (therap* or treatment* or intervention* or management* or model* or program* or treat* or training or rehabilitation or neuro-rehabilitation or neurorehabilitation or support* or counseling or counselling or remediation or session*))
S18	TI (cognitive-behavio#r-therapy or cognitive-behavio#ral-therapy) OR SU (cognitive-behavio#r-therapy or cognitive-behavio#ral-therapy)
S17	TI (cbt or act or (acceptance N2 therapy)) OR SU (cbt or act or (acceptance N2 therapy))
S16	MH Neuropsychology and (therap* or treatment* or intervention* or management* or model* or program* or treat* or training or rehabilitation or support* or counseling or counselling or remediation or session*)
S15	(MH "Support, Psychosocial+") OR (MH "Socioenvironmental Therapy+" or MH "Recreational therapy") or (MH "Rehabilitation, Psychosocial+")

© 2021 Rytter HM et al. *JAMA Network Open.*

S14	MH (Psychotherapy+ or Mindfulness+ or Counseling+ or Relaxation Techniques)
S13	TI ((information* or instruction* or educat*) N3 (material or advic* or advis* or handout* or hand-out* or pamphlet* or leaflet* or booklet* or brochure* or guid*)) OR SU ((information* or instruction* or educat*) N3 (material or advic* or advis* or handout* or hand-out* or pamphlet* or leaflet* or booklet* or brochure* or guid*))
S12	TI (self-management or self-instruction* or selfmanagement or selfinstruction* or self management or self instruction* or self-guid* or self guid* or selfguid* or guidance) OR SU (self-management or self-instruction* or selfmanagement or selfinstruction* or self management or self instruction* or self-guid* or self guid* or selfguid* or guidance)
S11	TI ((information or educat* or guid* or counsel* or councel* or Course* or Coach* or session* or consult* or guid* or advic* or advis* or handout* or hand-out* or pamphlet* or leaflet* or booklet* or brochure*) N10 (treatment* or therapy or intervention or management or patient* or tailor* or individual* or Patient-centred or Patient-centered or Patient-oriented or Patient-focused or Patient-based or Patient-tailored or client-cent* or client cent* or client-focus* or client focus* or Client-oriented or Client-based or Client-tailored or person-cent* or person cent* or person-focus* or person focus* or Person-oriented or Person-based or Person-tailored or Individual-centred or Individual-centered or Individual-focused or Individual-oriented or Individual-based or Individual-tailored)) OR SU ((information or educat* or guid* or counsel* or councel* or Course* or Coach* or session* or consult* or guid* or advic* or advis* or handout* or hand-out* or pamphlet* or leaflet* or booklet* or brochure*) N10 (treatment* or therapy or intervention or management or patient* or tailor* or individual* or Patient-centred or Patient-centered or Patient-oriented or Patient-focused or Patient-based or Patient-tailored or client-cent* or client cent* or client-focus* or client focus* or Client-oriented or Client-based or Client-tailored or person-cent* or person cent* or person-focus* or person focus* or Person-oriented or Person-based or Person-tailored or Individual-centred or Individual-centered or Individual-focused or Individual-oriented or Individual-based or Individual-tailored))
S10	TI (psychoeducat* or psycho-educat*) OR SU (psychoeducat* or psycho-educat*)
S9	MH ("Health education" or "Patient Education" or "Patient Discharge Education" or "Psychoeducation")
S8	S1 OR S2 OR S3 OR S4 OR S5 OR S6 OR S7
S7	TI (TBI or mTBI) OR SU (TBI or mTBI)
S6	TI (concussive or post-concussive or postconcussive or post concussive or sport#-concuss* or contusion*) OR SU (concussive or post-concussive or postconcussive or post concussive or sport#-concuss* or contusion*)
S5	TI (concussion* or post-concussion* or postconcussion* or post concussion) OR SU (concussion* or post-concussion* or postconcussion* or post concussion)
S4	TI ((traumatic or mild or moderat* or minor) N3 (brain or head) N3 (injur* or trauma)) OR SU ((traumatic or mild or moderat* or minor) N3 (brain or head) N3 (injur* or trauma))

© 2021 Rytter HM et al. *JAMA Network Open.*

S3	MH "Brain Contusions"
S2	(MH "Brain Injuries")
S1	(MH "Brain Concussion+")

PEDRO

PICOs searched combined

Abstract & Title: Concussi* OR post-concussi* OR postconcussi* OR "traumatic brain injury" OR "traumatic head injury"

Method: Clinical trial

Published since: All years

OT Seeker

PICOs searched combined

Any Field: ("traumatic brain injur*" OR "traumatic head injur*" OR concussi* OR post-concussi* OR postconcussi* or "post concussi*" or sports-concuss* or contusion*)

Method: Randomized Controlled Trial

PICO 2: Should patients with persistent post-concussive symptoms be offered graded physical exercise?

Search date: 09.03.2020

After search and sorting of duplicates, additional references are removed containing:

TITLE: child* (but not ”child* and adolescen*), pediatric*, case study, protocol, conference, congress, severe traumatic brain injury

---

Medline

© 2021 Rytter HM et al. *JAMA Network Open.*

#	Searches
1	exp brain injuries, traumatic/
2	exp Brain concussion/
3	((traumatic or mild or moderat* or minor) adj3 (brain or head) adj3 (injur* or trauma)).ti,bt,ab,kw,kf.
4	(concussion* or post-concussion* or postconcussion* or post concussion).ti,bt,ab,kw,kf.
5	(concussive or post-concussive or postconcussive or post concussive or sports-concuss* or contusion*).ti,bt,ab,kw,kf.
6	(TBI or mTBI).ti,bt,ab,kw,kf.
7	or/1-6
8	exp exercise/
9	exp exercise therapy/
10	exp Sports/
11	exp Physical Exertion/
12	exp Muscle strength/
13	exp Exercise Movement Techniques/
14	exp "Physical Education and Training"/
15	exp Hydrotherapy/
16	Weight-Bearing/
17	Tai Ji/
18	Yoga/
19	physical therapy modalities/
20	occupational therapy/
21	(exercis* or train* or stepped care or physical therap* or physiotherap* or kinesiotherapy or occupational therapy).ti,bt,kw,kf.
22	((muscl* or resistance or physical or endurance or strength* or balance or postur* or gait or threshold* or treadmill* or aquatic* or trunk or graded or gradual or progressive or controlled or incremented or symptom-based or symptombased or symptom based or guided or supervis* or stepped care or stepwise or step-wise) adj3 (train* or retrain* or re-train* or exercise* or rehabilitation or neurohabilitation or strength* or fit* or condition*

© 2021 Rytter HM et al. *JAMA Network Open.*

	or exposure or activit* or exertion or load* or physical therap* or physiotherap* or occupational therapy)).ti,bt,ab,kw,kf.
23	((graded or gradual or progressive) adj6 (return or return-to-work or return-to sport* or return-to-study or return-to-studies)).ti,bt,ab,kw,kf.
24	(active rehabilitation or aerobic* or anaerobic* or movement therapy or watergym* or water gym* or aquagym or gym* or hydrotherap* or (warm water adj4 (exercis* or train* or rehab)) or pilates or spinning).ti,bt,ab,kw,kf.
25	(running or walking or Jog* or stepping or zumba or kettlebelt or Swim* or Bicycl* or cycling or bicycling or spinning or rowing or pacing or paced or titreted or (weight* adj3 (lift* or train* or exercis*))).ti,bt,ab,kw,kf.
26	(yoga or tai ji or tai chi or qigong or qi-gong).ti,bt,ab,kw,kf.
27	((mckenzie or mechanical or Alexander or William or Feldenkrais) adj2 (technique* or model* or tool* or exercise* or therapy)).ti,bt,ab,kw,kf.
28	or/8-27
29	7 and 28
30	limit 29 to (randomized controlled trial or controlled clinical trial)
31	(((random* or cluster-random* or quasi-random* or control?ed or crossover or cross-over or blind* or mask*) adj4 (trial*1 or study or studies or analy*)) or rct).ti,bt,ab,kw,kf.
32	(placebo* or single-blind* or double-blind* or triple-blind*).ti,bt,ab,kw,kf.
33	((single or double or triple) adj2 (blind* or mask*)).ti,bt,ab,kw,kf.
34	((patient* or person* or participant* or population* or allocate* or assign*) adj3 (random* or blind* or mask*)).ti,bt,ab,kw,kf.
35	or/31-34
36	29 and 35
37	30 or 36
38	limit 37 to (yr="2016-2020" and (danish or english or norwegian or swedish))
39	exp Child/ not ((exp adult/ or exp Adolescent/) and exp Child/)
40	38 not 39
41	exp epidemiologic studies/
42	exp observational study/
43	exp comparative studies/

© 2021 Rytter HM et al. *JAMA Network Open.*

44	exp evaluation studies/
45	exp multicenter study/
46	exp twin study/
47	exp validation studies/
48	((Epidemiologic or cohort* or perspective or prospective or longitud* or follow-up or follow up or followup or retro-spective or retrospective or case-control* or case control* or observational or cross-section* or cross section* or multi-cent* or multicent* or evaluation or comparative or intervention or provoca* or validation) adj3 (study or studies or trial*1 or analys*)).ti,bt,ab,kw,kf.
49	or/41-48
50	29 and 49
51	limit 50 to (yr="2016-2020" and (english or danish or norwegian or swedish))
52	exp Child/ not ((exp adult/ or exp Adolescent/) and exp Child/)
53	51 not 52
54	40 or 53
55	limit 54 to (review or systematic reviews or news or editorial)
56	54 not 55
57	56 not (case study or case studies or conference abstract or protocol*).ti,bt,kw,kf.

Embase

#	Searches
1	exp traumatic brain injury/
2	exp Brain concussion/
3	exp brain contusion/
4	exp postconcussion syndrome/
5	((traumatic or mild or moderat* or minor) adj3 (brain or head) adj3 (injur* or trauma)).ti,ab,kw.
6	(concussion* or post-concussion* or postconcussion* or post concussion).ti,ab,kw.

© 2021 Rytter HM et al. *JAMA Network Open.*

7	(concussive or post-concussive or postconcussive or post concussive or sports-concuss* or contusion*).ti,ab,kw.
8	(TBI or mTBI).ti,ab,kw.
9	or/1-8
10	exp exercise/
11	exp kinesiotherapy/
12	exp Sports/
13	exp Physical Exertion/
14	exp Muscle strength/
15	exp Exercise Movement Techniques/
16	exp "Physical Education and Training"/
17	exp Hydrotherapy/
18	Weight-Bearing/
19	Tai Ji/
20	Yoga/
21	physical therapy modalities/
22	occupational therapy/
23	(exercis* or train* or stepped care or physical therap* or physiotherap* or kinesiotherapy or occupational therapy).ti,kw.
24	((muscl* or resistance or physical or endurance or strength* or balance or postur* or gait or aquatic* or trunk or graded or gradual or progressive or controlled or incremented or symptom-based or symptombased or symptom based or guided or supervis* or stepped care or stepwise or step-wise) adj3 (train* or retrain* or re-train* or exercise* or rehabilitation or neurorehabilitation or neuro-rehabilitation or strength* or fit* or condition* or exposure or activit* or exertion or load* or physical therap* or physiotherap* or occupational therapy)).ti,ab,kw.
25	((graded or gradual or progressive) adj6 (return or return-to-work or return-to sport* or return-to-study or return-to-studies)).ti,ab,kw.
26	(aerobic* or anaerobic* or movement therapy or watergym* or water gym* or aquagym or gym* or hydrotherap* or (warm water adj4 (exercis* or train* or rehab))).ti,ab,kw.
27	(running or walking or Jog* or stepping or zumba or kettlebelt or Swim* or Bicycl* or cycling or bicycling or spinning or rowing or pacing or paced or titreted or (weight* adj3 (lift* or train* or exercis*))).ti,ab,kw.

28	(yoga or tai ji or tai chi or qigong or qi-gong or pilates).ti,ab,kw.
29	((mckenzie or mechanical or Alexander or William or Feldenkrais) adj2 (technique* or model* or tool* or exercise* or therapy)).ti,ab,kw.
30	or/10-29
31	9 and 30

Cochrane Central

ID Search Hits

#1 MeSH descriptor: [Brain Injuries, Traumatic] this term only 441

#2 MeSH descriptor: [Brain Concussion] explode all trees 327

#3 MeSH descriptor: [Post-Concussion Syndrome] explode all trees 127

#4 ((traumatic or mild or moderat* or minor) NEAR/3 (brain or head) NEAR/3 (injur* or trauma)):ti,ab,kw 3816

#5 (concussion* or post-concussion* or postconcussion* or post concussion*):ti,ab,kw 754

#6 (concussive or post-concussive or postconcussive or "post concussive" or sport-concuss* or contusion*):ti,ab,kw 1316

#7 (TBI or mTBI):ti,ab,kw 2775

#8 #1 or #2 or #3 or #4 or #5 or #6 or #7 5864

#9 MeSH descriptor: [Exercise Therapy] explode all trees 12782

#10 MeSH descriptor: [Exercise] explode all trees 23147

#11 MeSH descriptor: [Sports] explode all trees 15040

#12 MeSH descriptor: [Physical Therapy Modalities] explode all trees24008

#13 MeSH descriptor: [Occupational Therapy] explode all trees 728

#14 MeSH descriptor: [Physical Exertion] explode all trees 3806

#15 MeSH descriptor: [Muscle Strength] explode all trees 5298

#16 MeSH descriptor: [Exercise Movement Techniques] explode all trees 1975

#17 MeSH descriptor: [Hydrotherapy] explode all trees 1519

#18 MeSH descriptor: [Weight-Bearing] explode all trees 995

#19 MeSH descriptor: [Tai Ji] explode all trees 334

#20 MeSH descriptor: [Yoga] explode all trees 620

#21 (exercis* or train* or stepped care or physical therap* or physiotherap* or kinesiotherapy or occupational therapy):ti,kw 115734

© 2021 Rytter HM et al. *JAMA Network Open.*

#22 ((muscl* or resistance or physical or endurance or strength* or balance or postur* or gait or aquatic* or trunk or graded or gradual or progressive or controlled or incremented or symptom-based or symptombased or symptom based or guided or supervis* or stepped care or stepwise or step-wise) NEAR/3 (train* or retrain* or re-train* or exercise* or rehabilitation or neurorehabilitation or neuro-rehabilitation or strength* or fit* or condition* or exposure or activit* or exertion or load* or physical therap* or physiotherap* or occupational therapy)):ti,ab,kw 254055

#23 ((graded or gradual or progressive) NEAR/3 (return or return-to-work or return-to sport* or return-to-study or return-to-studies)):ti,ab,kw 66

#24 (aerobic* or anaerobic* or movement therapy or watergym* or water gym* or aquagym or gym* or hydrotherap* or (warm water NEAR/4 (exercis* or train* or rehab*))):ti,ab,kw 27737

#25 (running or Jog* or zumba or kettlebelt or Swim* or Bicycl* or cycling or bicycling or spinning or walk* or rowing or pacing or paced or titreted or (weight* NEAR/3 (lift* or train* or exercis*))):ti,ab,kw 55950

#26 (yoga or tai ji or tai chi or qigong or qi-gong or pilates):ti,ab,kw 4931

#27 ((mckenzie or mechanical or Alexander or William or Feldenkrais) NEAR/2 (technique* or model* or tool* or exercise* or therapy)):ti,ab,kw 578

#28 #9 or #10 or #11 or #12 or #14 or #15 or #16 or #17 or #18 or #19 or #20 or #21 or #22 or #23 or #24 or #25 or #26 or #27 327552

#29 #8 AND #28 with Publication Year from 2016 to 2020, in Trials 880

Cinahl

S60	S44 AND S59
S59	S45 OR S46 OR S47 OR S48 OR S49 OR S50 OR S51 OR S52 OR S53 OR S54 OR S55 OR S56 OR S57 OR S58
S58	SU ((mckenzie or mechanical or Alexander or William or Feldenkrais) N2 (technique* or model* or tool* or exercise* or therapy))
S57	TI ((mckenzie or mechanical or Alexander or William or Feldenkrais) N2 (technique* or model* or tool* or exercise* or therapy))
S56	SU (yoga or tai ji or tai chi or qigong or qi-gong or pilates)
S55	TI (yoga or tai ji or tai chi or qigong or qi-gong or pilates)
S54	SU (active rehabilitation or aerobic* or anaerobic* or movement therapy or watergym* or water gym* or aquagym or gym* or hydrotherap* or (warm water N4 (exercis* or train* or rehab*)))
S53	TI (active rehabilitation or aerobic* or anaerobic* or movement therapy or watergym* or water gym* or aquagym or gym* or hydrotherap* or (warm water N4 (exercis* or train* or rehab*)))

© 2021 Rytter HM et al. *JAMA Network Open.*

S52	SU (running or Jog* or zumba or kettlebelt or Swim* or Bicycl* or cycling or bicycling or spinning or walk* or rowing or pacing or paced or titreted or (weight* N3 (lift* or train* or exercis*)))
S51	TI (running or Jog* or zumba or kettlebelt or Swim* or Bicycl* or cycling or bicycling or spinning or walk* or rowing or pacing or paced or titreted or (weight* N3 (lift* or train* or exercis*)))
S50	SU ((graded or gradual or progressive) N3 (return or return-to-work or return-to-sport* or return-to-study or return-to-studies or return-to-activit*))
S49	TI ((graded or gradual or progressive) N3 (return or return-to-work or return-to-sport* or return-to-study or return-to-studies or return-to-activit*))
S48	SU ((muscl* or resistance or physical or endurance or strength* or balance or postur* or threshold* or treadmill* or aquatic* or trunk or graded or gradual or progressive or controlled or incremented or symptom-based or symptombased or symptom based or guided or supervis* or stepped care or stepwise or step-wise) N3 (train* or retrain* or re-train* or exercise* or rehabilitation or strength* or fit* or condition* or exposure or activit* or exertion or load* or physical therap* or physiotherap* or occupational therapy))
S47	TI ((muscl* or resistance or physical or endurance or strength* or balance or postur* or threshold* or treadmill* or aquatic* or trunk or graded or gradual or progressive or controlled or incremented or symptom-based or symptombased or symptom based or guided or supervis* or stepped care or stepwise or step-wise) N3 (train* or retrain* or re-train* or exercise* or rehabilitation or strength* or fit* or condition* or exposure or activit* or exertion or load* or physical therap* or physiotherap* or occupational therapy))
S46	TI (exercis* or train* or stepped care or physical therap* or physiotherap* or kinesiotherapy or occupational therapy)
S45	MH ("Exercise+" OR "Exercise therapy+" OR "Sports+" OR "Physical Exertion+" OR "Muscle strength+" OR "Exercise Movement Techniques+" OR "physical therapy modalities+" OR "Occupational therapy" OR "Physical Education and Training+" OR "Hydrotherapy+" OR "Weight-Bearing" OR "Ta ji+" OR "Yoga+")
S44	S37 OR S38 OR S39 OR S40 OR S41 OR S42 OR S43
S43	TBI or mTBI
S42	(concussive or post-concussive or postconcussive or post concussive or sport#-concuss* or contusion*)
S41	(concussion* or post-concussion* or postconcussion* or post concussion)
S40	((traumatic or mild or moderat* or minor) N3 (brain or head) N3 (injur* or trauma))
S39	MH "Brain Contusions"

© 2021 Rytter HM et al. *JAMA Network Open.*

S38	(MH "Brain Injuries")
S37	(MH "Brain Concussion+")

PEDRO

Search date: 14.02.2020

PICOs searched combined

Abstract & Title: Concussi* OR post-concussi* OR postconcussi* OR "traumatic brain injury" OR "traumatic head injury"

Method: Clinical trial

Published since: All years

OT Seeker

Search date: 14.02.2020

PICOs searched combined

Any Field: ("traumatic brain injur*" OR "traumatic head injur*" OR concussi* OR post-concussi* OR postconcussi* or "post concussi*" or sports-concuss* or contusion*)

Method: Randomized Controlled Trial

PICO 3: Should patients with persistent vestibular dysfunction after concussion be offered vestibular rehabilitation?

Search date: 03.01.2020

Medline

#	Searches
1	exp brain injuries, traumatic/
2	exp brain concussion/
3	((traumatic or mild or moderat* or minor) adj3 (brain or head) adj3 (injur* or trauma)).ti,bt,ab,kw,kf.
4	(concussion* or post-concussion* or postconcussion* or post concussion*).ti,bt,ab,kw,kf.
5	(concussive or post-concussive or postconcussive or post concussive or sport?-concuss* or sport?-related or contusion*).ti,bt,ab,kw,kf.

© 2021 Rytter HM et al. *JAMA Network Open.*

6	(TBI or PCS).ti,bt,ab,kw,kf.
7	or/1-6
8	exp Vestibular Diseases/
9	exp vertigo/
10	dizziness/
11	perilymph/ and fistula/
12	(vertigo or dizzyness or dizziness or vestibulopath* or ((vestibular or cervicovestibular or cervico-vestibular or balance*) adj6 (disorder or hypofunction* or dysfunction* or impair* or disability* or pathology* or disturbance*))).ti,bt,ab,kw,kf.
13	((vestibular or cervicovestibular or cervico-vestibular) adj6 (rehabilitation or adaptation or habituation)).ti,bt,ab,kw,kf.
14	(neurolabyrinthitides or neurolabyrinthitis or (vestibular and (neuritis or neuronitis or neuritides))).ti,bt,ab,kw,kf.
15	((acoustic adj neuroma) or (acoustic adj neurinoma) or (acoustic adj neurilemoma) or (acoustic adj neurilemmoma) or (vestibular adj schwannoma) or (acoustic adj schwannoma) or (motion adj sensitivity) or (vestibular and peripheral) or (perilymphatic and fistula) or meniere* or (endolymphatic and hydrops) or (labyrinth* and hydrops) or (labyrinth* and syndrome) or bpv or bppv or antbppv).ti,bt,ab,kw,kf.
16	or/8-15
17	Rehabilitation, Vocational/
18	exp Exercise therapy/
19	exp exercise/
20	exp head movement/
21	exp physical therapy modalities/
22	Neurological Rehabilitation/
23	(rehabilitation or neurorehabilitation or neuro-rehabilitation or physiotherap* or (physical and therap*) or exercis* or habituat* or epley or canalith or semont or manoeuvre* or maneuver* or (reconditioning adj activit*) or posturography or ((posture or postural or sensory) adj6 (control* or learn* or relearn* or train* or retrain* or exercise)) or pfpp).ti,bt,ab,kw,kf.
24	((position* and procedure*) or (reposition* and procedure*) or (reposition* and particle*) or (visual and vestibular) or (functional and retrain*) or (occupational and retrain*) or (occupational and adaptation) or (cooksey and cawthorne)).ti,bt,ab,kw,kf.

© 2021 Rytter HM et al. *JAMA Network Open.*

25	or/17-24
26	7 and 16 and 25

Embase

#	Searches
1	exp traumatic brain injury/
2	exp brain concussion/
3	exp brain contusion/
4	exp postconcussion syndrome/
5	((traumatic or mild or moderat* or minor) adj3 (brain or head) adj3 (injur* or trauma)).ti,ab,kw.
6	(concussion* or post-concussion* or postconcussion* or post concussion*).ti,ab,kw.
7	(concussive or post-concussive or postconcussive or post concussive or sport?-concuss* or contusion*).ti,ab,kw.
8	(TBI or PCS).ti,ab,kw.
9	or/1-8
10	exp vestibular disorder/
11	exp vertigo/
12	dizziness/
13	perilymph/ and fistula/
14	(vertigo or dizzyness or dizziness or vestibulopath* or ((vestibular or cervicovestibular or cervico-vestibular or balance*) adj6 (disorder or hypofunction* or dysfunction* or impair* or disability* or pathology* or disturbance*))).ti,ab,kw.
15	((vestibular or cervicovestibular or cervico-vestibular) adj6 (rehabilitation or adaptation or habituation)).ti,ab,kw.
16	(neurolabyrinthitides or neurolabyrinthitis or (vestibular and (neuritis or neuronitis or neuritides))).ti,ab,kw.
17	((acoustic adj neuroma) or (acoustic adj neurinoma) or (acoustic adj neurilemoma) or (acoustic adj neurilemmoma) or (vestibular adj schwannoma) or (acoustic adj schwannoma) or (motion adj sensitivity) or (vestibular and peripheral) or (perilymphatic and fistula) or meniere* or (endolymphatic and hydrops) or (labyrinth* and hydrops) or (labyrinth* and syndrome) or bpv or bppv or antbppv).ti,ab,kw.
18	or/10-17

© 2021 Rytter HM et al. *JAMA Network Open.*

19	vocational rehabilitation/
20	exp kinesiotherapy/
21	exp exercise/
22	exp head movement/
23	exp physical therapy modalities/
24	neurorehabilitation/
25	(rehabilitation or neurorehabilitation or neuro-rehabilitation or physiotherap* or (physical and therap*) or exercis* or habituat* or epley or canalith or semont or manoeuvre* or maneuver* or (reconditioning adj activit*) or posturography or ((posture or postural or sensory) adj6 (control* or learn* or relearn* or train* or retrain* or exercise)) or pfpp).ti,ab,kw.
26	((position* and procedure*) or (reposition* and procedure*) or (reposition* and particle*) or (visual and vestibular) or (functional and retrain*) or (occupational and retrain*) or (occupational and adaptation) or (cooksey and cawthorne)).ti,ab,kw.
27	or/19-26
28	9 and 18 and 27

Cochrane Central

ID Search Hits

#1 MeSH descriptor: [Brain Injuries, Traumatic] explode all trees 600

#2 MeSH descriptor: [Brain Concussion] explode all trees 301

#3 MeSH descriptor: [Post-Concussion Syndrome] explode all trees 118

#4 ((traumatic or mild or moderat* or minor) NEAR/3 (brain or head) NEAR/3 (injur* or trauma)):ti,ab,kw 3679

#5 (concussion* or post-concussion* or postconcussion* or post concussion*):ti,ab,kw 712

#6 (concussive or post-concussive or postconcussive or "post concussive" or sport-concuss* or sport-concuss* or contusion*):ti,ab,kw 1287

#7 (TBI or PCS):ti,ab,kw 4661

#8 #1 or #2 or #3 or #4 or #5 or #6 or #7 7690

#9 MeSH descriptor: [Vestibular Diseases] explode all trees 594

#10 MeSH descriptor: [Vertigo] explode all trees 455

#11 MeSH descriptor: [Dizziness] explode all trees 692

#12 MeSH descriptor: [Perilymph] explode all trees 4

#13 MeSH descriptor: [Fistula] explode all trees 657

#14 #12 AND #13 0

#15 (vertigo or dizzyness or dizziness or vestibulopath* or ((vestibular or cervicovestibular or cervico-vestibular or balance*) NEAR/6 (disorder or hypofunction* or dysfunction* or impair* or disability* or pathology* or disturbance*))):ti,ab,kw 18030

#16 ((vestibular or cervicovestibular or cervico-vestibular) NEAR/6 (rehabilitation or adaptation or habituation)):ti,ab,kw 364

#17 (neurolabyrinthitides or neurolabyrinthitis or (vestibular and (neuritis or neuronitis or neuritides))):ti,ab,kw 82

#18 ((acoustic NEAR/1 neuroma) or (acoustic NEAR/1 neurinoma) or (acoustic NEAR/1 neurilemoma) or (acoustic NEAR/1 neurilemmoma) or (vestibular NEAR/1 schwannoma) or (acoustic NEAR/1 schwannoma) or (motion NEAR/1 sensitivity) or (vestibular and peripheral) or (perilymphatic and fistula) or meniere* or (endolymphatic and hydrops) or (labyrinth* and hydrops) or (labyrinth* and syndrome) or bpv or bppv or antbppv):ti,ab,kw 1116

#19 #9 or #10 or #11 or #14 or #15 or #16 or #17 or #18 18722

#20 MeSH descriptor: [Rehabilitation, Vocational] explode all trees 435

#21 MeSH descriptor: [Exercise Therapy] explode all trees 12650

#22 MeSH descriptor: [Exercise] explode all trees 22949

#23 MeSH descriptor: [Head Movements] explode all trees 176

#24 MeSH descriptor: [Physical Therapy Modalities] explode all trees23801

#25 MeSH descriptor: [Neurological Rehabilitation] explode all trees 2334

#26 (rehabilitation or neurorehabilitation or neuro-rehabilitation or physiotherap* or (physical and therap*) or exercis* or habituat* or epley or canalith or semont or manoeuvre* or maneuver* or (reconditioning adj activit*) or posturography or ((posture or postural or sensory) NEAR/6 (control* or learn* or relearn* or train* or retrain* or exercise)) or pfpp):ti,ab,kw 161249

#27 ((position* and procedure*) or (reposition* and procedure*) or (reposition* and particle*) or (visual and vestibular) or (functional and retrain*) or (occupational and retrain*) or (occupational and adaptation) or (cooksey and cawthorne)):ti,ab,kw 8035

#28 #20 or #21 or #22 or #23 or #24 or #25 or #26 or #27 174621

#29 #8 AND #19 AND #28 in Trials 99

Cinahl

#	Query
S18	S8 AND S17

© 2021 Rytter HM et al. *JAMA Network Open.*

S17	S9 OR S10 OR S11 OR S12 OR S13 OR S14 OR S15 OR S16
S16	((acoustic N1 neuroma) or (acoustic N1 neurinoma) or (acoustic N1 neurilemoma) or (acoustic N1 neurilemmoma) or (vestibular N1 schwannoma) or (acoustic N1 schwannoma) or (motion N1 sensitivity) or (vestibular and peripheral) or (perilymphatic and fistula) or meniere* or (endolymphatic and hydrops) or (labyrinth* and hydrops) or (labyrinth* and syndrome) or bpv or bppv or antbppv)
S15	(neurolabyrinthitides or neurolabyrinthitis or (vestibular and (neuritis or neuronitis or neuritides)))
S14	((vestibular or cervicovestibular or cervico-vestibular or balance*) N6 (rehabilitation or adaptation or habituation))
S13	(vertigo or dizzyness or dizziness or vestibulopath* or ((vestibular or cervicovestibular or cervico-vestibular or balance*) N6 (disorder or hypofunction* or dysfunction* or impair* or disability* or pathology* or disturbance*)))
S12	(MH "perilymph") AND (MH "fistula")
S11	MH dizziness
S10	(MH "Vertigo")
S9	MH "Vestibular Diseases"
S8	S1 OR S2 OR S3 OR S4 OR S5 OR S6 OR S7
S7	TBI or PCS
S6	(concussive or post-concussive or postconcussive or post concussive or sport#-concuss* or contusion*)
S5	(concussion* or post-concussion* or postconcussion* or post concussion)
S4	((traumatic or mild or moderat* or minor) N3 (brain or head) N3 (injur* or trauma))
S3	MH "Brain Contusions"
S2	(MH "Brain Injuries")
S1	(MH "Brain Concussion+")

PEDRO

PICOs searched combined

© 2021 Rytter HM et al. *JAMA Network Open.*

Abstract & Title: Concussi* OR post-concussi* OR postconcussi* OR "traumatic brain injury" OR "traumatic head injury"

Method: Clinical trial

Published since: All years

OT Seeker

PICOs searched combined

Any Field: ("traumatic brain injur*" OR "traumatic head injur*" OR concussi* OR post-concussi* OR postconcussi* or "post concussi*" or sports-concuss* or contusion*)

Method: Randomized Controlled Trial

PICO 4: Should patients with persistent post-concussive symptoms be offered spinal manual therapy, i.e. mobilization and manipulation of the neck and spine?

Search date: 06.01.2020

Medline

#	Searches
1	exp brain injuries, traumatic/
2	exp brain concussion/
3	((traumatic or mild or moderat* or minor) adj3 (brain or head) adj3 (injur* or trauma)).ti,bt,ab,kw,kf.
4	(concussion* or post-concussion* or postconcussion* or post concussion*).ti,bt,ab,kw,kf.
5	(concussive or post-concussive or postconcussive or post concussive or sport?-concuss* or

© 2021 Rytter HM et al. *JAMA Network Open.*

	contusion*).ti,bt,ab,kw,kf.
6	(TBI or PCS).ti,bt,ab,kw,kf.
7	or/1-6
8	Musculoskeletal Manipulations/
9	Manipulation, Chiropractic/
10	Manipulation, Orthopedic/
11	Manipulation, Osteopathic/
12	Manipulation, Spinal/
13	(adjust* adj6 (chiropract* or spinal or lumbar or cervical or thoracic or instrument* or tool* or electric)).ti,bt,ab,kf.
14	(HVLA or high velocity low amplitude).ti,bt,ab,kw.
15	(manipulat* and (chiropract* or naprapath* or osteopath* or oesteopath* or orthopedic* or orthopaedic*)).ti,bt,ab,kf.
16	(manipulat* and (spinal or spine or low* back or joint* or lumbar or neck or thoracic or cervical or MSK or musculoskeletal or vertebr*)).ti,bt,ab,kf.
17	(manipulat* and (physiotherap* or physical therap*)).ti,bt,ab,kf.
18	(manipulat* and technique*).ti,bt,ab,kw,kf.
19	(mobili?at* and (chiropract* or naprapath* or osteopath* or oesteopath* or orthopedic* or orthopaedic*)).ti,bt,ab,kf.
20	(mobili?at* and (spinal or spine or low* back or joint* or lumbar or neck or thoracic or cervical or MSK or musculoskeletal or vertebr*)).ti,bt,ab,kf.
21	((therap* or treat* or intervention* or manag* or rehabilit* or neurorehabilitation or strateg*) adj6 (manual or manipulat* or mobili?at* or MSK or musculoskeletal)).ti,bt,ab,kf.
22	(traction adj3 (manual or passive or mechanical or non-surgical or nonsurgical)).ti,bt,ab,kf.
23	(flexion-distraction or flexion distraction).ti,bt,ab,kf.
24	(activator adj3 (method* or technique* or instrument*)).ti,bt,ab,kf.
25	((mechanical or Alexander or Atlas Orthogonal or Atlas Specific or Bilateral Nasal Specific or Blair Upper Cervical or Chiropractic Manipulative Reflex or Cox or Derifield-Thompson or Directional Non-Force or Diversified or Endo-Nasal or feldenkrais or William or Flexion-Distraction or Full Spine or Gonstead or Grostic or Harrison or Koren Specific or Logan Basic or McTimoney or Maitland or Mulligan or muligan or Neuro Emotional or NUCCA or Palmer or Pierce-Stillwagon or Sacro Occipital or Thompson Terminal Point or Toftness or Toggle Recoil or Torque Release

© 2021 Rytter HM et al. *JAMA Network Open.*

	or Webster or muscle energy) adj1 (Technique* or model* or tool* or exercise* or therapy)).ti,bt,ab,kw,kf.
26	(reflexolog* or cyriax or High velocity or low amplitude or Subluxation or Joint dysfunction or hypomobility or muscle energy technique* or therapeutic touch or stretching).ti,bt,ab,kw,kf.
27	(vibration adj5 (therap* or treatment*)).ti,bt,ab,kw,kf.
28	or/8-27
29	7 and 28

Embase

#	Searches
1	exp traumatic brain injury/
2	exp brain concussion/
3	exp brain contusion/
4	exp postconcussion syndrome/
5	((traumatic or mild or moderat* or minor) adj3 (brain or head) adj3 (injur* or trauma)).ti,ab,kw.
6	(concussion* or post-concussion* or postconcussion* or post concussion*).ti,ab,kw.
7	(concussive or post-concussive or postconcussive or post concussive or sport?-concuss* or contusion*).ti,ab,kw.
8	(TBI or PCS).ti,ab,kw.
9	or/1-8
10	exp musculoskeletal manipulation/
11	(adjust* adj6 (chiropract* or spinal or lumbar or cervical or thoracic or instrument* or tool* or electric)).ti,ab,kw.
12	(HVLA or high velocity low amplitude).ti,ab,kw.
13	(manipulat* and (chiropract* or naprapath* or oesteopath* or osteopath* or osteopath* or orthopedic* or orthopaedic*)).ti,ab,kw.
14	(manipulat* and (spinal or spine or low* back or joint* or lumbar or neck or thoracic or cervical or MSK or musculoskeletal or vertebr*)).ti,ab,kw.
15	(manipulat* and (physiotherap* or physical therap*)).ti,ab,kw.
16	(manipulat* and technique*).ti,ab,kw.

17	(mobili?at* and (chiropract* or naprapath* or osteopath* or osteopath* or orthopedic* or orthopaedic*)).ti,ab,kw.
18	((therap* or treat* or intervention* or manag* or rehabilit* or neurorehabilitation or strateg*) adj6 (manual or manipulat* or mobili?at* or MSK or musculoskeletal)).ti,ab,kw.
19	(traction adj3 (manual or passive or mechanical or non-surgical or nonsurgical)).ti,ab,kw.
20	(flexion-distraction or flexion distraction).ti,ab,kw.
21	(activator adj3 (method* or technique* or instrument*)).ti,ab,kw.
22	((mechanical or Alexander or Atlas Orthogonal or Atlas Specific or Bilateral Nasal Specific or Blair Upper Cervical or Chiropractic Manipulative Reflex or Cox or Derifield-Thompson or Directional Non-Force or Diversified or Endo-Nasal or feldenkrais or William or Flexion-Distraction or Full Spine or Gonstead or Grostic or Harrison or Koren Specific or Logan Basic or McTimoney or Maitland or Mulligan or muligan or Neuro Emotional or NUCCA or Palmer or Pierce-Stillwagon or Sacro Occipital or Thompson Terminal Point or Toftness or Toggle Recoil or Torque Release or Webster or muscle energy) adj1 (Technique* or model* or tool* or exercise* or therapy)).ti,ab,kw.
23	(reflexolog* or cyriax or High velocity or low amplitude or Subluxation or Joint dysfunction or hypomobility or muscle energy technique* or therapeutic touch or stretching).ti,ab,kw.
24	or/10-23
25	9 and 24

Cochrane Central

ID Search Hits

#1 MeSH descriptor: [Brain Injuries, Traumatic] explode all trees 600

#2 MeSH descriptor: [Brain Concussion] explode all trees 301

#3 MeSH descriptor: [Post-Concussion Syndrome] explode all trees 118

#4 ((traumatic or mild or moderat* or minor) NEAR/3 (brain or head) NEAR/3 (injur* or trauma)):ti,ab,kw 3679

#5 (concussion* or post-concussion* or postconcussion* or post concussion*):ti,ab,kw 712

#6 (concussive or post-concussive or postconcussive or "post concussive" or sport-concuss* or sports-concuss* or contusion*):ti,ab,kw 1298

#7 (TBI or PCS):ti,ab,kw 4661

#8 #1 or #2 or #3 or #4 or #5 or #6 or #7 7690

#9 MeSH descriptor: [Musculoskeletal Manipulations] explode all trees 2730

#10 (adjust* NEAR/6 (chiropract* or spinal or lumbar or cervical or thoracic or instrument* or tool* or electric)):ti,ab,kw423

© 2021 Rytter HM et al. *JAMA Network Open.*

#11 (HVLA or high velocity low amplitude):ti,ab,kw 223

#12 (manipulat* and (chiropract* or naprapath* or oesteopath* or osteopath* or osteopath* or orthopedic* or orthopaedic*)):ti,ab,kw 1335

#13 (manipulat* and (spinal or spine or low* back or joint* or lumbar or neck or thoracic or cervical or MSK or musculoskeletal or vertebr*)):ti,ab,kw 3356

#14 (manipulat* and (physiotherap* or physical therap*)):ti,ab,kw 1410

#15 (manipulat* and technique*):ti,ab,kw 1644

#16 (mobili?at* and (chiropract* or naprapath* or osteopath* or osteopath* or orthopedic* or orthopaedic*)):ti,ab,kw 863

#17 ((therap* or treat* or intervention* or manag* or rehabilit* or neurorehabilitation or strateg*) NEAR/6 (manual or manipulat* or mobili?at* or MSK or musculoskeletal)):ti,ab,kw12303

#18 (traction NEAR/3 (manual or passive or mechanical or non-surgical or nonsurgical)):ti,ab,kw 122

#19 (flexion-distraction or flexion distraction):ti,ab,kw 66

#20 (activator NEAR/3 (method* or technique* or instrument*)):ti,ab,kw 53

#21 ((mechanical or Alexander or Atlas Orthogonal or Atlas Specific or Bilateral Nasal Specific or Blair Upper Cervical or Chiropractic Manipulative Reflex or Cox or Derifield-Thompson or Directional Non-Force or Diversified or Endo-Nasal or feldenkrais or William or Flexion-Distraction or Full Spine or Gonstead or Grostic or Harrison or Koren Specific or Logan Basic or McTimoney or Maitland or Mulligan or muligan or Neuro Emotional or NUCCA or Palmer or Pierce-Stillwagon or Sacro Occipital or Thompson Terminal Point or Toftness or Toggle Recoil or Torque Release or Webster or muscle energy) NEAR/1 (Technique* or model* or tool* or exercise* or therapy)):ti,ab,kw 9285

#22 (reflexolog* or cyriax or High velocity or low amplitude or Subluxation or Joint dysfunction or hypomobility or muscle energy technique* or therapeutic touch or stretching):ti,ab,kw 11821

#23 #9 or #10 or #11 or #12 or #13 or #14 or #15 or #16 or #17 or #18 or #19 or #20 or #21 or #22 35471

#24 #8 AND #23 in Trials 308

Cinahl

#	Query
S18	S8 AND S17
S17	S9 OR S10 OR S11 OR S12 OR S13 OR S14 OR S15 OR S16
S16	((reflexolog* or cyriax or High velocity or low amplitude or Subluxation or Joint dysfunction or hypomobility or muscle energy technique* or therapeutic touch or stretching)) or (vibration N5 (therap* or treatment*))
S15	((mechanical or Alexander or Atlas Orthogonal or Atlas Specific or Bilateral Nasal Specific or Blair Upper Cervical or Chiropractic Manipulative Reflex or Cox or Derifield-Thompson or Directional

© 2021 Rytter HM et al. *JAMA Network Open.*

	Non-Force or Diversified or Endo-Nasal or feldenkrais or William or Flexion-Distraction or Full Spine or Gonstead or Grostic or Harrison or Koren Specific or Logan Basic or McTimoney or Maitland or Mulligan or muligan or Neuro Emotional or NUCCA or Palmer or Pierce-Stillwagon or Sacro Occipital or Thompson Terminal Point or Toftness or Toggle Recoil or Torque Release or Webster or muscle energy) N1 (Technique* or model* or tool* or exercise* or therapy))
S14	((therap* or treat* or intervention* or manag* or rehabilit* or neurorehabilitation or strateg*) N6 (manual or manipulat* or mobili#at* or MSK or musculoskeletal)) or ((traction N3 (manual or passive or mechanical or non-surgical or nonsurgical))) or (activator N3 (method* or technique* or instrument*))
S13	(mobili#at* and (chiropract* or naprapath* or osteopath* or oesteopath* or orthopedic* or orthopaedic* or spinal or spine or low* back or joint* or lumbar or neck or thoracic or cervical or MSK or musculoskeletal or vertebr*))
S12	(manipulat* and (chiropract* or naprapath* or osteopath* or oesteopath* or orthopedic* or orthopaedic* or spinal or spine or low* back or joint* or lumbar or neck or thoracic or cervical or MSK or musculoskeletal or vertebr* or physiotherap* or physical therap* or technique*))
S11	(HVLA or high velocity low amplitude or flexion-distraction or flexion distraction)
S10	(adjust* N6 (chiropract* or spinal or lumbar or cervical or neck* or thoracic or instrument* or tool* or electric))
S9	MH "Manual therapy+"
S8	S1 OR S2 OR S3 OR S4 OR S5 OR S6 OR S7
S7	TBI or PCS
S6	(concussive or post-concussive or postconcussive or post concussive or sport#-concuss* or contusion*)
S5	(concussion* or post-concussion* or postconcussion* or post concussion)
S4	((traumatic or mild or moderat* or minor) N3 (brain or head) N3 (injur* or trauma))
S3	MH "Brain Contusions"
S2	(MH "Brain Injuries")
S1	(MH "Brain Concussion+")

PEDRO

© 2021 Rytter HM et al. *JAMA Network Open.*

PICOs searched combined

Abstract & Title: Concussi* OR post-concussi* OR postconcussi* OR "traumatic brain injury" OR "traumatic head injury"

Method: Clinical trial

Published since: All years

OT Seeker

PICOs searched combined

Any Field: ("traumatic brain injur*" OR "traumatic head injur*" OR concussi* OR post-concussi* OR postconcussi* or "post concussi*" or sports-concuss* or contusion*)

Method: Randomized Controlled Trial

PICO 5: Should patients with persistent visual symptoms after concussion be offered oculomotor vision therapy?

Search date: 03.01.2020

Medline

#	Searches
1	exp brain injuries, traumatic/
2	exp brain concussion/
3	((traumatic or mild or moderat* or minor) adj3 (brain or head) adj3 (injur* or trauma)).ti,bt,ab,kw,kf.
4	(concussion* or post-concussion* or postconcussion* or post concussion*).ti,bt,ab,kw,kf.
5	(concussive or post-concussive or postconcussive or post concussive or sport?-concuss* or contusion*).ti,bt,ab,kw,kf.
6	(TBI or PCS).ti,bt,ab,kw,kf.
7	or/1-6
8	exp Visual Perception/
9	exp Eye Movements/
10	Visual cortex/

© 2021 Rytter HM et al. *JAMA Network Open.*

11	Evoked Potentials, Visual/
12	exp Eye Manifestations/
13	exp Visual pathways/
14	Oculomotor Nerve Injuries/
15	Reflex, Vestibulo-Ocular/
16	Orthoptics/
17	(binocular vision or ((vision or visual or visual-evoked or visual-related or vision-evoked or vision-related or optical or binocular or convergence or vergence or saccadic or oculomotor* or oculo-motor* or accommodative or eye movement* or reading) adj6 (therap* or treat* or exercise* or train* or learn* or relearn* or rehabilitat* or neuro-rehabilitation or neurorehabilitation or coordination or attention or potential* or active* or dysfunction* or insufficien* or disorder* or defici* or symptom* or impairment* or injur*))).ti,bt,ab,kw,kf.
18	(Orthoptic* or Vergence or accommodation or eye movement* or Eye Manifestations or visual pathway* or visual nervous system or visual perception or visual cortex or "V1" or Striate Cortex or Extrastriate Cortex or oculomotor system or oculo-motor system or Vestibo-Ocular reflex* or Vestibuloocular or "Near point of convergence" or Convergence amplitude* or Fusional vergence or fusional reserve* or Vergence facility or Vergence flexibility or Stereopsis or Stereoacuity or "Amplitude of accommodation" or Accommodative amplitude* or "facility of accommodation" or Accommodative facility or Developmental Eye Movement test or "Visual search and Attention Test" or Convergence insufficiency symptom survey or Vergence latency or Vergence peek velocity or Vergence accuracy or Accommodative latency or Accommodative peek velocity or Accommodative accuracy or Eye tracking or Versional peek velocity or Fixation stability or Versional latency or VEP).ti,bt,ab,kw,kf.
19	or/8-18
20	7 and 19

Embase

#	Searches
1	exp traumatic brain injury/
2	exp brain concussion/
3	exp brain contusion/
4	exp postconcussion syndrome/
5	((traumatic or mild or moderat* or minor) adj3 (brain or head) adj3 (injur* or trauma)).ti,ab,kw.

© 2021 Rytter HM et al. *JAMA Network Open.*

6	(concussion* or post-concussion* or postconcussion* or post concussion*).ti,ab,kw.
7	(concussive or post-concussive or postconcussive or post concussive or sport?-concuss* or contusion*).ti,ab,kw.
8	(TBI or PCS).ti,ab,kw.
9	or/1-8
10	exp Binocular vision/
11	exp Eye Movements/
12	Visual cortex/
13	Visual Evoked Potential/
14	Eye manifestations/
15	exp Oculomotor Nerve Injury/
16	exp visual nervous system/
17	Vestibuloocular Reflex/
18	Orthoptics/
19	(binocular vision or ((vision or visual or visual-evoked or visual-related or vision-evoked or vision-related or optical or binocular or convergence or vergence or saccadic or oculomotor* or oculo-motor* or accommodative or eye movement* or reading) adj6 (therap* or treat* or exercise* or train* or learn* or relearn* or rehabilitat* or neuro-rehabilitation or neurorehabilitation or coordinat* or attention or potential* or active* or dysfunction* or insufficien* or disorder* or defici* or symptom* or impairment* or injur*))).ti,ab,kw.
20	limit 19 to last 2 years
21	(Orthoptic* or Vergence or accommodation or eye movement* or Eye Manifestations or visual pathway* or visual nervous system or vestibular ocular response or visual cortex or "V1" or Striate Cortex or Extrastriate Cortex or oculomotor system or oculo-motor system or Vestibo-Ocular reflex* or Vestibuloocular or "Near point of convergence" or Convergence amplitude* or Fusional vergence or fusional reserve* or Vergence facility or Vergence flexibility or Stereopsis or Stereoacuity or "Amplitude of accommodation" or Accommodative amplitude* or "facility of accommodation" or Accommodative facility or Developmental Eye Movement test or "Visual search and Attention Test" or Convergence insufficiency symptom survey or Vergence latency or Vergence peek velocity or Vergence accuracy or Accommodative latency or Accommodative peek velocity or Accommodative accuracy or Eye tracking or Versional peek velocity or Fixation stability or Versional latency or VEP).ti,ab,kw.
22	or/10-18,20-21
23	9 and 22

© 2021 Rytter HM et al. *JAMA Network Open.*

Cochrane Central

ID	Search	Hits
#1	MeSH descriptor: [Brain Injuries, Traumatic] explode all trees	600
#2	MeSH descriptor: [Brain Concussion] explode all trees	301
#3	MeSH descriptor: [Post-Concussion Syndrome] explode all trees	118
#4	((traumatic or mild or moderat* or minor) NEAR/3 (brain or head) NEAR/3 (injur* or trauma)):ti,ab,kw	3679
#5	(concussion* or post-concussion* or postconcussion* or post concussion*):ti,ab,kw	712
#6	(concussive or post-concussive or postconcussive or "post concussive" or sport-concuss* or sports-concuss* or contusion*):ti,ab,kw	1287
#7	(TBI or PCS):ti,ab,kw	4661
#8	#1 or #2 or #3 or #4 or #5 or #6 or #7	7690
#9	MeSH descriptor: [Visual Perception] explode all trees	9386
#10	MeSH descriptor: [Eye Movements] explode all trees	1320
#11	MeSH descriptor: [Visual Cortex] explode all trees	226
#12	MeSH descriptor: [Evoked Potentials, Visual] this term only	478
#13	MeSH descriptor: [Eye Manifestations] explode all trees	138
#14	MeSH descriptor: [Visual Pathways] explode all trees	102
#15	MeSH descriptor: [Oculomotor Nerve Injuries] explode all trees	0
#16	MeSH descriptor: [Reflex, Vestibulo-Ocular] explode all trees	85
#17	MeSH descriptor: [Orthoptics] explode all trees	40
#18	(binocular vision or ((vision or visual or visual-evoked or visual-related or vision-evoked or vision-related or optical or binocular or convergence or vergence or saccadic or oculomotor* or oculo-motor* or accommodative or eye movement* or reading) NEAR/6 (therap* or treat* or exercise* or train* or learn* or relearn* or rehabilitat* or neuro-rehabilitation or neurorehabilitation or coordination or attention or potential* or active* or dysfunction* or insufficien* or disorder* or defici* or symptom* or impairment* or injur*))):ti,ab,kw	37219
#19	(Orthoptic* or Vergence or accommodation or eye movement* or Eye Manifestations or visual pathway* or visual nervous system or visual perception or visual cortex or "V1" or Striate Cortex or Extrastriate Cortex or oculomotor system or oculo-motor system or Vestibo-Ocular reflex* or Vestibuloocular or "Near point of convergence" or Convergence amplitude* or Fusional vergence or fusional reserve* or Vergence facility or Vergence flexibility or Stereopsis or Stereoacuity or "Amplitude of accommodation" or Accommodative amplitude* or "facility of accommodation" or Accommodative facility or Developmental Eye Movement test or "Visual search and Attention Test" or Convergence insufficiency symptom survey or Vergence latency or Vergence peek velocity or Vergence accuracy or	

© 2021 Rytter HM et al. *JAMA Network Open.*

Accommodative latency or Accommodative peek velocity or Accommodative accuracy or Eye tracking or Versional peek velocity or Fixation stability or Versional latency or VEP):ti,ab,kw 18765

#20 #9 or #10 or #11 or #12 or #13 or #14 or #15 or #16 or #17 or #18 or #1955241

#21 #8 AND #20 in Trials 439

Cinahl

#	Query
S13	S8 AND S12
S12	S9 OR S10 OR S11
S11	(Orthoptic* or Vergence or accommodation or eye movement* or Eye Manifestations or visual pathway* or visual nervous system or visual perception or visual cortex or "V1" or Striate Cortex or Extrastriate Cortex or oculomotor system or oculo-motor system or Vestibo-Ocular reflex* or Vestibuloocular or "Near point of convergence" or Convergence amplitude* or Fusional vergence or fusional reserve* or Vergence facility or Vergence flexibility or Stereopsis or Stereoacuity or "Amplitude of accommodation" or Accommodative amplitude* or "facility of accommodation" or Accommodative facility or Developmental Eye Movement test or "Visual search and Attention Test" or Convergence insufficiency symptom survey or Vergence latency or Vergence peek velocity or Vergence accuracy or Accommodative latency or Accommodative peek velocity or Accommodative accuracy or Eye tracking or Versional peek velocity or Fixation stability or Versional latency or VEP)
S10	(binocular vision or ((vision or visual or visual-evoked or visual-related or vision-evoked or vision-related or optical or binocular or convergence or vergence or saccadic or oculomotor* or oculo-motor* or accommodative or eye movement* or reading) N3 (therap* or treat* or exercise* or train* or learn* or relearn* or rehabilitat* or neuro-rehabilitation or neurorehabilitation or coordination or attention or potential* or active* or dysfunction* or insufficien* or disorder* or defici* or symptom* or impairment* or injur*)))
S9	MH "Visual Perception+" OR MH "Eye Movements+" OR MH Visual cortex OR MH "Evoked Potentials, Visual" OR MH "Visual pathways" OR MH Oculomotor Nerve Injuries OR MH "Reflex, Vestibulo-Ocular" OR MH Orthoptics+
S8	S1 OR S2 OR S3 OR S4 OR S5 OR S6 OR S7
S7	TBI or PCS
S6	(concussive or post-concussive or postconcussive or post concussive or sport#-concuss* or contusion*)
S5	(concussion* or post-concussion* or postconcussion* or post concussion)

S4	((traumatic or mild or moderat* or minor) N3 (brain or head) N3 (injur* or trauma))
S3	MH "Brain Contusions"
S2	(MH "Brain Injuries")
S1	(MH "Brain Concussion+")

PEDRO

PICOs searched combined, 83 found

Abstract & Title: Concussi* OR post-concussi* OR postconcussi* OR "traumatic brain injury" OR "traumatic head injury"

Method: Clinical trial

Published since: All years

OT Seeker

PICOs searched combined, 14 found

Any Field: ("traumatic brain injur*" OR "traumatic head injur*" OR concussi* OR post-concussi* OR postconcussi* or "post concussi*" or sports-concuss* or contusion*)

Method: Randomized Controlled Trial

PICO 6: Should patients with persistent post-concussive symptoms be offered psychological treatment?

PICO 1 and PICO 6 searched combined, search date: 17.02.2020

See PICO 1

PICO 7: Should patients with persistent post-concussive symptoms be offered an interdisciplinary coordinated rehabilitative treatment?

© 2021 Rytter HM et al. *JAMA Network Open.*

Search date: 30.01.2020

Medline

#	Searches
1	brain injuries, traumatic/
2	exp Brain concussion/
3	((traumatic or mild or moderat* or minor) adj3 (brain or head) adj3 (injur* or trauma)).ti,bt,ab,kw,kf.
4	(concussion* or post-concussion* or postconcussion* or post concussion).ti,bt,ab,kw,kf.
5	(concussive or post-concussive or postconcussive or post concussive or sport?-concuss* or contusion*).ti,bt,ab,kw,kf.
6	(TBI or mTBI).ti,bt,ab,kw,kf.
7	or/1-6
8	Rehabilitation/
9	Activities of Daily Living/
10	Neurological rehabilitation/
11	Telerehabilitation/
12	exp Patient-Centered Care/
13	((Multidisciplinary or Multi-disciplinary or Interdisciplinary or Inter-disciplinary or complex or multimodal* or multi-modal* or cross-disciplinary or crossdisciplinary or multi-dimensional or multidimensional or biopsychosocial or bio-psycho-social or multi-facet* or multifacet* or complimentary or comprehensive or multiple or addition*) adj6 (therap* or management* or intervention* or model* or program* or plan or treat* or care or approach* or action* or train* or retrain* or re-train* or learn* or relearn* or re-learn* or rehabilitation or neurorehabilitation or neuro-rehabilitation or counseling or counselling or remediation or physiotherapy or physical therapy or occupational therapy)).ti,bt,ab,kw,kf.
14	(vocational adj1 (rehabilitation or therapy or treatment or program* or plan* or approach or training or neurohabilitation or neuro-rehabilitation)).ti,bt,ab,kw,kf.
15	((return-to-work or return to work or return-to-activit* or return to activit* or return-to-play or return to play or return-to-sport* or return to sport or return-to-learn* or return to learn* or return-to-academic* or return to

© 2021 Rytter HM et al. *JAMA Network Open.*

	academic* or return-to-study or return to study or return-to-studies or return to studies) adj3 (treatment* or intervention* or model* or program* or plan or training or exercise* or rehabilitation or neurorehabilitation or neuro-rehabilitation or counseling or counselling or remediation)).ti,bt,kw,kf.
16	((integrated or integrative or individualized or holistic or relational or tailor*) adj3 (therap* or treatment* or intervention* or management* or model* or program* or plan or care or approach* or action* or training or exercise* or rehabilitation or neurorehabilitation or neuro-rehabilitation or support* or counseling or counselling or remediation)).ti,bt,kw,kf.
17	((personalis* or personaliz* or Person-centred or Person-centered or Person-oriented or Person-focused or Person-based or Person-tailored or Patient-centred or Patient-centered or Patient-oriented or Patient-focused or Patient-based or Patient-tailored or Personally-centred or Personally-centered or Personally-oriented or Personally-focused or Personally-based or Personally-tailor* or Client-centred or Client-centered or Client-oriented or Client-focused or Client-based or Client-tailored or individuali#ed or Individual-centred or Individual-centered or Individual-focused or Individual-oriented or Individual-based or Individual-tailored or individually-centred or individually-centered or Individually-focused or Individually-oriented or Individually-based or Individually-tailored or relationship-centred or relationship-centered) adj3 (therap* or treatment* or intervention* or management* or model* or program* or plan or care or approach* or action* or training or exercise* or rehabilitation or neurorehabilitation or neuro-rehabilitation or counseling or counselling or remediation)).ti,bt,kw,kf.
18	((Person* or patient* or client* or individual*) adj1 (centred or center* or orient* or focus* or based or tailor*) adj2 (therap* or treatment* or intervention* or management* or model* or program* or plan or care or approach* or action* or training or exercise* or rehabilitation or neurorehabilitation or neuro-rehabilitation or counseling or counselling or remediation)).ti,bt,kw,kf.
19	(("activities of daily living" or daily life activit* or daily living activit*) and (therap* or treatment* or intervention* or management* or model* or program* or plan or care or approach* or action* or training or exercise* or rehabilitation or neurorehabilitation or neuro-rehabilitation or support* or counseling or counselling or remediation)).ti,bt,kw,kf.
20	or/8-19
21	7 and 20

Embase

#	Searches
1	traumatic brain injury/
2	exp brain concussion/

© 2021 Rytter HM et al. *JAMA Network Open.*

3	exp brain contusion/
4	exp postconcussion syndrome/
5	((traumatic or mild or moderat* or minor) adj3 (brain or head) adj3 (injur* or trauma)).ti,ab,kw.
6	(concussion* or post-concussion* or postconcussion* or post concussion*).ti,ab,kw.
7	(concussive or post-concussive or postconcussive or post concussive or sport?-concuss* or contusion*).ti,ab,kw.
8	(TBI or mTBI).ti,ab,kw.
9	or/1-8
10	rehabilitation/ or athletic rehabilitation/ or community based rehabilitation/ or functional training/ or home rehabilitation/ or exp neurorehabilitation/ or rehabilitation care/ or telerehabilitation/ or vocational rehabilitation/ or geriatric rehabilitation/
11	Activities of Daily Living/
12	daily life activity/
13	rehabilitation medicine/
14	multidisciplinary team/
15	community based rehabilitation/
16	holistic care/
17	((Multidisciplinary or Multi-disciplinary or Interdisciplinary or Inter-disciplinary or complex or multimodal* or multi-modal* or cross-disciplinary or crossdisciplinary or multi-dimensional or multidimensional or biopsychosocial or bio-psycho-social or multi-facet* or multifacet* or comprehensive or complimentary or multiple or addition*) adj6 (therap* or management* or intervention* or model* or program* or plan or treat* or care or approach* or action* or train* or retrain* or re-train* or learn* or relearn* or re-learn* or rehabilitation or neurorehabilitation or neuro-rehabilitation or counseling or counselling or remediation or physiotherapy or physical therapy or occupational therapy)).ti,ab,kw.
18	(vocational adj1 (rehabilitation or therapy or treatment or program* or plan* or approach or training or neurohabilitation or neuro-rehabilitation)).ti,ab,kw.
19	((return-to-work or return to work or return-to-activit* or return to activit* or return-to-play or return to play or return-to-sport* or return to sport or return-to-learn* or return to learn* or return-to-academic* or return to academic* or return-to-study or return to study or return-to-studies or return to studies) adj3 (treatment* or intervention* or model* or program* or plan or training or exercise* or rehabilitation or neurorehabilitation or neuro-rehabilitation or counseling or counselling or remediation)).ti,kw.
20	((integrated or integrative or individualized or holistic or relational or tailor*) adj3 (therap* or treatment* or

© 2021 Rytter HM et al. *JAMA Network Open.*

	intervention* or management* or model* or program* or plan or care or approach* or action* or training or exercise* or rehabilitation or neurorehabilitation or neuro-rehabilitation or support* or counseling or counselling or remediation)).ti,kw.
21	((personalis* or personaliz* or Person-centred or Person-centered or Person-oriented or Person-focused or Person-based or Person-tailored or Patient-centred or Patient-centered or Patient-oriented or Patient-focused or Patient-based or Patient-tailored or Personally-centred or Personally-centered or Personally-oriented or Personally-focused or Personally-based or Personally-tailor* or Client-centred or Client-centered or Client-oriented or Client-focused or Client-based or Client-tailored or individuali#ed or Individual-centred or Individual-centered or Individual-focused or Individual-oriented or Individual-based or Individual-tailored or individually-centred or individually-centered or Individually-focused or Individually-oriented or Individually-based or Individually-tailored or relationship-centred or relationship-centered) adj3 (therap* or treatment* or intervention* or management* or model* or program* or plan or care or approach* or action* or training or exercise* or rehabilitation or neurorehabilitation or neuro-rehabilitation or counseling or counselling or remediation)).ti,kw.
22	((Person* or patient* or client* or individual*) adj1 (centred or center* or orient* or focus* or based or tailor*) adj2 (therap* or treatment* or intervention* or management* or model* or program* or plan or care or approach* or action* or training or exercise* or rehabilitation or neurorehabilitation or neuro-rehabilitation or counseling or counselling or remediation)).ti,kw.
23	(("activities of daily living" or daily life activit* or daily living activit*) and (therap* or treatment* or intervention* or management* or model* or program* or plan or care or approach* or action* or training or exercise* or rehabilitation or neurorehabilitation or neuro-rehabilitation or support* or counseling or counselling or remediation)).ti,kw.
24	or/10-23
25	9 and 24

PsycINFO

#	Searches
1	traumatic brain injury/
2	exp Brain concussion/
3	((traumatic or mild or moderat* or minor) adj3 (brain or head) adj3 (injur* or trauma)).ti,ab,id.
4	(concussion* or post-concussion* or postconcussion* or post concussion).ti,ab,id.
5	(concussive or post-concussive or postconcussive or post concussive or sport?-concuss* or contusion*).ti,ab,id.
6	(TBI or mTBI).ti,ab,id.

© 2021 Rytter HM et al. *JAMA Network Open.*

7	or/1-6
8	Interdisciplinary Treatment Approach/
9	rehabilitation/
10	rehabilitation counseling/
11	psychosocial rehabilitation/
12	telerehabilitation/
13	Vocational rehabilitation/
14	Activities of Daily Living/
15	Biopsychosocial Approach/
16	holistic health/
17	((Multidisciplinary or Multi-disciplinary or Interdisciplinary or Inter-disciplinary or complex or multimodal* or multi-modal* or cross-disciplinary or crossdisciplinary or multi-dimensional or multidimensional or biopsychosocial or bio-psycho-social or multi-facet* or multifacet* or comprehensive or complimentary or outpatient* or out-patient* or different or multiple or addition*) adj6 (therap* or management* or intervention* or model* or program* or plan or treat* or care or approach* or action* or interview* or train* or retrain* or re-train* or learn* or relearn* or re-learn* or rehabilitation or neurorehabilitation or neuro-rehabilitation or support* or counseling or counselling or remediation)).ti,ab,id.
18	((return-to-work or return to work or return-to-activit* or return to activit* or return-to-play or return to play or return-to-sport* or return to sport or return-to-learn* or return to learn* or return-to-academic* or return to academic* or return-to-study or return to study or return-to-studies or return to studies) and (therap* or management* or intervention* or model* or program* or plan or treat* or care or approach* or action* or interview* or train* or retrain* or re-train* or learn* or relearn* or re-learn* or rehabilitation or neurorehabilitation or neuro-rehabilitation or support* or counseling or counselling or remediation)).ti,ab,id.
19	(vocational adj1 (rehabilitation or therapy or treatment or program* or plan* or approach or training or neurohabilitation or neuro-rehabilitation)).ti,ab,id.
20	exp Psychosocial rehabilitation/
21	((Psychosocial or psycho-social or neuro-psychologic* or neuropsychologic* or non-pharmacologic* or nonpharmacologic* or non pharmacolog* or non-drug or nondrug or non drug or rehabilitative) adj6 (therap* or treat* or intervention* or program* or plan* or approach* or action* or care or model* or modal* or multimodal* or multi-modal* or interview* or management or training or rehabilitation or support* or counseling or counselling or remediation)).ti,ab,id.
22	((integrated or integrative or individualized or holistic or relational or tailor*) adj3 (therap* or treatment* or

	intervention* or management* or model* or program* or plan or modal* or multimodal* or multi-modal* or treat* or care or approach* or action* or interview* or training or rehabilitation or neurorehabilitation or neuro-rehabilitation or support* or counseling or counselling or remediation)).ti,ab,id.
23	((personalis* or personaliz* or Person-centred or Person-centered or Person-oriented or Person-focused or Person-based or Person-tailored or Patient-centred or Patient-centered or Patient-oriented or Patient-focused or Patient-based or Patient-tailored or Personally-centred or Personally-centered or Personally-oriented or Personally-focused or Personally-based or Personally-tailor* or Client-centred or Client-centered or Client-oriented or Client-focused or Client-based or Client-tailored or individuali#ed or Individual-centred or Individual-centered or Individual-focused or Individual-oriented or Individual-based or Individual-tailored or individually-centred or individually-centered or Individually-focused or Individually-oriented or Individually-based or Individually-tailored or relationship-centred or relationship-centered) adj3 (therap* or treatment* or intervention* or management* or model* or program* or plan or modal* or multimodal* or multi-modal* or treat* or care or approach* or action* or interview* or training or rehabilitation or neurorehabilitation or neuro-rehabilitation or support* or counseling or counselling or remediation)).ti,ab,id.
24	((Person* or patient* or client* or individual*) adj2 (centred or center* or orient* or focus* or based or tailor*) adj6 (therap* or treatment* or intervention or management* or model* or program* or plan or modal* or multimodal* or multi-modal* or treat* or care or approach* or action* or interview* or training or rehabilitation or neurorehabilitation or neuro-rehabilitation or support* or counseling or counselling or remediation)).ti,ab,id.
25	(("activities of daily living" or daily life activit* or daily living activit*) adj6 (therap* or treatment* or intervention* or management* or model* or program* or plan or care or approach* or action* or training or exercise* or rehabilitation or neurorehabilitation or neuro-rehabilitation or support* or counseling or counselling or remediation)).ti,id.
26	or/8-25
27	7 and 26

Cochrane Central

ID Search Hits

#1 MeSH descriptor: [Brain Concussion] explode all trees 327

#2 MeSH descriptor: [Post-Concussion Syndrome] explode all trees 127

#3 ((mild or moderat* or minor) NEAR/3 (brain or head) NEAR/3 (injur* or trauma)):ti,ab,kw 756

#4 (concussion* or post-concussion* or postconcussion* or post concussion*):ti,ab,kw 754

#5 (concussive or post-concussive or postconcussive or "post concussive" or sport-concuss* or sport-concuss* or contusion*):ti,ab,kw 1316

© 2021 Rytter HM et al. *JAMA Network Open.*

#6 (TBI or mTBI):ti,ab,kw 2775

#7 #1 or #2 or #3 or #4 or #5 or #6 4527

#8 ((Multidisciplinary or Multi-disciplinary or Interdisciplinary or Inter-disciplinary or complex or multimodal* or multi-modal* or cross-disciplinary or crossdisciplinary or multi-dimensional or multidimensional or biopsychosocial or bio-psycho-social or multi-facet* or multifacet* or comprehensive or complimentary or multiple or addition*) NEAR/6 (therap* or management* or intervention* or model* or program* or plan or treat* or care or approach* or action* or train* or retrain* or re-train* or learn* or relearn* or re-learn* or rehabilitation or neurorehabilitation or neuro-rehabilitation or counseling or counselling or remediation or physiotherapy or physical therapy or occupational therapy)):ti,ab,kw 91902

#9 (vocational NEAR/1 (rehabilitation or therapy or treatment or program* or plan* or approach or training or neurohabilitation or neuro-rehabilitation)):ti,ab,kw 801

#10 ((return-to-work or return to work or return-to-activit* or return to activit* or return-to-play or return to play or return-to-sport* or return to sport or return-to-learn* or return to learn* or return-to-academic* or return to academic* or return-to-study or return to study or return-to-studies or return to studies) NEAR/3 (treatment* or intervention* or model* or program* or plan or training or exercise* or rehabilitation or neurorehabilitation or neuro-rehabilitation or counseling or counselling or remediation)):ti,ab,kw 363760

#11 ((integrated or integrative or individualized or holistic or relational or tailor*) NEAR/3 (therap* or treatment* or intervention* or management* or model* or program* or plan or care or approach* or action* or training or exercise* or rehabilitation or neurorehabilitation or neuro-rehabilitation or support* or counseling or counselling or remediation)):ti,ab,kw 18734

#12 ((personalis* or personaliz* or Person-centred or Person-centered or Person-oriented or Person-focused or Person-based or Person-tailored or Patient-centred or Patient-centered or Patient-oriented or Patient-focused or Patient-based or Patient-tailored or Personally-centred or Personally-centered or Personally-oriented or Personally-focused or Personally-based or Personally-tailor* or Client-centred or Client-centered or Client-oriented or Client-focused or Client-based or Client-tailored or individuali#ed or Individual-centred or Individual-centered or Individual-focused or Individual-oriented or Individual-based or Individual-tailored or individually-centred or individually-centered or Individually-focused or Individually-oriented or Individually-based or Individually-tailored or relationship-centred or relationship-centered) NEAR/3 (therap* or treatment* or intervention* or management* or model* or program* or plan or care or approach* or action* or training or exercise* or rehabilitation or neurorehabilitation or neuro-rehabilitation or counseling or counselling or remediation)):ti,ab,kw 6720

#13 ((Person* or patient* or client* or individual*) NEAR/1 (centred or center* or orient* or focus* or based or tailor*) NEAR/2 (therap* or treatment* or intervention* or management* or model* or program* or plan or care or approach* or action* or training or exercise* or rehabilitation or neurorehabilitation or neuro-rehabilitation or counseling or counselling or remediation)).ti,ab,kw 1394

#14 (("activities of daily living" or daily life activit* or daily living activit*) and (therap* or treatment* or intervention* or management* or model* or program* or plan or care or approach* or action* or training or exercise* or rehabilitation or neurorehabilitation or neuro-rehabilitation or support* or counseling or counselling or remediation)):ti,ab,kw 18558

#15 #8 or #9 or #10 or #11 or #12 or #13 or #14 425327

#16 #7 AND #15 with Publication Year from 2016 to 2020, in Trials 925

© 2021 Rytter HM et al. *JAMA Network Open.*

Cinahl

#	Query
S24	S8 AND S23
S23	S9 OR S10 OR S11 OR S12 OR S13 OR S14 OR S15 OR S16 OR S17 OR S18 OR S19 OR S20 OR S21 OR S22
S22	SU (("activities of daily living" or daily life activit* or daily living activit*) N6 (therap* or treatment* or intervention* or management* or model* or program* or plan or care or approach* or action* or training or exercise* or rehabilitation or neurorehabilitation or neuro-rehabilitation or support* or counseling or counselling or remediation)) OR TI (("activities of daily living" or daily life activit* or daily living activit*) N6 (therap* or treatment* or intervention* or management* or model* or program* or plan or care or approach* or action* or training or exercise* or rehabilitation or neurorehabilitation or neuro-rehabilitation or support* or counseling or counselling or remediation))
S21	SU ((Person* or patient* or client* or individual*) N2 (centred or center* or orient* or focus* or based or tailor*) N3 (therap* or treatment* or intervention or management* or model* or program* or plan or modal* or multimodal* or multi-modal* or treat* or care or approach* or action* or interview* or training or rehabilitation or neurorehabilitation or neuro-rehabilitation or support* or counseling or counselling or remediation)) OR TI ((Person* or patient* or client* or individual*) N2 (centred or center* or orient* or focus* or based or tailor*) N3 (therap* or treatment* or intervention or management* or model* or program* or plan or modal* or multimodal* or multi-modal* or treat* or care or approach* or action* or interview* or training or rehabilitation or neurorehabilitation or neuro-rehabilitation or support* or counseling or counselling or remediation))
S20	SU ((personalis* or personaliz* or Person-centred or Person-centered or Person-oriented or Person-focused or Person-based or Person-tailored or Patient-centred or Patient-centered or Patient-oriented or Patient-focused or Patient-based or Patient-tailored or Personally-centred or Personally-centered or Personally-oriented or Personally-focused or Personally-based or Personally-tailor* or Client-centred or Client-centered or Client-oriented or Client-focused or Client-based or Client-tailored or individuali#ed or Individual-centred or Individual-centered or Individual-focused or Individual-oriented or Individual-based or Individual-tailored or individually-centred or individually-centered or Individually-focused or Individually-oriented or Individually-based or Individually-tailored or relationship-centred or relationship-centered) N3 (therap* or treatment* or intervention* or management* or model* or program* or plan or modal* or multimodal* or multi-modal* or treat* or care or approach* or action* or interview* or training or rehabilitation or neurorehabilitation or neuro-rehabilitation or support* or counseling or counselling or remediation)) OR TI ((personalis* or personaliz* or Person-centred or Person-centered or Person-oriented or Person-focused or Person-based or Person-tailored or Patient-centred or Patient-centered or Patient-oriented or Patient-focused or Patient-based or Patient-tailored or Personally-centred or Personally-centered or Personally-oriented or Personally-focused

© 2021 Rytter HM et al. *JAMA Network Open.*

	or Personally-based or Personally-tailor* or Client-centred or Client-centered or Client-oriented or Client-focused or Client-based or Client-tailored or individuali#ed or Individual-centred or Individual-centered or Individual-focused or Individual-oriented or Individual-based or Individual-tailored or individually-centred or individually-centered or Individually-focused or Individually-oriented or Individually-based or Individually-tailored or relationship-centred or relationship-centered) N3 (therap* or treatment* or intervention* or management* or model* or program* or plan or modal* or multimodal* or multi-modal* or treat* or care or approach* or action* or interview* or training or rehabilitation or neurorehabilitation or neuro-rehabilitation or support* or counseling or counselling or remediation))
S19	TI ((integrated or integrative or individualized or holistic or relational or tailor*) N3 (therap* or treatment* or intervention* or management* or model* or program* or plan or modal* or multimodal* or multi-modal* or treat* or care or approach* or action* or interview* or training or rehabilitation or neurorehabilitation or neuro-rehabilitation or support* or counseling or counselling or remediation))
S18	SU ((integrated or integrative or individualized or holistic or relational or tailor*) N3 (therap* or treatment* or intervention* or management* or model* or program* or plan or modal* or multimodal* or multi-modal* or treat* or care or approach* or action* or interview* or training or rehabilitation or neurorehabilitation or neuro-rehabilitation or support* or counseling or counselling or remediation))
S17	TI ((Psychosocial or psycho-social or neuro-psychologic* or neuropsychologic* or rehabilitative) N3 (therap* or treat* or intervention* or program* or plan* or approach* or action* or care or model* or modal* or multimodal* or multi-modal* or management or training or rehabilitation or support* or counseling or counselling or remediation))
S16	SU ((Psychosocial or psycho-social or neuro-psychologic* or neuropsychologic* or rehabilitative) N3 (therap* or treat* or intervention* or program* or plan* or approach* or action* or care or model* or modal* or multimodal* or multi-modal* or management or training or rehabilitation or support* or counseling or counselling or remediation))
S15	TI (vocational N1 (rehabilitation or therapy or treatment or program* or plan* or approach or training or neurohabilitation or neuro-rehabilitation))
S14	SU (vocational N1 (rehabilitation or therapy or treatment or program* or plan* or approach or training or neurohabilitation or neuro-rehabilitation))
S13	((Multidisciplinary or Multi-disciplinary or Interdisciplinary or Inter-disciplinary or complex or multimodal* or multi-modal* or cross-disciplinary or crossdisciplinary or multi-dimensional or multidimensional or biopsychosocial or bio-psycho-social or multi-facet* or multifacet* or comprehensive or complimentary or outpatient* or out-patient* or multiple or addition*) N6 (therap* or management* or intervention* or model* or program* or plan or treat* or care or approach* or action* or interview* or train* or retrain* or re-train* or learn* or relearn* or re-learn* or rehabilitation or neurorehabilitation or neuro-rehabilitation or support* or counseling or counselling or remediation or physiotherapy or physical therapy or occupational therapy or physical therapy modalit*))

© 2021 Rytter HM et al. *JAMA Network Open.*

S12	SU ((Multidisciplinary or Multi-disciplinary or Interdisciplinary or Inter-disciplinary or complex or multimodal* or multi-modal* or cross-disciplinary or crossdisciplinary or multi-dimensional or multidimensional or biopsychosocial or bio-psycho-social or multi-facet* or multifacet* or comprehensive or complimentary or outpatient* or out-patient* or multiple or addition*) N6 (therap* or management* or intervention* or model* or program* or plan or treat* or care or approach* or action* or train* or retrain* or re-train* or learn* or relearn* or re-learn* or rehabilitation or neurorehabilitation or neuro-rehabilitation or support* or counseling or counselling or remediation or physiotherapy or physical therapy or occupational therapy or physical therapy modalit*))
S11	MH ("Rehabilitation, athletic" or "Home rehabilitation" or "Rehabilitation, geriatric" or "Rehabilitation research" or "Rehabilitation, vocational" or "Rehabilitation, community-based" or "Rehabilitation nursing" or "Research, interdisciplinary" or "Telerehabilitation" or "Activities of Daily Living")
S10	(MH "Support, Psychosocial+") or (MH "Rehabilitation, Psychosocial+")
S9	MH ("Rehabilitation" or "Multidisciplinary Care Team" or "Holistic Health")
S8	S1 OR S2 OR S3 OR S4 OR S5 OR S6 OR S7
S7	TBI or mTBI
S6	(concussive or post-concussive or postconcussive or post concussive or sport#-concuss* or contusion*)
S5	(concussion* or post-concussion* or postconcussion* or post concussion)
S4	((traumatic or mild or moderat* or minor) N3 (brain or head) N3 (injur* or trauma))
S3	MH "Brain Contusions"
S2	(MH "Brain Injuries")
S1	(MH "Brain Concussion+")

PEDRO

PICOs searched combined

Abstract & Title: Concussi* OR post-concussi* OR postconcussi* OR "traumatic brain injury" OR "traumatic head injury"

Method: Clinical trial

© 2021 Rytter HM et al. *JAMA Network Open.*

Published since: All years

OT Seeker

PICOs searched combined

Any Field: ("traumatic brain injur*" OR "traumatic head injur*" OR concussi* OR post-concussi* OR postconcussi* or "post concussi*" or sports-concuss* or contusion*)

Method: Randomized Controlled Trial

© 2021 Rytter HM et al. *JAMA Network Open.*

eTable 2. Clinical Questions in PICO Form, Including Specified Outcome Measures

PICO 1
Should adults with concussions be offered early intervention in the form of early information and advice?

Population
Persons ≥18 years diagnosed with concussion.

Intervention
Early information, guidance and advice on symptoms, restitution, symptom management and self-care either as telephone counseling, individual or group-based counseling and / or by use of written material, ie. initiated within 4 weeks after concussion by professionals e.g., healthcare professionals for patients with concussion.

Comparison
Treatment as usual

**Outcome measures**	**Time frame**	**Critical/Important**
The collective burden of post-concussion symptoms	*Follow-up (2 weeks after the end of treatment))*	*Critical*
Physical functioning	*Follow-up (1 month after the end of treatment)*	*Important*
Emotional symptoms	*Follow-up (1 month after the end of treatment)*	*Critical*
Behavioral response	*Follow-up (14 days after the end of treatment)*	*Important*

© 2021 Rytter HM et al. *JAMA Network Open.*

Cognitive functioning	*Follow-up (1 month after the end of treatment)*	*Important*
Quality of life	*Follow-up (1 month after the end of treatment)*	*Important*
Pain	*Follow-up (1 month after the end of treatment)*	*Important*
Negative effect on prognosis	*Follow-up (1 month after the end of treatment)*	*Important*
Return to daily activities	*Follow-up (1 month after the end of treatment)*	*Important*

PICO 2

Should adults with symptoms ≥ 4 weeks after concussion be offered graded physical exercise?

Population

Persons ≥18 years diagnosed with concussion suffering from long-term symptoms ≥ 4 weeks after concussion.

Intervention

Graded physical exercise with an increase in intensity/difficulty over time, including but not limited to: Unspecified physiotherapy, unspecified physical activity, neuromotor training, sensorimotor training, aerobic and anaerobic training at least once a week for at least 4 weeks.

Comparison

Exercise, not graded

**Outcome measures**	**Time frame**	**Critical/Important**
The collective burden of post-concussion symptoms	*After the end of treatment*	*Critical*
The collective burden of post-concussion symptoms	*Follow-up (3 months after the end of treatment)*	*Important*
Physical functioning	*After the end of treatment*	*Critical*
Physical functioning	*Follow-up (3 months after the end of treatment)*	*Important*
Emotional symptoms	*Follow-up (3 months after the end of treatment)*	*Important*
Behavioral response	*Follow-up (14 days after the end of treatment)*	*Important*
Quality of life	*Follow-up (3 months after the end of treatment)*	*Important*

© 2021 Rytter HM et al. *JAMA Network Open.*

Pain	*After the end of treatment*	*Important*
Negative effect on prognosis	*After the end of treatment*	*Important*
Return to daily activities	*Follow-up (3 months after the end of treatment)*	*Important*

PICO 3

Should adults suffering from persistent vestibular symptoms ≥ 4 weeks after concussion be offered vestibular rehabilitation?

Population

Persons ≥18 years diagnosed with concussion and with persistent vestibular symptoms ≥ 4 weeks after concussion.

Intervention

Vestibular rehabilitation including otolith repositioning maneuvers, habituation exercises, adaptation exercises, substitution training and balance training at least once a week for 4 weeks.

Comparison

No vestibular rehabilitation.

**Outcome measures**	**Time frame**	**Critical/Important**
The collective burden of post-concussion symptoms	*After the end of treatment*	*Critical*
The collective burden of post-concussion symptoms	*Follow-up (3 months after the end of treatment)*	*Important*
Vestibular dysfunction (Dizziness)	*After the end of treatment*	*Critical*
Vestibular dysfunction (Dizziness)	*Follow-up (3 months after the end of treatment)*	*Important*
Emotional symptoms	*Follow-up (3 months after the end of treatment)*	*Important*
Behavioral response	*Follow-up (14 days after the end of treatment)*	*Important*
Quality of life	*Follow-up (3 months after the end of treatment)*	*Important*
Physical functioning	*After the end of treatment*	*Important*
Physical functioning	*Follow-up (3 months after the end of treatment)*	*Important*
Negative effect on prognosis	*After the end of treatment*	*Important*
Return to daily activities	*Follow-up (3 months after the end of treatment)*	*Important*

© 2021 Rytter HM et al. *JAMA Network Open.*

PICO 4

Should adults suffering from long-term symptoms ≥ 4 weeks after concussion be offered manual treatment with mobilization and manipulation techniques of neck and back?

Population

Persons ≥18 years diagnosed with concussion suffering from long-term symptoms ≥ 4 weeks after concussion.

Intervention

Manual treatment in the form of mobilization and/or manipulation of neck and back. Mobilization is defined as a passive movement in which a clinician moves one or more joints through the active and passive range of motion out to the elastic barrier in one or more directions repeatedly. Manipulation is defined as a passive movement in which a clinician moves one or more joints through the active and passive range of motion to the elastic barrier. The clinician gives the patient a fast impulse with low amplitude, whereby the joint is moved through the elastic barrier, most often accompanied by an audible "crack" (46).

Comparison

No manual treatment.

**Outcome measures**	**Time frame**	**Critical/Important**
The collective burden of post-concussion symptoms	*After the end of treatment*	*Important*
The collective burden of post-concussion symptoms	*Follow-up (3 months after the end of treatment)*	*Important*
Emotional symptoms	*Follow-up (3 months after the end of treatment)*	*Important*
Behavioral response	*Follow-up (14 days after the end of treatment)*	*Important*
Quality of life	*Follow-up (3 months after the end of treatment)*	*Important*
Physical functioning	*After the end of treatment*	*Critical*
Physical functioning	*Follow-up (3 months after the end of treatment)*	*Important*
Pain	*After the end of treatment*	*Critical*
Negative effect on prognosis	*After the end of treatment*	*Important*
Return to daily activities	*After the end of treatment*	*Important*

© 2021 Rytter HM et al. *JAMA Network Open.*

PICO 5

Should adults suffering from long-term visual symptoms ≥ 4 weeks after concussion be offered oculomotor vision therapy?

Population

Persons ≥18 years diagnosed with concussion suffering from long-term visual symptoms ≥ 4 weeks after concussion.

Intervention

Vision therapy focusing on visual symptoms after concussion, i.e. oculomotor vision therapy focusing on vergence and accommodation systems, including computer-based training and optical instrument training. The treatment must be given in a minimum of 1 session per week for a period of at least 4 weeks.

Comparison

No vision training

**Outcome measures**	**Time frame**	**Critical/Important**
The collective burden of post-concussion symptoms	*After the end of treatment*	*Important*
Oculomotor dysfunction at psychophysical examination and objective registration	*After the end of treatment*	*Critical*
Visual symptoms	*After the end of treatment*	*Critical*
Physical functioning	*Follow-up (3 months after the end of treatment)*	*Important*
Emotional symptoms	*Follow-up (3 months after the end of treatment)*	*Important*
Behavioral response	*Follow-up (3 months after the end of treatment)*	*Important*
Cognitive functioning	*After the end of treatment*	*Important*
Quality of life	*follow-up (3 months after the end of treatment)*	*Important*
Negative effect on prognosis	*After the end of treatment*	*Important*
Return to daily activities	*Follow-up (3 months after the end of treatment)*	*Important*

PICO 6

Should adults with long-term symptoms ≥ 4 weeks after concussion be offered psychological treatment?

Population

Persons ≥18 years diagnosed with concussion suffering from long-term symptoms ≥ 4 weeks after concussion.

Intervention

© 2021 Rytter HM et al. *JAMA Network Open.*

Psychological treatment performed by professionals with a professional background of relevance for a minimum of 1 hour per week either as individual or group therapy for at least 4 weeks.

Comparison
No psychological treatment.

**Outcome measures**	**Time frame**	**Critical/Important**
The collective burden of post-concussion symptoms	*After the end of treatment*	*Critical*
The collective burden of post-concussion symptoms	*Follow-up (3 months after the end of treatment)*	*Important*
Physical functioning	*Follow-up (3 months after the end of treatment)*	*Important*
Emotional symptoms	*Follow-up (3 months after the end of treatment)*	*Critical*
Behavioral response	*After the end of treatment*	*Important*
Cognitive functioning	*After the end of treatment*	*Important*
Quality of life	*Follow-up (3 months after the end of treatment)*	*Important*
Negative effect on prognosis	*After the end of treatment*	*Important*
Return to daily activities	*Follow-up (3 months after the end of treatment)*	*Important*

PICO 7

Should adults suffering from long-term symptoms ≥ 4 weeks after concussion be offered an interdisciplinary coordinated rehabilitative treatment?

Population
Persons ≥18 years diagnosed with concussion suffering from long-term symptoms ≥ 4 weeks after concussion.

Intervention
An interdisciplinary coordinated rehabilitative treatment incorporating physical tiered training, physiotherapeutic training/coaching, psychoeducation, neuropsychological and psychotherapeutic counselling and/or optometry treatment at least once a week over a minimum 4-week period performed by professionals with relevant neuroscientific background, for instance physiotherapist, ergotherapist, nurse, rehabilitation specialist, neuropsychologist or doctor. An interdisciplinary treatment should be understood as a treatment with at least two or more interventions and appears as an overall interdisciplinary effort where professionals collaborate on the patient.

Comparison
An effort that allow treatment with one or more professional groups but is not delivered as an interdisciplinary

© 2021 Rytter HM et al. *JAMA Network Open.*

coordinated rehabilitative treatment.

**Outcome measures**	**Time frame**	**Critical/Important**
The collective burden of post-concussion symptoms	*After the end of treatment*	*Critical*
The collective burden of post-concussion symptoms	*Follow-up (3 months after the end of treatment)*	*Important*
Physical functioning	*After the end of treatment*	*Important*
Emotional symptoms	*Follow-up (3 months after the end of treatment)*	*Important*
Behavioral response	*After the end of treatment*	*Important*
Cognitive functioning	*After the end of treatment*	*Important*
Quality of life	*Follow-up (3 months after the end of treatment)*	*Important*
Pain	*After the end of treatment*	*Important*
Negative effect on prognosis	*After the end of treatment*	*Important*
Return to daily activities	*Follow-up (3 months after the end of treatment)*	*Critical*

© 2021 Rytter HM et al. *JAMA Network Open.*

eFigure 1. Flow Charts With Results From the Systematic Review Process for PICO 1-7

PICO 1

Prisma Flowchart systematic reviews.

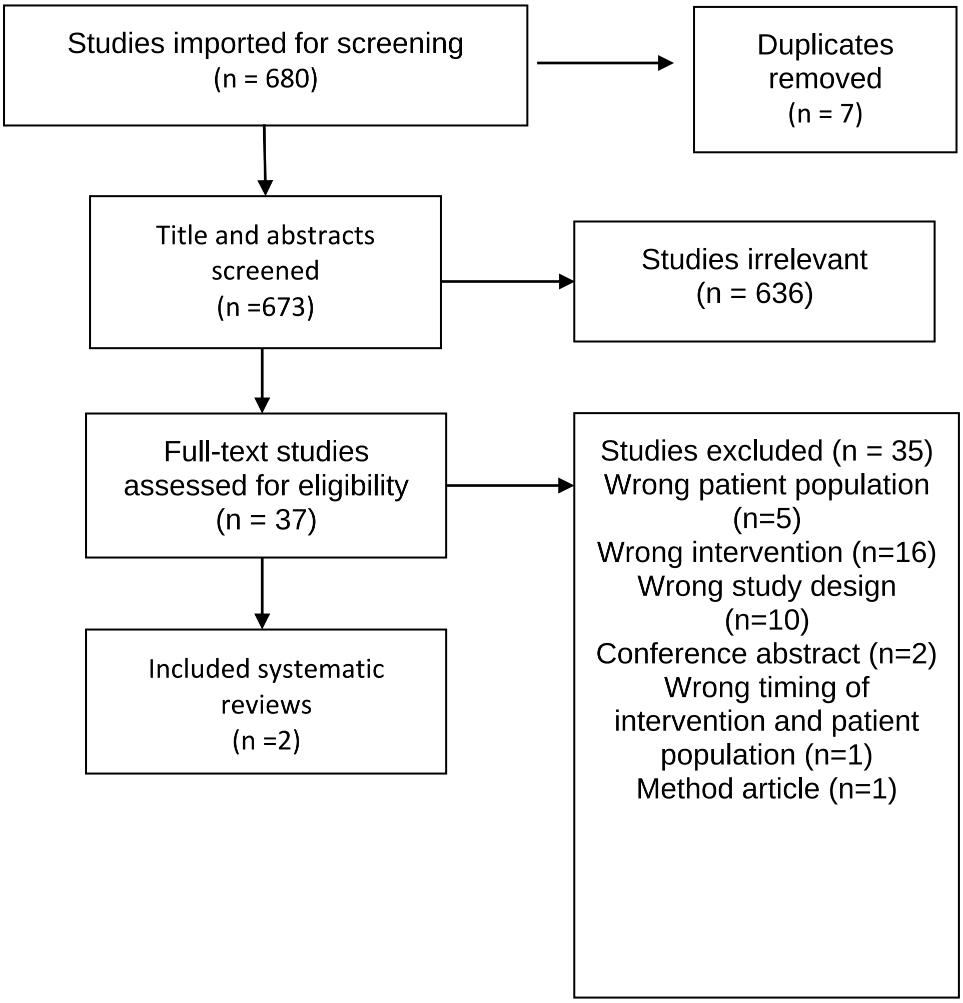

© 2021 Rytter HM et al. *JAMA Network Open.*

Prisma flowchart primary studies.

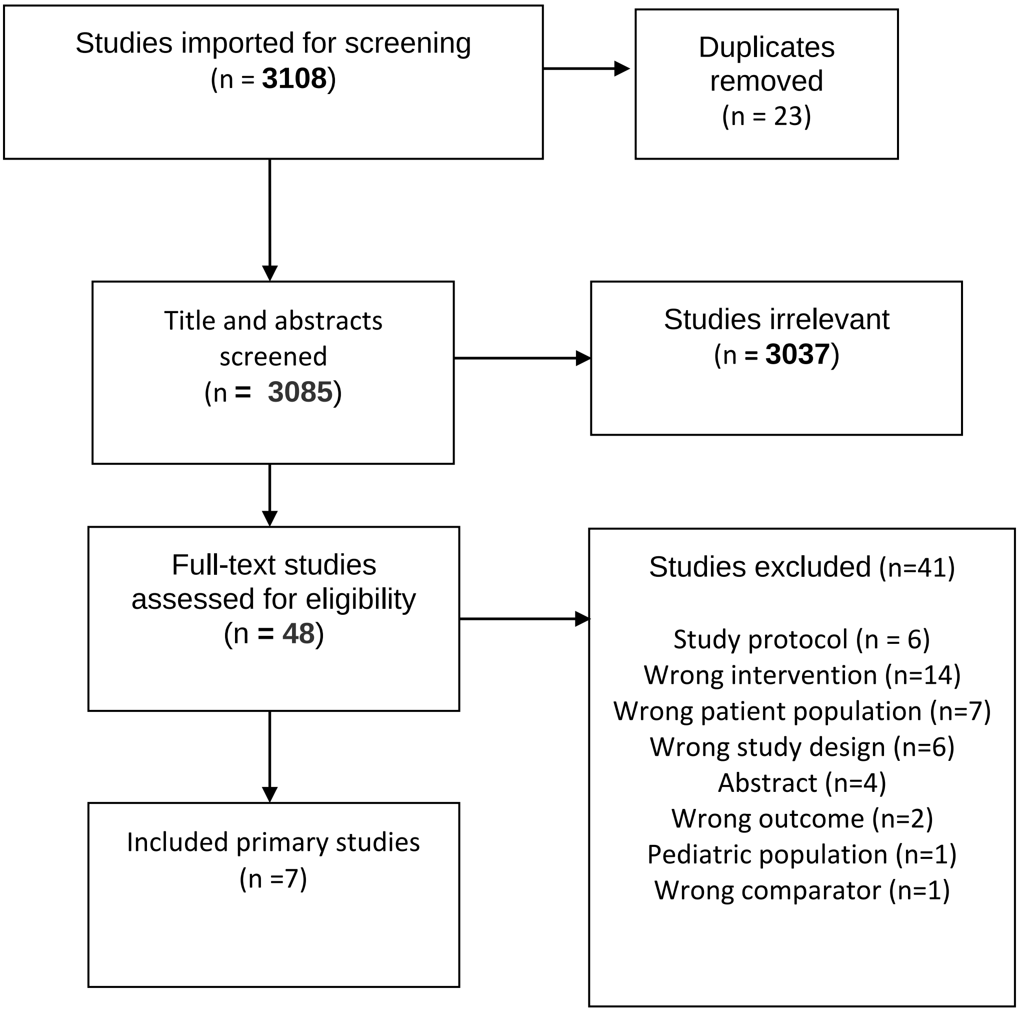

© 2021 Rytter HM et al. *JAMA Network Open.*

PICO 2

Prisma flowchart systematic reviews.

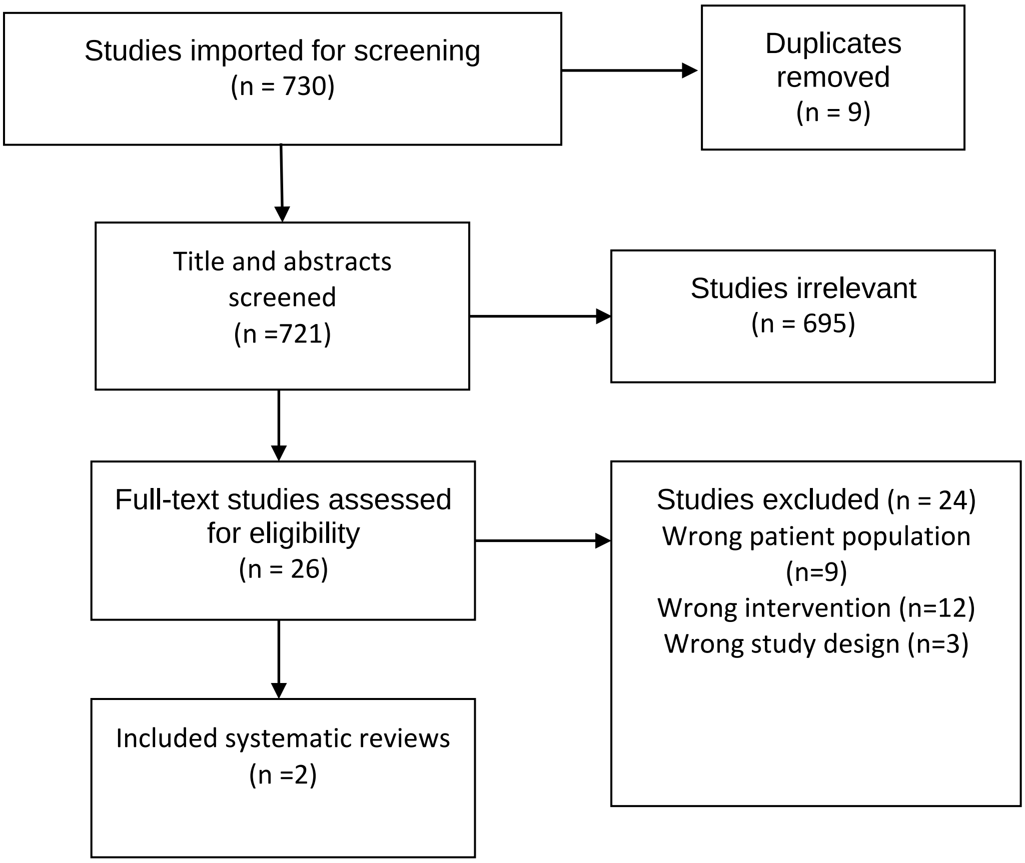

© 2021 Rytter HM et al. *JAMA Network Open.*

Prisma Flowchart primary studies.

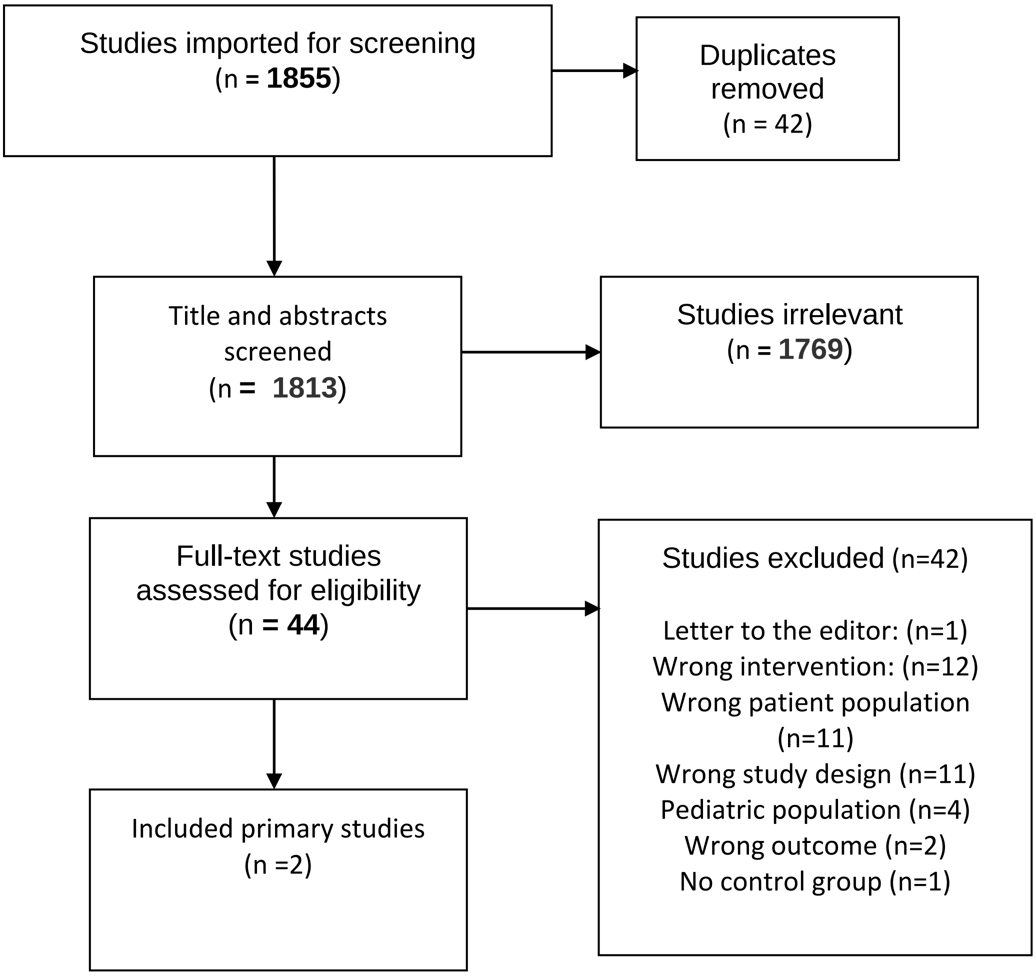

© 2021 Rytter HM et al. *JAMA Network Open.*

PICO 3

Prisma flowchart systematic reviews.

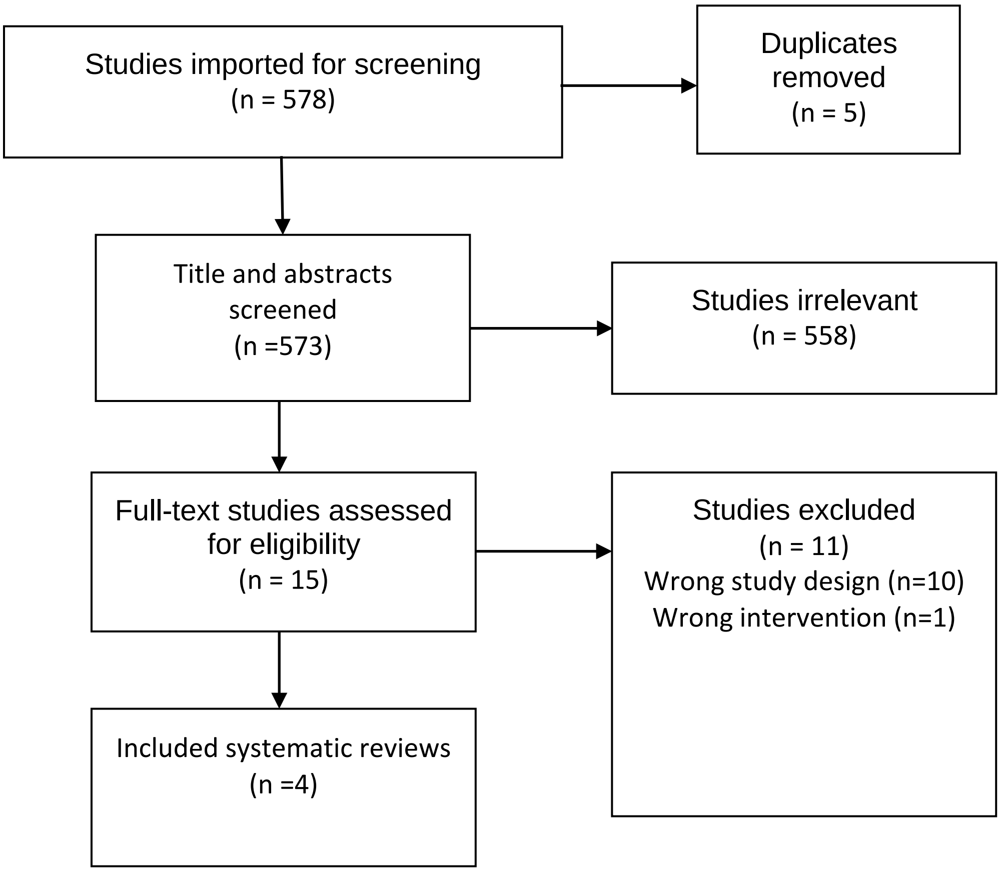

© 2021 Rytter HM et al. *JAMA Network Open.*

Prisma Flowchart primary studies.

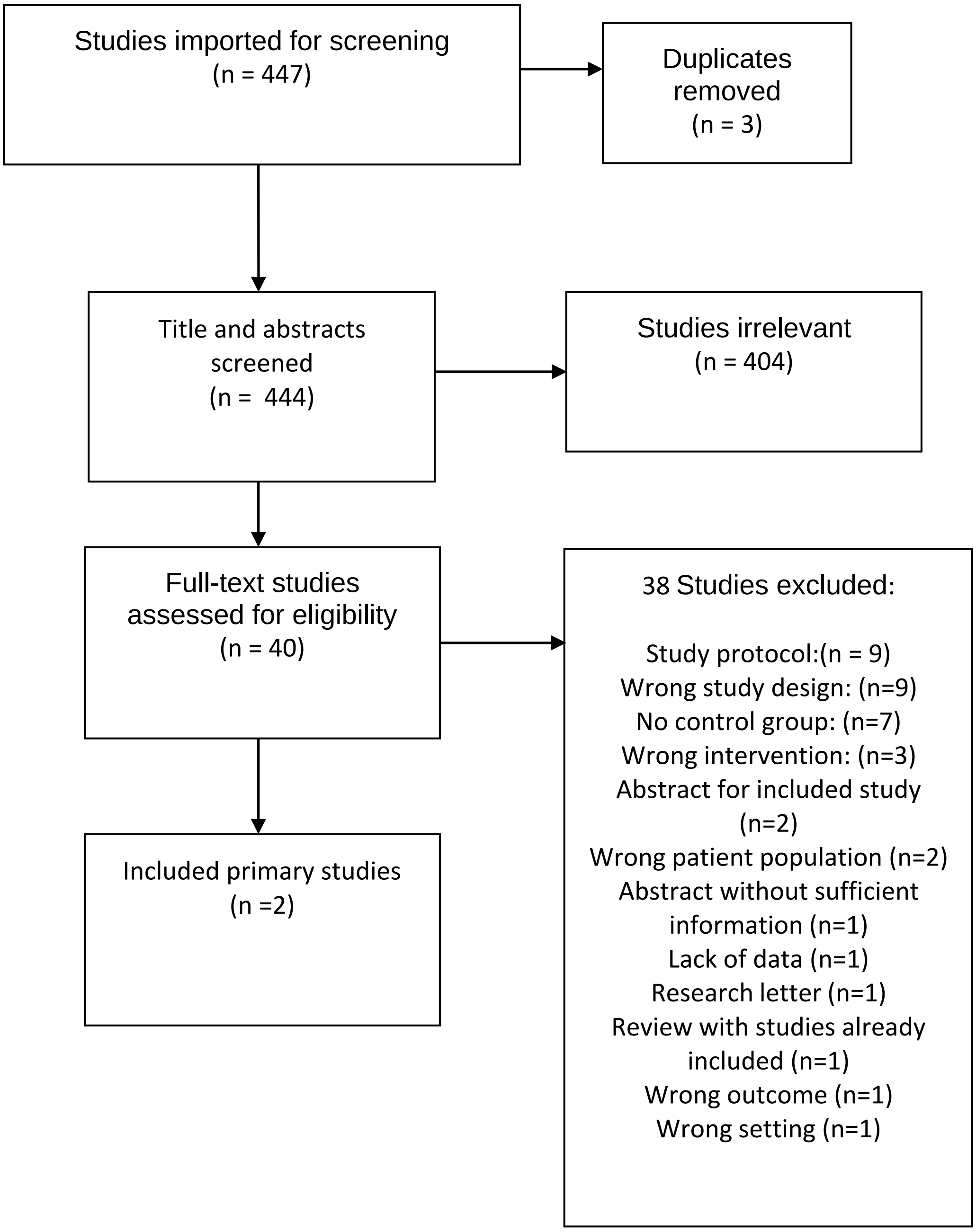

© 2021 Rytter HM et al. *JAMA Network Open.*

PICO 4

Prisma Flowchart systematic reviews.

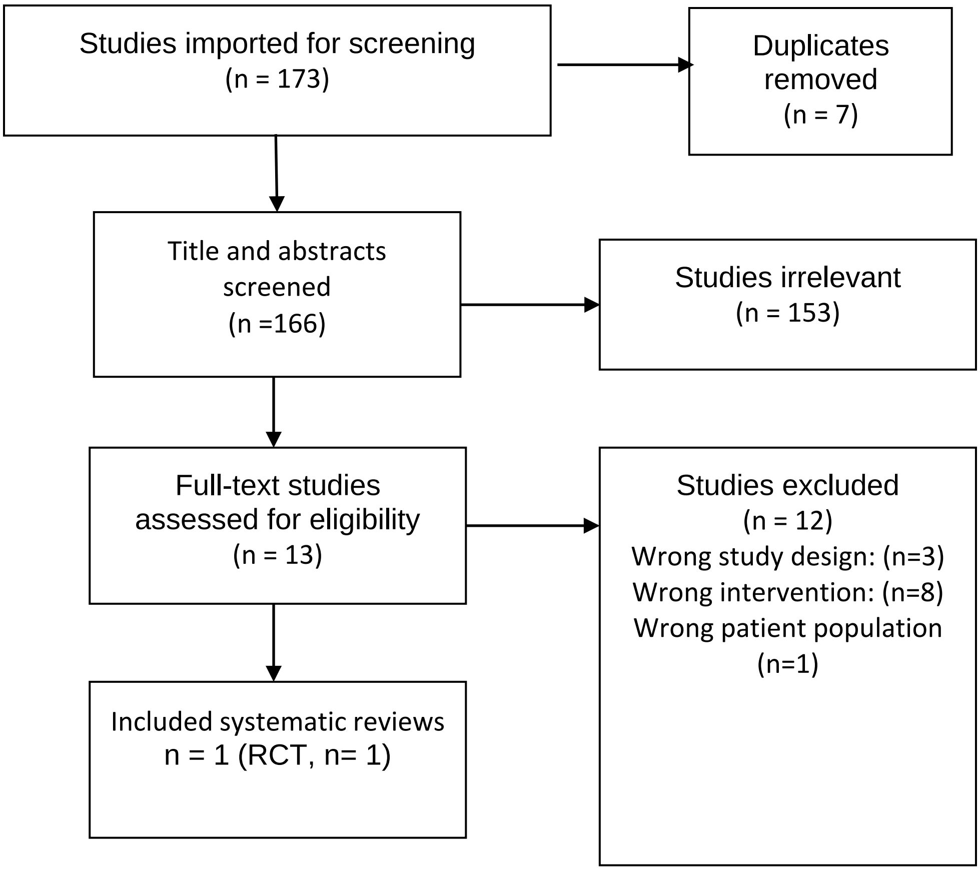

© 2021 Rytter HM et al. *JAMA Network Open.*

Prisma flowchart primary studies.

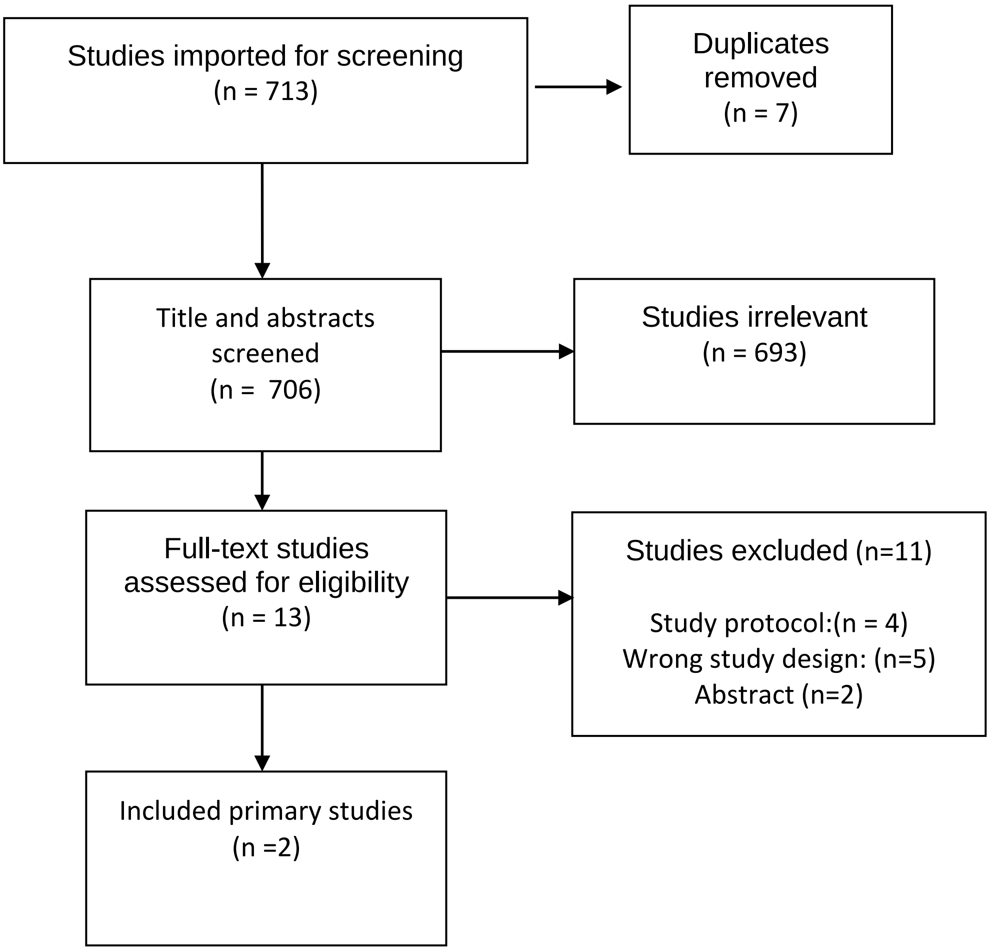

© 2021 Rytter HM et al. *JAMA Network Open.*

PICO 5

Prisma Flowchart systematic reviews.

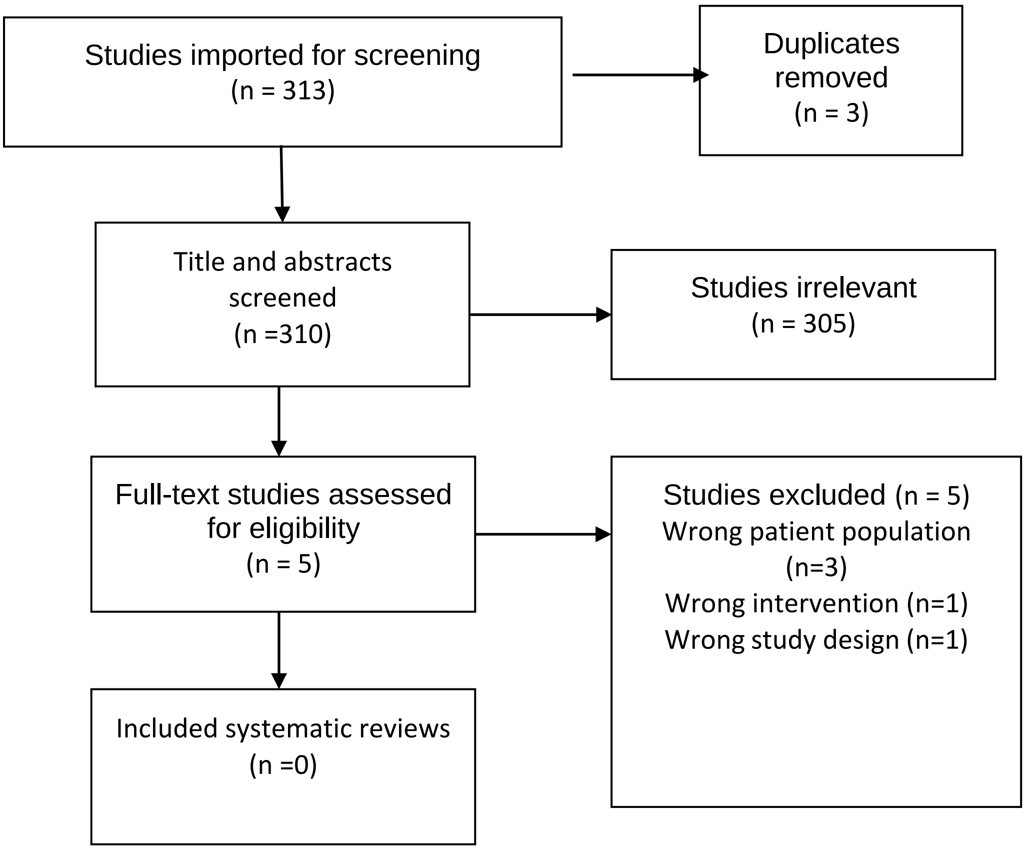

© 2021 Rytter HM et al. *JAMA Network Open.*

Prisma flowchart primary studies.

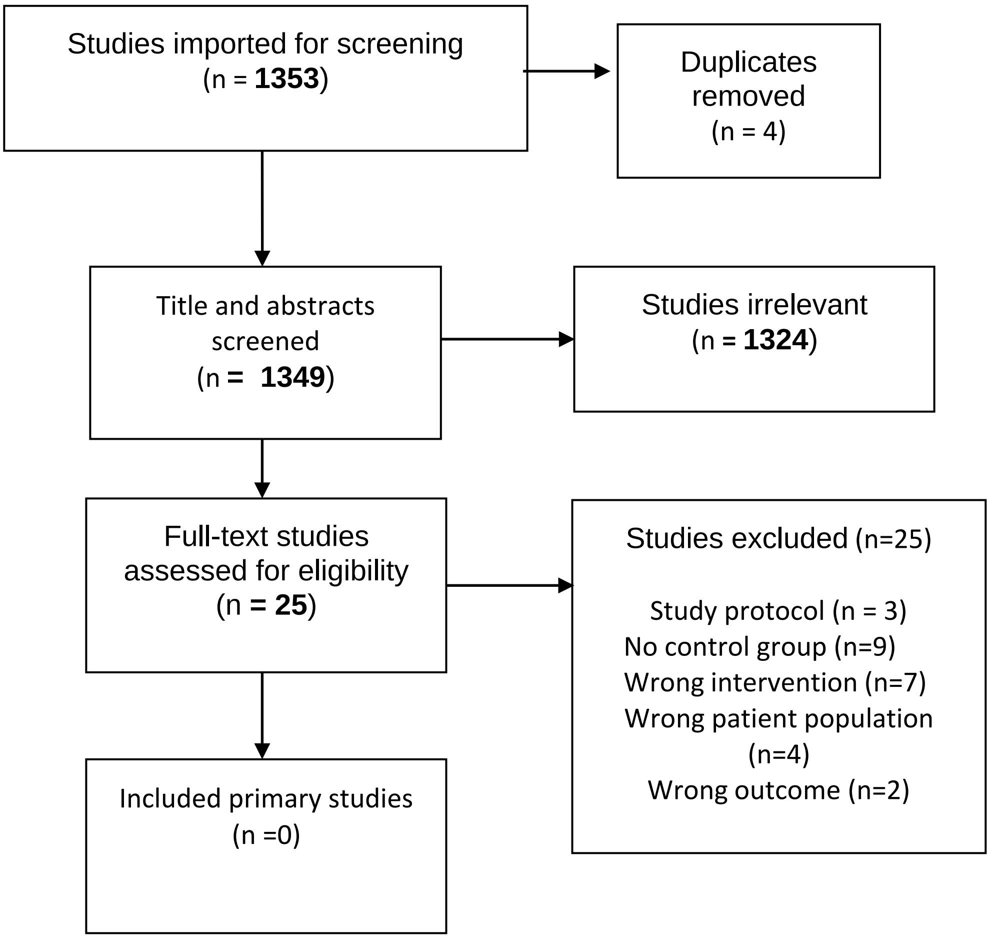

© 2021 Rytter HM et al. *JAMA Network Open.*

PICO 6

Prisma Flowchart systematic reviews.

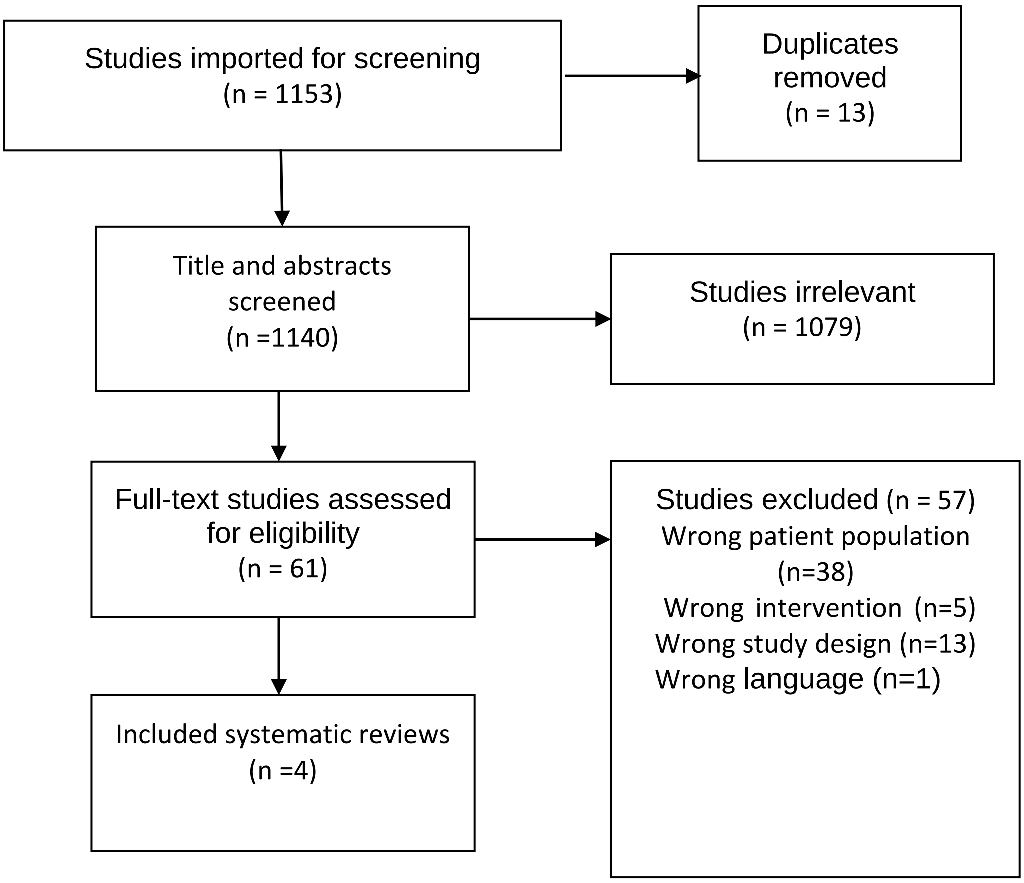

© 2021 Rytter HM et al. *JAMA Network Open.*

Prisma flowchart primary studies.

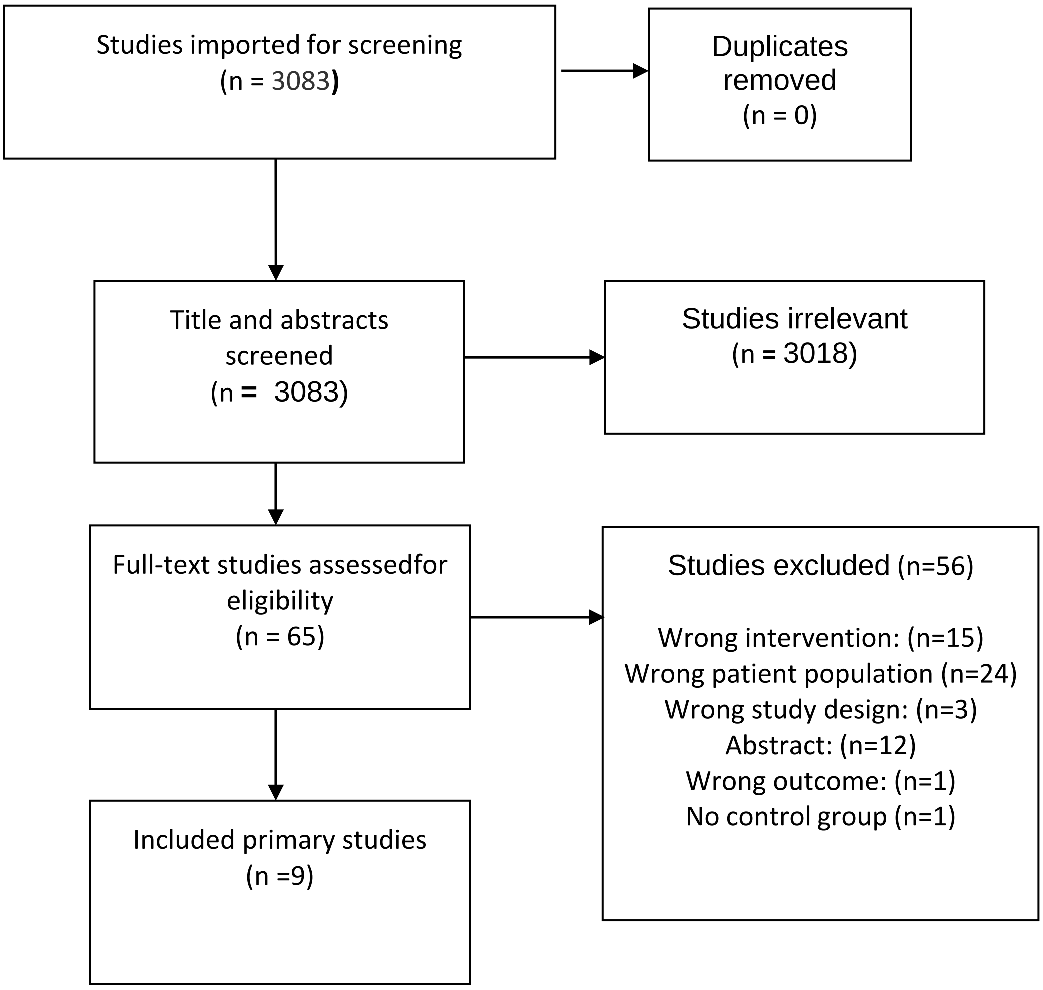

© 2021 Rytter HM et al. *JAMA Network Open.*

PICO 7

Prisma Flowchart systematic reviews.

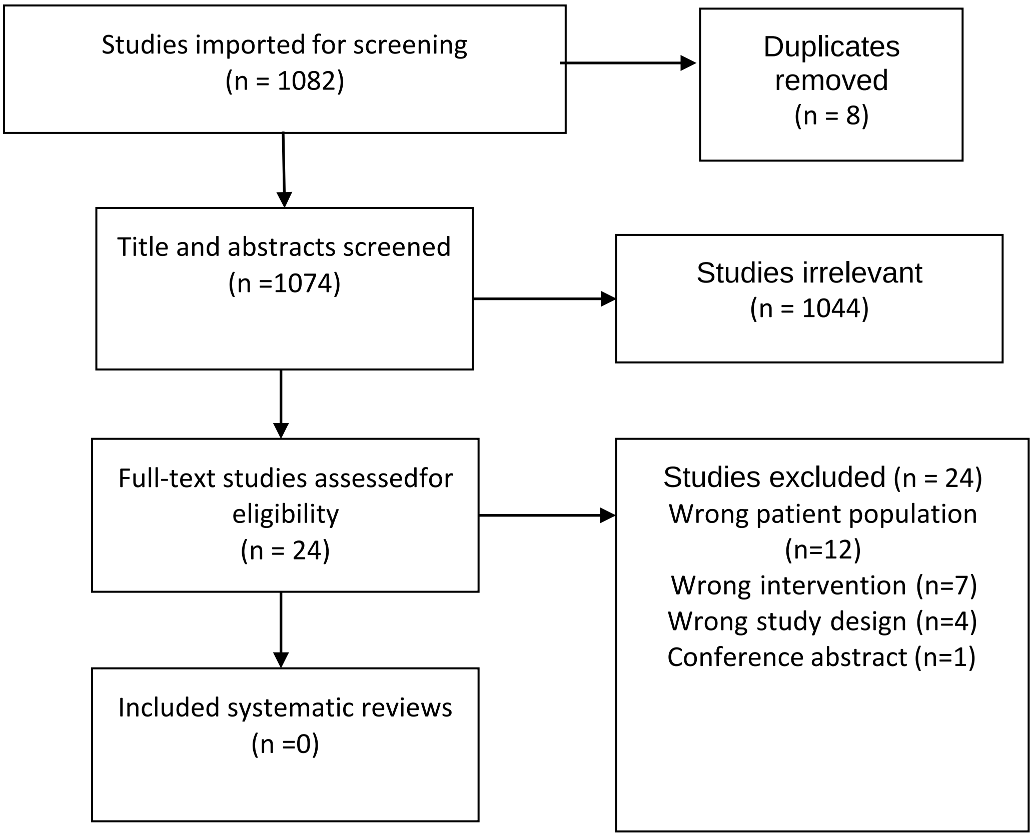

© 2021 Rytter HM et al. *JAMA Network Open.*

Prisma flowchart primary studies.

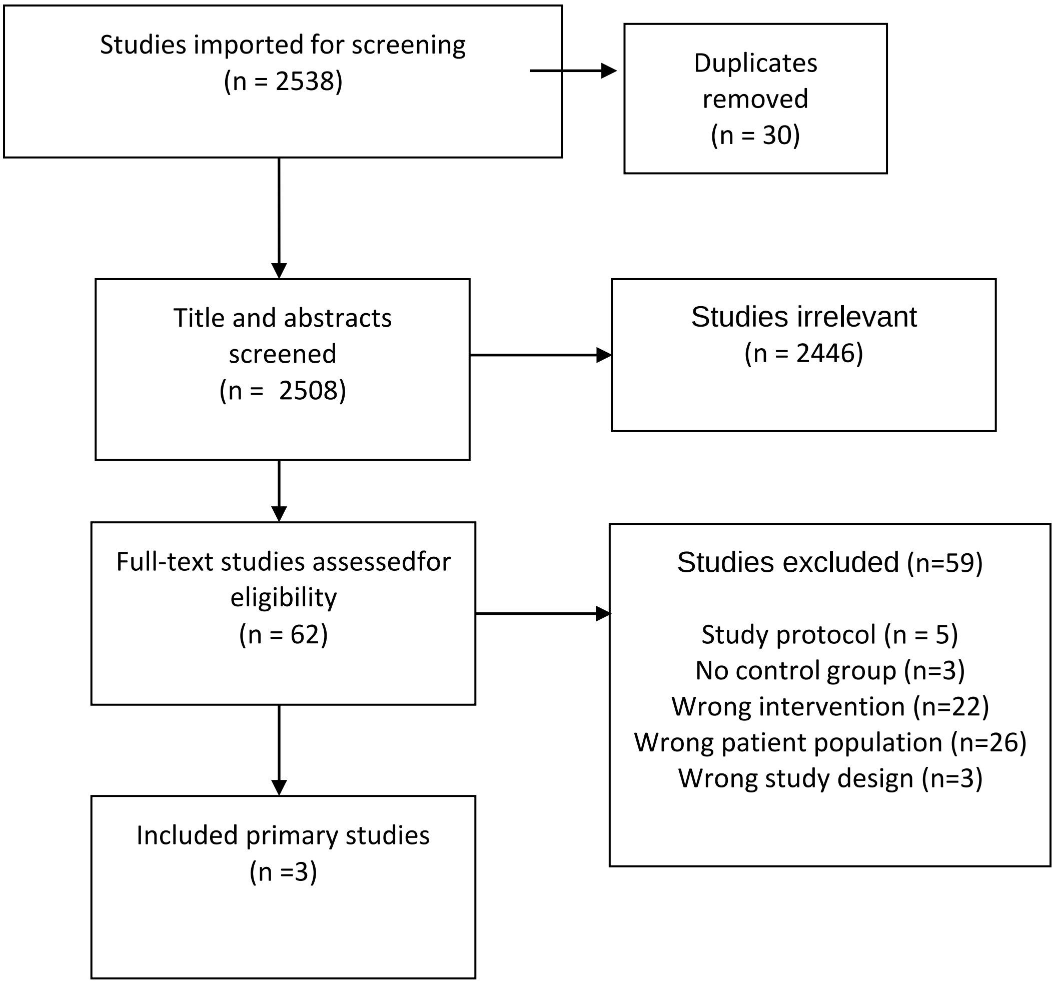

© 2021 Rytter HM et al. *JAMA Network Open.*

eTable 3. Evaluation of Systematic Reviews Based on AMSTAR Tool

Article	Was meta-analysis performed for the relevant outcomes?	1. Was an 'a priori' design provided?	2. Was there duplicate study selection and data extraction?	3. Was a comprehensive literature search performed?	4. Was the status of publication (i.e. grey literature) used as an inclusion criterion?	5. Was a list of studies (included and excluded) provided?	6. Were the characteristics of the included studies provided?	7. Was the scientific quality of the included studies assessed and documented?	8. Was the scientific quality of the included studies used appropriately in formulating conclusions?	9. Were the methods used to combine the findings of studies appropriate?	10. Was the likelihood of publication bias assessed?	11. Was the conflict of interest included?
Eliyahu 2016	no	Yes	Yes	Yes	Yes	No	Yes	Yes	Yes	Not applicable	No	Yes
Gravel 2013	yes	Yes	Yes	Yes	Yes	No	Yes	Yes	Yes	Yes	No	No
Quatman-Yates 2016	No	No	Yes	Yes	Yes	No	Yes	Yes	Yes	Yes	No	No
Lal 2018	yes	Can't answer	Yes	Yes	Yes	Yes	No	Yes	Yes	Yes	Yes	Yes
Kinne 2018	No	No	Yes	Yes	No	No	Yes	Yes	Yes	No	No	No
Murray 2017	No	No	Yes	Yes	Yes	No	Yes	Yes	Yes	No	No	No
Schneider 2017	No	Yes	Yes	Yes	No	No	Yes	Yes	Yes	Yes	Yes	No
Al Sayegh 2010	No	No	No	Yes	No	No	No	Yes	Yes	Not applicable	No	No
Bergersen 2017	No	No	Yes	Yes	No	No	Yes	Yes	Yes	No	No	No
Snell 2009	No	No	Yes	Yes	Yes	No	Yes	Yes	Yes	Can't answer	No	No
Sullivan 2018	No	Yes	Yes	Yes	No	No	Yes	Yes	Yes	Not applicable	Yes	No
Gertler 2015	Yes	Yes	Yes	Yes	Yes	Yes	Yes	Yes	Yes	Yes	No	No

© 2021 Rytter HM et al. *JAMA Network Open.*

eFigure 2. Meta-Analyses for PICO 1, 2, 3, 4, 6, 7 per Specified Outcome Measures

1 Intervention vs Control

1.1 The Collective Burden of Post-concussion Symptoms (Follow-up, 14 days after EoT) (Rivermead)

Study or Subgroup	Intervention Mean	Intervention SD	Intervention Total	Control Mean	Control SD	Control Total	Weight	Mean Difference IV, Fixed, 95% CI
Suffoletto 2013	17	20.74	14	20	15.55	22	100.0%	-3.00 [-15.66, 9.66]
**Total (95% CI)**			**14**			**22**	**100.0%**	**-3.00 [-15.66, 9.66]**

Heterogeneity: Not applicable
Test for overall effect: Z = 0.46 (P = 0.64)

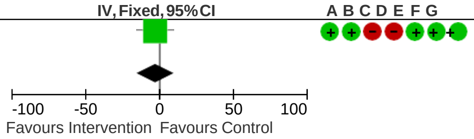

Risk of bias legend
(A) Random sequence generation (selection bias)
(B) Allocation concealment (selection bias)
(C) Blinding of participants and personnel (performance bias)
(D) Blinding of outcome assessment (detection bias)
(E) Incomplete outcome data (attrition bias)
(F) Selective reporting (reporting bias)
(G) Other bias

1.2 The Collective Burden of Post-concussion Symptoms (Follow-up, 3 months after EoT) (Head Injury Symptom Checklist)

Study or Subgroup	Mean Difference	SE	Weight	Mean Difference IV, Fixed, 95% CI
Bell 2008	6.6	2.752	100.0%	6.60 [1.21, 11.99]
**Total (95% CI)**			**100.0%**	**6.60 [1.21, 11.99]**

Heterogeneity: Not applicable
Test for overall effect: Z = 2.40 (P = 0.02)

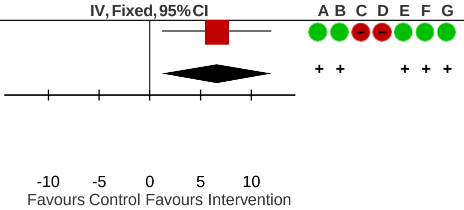

Risk of bias legend
(A) Random sequence generation (selection bias)
(B) Allocation concealment (selection bias)
(C) Blinding of participants and personnel (performance bias)
(D) Blinding of outcome assessment (detection bias)
(E) Incomplete outcome data (attrition bias)
(F) Selective reporting (reporting bias)
(G) Other bias

1.3 The Collective Burden of Post-concussion Symptoms (Followup, 6 months after EoT) (Frequency of Symptoms)

Study or Subgroup	Intervention Mean	Intervention SD	Intervention Total	Control Mean	Control SD	Control Total	Weight	Mean Difference IV, Fixed, 95% CI
Mittenberg 1996	1.62	2.04	29	3.1	3.19	29	100.0%	-1.48 [-2.86, -0.10]
**Total (95% CI)**			**29**			**29**	**100.0%**	**-1.48 [-2.86, -0.10]**

Heterogeneity: Not applicable
Test for overall effect: Z = 2.10 (P = 0.04)

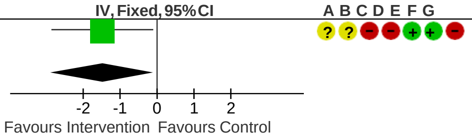

Risk of bias legend
(A) Random sequence generation (selection bias)
(B) Allocation concealment (selection bias)
(C) Blinding of participants and personnel (performance bias)
(D) Blinding of outcome assessment (detection bias)
(E) Incomplete outcome data (attrition bias)
(F) Selective reporting (reporting bias)
(G) Other bias

© 2021 Rytter HM et al. *JAMA Network Open.*

1.4 Number of Patients with Significant Symptom Burden Measured by PCSS>20 (Follow-up)

Study or Subgroup	Intervention Events	Intervention Total	Control Events	Control Total	Weight	Risk Ratio IV, Fixed, 95% CI	Risk of Bias A B C D E F G
Varner 2017	12	36	11	40	100.0%	1.21 [0.61, 2.40]	+ + − − + + +
**Total (95% CI)**		**36**		**40**	**100.0%**	**1.21 [0.61, 2.40]**	
Total events	12		11				

Heterogeneity: Not applicable
Test for overall effect: Z = 0.55 (P = 0.58)

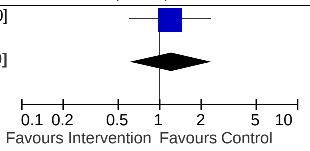

Risk of bias legend
(A) Random sequence generation (selection bias)
(B) Allocation concealment (selection bias)
(C) Blinding of participants and personnel (performance bias)
(D) Blinding of outcome assessment (detection bias)
(E) Incomplete outcome data (attrition bias)
(F) Selective reporting (reporting bias)
(G) Other bias

1.5 Pain, Headache (End of Treatment)

Study or Subgroup	Intervention Mean	Intervention SD	Intervention Total	Control Mean	Control SD	Control Total	Weight	Mean Difference IV, Fixed, 95% CI	Risk of Bias A B C D E F G
Suffoletto 2013	0.99	0.98	18	1.19	0.89	25	100.0%	-0.20 [-0.77, 0.37]	+ + − − + + +
**Total (95% CI)**			**18**			**25**	**100.0%**	**-0.20 [-0.77, 0.37]**	

Heterogeneity: Not applicable
Test for overall effect: Z = 0.69 (P = 0.49)

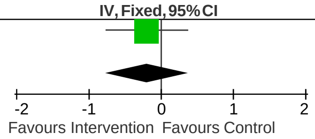

Risk of bias legend
(A) Random sequence generation (selection bias)
(B) Allocation concealment (selection bias)
(C) Blinding of participants and personnel (performance bias)
(D) Blinding of outcome assessment (detection bias)
(E) Incomplete outcome data (attrition bias)
(F) Selective reporting (reporting bias)
(G) Other bias

1.6 Number of Patients with Affected School Attendance (Follow-up, 3 months after EoT)

Study or Subgroup	Intervention Events	Intervention Total	Control Events	Control Total	Weight	Risk Ratio M-H, Fixed, 95% CI	Risk of Bias A B C D E F G
Bell 2008	6	146	12	166	100.0%	0.57 [0.22, 1.48]	+ + − − + + +
**Total (95% CI)**		**146**		**166**	**100.0%**	**0.57 [0.22, 1.48]**	
Total events	6		12				

Heterogeneity: Not applicable
Test for overall effect: Z = 1.16 (P = 0.25)

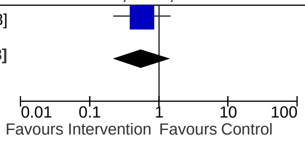

Risk of bias legend
(A) Random sequence generation (selection bias)
(B) Allocation concealment (selection bias)
(C) Blinding of participants and personnel (performance bias)
(D) Blinding of outcome assessment (detection bias)
(E) Incomplete outcome data (attrition bias)
(F) Selective reporting (reporting bias)
(G) Other bias

© 2021 Rytter HM et al. *JAMA Network Open.*

1.7 Number of Patients with Headache (Follow-up, 3 - 6 months after EoT)

Study or Subgroup	Intervention Events	Intervention Total	Control Events	Control Total	Weight	Risk Ratio IV, Random, 95% CI	Risk of Bias A	B	C	D	E	F	G
Bell 2008	43	146	58	166	40.8%	0.84 [0.61, 1.17]	+	+	−	−	+	+	+
Heskestad 2010	9	22	5	28	21.8%	2.29 [0.90, 5.86]	−	−	−	−	−	+	+
Mittenberg 1996	13	29	25	29	37.5%	0.52 [0.34, 0.80]	?	?			+	+	−
**Total (95% CI)**		**197**		**223**	**100.0%**	**0.87 [0.48, 1.60]**							
Total events	65		88										

Heterogeneity: Tau² = 0.20; Chi² = 8.68, df = 2 (P = 0.01); I² = 77%
Test for overall effect: Z = 0.44 (P = 0.66)

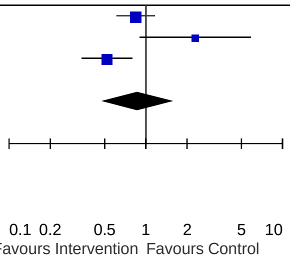

Risk of bias legend
(A) Random sequence generation (selection bias)
(B) Allocation concealment (selection bias)
(C) Blinding of participants and personnel (performance bias)
(D) Blinding of outcome assessment (detection bias)
(E) Incomplete outcome data (attrition bias)
(F) Selective reporting (reporting bias)
(G) Other bias

1.8 Emotional Symptoms, Depression (BDI and HADS / Depression Scale) (Follow-up, 3 months after EoT)

Study or Subgroup	Intervention Mean	Intervention SD	Intervention Total	Control Mean	Control SD	Control Total	Weight	Std. Mean Difference IV, Random, 95% CI	Risk of Bias A	B	C	D	E	F	G
Heskestad 2010	7.2	5.98	22	7.8	7.82	28	33.8%	-0.08 [-0.64, 0.48]	−	−	−	−	−	+	+
Matuseviciene 2013	3.4	0.58	48	3.5	0.5	49	66.2%	-0.18 [-0.58, 0.22]	?	?			?	+	−
**Total (95% CI)**			**70**			**77**	**100.0%**	**-0.15 [-0.47, 0.18]**							

Heterogeneity: Tau² = 0.00; Chi² = 0.08, df = 1 (P = 0.78); I² = 0%
Test for overall effect: Z = 0.90 (P = 0.37)

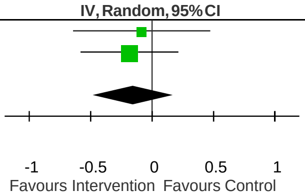

Risk of bias legend
(A) Random sequence generation (selection bias)
(B) Allocation concealment (selection bias)
(C) Blinding of participants and personnel (performance bias)
(D) Blinding of outcome assessment (detection bias)
(E) Incomplete outcome data (attrition bias)
(F) Selective reporting (reporting bias)
(G) Other bias

1.9 Number of Patients with Depression (Follow-up, 14 days - 6 months after EoT)

Study or Subgroup	Intervention Events	Intervention Total	Control Events	Control Total	Weight	Risk Ratio M-H, Random, 95% CI	Risk of Bias A	B	C	D	E	F	G
Mittenberg 1996	8	29	16	29	53.9%	0.50 [0.25, 0.98]	?	?	−	−	+	+	−
Suffoletto 2013	6	14	3	22	46.1%	3.14 [0.93, 10.57]	+	+			+	+	+
**Total (95% CI)**		**43**		**51**	**100.0%**	**1.17 [0.19, 7.10]**							
Total events	14		19										

Heterogeneity: Tau² = 1.46; Chi² = 6.81, df = 1 (P = 0.009); I² = 85%
Test for overall effect: Z = 0.17 (P = 0.87)

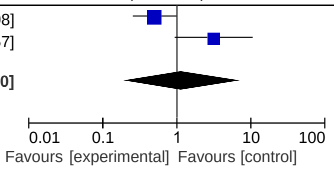

Risk of bias legend
(A) Random sequence generation (selection bias)
(B) Allocation concealment (selection bias)
(C) Blinding of participants and personnel (performance bias)
(D) Blinding of outcome assessment (detection bias)
(E) Incomplete outcome data (attrition bias)
(F) Selective reporting (reporting bias)
(G) Other bias

© 2021 Rytter HM et al. *JAMA Network Open.*

1.10 Number of Patients with Anxiety (Follow-up, 14 days - 6 months after EoT)

Study or Subgroup	Intervention Events	Intervention Total	Control Events	Control Total	Weight	Risk Ratio IV, Random, 95% CI
Mittenberg 1996	11	29	16	29	28.2%	0.69 [0.39, 1.22]
Suffoletto 2013	6	14	10	22	15.9%	0.94 [0.44, 2.01]
Bell 2008	35	146	37	166	55.8%	1.08 [0.72, 1.61]
**Total (95% CI)**		**189**		**217**	**100.0%**	**0.93 [0.69, 1.26]**
Total events	52		63			

Heterogeneity: Tau² = 0.00; Chi² = 1.58, df = 2 (P = 0.45); I² = 0%
Test for overall effect: Z = 0.48 (P = 0.63)

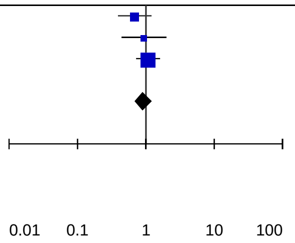

Risk of bias legend
(A) Random sequence generation (selection bias)
(B) Allocation concealment (selection bias)
(C) Blinding of participants and personnel (performance bias)
(D) Blinding of outcome assessment (detection bias)
(E) Incomplete outcome data (attrition bias)
(F) Selective reporting (reporting bias)
(G) Other bias

1.11 Average Number of Days at Work (Follow-up, 4 weeks after EoT)

Study or Subgroup	Intervention Mean	Intervention SD	Intervention Total	Control Mean	Control SD	Control Total	Weight	Mean Difference IV, Fixed, 95% CI
Varner 2017	5	2.96	46	4	5.9	44	100.0%	1.00 [-0.94, 2.94]
**Total (95% CI)**			**46**			**44**	**100.0%**	**1.00 [-0.94, 2.94]**

Heterogeneity: Not applicable
Test for overall effect: Z = 1.01 (P = 0.31)

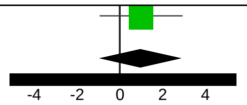

Risk of bias legend
(A) Random sequence generation (selection bias)
(B) Allocation concealment (selection bias)
(C) Blinding of participants and personnel (performance bias)
(D) Blinding of outcome assessment (detection bias)
(E) Incomplete outcome data (attrition bias)
(F) Selective reporting (reporting bias)
(G) Other bias

1.12 Number of Patients with Affected Work Functioning (Follow-up, 3 months after EoT)

Study or Subgroup	Intervention Events	Intervention Total	Control Events	Control Total	Weight	Risk Ratio IV, Fixed, 95% CI
Bell 2008	27	146	49	166	100.0%	0.63 [0.41, 0.95]
**Total (95% CI)**		**146**		**166**	**100.0%**	**0.63 [0.41, 0.95]**
Total events	27		49			

Heterogeneity: Not applicable
Test for overall effect: Z = 2.21 (P = 0.03)

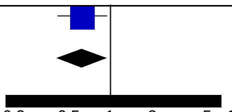

Risk of bias legend
(A) Random sequence generation (selection bias)
(B) Allocation concealment (selection bias)
(C) Blinding of participants and personnel (performance bias)
(D) Blinding of outcome assessment (detection bias)
(E) Incomplete outcome data (attrition bias)
(F) Selective reporting (reporting bias)
(G) Other bias

© 2021 Rytter HM et al. *JAMA Network Open.*

1.13 Number of Patients with Poor Walking Function (Follow-up, 3 months after EoT)

Study or Subgroup	Intervention Events	Intervention Total	Control Events	Control Total	Weight	Risk Ratio IV, Fixed, 95% CI	Risk of Bias A	B	C	D	E	F	G
Bell 2008	9	146	12	166	100.0%	0.85 [0.37, 1.97]	+	+	−	−	+	+	+
**Total (95% CI)**		**146**		**166**	**100.0%**	**0.85 [0.37, 1.97]**							
Total events	9		12										

Heterogeneity: Not applicable
Test for overall effect: Z = 0.37 (P = 0.71)

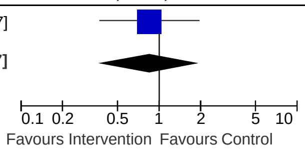

Risk of bias legend
(A) Random sequence generation (selection bias)
(B) Allocation concealment (selection bias)
(C) Blinding of participants and personnel (performance bias)
(D) Blinding of outcome assessment (detection bias)
(E) Incomplete outcome data (attrition bias)
(F) Selective reporting (reporting bias)
(G) Other bias

1.14 Number of Patients with Memory and Concentration Problems (Follow-up, 3 - 6 months after EoT)

Study or Subgroup	Intervention Events	Intervention Total	Control Events	Control Total	Weight	Risk Ratio IV, Random, 95% CI	Risk of Bias A	B	C	D	E	F	G
Bell 2008	26	146	50	166	58.9%	0.59 [0.39, 0.90]	+	+	−	−	+	+	+
Mittenberg 1996	11	29	23	29	41.1%	0.48 [0.29, 0.79]	?	?			+	+	−
**Total (95% CI)**		**175**		**195**	**100.0%**	**0.54 [0.39, 0.75]**							
Total events	37		73										

Heterogeneity: Tau² = 0.00; Chi² = 0.41, df = 1 (P = 0.52); I² = 0%
Test for overall effect: Z = 3.74 (P = 0.0002)

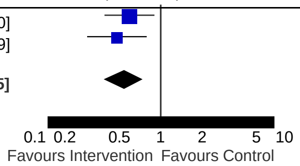

Risk of bias legend
(A) Random sequence generation (selection bias)
(B) Allocation concealment (selection bias)
(C) Blinding of participants and personnel (performance bias)
(D) Blinding of outcome assessment (detection bias)
(E) Incomplete outcome data (attrition bias)
(F) Selective reporting (reporting bias)
(G) Other bias

1.15 Number of Patients with Affected Leisure Time (Follow-up, 3 months after EoT)

Study or Subgroup	Intervention Events	Intervention Total	Control Events	Control Total	Weight	Risk Ratio IV, Fixed, 95% CI	Risk of Bias A	B	C	D	E	F	G
Bell 2008	20	146	39	166	100.0%	0.58 [0.36, 0.95]	+	+	−	−	+	+	+
**Total (95% CI)**		**146**		**166**	**100.0%**	**0.58 [0.36, 0.95]**							
Total events	20		39										

Heterogeneity: Not applicable
Test for overall effect: Z = 2.15 (P = 0.03)

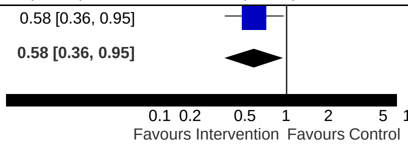

Risk of bias legend
(A) Random sequence generation (selection bias)
(B) Allocation concealment (selection bias)
(C) Blinding of participants and personnel (performance bias)
(D) Blinding of outcome assessment (detection bias)
(E) Incomplete outcome data (attrition bias)
(F) Selective reporting (reporting bias)
(G) Other bias

© 2021 Rytter HM et al. *JAMA Network Open.*

1.16 Number of Patients Showing Irritability (Follow-up, 3 - 6 months after EoT)

Study or Subgroup	Intervention Events	Intervention Total	Control Events	Control Total	Weight	Risk Ratio IV, Random, 95% CI
Bell 2008	34	146	45	166	40.4%	0.86 [0.58, 1.26]
Heskestad 2010	4	22	5	28	4.2%	1.02 [0.31, 3.35]
Mittenberg 1996	18	29	24	29	55.4%	0.75 [0.54, 1.04]
**Total (95% CI)**		**197**		**223**	**100.0%**	**0.80 [0.63, 1.03]**
Total events	56		74			

Heterogeneity: $Tau^2 = 0.00$; $Chi^2 = 0.44$, df = 2 (P = 0.80); $I^2 = 0\%$
Test for overall effect: Z = 1.76 (P = 0.08)

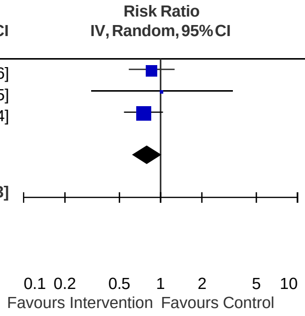

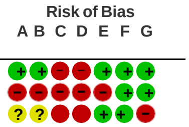

Risk of bias legend
(A) Random sequence generation (selection bias)
(B) Allocation concealment (selection bias)
(C) Blinding of participants and personnel (performance bias)
(D) Blinding of outcome assessment (detection bias)
(E) Incomplete outcome data (attrition bias)
(F) Selective reporting (reporting bias)
(G) Other bias

© 2021 Rytter HM et al. *JAMA Network Open.*

Figures

1.1 The Collective Burden of Postconcussion Symptoms (End of Treatment)

Study or Subgroup	Intervention Mean	Intervention SD	Intervention Total	Control Mean	Control SD	Control Total	Weight	Mean Difference IV, Random, 95% CI
Rytter 2019	32.29	14.18	45	37.5	8.48	44	46.5%	-5.21 [-10.05, -0.37]
Thastum 2019	23.5	13.25	57	27.9	11.07	55	53.5%	-4.40 [-8.92, 0.12]
**Total (95% CI)**			**102**			**99**	**100.0%**	**-4.78 [-8.08, -1.47]**

Heterogeneity: Tau² = 0.00; Chi² = 0.06, df = 1 (P = 0.81); I² = 0%
Test for overall effect: Z = 2.84 (P = 0.005)

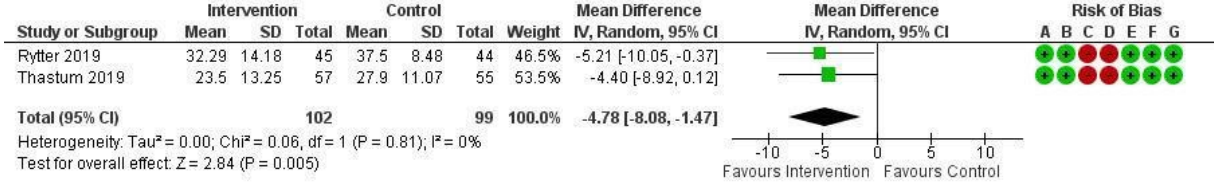

Risk of bias legend
(A) Random sequence generation (selection bias)
(B) Allocation concealment (selection bias)
(C) Blinding of participants and personnel (performance bias)
(D) Blinding of outcome assessment (detection bias)
(E) Incomplete outcome data (attrition bias)
(F) Selective reporting (reporting bias)
(G) Other bias

1.2 The Collective Burden of Postconcussion Symptoms (Follow-up, 3 - 6 months after EoT)

Study or Subgroup	Intervention Mean	Intervention SD	Intervention Total	Control Mean	Control SD	Control Total	Weight	Mean Difference IV, Random, 95% CI
Rytter 2019	29.69	12.92	45	35.3	7.57	44	61.9%	-5.61 [-10.00, -1.22]
Thastum 2019	-17.4	17.41	57	-9.9	12.48	55	38.1%	-7.50 [-13.10, -1.90]
**Total (95% CI)**			**102**			**99**	**100.0%**	**-6.33 [-9.78, -2.88]**

Heterogeneity: Tau² = 0.00; Chi² = 0.27, df = 1 (P = 0.60); I² = 0%
Test for overall effect: Z = 3.59 (P = 0.0003)

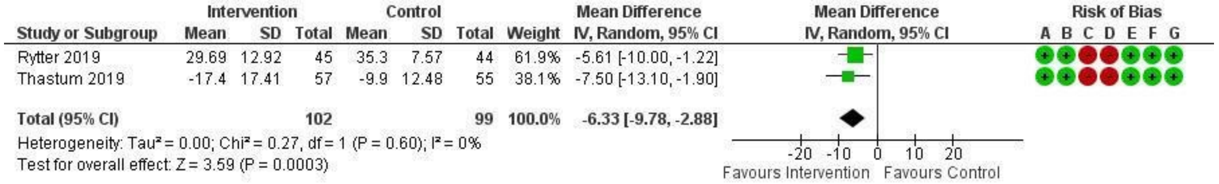

Risk of bias legend
(A) Random sequence generation (selection bias)
(B) Allocation concealment (selection bias)
(C) Blinding of participants and personnel (performance bias)
(D) Blinding of outcome assessment (detection bias)
(E) Incomplete outcome data (attrition bias)
(F) Selective reporting (reporting bias)
(G) Other bias

1.3 Physical Functioning (End of Treatment)

Study or Subgroup	Intervention Mean	Intervention SD	Intervention Total	Control Mean	Control SD	Control Total	Weight	Std. Mean Difference IV, Random, 95% CI
Rytter 2019	75.11	18.93	45	66.14	19.07	44	43.7%	0.47 [0.05, 0.89]
Thastum 2019	-38.8	45.2257	57	-45	10.3574	55	56.3%	0.19 [-0.19, 0.56]
**Total (95% CI)**			**102**			**99**	**100.0%**	**0.31 [0.03, 0.59]**

Heterogeneity: Tau² = 0.00; Chi² = 0.97, df = 1 (P = 0.33); I² = 0%
Test for overall effect: Z = 2.18 (P = 0.03)

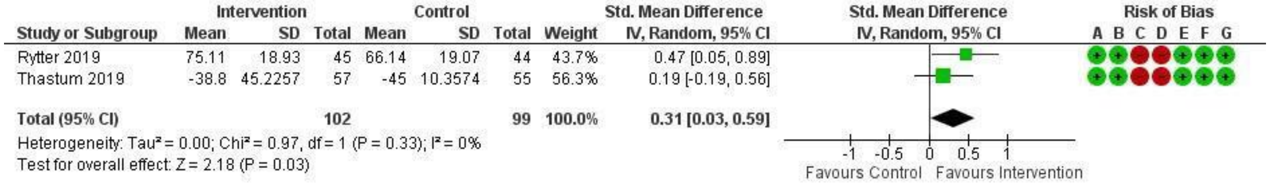

Risk of bias legend
(A) Random sequence generation (selection bias)
(B) Allocation concealment (selection bias)
(C) Blinding of participants and personnel (performance bias)
(D) Blinding of outcome assessment (detection bias)
(E) Incomplete outcome data (attrition bias)
(F) Selective reporting (reporting bias)
(G) Other bias

1.4 Physical Functioning (Follow-up, 3 - 6 months after EoT)

Study or Subgroup	Intervention Mean	Intervention SD	Intervention Total	Control Mean	Control SD	Control Total	Weight	Std. Mean Difference IV, Random, 95% CI
Rytter 2019	76.33	20.15	45	67.96	20.15	44	44.7%	0.41 [-0.01, 0.83]
Thastum 2019	9.3	7.9	57	4.7	8.5	55	55.3%	0.56 [0.18, 0.93]
**Total (95% CI)**			**102**			**99**	**100.0%**	**0.49 [0.21, 0.77]**

Heterogeneity: Tau² = 0.00; Chi² = 0.25, df = 1 (P = 0.61); I² = 0%
Test for overall effect: Z = 3.43 (P = 0.0006)

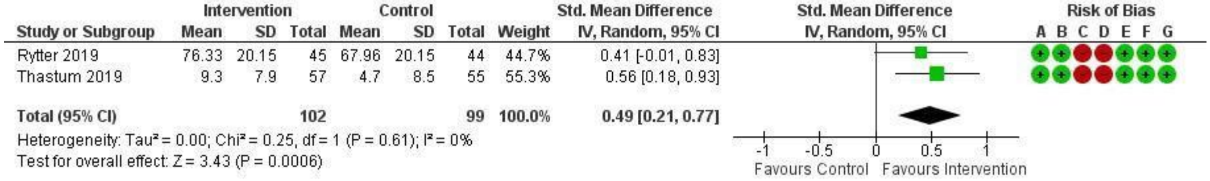

Risk of bias legend
(A) Random sequence generation (selection bias)
(B) Allocation concealment (selection bias)
(C) Blinding of participants and personnel (performance bias)
(D) Blinding of outcome assessment (detection bias)
(E) Incomplete outcome data (attrition bias)
(F) Selective reporting (reporting bias)
(G) Other bias

© 2021 Rytter HM et al. *JAMA Network Open.*

1.5 Emotional Symptoms (Follow-up, 3 - 6 months after EoT)

	Intervention			Control				Std. Mean Difference
Study or Subgroup	Mean	SD	Total	Mean	SD	Total	Weight	IV, Random, 95% CI
Rytter 2019	13.07	9.27	45	18.52	10.29	44	43.6%	-0.55 [-0.98, -0.13]
Thastum 2019	-0.7	1.13	57	-0.4	1.03	55	56.4%	-0.28 [-0.65, 0.10]
**Total (95% CI)**			**102**			**99**	**100.0%**	**-0.40 [-0.68, -0.12]**

Heterogeneity: Tau² = 0.00; Chi² = 0.92, df = 1 (P = 0.34); I² = 0%
Test for overall effect: Z = 2.77 (P = 0.006)

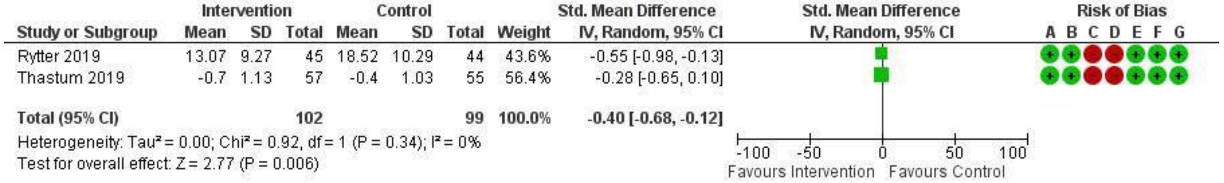

Risk of bias legend
(A) Random sequence generation (selection bias)
(B) Allocation concealment (selection bias)
(C) Blinding of participants and personnel (performance bias)
(D) Blinding of outcome assessment (detection bias)
(E) Incomplete outcome data (attrition bias)
(F) Selective reporting (reporting bias)
(G) Other bias

1.6 Behavioral Responses (Follow-up, 3 months after EoT)

	Intervention			Control				Mean Difference
Study or Subgroup	Mean	SD	Total	Mean	SD	Total	Weight	IV, Fixed, 95% CI
Thastum 2019	-31.4	24.54	57	-19.9	21.08	55	100.0%	-11.50 [-19.96, -3.04]
**Total (95% CI)**			**57**			**55**	**100.0%**	**-11.50 [-19.96, -3.04]**

Heterogeneity: Not applicable
Test for overall effect: Z = 2.66 (P = 0.008)

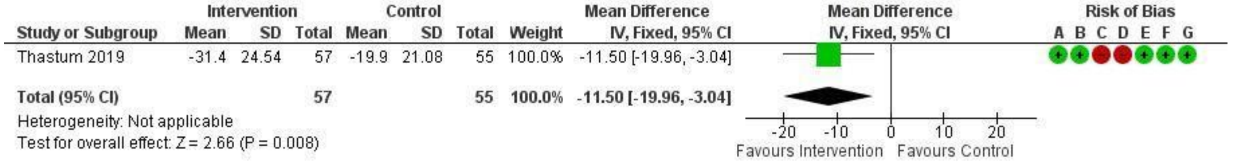

Risk of bias legend
(A) Random sequence generation (selection bias)
(B) Allocation concealment (selection bias)
(C) Blinding of participants and personnel (performance bias)
(D) Blinding of outcome assessment (detection bias)
(E) Incomplete outcome data (attrition bias)
(F) Selective reporting (reporting bias)
(G) Other bias

1.7 Quality of Life (Follow-up, 3 - 6 months after EoT)

	Intervention			Control				Std. Mean Difference
Study or Subgroup	Mean	SD	Total	Mean	SD	Total	Weight	IV, Random, 95% CI
Rytter 2019	3.33	1.19	45	2.52	1.02	44	47.3%	0.72 [0.29, 1.15]
Thastum 2019	23.5	27.15	57	16.8	25.16	55	52.7%	0.25 [-0.12, 0.63]
**Total (95% CI)**			**102**			**99**	**100.0%**	**0.48 [0.02, 0.94]**

Heterogeneity: Tau² = 0.07; Chi² = 2.63, df = 1 (P = 0.11); I² = 62%
Test for overall effect: Z = 2.03 (P = 0.04)

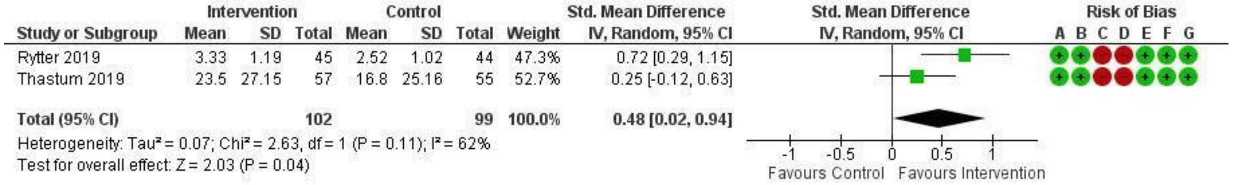

Risk of bias legend
(A) Random sequence generation (selection bias)
(B) Allocation concealment (selection bias)
(C) Blinding of participants and personnel (performance bias)
(D) Blinding of outcome assessment (detection bias)
(E) Incomplete outcome data (attrition bias)
(F) Selective reporting (reporting bias)
(G) Other bias

1.8 Pain, Headache (End of Treatment)

	Intervention			Control				Mean Difference
Study or Subgroup	Mean	SD	Total	Mean	SD	Total	Weight	IV, Fixed, 95% CI
Rytter 2019	2.35	4.5	45	0.93	3.47	44	100.0%	1.42 [-0.25, 3.09]
**Total (95% CI)**			**45**			**44**	**100.0%**	**1.42 [-0.25, 3.09]**

Heterogeneity: Not applicable
Test for overall effect: Z = 1.67 (P = 0.10)

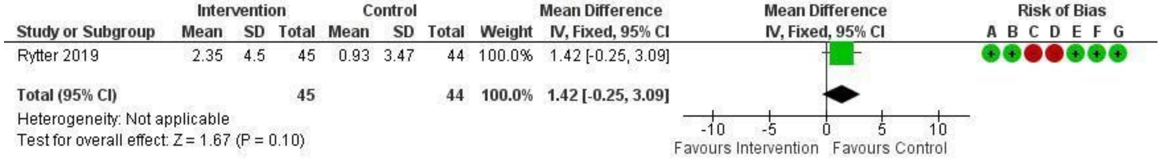

Risk of bias legend
(A) Random sequence generation (selection bias)
(B) Allocation concealment (selection bias)
(C) Blinding of participants and personnel (performance bias)
(D) Blinding of outcome assessment (detection bias)
(E) Incomplete outcome data (attrition bias)
(F) Selective reporting (reporting bias)
(G) Other bias

© 2021 Rytter HM et al. *JAMA Network Open.*

1.9 General Satisfaction with Current Working Conditions (Follow-up, 6 months after EoT)

	Intervention			Control				Mean Difference	Risk of Bias
Study or Subgroup	Mean	SD	Total	Mean	SD	Total	Weight	IV, Fixed, 95% CI	A B C D E F G
Rytter 2019	2.47	1.4	45	1.84	1.1	44	100.0%	0.63 [0.11, 1.15]	+ + − − + + +
**Total (95% CI)**			**45**			**44**	**100.0%**	**0.63 [0.11, 1.15]**	

Heterogeneity: Not applicable
Test for overall effect: Z = 2.36 (P = 0.02)

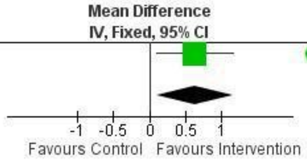

Risk of bias legend
(A) Random sequence generation (selection bias)
(B) Allocation concealment (selection bias)
(C) Blinding of participants and personnel (performance bias)
(D) Blinding of outcome assessment (detection bias)
(E) Incomplete outcome data (attrition bias)
(F) Selective reporting (reporting bias)
(G) Other bias

© 2021 Rytter HM et al. *JAMA Network Open.*

Figures

1.1 Dizziness (End of Treatment)

Study or Subgroup	Intervention Mean	Intervention SD	Intervention Total	Control Mean	Control SD	Control Total	Weight	Mean Difference IV, Random, 95% CI
Kleffelgaard 2019	32.9	21.3	32	36.4	22.7	31	100.0%	-3.50 [-14.38, 7.38]
**Total (95% CI)**			**32**			**31**	**100.0%**	**-3.50 [-14.38, 7.38]**

Heterogeneity: Not applicable
Test for overall effect: Z = 0.63 (P = 0.53)

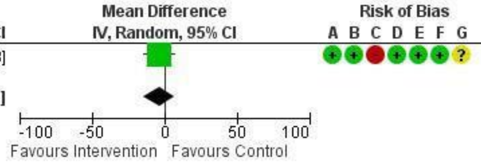

Risk of bias legend
(A) Random sequence generation (selection bias)
(B) Allocation concealment (selection bias)
(C) Blinding of participants and personnel (performance bias)
(D) Blinding of outcome assessment (detection bias)
(E) Incomplete outcome data (attrition bias)
(F) Selective reporting (reporting bias)
(G) Other bias

1.2 Physical Functioning (End of Treatment) (HiMAT)

Study or Subgroup	Intervention Mean	Intervention SD	Intervention Total	Control Mean	Control SD	Control Total	Weight	Mean Difference IV, Fixed, 95% CI
Kleffelgaard 2019	47.6	7	29	41.2	12.3	23	100.0%	6.40 [0.76, 12.04]
**Total (95% CI)**			**29**			**23**	**100.0%**	**6.40 [0.76, 12.04]**

Heterogeneity: Not applicable
Test for overall effect: Z = 2.23 (P = 0.03)

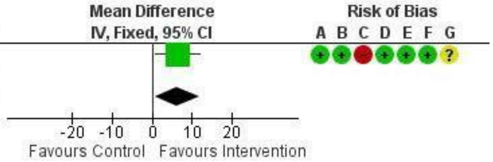

Risk of bias legend
(A) Random sequence generation (selection bias)
(B) Allocation concealment (selection bias)
(C) Blinding of participants and personnel (performance bias)
(D) Blinding of outcome assessment (detection bias)
(E) Incomplete outcome data (attrition bias)
(F) Selective reporting (reporting bias)
(G) Other bias

1.3 Number of Patients Assessed Ready to Return to Sport (End of Treatment)

Study or Subgroup	Intervention Events	Intervention Total	Control Events	Control Total	Weight	Risk Ratio IV, Fixed, 95% CI
Schneider 2014	11	15	1	14	100.0%	10.27 [1.52, 69.55]
**Total (95% CI)**		**15**		**14**	**100.0%**	**10.27 [1.52, 69.55]**
Total events	11		1			

Heterogeneity: Not applicable
Test for overall effect: Z = 2.39 (P = 0.02)

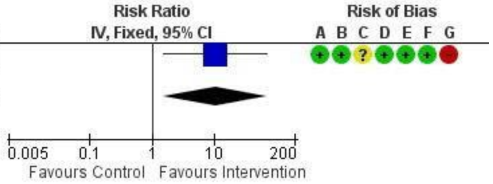

Risk of bias legend
(A) Random sequence generation (selection bias)
(B) Allocation concealment (selection bias)
(C) Blinding of participants and personnel (performance bias)
(D) Blinding of outcome assessment (detection bias)
(E) Incomplete outcome data (attrition bias)
(F) Selective reporting (reporting bias)
(G) Other bias

1.4 Physical Functioning (Follow-up, 2 months after EoT) (HiMAT)

Study or Subgroup	Intervention Mean	Intervention SD	Intervention Total	Control Mean	Control SD	Control Total	Weight	Mean Difference IV, Fixed, 95% CI
Kleffelgaard 2019	47.3	8.2	25	44.3	9.6	26	100.0%	3.00 [-1.89, 7.89]
**Total (95% CI)**			**25**			**26**	**100.0%**	**3.00 [-1.89, 7.89]**

Heterogeneity: Not applicable
Test for overall effect: Z = 1.20 (P = 0.23)

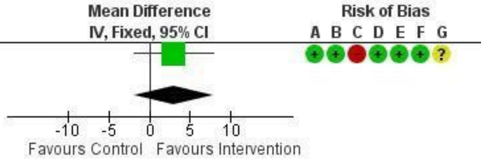

Risk of bias legend
(A) Random sequence generation (selection bias)
(B) Allocation concealment (selection bias)
(C) Blinding of participants and personnel (performance bias)
(D) Blinding of outcome assessment (detection bias)
(E) Incomplete outcome data (attrition bias)
(F) Selective reporting (reporting bias)
(G) Other bias

© 2021 Rytter HM et al. *JAMA Network Open.*

1.5 Dizziness (Follow-up, 2 months after EoT)

	Intervention			Control				Mean Difference
Study or Subgroup	Mean	SD	Total	Mean	SD	Total	Weight	IV, Fixed, 95% CI
Kleffelgaard 2019	32.1	20.7	27	30	24.3	28	100.0%	2.10 [-9.82, 14.02]
**Total (95% CI)**			**27**			**28**	**100.0%**	**2.10 [-9.82, 14.02]**

Heterogeneity: Not applicable
Test for overall effect: Z = 0.35 (P = 0.73)

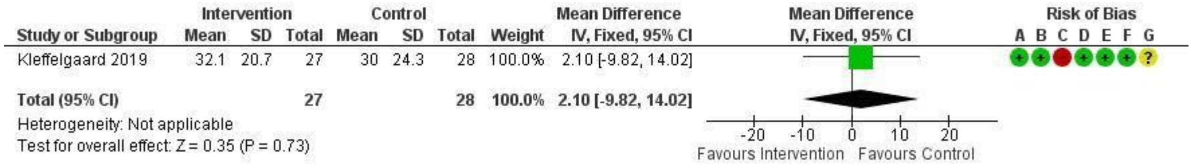

Risk of bias legend
(A) Random sequence generation (selection bias)
(B) Allocation concealment (selection bias)
(C) Blinding of participants and personnel (performance bias)
(D) Blinding of outcome assessment (detection bias)
(E) Incomplete outcome data (attrition bias)
(F) Selective reporting (reporting bias)
(G) Other bias

1.6 Emotional Symptoms, Anxiety (Follow-up, 2 months after EoT)

	Intervention			Control				Mean Difference
Study or Subgroup	Mean	SD	Total	Mean	SD	Total	Weight	IV, Fixed, 95% CI
Kleffelgaard 2019	7.6	5	27	6.9	3.9	28	100.0%	0.70 [-1.68, 3.08]
**Total (95% CI)**			**27**			**28**	**100.0%**	**0.70 [-1.68, 3.08]**

Heterogeneity: Not applicable
Test for overall effect: Z = 0.58 (P = 0.56)

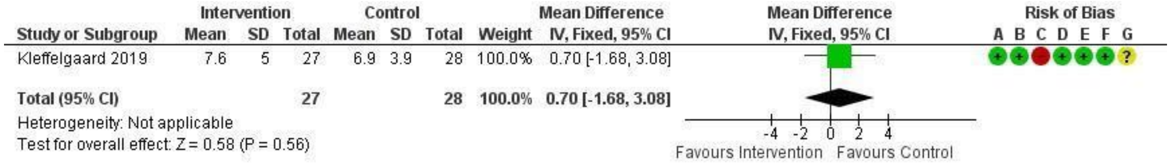

Risk of bias legend
(A) Random sequence generation (selection bias)
(B) Allocation concealment (selection bias)
(C) Blinding of participants and personnel (performance bias)
(D) Blinding of outcome assessment (detection bias)
(E) Incomplete outcome data (attrition bias)
(F) Selective reporting (reporting bias)
(G) Other bias

1.7 Emotional Symptoms, Depression (Follow-up, 2 months after EoT)

	Intervention			Control				Mean Difference
Study or Subgroup	Mean	SD	Total	Mean	SD	Total	Weight	IV, Fixed, 95% CI
Kleffelgaard 2019	6.7	5.2	27	5.1	4.8	28	100.0%	1.60 [-1.05, 4.25]
**Total (95% CI)**			**27**			**28**	**100.0%**	**1.60 [-1.05, 4.25]**

Heterogeneity: Not applicable
Test for overall effect: Z = 1.18 (P = 0.24)

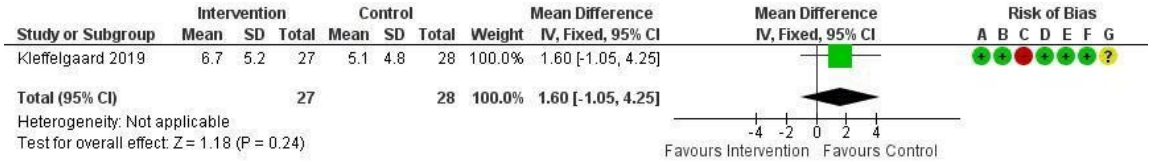

Risk of bias legend
(A) Random sequence generation (selection bias)
(B) Allocation concealment (selection bias)
(C) Blinding of participants and personnel (performance bias)
(D) Blinding of outcome assessment (detection bias)
(E) Incomplete outcome data (attrition bias)
(F) Selective reporting (reporting bias)
(G) Other bias

© 2021 Rytter HM et al. *JAMA Network Open.*

Figures

1.1 Number of Patients Assessed Ready to return to Sport (End of Treatment)

Study or Subgroup	Intervention Events	Intervention Total	Control Events	Control Total	Weight	Risk Ratio IV, Fixed, 95% CI	Risk of Bias A	B	C	D	E	F	G
Schneider 2014	11	15	1	14	100.0%	10.27 [1.52, 69.55]	+	+	?	+	+	+	−
**Total (95% CI)**		**15**		**14**	**100.0%**	**10.27 [1.52, 69.55]**							
Total events	11		1										

Heterogeneity: Not applicable
Test for overall effect: Z = 2.39 (P = 0.02)

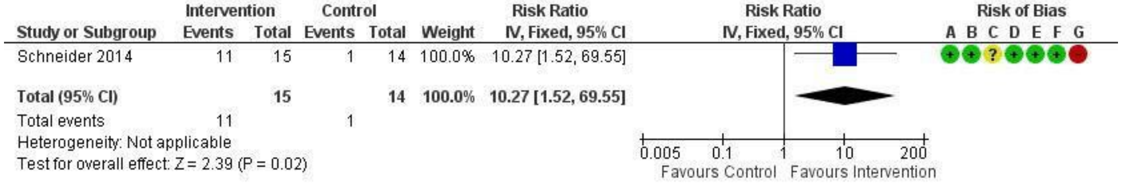

Risk of bias legend
(A) Random sequence generation (selection bias)
(B) Allocation concealment (selection bias)
(C) Blinding of participants and personnel (performance bias)
(D) Blinding of outcome assessment (detection bias)
(E) Incomplete outcome data (attrition bias)
(F) Selective reporting (reporting bias)
(G) Other bias

© 2021 Rytter HM et al. *JAMA Network Open.*

1 Intervention vs Control

1.1 The Collective Burden of Post-concussion Symptoms (End of treatment) (Rivermead)

Study or Subgroup	Intervention Mean	Intervention SD	Intervention Total	Control Mean	Control SD	Control Total	Weight	Mean Difference IV, Random, 95% CI
Potter 2016	26.3	16.4	25	27.8	9	20	15.1%	-1.50 [-9.04, 6.04]
Rytter 2019	32.29	14.18	45	37.5	8.48	44	36.6%	-5.21 [-10.05, -0.37]
Silverberg 2013	17.9	14.5	13	28.7	14.5	11	6.3%	-10.80 [-22.44, 0.84]
Thastum 2019	23.5	13.25	57	27.9	11.07	55	42.0%	-4.40 [-8.92, 0.12]
**Total (95% CI)**			**140**			**130**	**100.0%**	**-4.66 [-7.59, -1.74]**

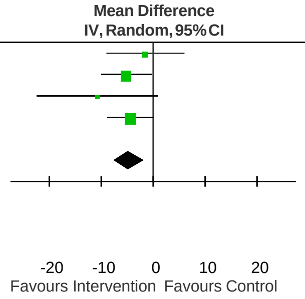

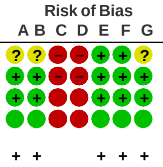

Heterogeneity: Tau² = 0.00; Chi² = 1.80, df = 3 (P = 0.61); I² = 0%
Test for overall effect: Z = 3.12 (P = 0.002)

Risk of bias legend
(A) Random sequence generation (selection bias)
(B) Allocation concealment (selection bias)
(C) Blinding of participants and personnel (performance bias)
(D) Blinding of outcome assessment (detection bias)
(E) Incomplete outcome data (attrition bias)
(F) Selective reporting (reporting bias)
(G) Other bias

1.2 Number of Patients with Post-concussion Symptoms (End of treatment)

Study or Subgroup	Intervention Events	Intervention Total	Control Events	Control Total	Weight	Risk Ratio IV, Random, 95% CI
Caplain 2019	2	34	24	46	42.5%	0.11 [0.03, 0.44]
Silverberg 2013	7	13	10	11	57.5%	0.59 [0.35, 1.01]
**Total (95% CI)**		**47**		**57**	**100.0%**	**0.29 [0.06, 1.46]**
Total events	9		34			

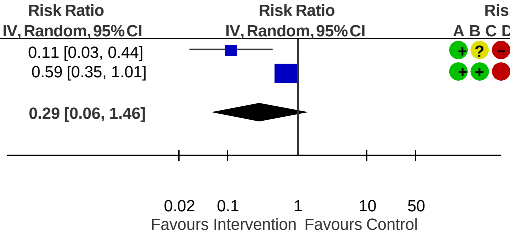

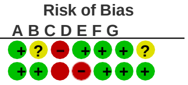

Heterogeneity: Tau² = 1.09; Chi² = 4.87, df = 1 (P = 0.03); I² = 79%
Test for overall effect: Z = 1.50 (P = 0.13)

Risk of bias legend
(A) Random sequence generation (selection bias)
(B) Allocation concealment (selection bias)
(C) Blinding of participants and personnel (performance bias)
(D) Blinding of outcome assessment (detection bias)
(E) Incomplete outcome data (attrition bias)
(F) Selective reporting (reporting bias)
(G) Other bias

© 2021 Rytter HM et al. *JAMA Network Open.*

1.3 The Collective Burden of Post-concussion Symptoms (Follow-up, 3 - 7 months after EoT) (Rivermead)

Study or Subgroup	Intervention			Control			Weight	Std. Mean Difference
	Mean	SD	Total	Mean	SD	Total		IV, Random, 95% CI
ElgmarkAndersson 2007	-5.2	5.3	226	-4.4	5.3	101	36.7%	-0.15 [-0.39, 0.08]
Kjeldgaard 2014	-32	12.6	34	-25.7	12.8	37	11.0%	-0.49 [-0.96, -0.02]
Rytter 2019	29.69	12.92	45	35.3	7.57	44	13.5%	-0.52 [-0.95, -0.10]
Thastum 2019	-17.4	17.41	57	-9.9	12.48	55	16.7%	-0.49 [-0.87, -0.11]
Vikane 2017	14	35.5	81	21.5	37.03	70	22.1%	-0.21 [-0.53, 0.11]
**Total (95% CI)**			**443**			**307**	**100.0%**	**-0.31 [-0.47, -0.14]**

Heterogeneity: Tau² = 0.00; Chi² = 4.56, df = 4 (P = 0.34); I² = 12%
Test for overall effect: Z = 3.70 (P = 0.0002)

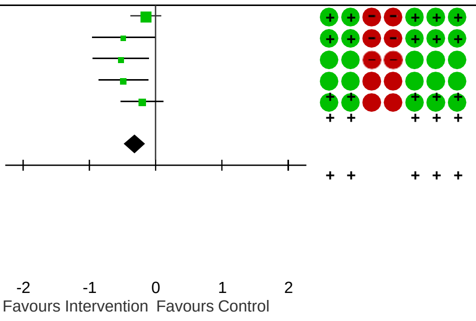

Risk of bias legend
(A) Random sequence generation (selection bias)
(B) Allocation concealment (selection bias)
(C) Blinding of participants and personnel (performance bias)
(D) Blinding of outcome assessment (detection bias)
(E) Incomplete outcome data (attrition bias)
(F) Selective reporting (reporting bias)
(G) Other bias

1.4 Physical Functioning (Follow-up, 3 - 12 months after EoT)

Study or Subgroup	Intervention			Control			Weight	Std. Mean Difference
	Mean	SD	Total	Mean	SD	Total		IV, Random, 95% CI
ElgmarkAndersson 2007	90.6	17.26	226	90.6	15.38	101	30.5%	0.00 [-0.23, 0.23]
Kjeldgaard 2014	70.7	15.7	35	75.4	18.4	37	21.5%	-0.27 [-0.74, 0.19]
Rytter 2019	76.33	20.15	45	67.96	20.15	44	23.2%	0.41 [-0.01, 0.83]
Thastum 2019	9.3	7.91	57	4.7	8.5	55	24.8%	0.56 [0.18, 0.93]
**Total (95% CI)**			**363**			**237**	**100.0%**	**0.18 [-0.17, 0.52]**

Heterogeneity: Tau² = 0.09; Chi² = 10.66, df = 3 (P = 0.01); I² = 72%
Test for overall effect: Z = 1.00 (P = 0.32)

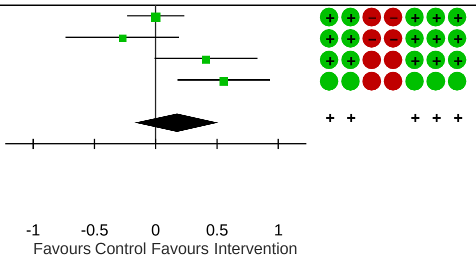

Risk of bias legend
(A) Random sequence generation (selection bias)
(B) Allocation concealment (selection bias)
(C) Blinding of participants and personnel (performance bias)
(D) Blinding of outcome assessment (detection bias)
(E) Incomplete outcome data (attrition bias)
(F) Selective reporting (reporting bias)
(G) Other bias

© 2021 Rytter HM et al. *JAMA Network Open.*

1.5 Emotional Symptoms (Follow-up, 3 - 7 months after EoT)

	Intervention			Control				Std. Mean Difference
Study or Subgroup	Mean	SD	Total	Mean	SD	Total	Weight	IV, Random, 95% CI
Kjeldgaard 2014	-59.3	8.89	34	-55.7	9.79	36	17.5%	-0.38 [-0.85, 0.09]
Rytter 2019	13.07	9.27	45	18.52	10.29	44	21.4%	-0.55 [-0.98, -0.13]
Thastum 2019	-0.7	1.13	57	-0.4	1.03	55	26.7%	-0.28 [-0.65, 0.10]
Vikane 2017	8	23.07	81	9.5	22.2	70	34.4%	-0.07 [-0.39, 0.25]
**Total (95% CI)**			**217**			**205**	**100.0%**	**-0.28 [-0.49, -0.07]**

Heterogeneity: Tau² = 0.01; Chi² = 3.47, df = 3 (P = 0.32); I² = 14%
Test for overall effect: Z = 2.64 (P = 0.008)

Std. Mean Difference IV, Random, 95% CI — Risk of Bias A B C D E F G

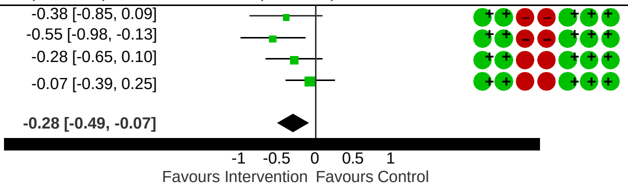

Risk of bias legend
(A) Random sequence generation (selection bias)
(B) Allocation concealment (selection bias)
(C) Blinding of participants and personnel (performance bias)
(D) Blinding of outcome assessment (detection bias)
(E) Incomplete outcome data (attrition bias)
(F) Selective reporting (reporting bias)
(G) Other bias

1.6 Behavioral Responses (End of Treatment)

	Intervention			Control				Mean Difference
Study or Subgroup	Mean	SD	Total	Mean	SD	Total	Weight	IV, Fixed, 95% CI
Thastum 2019	38.8	21.35	57	42.9	20.94	55	100.0%	-4.10 [-11.93, 3.73]
**Total (95% CI)**			**57**			**55**	**100.0%**	**-4.10 [-11.93, 3.73]**

Heterogeneity: Not applicable
Test for overall effect: Z = 1.03 (P = 0.30)

Mean Difference IV, Fixed, 95% CI — Risk of Bias A B C D E F G

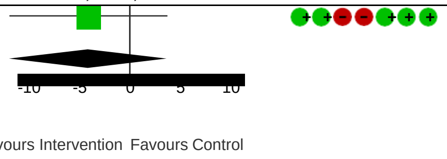

Risk of bias legend
(A) Random sequence generation (selection bias)
(B) Allocation concealment (selection bias)
(C) Blinding of participants and personnel (performance bias)
(D) Blinding of outcome assessment (detection bias)
(E) Incomplete outcome data (attrition bias)
(F) Selective reporting (reporting bias)
(G) Other bias

1.7 Quality of Life (Follow-up, 3 - 6 months after EoT)

	Intervention			Control				Std. Mean Difference
Study or Subgroup	Mean	SD	Total	Mean	SD	Total	Weight	IV, Random, 95% CI
Kjeldgaard 2014	52.5	20.8	35	49.5	19.7	37	30.0%	0.15 [-0.32, 0.61]
Rytter 2019	3.33	1.19	45	2.52	1.02	44	32.5%	0.72 [0.29, 1.15]
Thastum 2019	23.5	27.15	57	16.8	25.16	55	37.5%	0.25 [-0.12, 0.63]
**Total (95% CI)**			**137**			**136**	**100.0%**	**0.37 [0.04, 0.71]**

Heterogeneity: Tau² = 0.04; Chi² = 3.88, df = 2 (P = 0.14); I² = 48%
Test for overall effect: Z = 2.18 (P = 0.03)

Std. Mean Difference IV, Random, 95% CI — Risk of Bias A B C D E F G

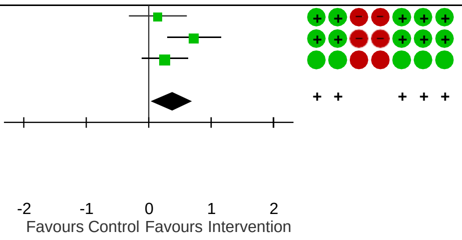

Risk of bias legend
(A) Random sequence generation (selection bias)
(B) Allocation concealment (selection bias)
(C) Blinding of participants and personnel (performance bias)
(D) Blinding of outcome assessment (detection bias)
(E) Incomplete outcome data (attrition bias)
(F) Selective reporting (reporting bias)
(G) Other bias

© 2021 Rytter HM et al. *JAMA Network Open.*

1.8 Return to usual Activity Levels (Follow-up, 3 - 7 months after EoT)

	Intervention			Control				Std. Mean Difference	Risk of Bias
**Study or Subgroup**	**Mean**	**SD**	**Total**	**Mean**	**SD**	**Total**	**Weight**	**IV, Random, 95% CI**	**A B C D E F G**
ElgmarkAndersson 2007	-20.3	4	226	-19.8	4	101	58.4%	-0.12 [-0.36, 0.11]	+ + − − + + +
Rytter 2019	57.11	23.32	45	68.86	17.58	44	41.6%	-0.56 [-0.99, -0.14]	
**Total (95% CI)**			**271**			**145**	**100.0%**	**-0.31 [-0.73, 0.12]**	

Heterogeneity: Tau² = 0.07; Chi² = 3.14, df = 1 (P = 0.08); I² = 68%
Test for overall effect: Z = 1.42 (P = 0.16)

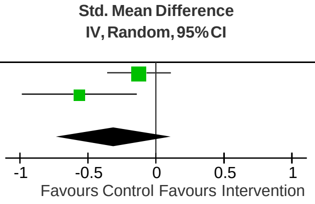

Risk of bias legend
(A) Random sequence generation (selection bias)
(B) Allocation concealment (selection bias)
(C) Blinding of participants and personnel (performance bias)
(D) Blinding of outcome assessment (detection bias)
(E) Incomplete outcome data (attrition bias)
(F) Selective reporting (reporting bias)
(G) Other bias

1.9 Number of Patients Return to Work (Follow-up, 7 months after EoT)

	Intervention		Control			Risk Ratio	Risk of Bias
**Study or Subgroup**	**Events**	**Total**	**Events**	**Total**	**Weight**	**IV, Random, 95% CI**	**A B C D E F G**
Vikane 2017	49	81	50	70	100.0%	0.85 [0.67, 1.07]	+ + − − + + +
**Total (95% CI)**		**81**		**70**	**100.0%**	**0.85 [0.67, 1.07]**	
Total events	49		50				

Heterogeneity: Not applicable
Test for overall effect: Z = 1.42 (P = 0.16)

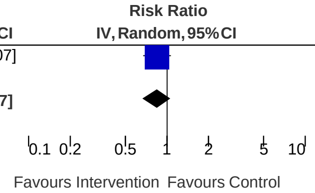

Risk of bias legend
(A) Random sequence generation (selection bias)
(B) Allocation concealment (selection bias)
(C) Blinding of participants and personnel (performance bias)
(D) Blinding of outcome assessment (detection bias)
(E) Incomplete outcome data (attrition bias)
(F) Selective reporting (reporting bias)
(G) Other bias

© 2021 Rytter HM et al. *JAMA Network Open.*

PICO 7 Interdisciplinary Coordinated Rehabilitative Treatment

1 Intervention vs Control

1.1 The Collective Burden of Symptoms, (End of Treatment) (Rivermead)

	Intervention			Control				Mean Difference
**Study or Subgroup**	**Mean**	**SD**	**Total**	**Mean**	**SD**	**Total**	**Weight**	**IV, Random, 95% CI**
Rytter 2019	32.29	14.18	45	37.5	8.48	44	46.5%	-5.21 [-10.05, -0.37]
Thastum 2019	23.5	13.25	57	27.9	11.07	55	53.5%	-4.40 [-8.92, 0.12]
**Total (95% CI)**			**102**			**99**	**100.0%**	**-4.78 [-8.08, -1.47]**

Heterogeneity: Tau² = 0.00; Chi² = 0.06, df = 1 (P = 0.81); I² = 0%
Test for overall effect: Z = 2.84 (P = 0.005)

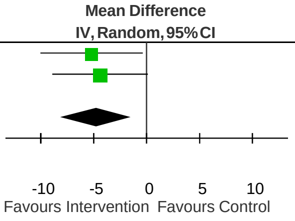

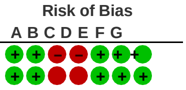

Risk of bias legend
(A) Random sequence generation (selection bias)
(B) Allocation concealment (selection bias)
(C) Blinding of participants and personnel (performance bias)
(D) Blinding of outcome assessment (detection bias)
(E) Incomplete outcome data (attrition bias)
(F) Selective reporting (reporting bias)
(G) Other bias

1.2 The Collective Burden of Symptoms, (Follow-up, 3 - 9 months after EoT) (Rivermead)

	Intervention			Control				Mean Difference
**Study or Subgroup**	**Mean**	**SD**	**Total**	**Mean**	**SD**	**Total**	**Weight**	**IV, Random, 95% CI**
Rytter 2019	29.69	12.92	45	35.3	7.57	44	56.9%	-5.61 [-10.00, -1.22]
Thastum 2019	-17.4	17.41	57	-9.9	12.48	55	35.0%	-7.50 [-13.10, -1.90]
Vikane 2017	14	35.5	81	21.5	37.03	70	8.1%	-7.50 [-19.12, 4.12]
**Total (95% CI)**			**183**			**169**	**100.0%**	**-6.42 [-9.73, -3.11]**

Heterogeneity: Tau² = 0.00; Chi² = 0.31, df = 2 (P = 0.86); I² = 0%
Test for overall effect: Z = 3.80 (P = 0.0001)

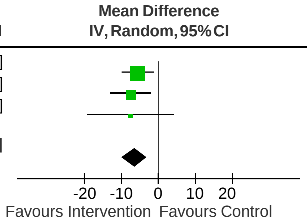

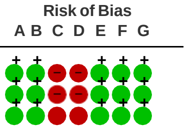

Risk of bias legend
(A) Random sequence generation (selection bias)
(B) Allocation concealment (selection bias)
(C) Blinding of participants and personnel (performance bias)
(D) Blinding of outcome assessment (detection bias)
(E) Incomplete outcome data (attrition bias)
(F) Selective reporting (reporting bias)
(G) Other bias

1.3 Physical Functioning (End of Treatment)

	Intervention			Control				Std. Mean Difference
**Study or Subgroup**	**Mean**	**SD**	**Total**	**Mean**	**SD**	**Total**	**Weight**	**IV, Random, 95% CI**
Rytter 2019	75.11	18.93	45	66.14	19.07	44	43.7%	0.47 [0.05, 0.89]
Thastum 2019	-38.8	45.2257	57	-45	10.3574	55	56.3%	0.19 [-0.19, 0.56]
**Total (95% CI)**			**102**			**99**	**100.0%**	**0.31 [0.03, 0.59]**

Heterogeneity: Tau² = 0.00; Chi² = 0.97, df = 1 (P = 0.33); I² = 0%
Test for overall effect: Z = 2.18 (P = 0.03)

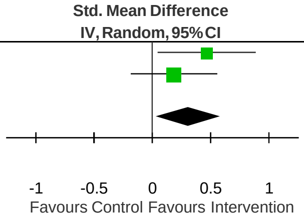

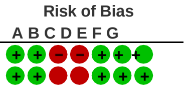

Risk of bias legend
(A) Random sequence generation (selection bias)
(B) Allocation concealment (selection bias)
(C) Blinding of participants and personnel (performance bias)
(D) Blinding of outcome assessment (detection bias)
(E) Incomplete outcome data (attrition bias)
(F) Selective reporting (reporting bias)
(G) Other bias

© 2021 Rytter HM et al. *JAMA Network Open.*

1.4 Emotional symptoms (Follow-up, 3 - 9 months after EoT)

	Intervention			Control				Std. Mean Difference
**Study or Subgroup**	**Mean**	**SD**	**Total**	**Mean**	**SD**	**Total**	**Weight**	**IV, Random, 95% CI**
Rytter 2019	13.07	9.27	45	18.52	10.29	44	27.8%	-0.55 [-0.98, -0.13]
Thastum 2019	-0.7	1.13	57	-0.4	1.03	55	32.9%	-0.28 [-0.65, 0.10]
Vikane 2017	8	23.07	81	9.5	22.2	70	39.3%	-0.07 [-0.39, 0.25]
**Total (95% CI)**			**183**			**169**	**100.0%**	**-0.27 [-0.54, 0.00]**

Heterogeneity: Tau² = 0.02; Chi² = 3.24, df = 2 (P = 0.20); I² = 38%
Test for overall effect: Z = 1.95 (P = 0.05)

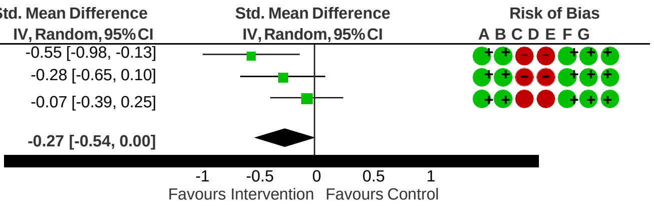

Risk of bias legend
(A) Random sequence generation (selection bias)
(B) Allocation concealment (selection bias)
(C) Blinding of participants and personnel (performance bias)
(D) Blinding of outcome assessment (detection bias)
(E) Incomplete outcome data (attrition bias)
(F) Selective reporting (reporting bias)
(G) Other bias

1.5 Behavioral Responses (End of Treatment)

	Intervention			Control				Mean Difference
**Study or Subgroup**	**Mean**	**SD**	**Total**	**Mean**	**SD**	**Total**	**Weight**	**IV, Fixed, 95% CI**
Thastum 2019	38.8	21.35	57	42.9	20.94	55	100.0%	-4.10 [-11.93, 3.73]
**Total (95% CI)**			**57**			**55**	**100.0%**	**-4.10 [-11.93, 3.73]**

Heterogeneity: Not applicable
Test for overall effect: Z = 1.03 (P = 0.30)

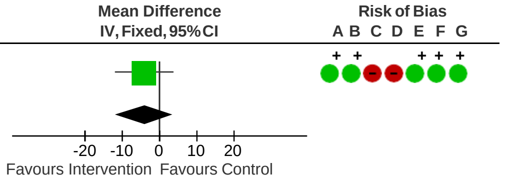

Risk of bias legend
(A) Random sequence generation (selection bias)
(B) Allocation concealment (selection bias)
(C) Blinding of participants and personnel (performance bias)
(D) Blinding of outcome assessment (detection bias)
(E) Incomplete outcome data (attrition bias)
(F) Selective reporting (reporting bias)
(G) Other bias

© 2021 Rytter HM et al. *JAMA Network Open.*

1.7 Quality of Life (Follow-up, 3 - 9 months after EoT)

Study or Subgroup	Intervention Mean	Intervention SD	Intervention Total	Control Mean	Control SD	Control Total	Weight	Std. Mean Difference IV, Random, 95% CI
Rytter 2019	3.33	1.19	45	2.52	1.02	44	47.3%	0.72 [0.29, 1.15]
Thastum 2019	23.5	27.15	57	16.8	25.16	55	52.7%	0.25 [-0.12, 0.63]
**Total (95% CI)**			**102**			**99**	**100.0%**	**0.48 [0.02, 0.94]**

Heterogeneity: Tau² = 0.07; Chi² = 2.63, df = 1 (P = 0.11); I² = 62%
Test for overall effect: Z = 2.03 (P = 0.04)

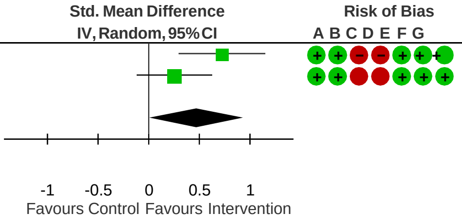

Risk of bias legend
(A) Random sequence generation (selection bias)
(B) Allocation concealment (selection bias)
(C) Blinding of participants and personnel (performance bias)
(D) Blinding of outcome assessment (detection bias)
(E) Incomplete outcome data (attrition bias)
(F) Selective reporting (reporting bias)
(G) Other bias

1.8 Pain, Headache (End of Treatment)

Study or Subgroup	Intervention Mean	Intervention SD	Intervention Total	Control Mean	Control SD	Control Total	Weight	Mean Difference IV, Fixed, 95% CI
Rytter 2019	2.35	4.5	45	0.93	3.47	44	100.0%	1.42 [-0.25, 3.09]
**Total (95% CI)**			**45**			**44**	**100.0%**	**1.42 [-0.25, 3.09]**

Heterogeneity: Not applicable
Test for overall effect: Z = 1.67 (P = 0.10)

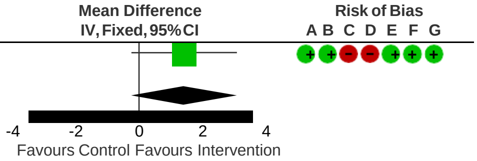

Risk of bias legend
(A) Random sequence generation (selection bias)
(B) Allocation concealment (selection bias)
(C) Blinding of participants and personnel (performance bias)
(D) Blinding of outcome assessment (detection bias)
(E) Incomplete outcome data (attrition bias)
(F) Selective reporting (reporting bias)
(G) Other bias

1.9 General Satisfaction with Current Working Conditions, (Follow-up, 6 months after EoT)

Study or Subgroup	Intervention Mean	Intervention SD	Intervention Total	Control Mean	Control SD	Control Total	Weight	Mean Difference IV, Fixed, 95% CI
Rytter 2019	2.47	1.4	45	1.84	1.1	44	100.0%	0.63 [0.11, 1.15]
**Total (95% CI)**			**45**			**44**	**100.0%**	**0.63 [0.11, 1.15]**

Heterogeneity: Not applicable
Test for overall effect: Z = 2.36 (P = 0.02)

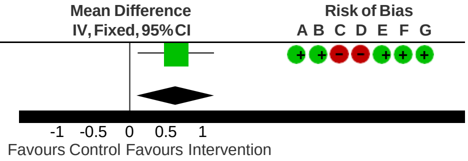

Risk of bias legend
(A) Random sequence generation (selection bias)
(B) Allocation concealment (selection bias)
(C) Blinding of participants and personnel (performance bias)
(D) Blinding of outcome assessment (detection bias)
(E) Incomplete outcome data (attrition bias)
(F) Selective reporting (reporting bias)
(G) Other bias

© 2021 Rytter HM et al. *JAMA Network Open.*

1.10 Return to Work (Follow-up, 6 months after EoT)

Study or Subgroup	Intervention Events	Intervention Total	Control Events	Control Total	Weight	Risk Ratio IV, Fixed, 95% CI	Risk of Bias A	B	C	D	E	F	G
Vikane 2017	49	81	50	70	100.0%	0.85 [0.67, 1.07]	+	+	−	−	+	+	+
**Total (95% CI)**		**81**		**70**	**100.0%**	**0.85 [0.67, 1.07]**							
Total events	49		50										

Heterogeneity: Not applicable
Test for overall effect: Z = 1.42 (P = 0.16)

Risk Ratio
IV, Fixed, 95% CI

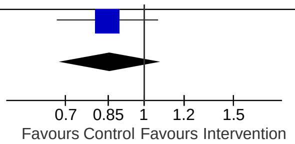

Risk of bias legend
(A) Random sequence generation (selection bias)
(B) Allocation concealment (selection bias)
(C) Blinding of participants and personnel (performance bias)
(D) Blinding of outcome assessment (detection bias)
(E) Incomplete outcome data (attrition bias)
(F) Selective reporting (reporting bias)
(G) Other bias

© 2021 Rytter HM et al. *JAMA Network Open.*

eTable 4. Summary of Findings Table, Including the Rating of the Evidence and Risk of Bias Assessment forPICO 1-7

Early information and advice (PICO 1)

Outcome measures Time frame	Results and measurements	Effect estimates		Confidence in the estimates (that they reflect the true effect in the population).	Summary
		Treatment as usual	Systematically offered early information and advice		
Number of patients with anxiety Follow-up (14 days - 6 months after the end of treatment)	Relative risk: 0.93 (CI 95% 0.69 - 1.26) Based on data from 406 patients in 3 studies[1]	**272** per 1.000	**245** per 1.000	**Very low** due to serious risk of bias, due to serious absence of transferability, due to seriously inaccurate effect estimate[2]	Early information and advice may not affect the number of patients with anxiety at follow-up (14 days - 6 months after the end of treatment)
		Difference: **27 fewer per 1.000** (CI 95% 112 fewer - 103 more)			
Number of patients with depression Follow-up (14 days - 6 months after the end of treatment)	Relative risk: 1.17 (CI 95% 0.19 - 7.1) Based on data from 94 patients in 2 studies[3]	**373** per 1.000	**436** per 1.000	**Very low** Due to very seriously inaccurate effect estimate, due to serious risk of bias, due to seriously inconsistent results[4]	Early information and advice may not affect the number of patients with depression at follow-up (14 days - 6 months after the end of treatment)
		Difference: **63 more per 1.000** (CI 95% 302 fewer - 2275 more)			
Number of patients with memory and concentration problems Follow-up (3-6 months after the end of treatment)	Relative risk: 0.54 (CI 95% 0.39 - 0.75) Based on data from 370 patients in 2 studies[5]	**374** per 1.000	**202** per 1.000	**Low** due to serious risk of bias, due to serious absence of transferability[6]	Early information and advice may affect the number of patients with memory- and concentration problems at follow-up (3-6 months after the end of treatment)
		Difference: **172 fewer per 1.000** (CI 95% 228 fewer - 94 fewer)			

© 2021 Rytter HM et al. *JAMA Network Open.*

Number of patients showing irritability Follow-up (3-6 months after the end of treatment)	Relative risk: 0.8 (CI 95% 0.63 - 1.03) Based on data from 420 patients in 3 studies[7]	**327** per 1.000	**262** per 1.000	**Very low** due to serious risk of bias, due to serious absence of transferability, due to seriously inaccurate effect estimate[8]	Early information and advice may not affect the number of patients showing irritability at follow-up (3-6 months after the end of treatment)
		Difference: **65 fewer per 1.000** (CI 95% 121 fewer - 10 more)			
Number of patients with headache Follow-up (3-6 months after the end of treatment)	Relative risk: 0.87 (CI 95% 0.48 - 1.6) Based on data from 420 patients in 3 studies[9]	**390** per 1.000	**339** per 1.000	**Very low** due to very serious risk of bias, due to seriously inconsistent results, due to serious absence of transferability, due to seriously inaccurate effect estimate[10]	Early information and advice may not affect the number of patients with headache at follow-up (3-6 months after the end of treatment)
		Difference: **51 fewer per 1.000** (CI 95% 207 fewer - 250 more)			
Number of patients with poor walking function Follow-up (3 months after the end of treatment)	Relative risk: 0.85 (CI 95% 0.37 - 1.97) Based on data from 312 patients in 1 studies[11]	**72** per 1.000	**61** per 1.000	**Very low** due to serious absence of transferability, due to serious risk of bias, due to seriously inaccurate effect estimate [12]	Early information and advice may not affect the number of patients with poor walking function at follow-up (3 months after the end of treatment)
		Difference: **11 fewer per 1.000** (CI 95% 45 fewer - 70 more)			
Number of patients with affected leisure time Follow-up (3 months after the end of treatment)	Relative risk: 0.58 (CI 95% 0.36 - 0.95) Based on data from 312 patients in 1 studies[13]	**235** per 1.000	**136** per 1.000	**Very low** Due to serious absence of transferability, due to seriously inaccurate effect estimate, due to serious risk of bias[14]	Early information and advice may reduce the number of patients who experience that leisure time is affected at follow-up (3 months after the end of treatment)
		Difference: **99 fewer per 1.000** (CI 95% 150 fewer - 12 fewer)			
Number of patients with affected work functioning Follow-up (3 months after the end of treatment)	Relative risk: 0.63 (CI 95% 0.41 - 0.95) Based on data from 312 patients in 1 studies[15]	**295** per 1.000	**186** per 1.000	**Very low** Due to serious absence of transferability, due to seriously inaccurate effect estimate, due to serious risk of bias[16]	Early information and advice may reduce the number of patients who experience that work life is affected at follow-up (3 months after the end of treatment)
		Difference: **109 fewer per 1.000** (CI 95% 174 fewer - 15 fewer)			
Number of patients with affected school attendance Follow-up (3 months after the end of treatment)	Relative risk: 0.57 (CI 95% 0.22 - 1.48) Based on data from 18 patients in 1 studies[17]	**72** per 1.000	**41** per 1.000	**Very low** Due to serious absence of transferability, due to seriously inaccurate effect estimate, due to serious risk of bias[18]	Early information and advice may not reduce the number of patients who experience that school attendance is affected at follow-up (3 months after the end of treatment)
		Difference: **31 fewer per 1.000** (CI 95% 56 fewer - 35 more)			
Number of patients with	Relative risk: 1.21 (CI 95% 0.61 - 2.4)	**275** per 1.000	**333** per 1.000	**Very low** due to very seriously inaccurate	Early information and advice may not affect the

© 2021 Rytter HM et al. *JAMA Network Open.*

significant symptom burden measured by PCSS>20 Follow-up (4 weeks after the end of treatment)	Based on data from 76 patients in 1 studies[19]	Difference: **58 more per 1.000** (CI 95% 107 fewer - 385 more)	effect estimate, due to serious risk of bias[20]	number of patients with significant symptom load (measured by PCSS>20) at follow-up (4 weeks after the end of treatment)
The collective burden of post-concussion symptoms Follow-up (6 months after the end of treatment)	Measured with: Frequency of post-concussion symptoms Scale: - Lower better Based on data from 58 patients in 1 study[21]	Difference: **MD 1.48 lower** (CI 95% 2.86 lower - 0.10 lower)	**Very low** due to very serious risk of bias, due to very seriously inaccurate effect estimate[22]	Early information and advice may somewhat reduce the frequency of post-concussion symptoms at follow-up (6 months after the end of treatment)
The collective burden of post-concussion symptoms Follow-up (3 months after the end of treatment)	Measured with: Head injury symptom checklist Scale: 0-16 Higher better Based on data from 312 patients in 1 study[23]	Difference: **MD 6.60 higher** (CI 95% 1.21 higher - 11.99 higher)	**Very low** Due to serious absence of transferability, due to serious risk of bias, due to seriously inaccurate effect estimate[24]	Early information and advice may somewhat lower the collective burden of post-concussion symptoms at follow-up (3 months after the end of treatment).
The collective burden of post-concussion symptoms Follow-up (14 days after the end of treatment)	Measured with: Rivermead postconcussion symptoms questionnaire, total score Scale: 0-64 Lower better Based on data from 36 patients in 1 study[25]	Difference: **MD 3 lower** (CI 95% 15.66 lower - 9.66 higher)	**Very low** due to very seriously inaccurate effect estimate, due to serious risk of bias[26]	Early information and advice may not affect the collective burden of post-concussion symptoms measured with: Rivermead postconcussion scale at follow-up (14 days after the end of treatment)
Emotional symptoms (depression) Follow-up (3 months after the end of treatment)	Measured with: BDI and HADS depression scale Scale: - Lower better Based on data from 147 patients in 2 studies[27]	Difference: **SMD 0.15 lower** (CI 95% 0.47 lower - 0.18 higher)	**Very low** due to very serious risk of bias, due to serious absence of transferability, due to seriously inaccurate effect estimate[28]	Early information and advice may not affect the degree of depression at follow-up (3 months after the end of treatment)
Pain (headache) End of treatment	Measured with: Mean headache score (Likert scale) Scale: 0-10 Lower better Based on data from 43 patients in 1 study[29]	Difference: **MD 0.20 lower** (CI 95% 0.77 lower - 0.37 higher)	**Very low** due to very seriously inaccurate effect estimate, due to very serious risk of bias[30]	Early information and advice may not affect the degree of headache after the end of treatment
Average number of days at work Follow-up (4 weeks after the	Measured with: Average number of days at work Scale: - Higher better Based on data from 90 patients in 1 study[31]	Difference: **MD 1 higher** (CI 95% 0.94 lower - 2.94 higher)	**Very low** due to very seriously inaccurate effect estimate, due to serious risk of bias[32]	Early information and advice may not affect the average number of days at work at follow-up (4 weeks after the end of treatment)

end of treatment)				
Negative effect on prognosis Follow-up (1 month after the end of treatment)				We found no studies that estimated the negative effect on the prognosis at follow-up

1. Meta-analysis for PICO 1 with included studies: Suffoletto et al. 2913, Mittenberg et al. 1996, Bell et al. 2008. **Baseline risk/comparator** Control arm in reference used for the intervention.
2. **Risk of bias: Serious.** Insufficiently hidden randomization, inadequate allocation sequence generation, lack of blinding, insufficient/lack of blinding in the evaluation. **Lack of transferability: Serious**. Patients aged 16 years, or more are included. **Inaccurate effect estimate: Serious.** Wide confidence intervals.
3. Meta-analysis for PICO 1 with included studies: Suffoletto et al. 2013, Mittenberg et al. 1996. **Baseline risk/comparator** Control arm in reference used for the intervention.
4. **Risk of bias: Serious.** Lack of blinding of the participants, inadequate allocation sequence generation, insufficiently hidden randomization, insufficient/lack of blinding in the evaluation; **Inconsistent results**: **Serious**. The statistical heterogeneity is high. I2 = 85%; **Inaccurate effect estimate: Very serious**. Data from only one study, few patients included in the study;
5. Meta-analysis for PICO 1 with included studies: Bell et al. 2008, Mittenberg et al. 1996 **Baseline risk/comparator** Control arm in reference used for the intervention.
6. **Risk of bias: Serious.** inadequate allocation sequence generation, insufficiently hidden randomization, lack of blinding, insufficient/lack of blinding in the evaluation; **Lack of transferability: Serious**. Patients aged 16 years, or more are included
7. Meta-analysis for PICO 1 with included studies: Bell et al. 2008, Heskestad et al. 2010, Mittenberg et al. 1996. **Baseline risk/comparator** Control arm in reference used for the intervention.
8. **Risk of bias: Very serious.** inadequate allocation sequence generation, insufficiently hidden randomization, lack of blinding, insufficient/lack of blinding in the evaluation.; **Lack of transferability: Serious**. Patients aged 16 years, or more are included. **Inaccurate effect estimate: Serious**. Wide confidence intervals.
9. Meta-analysis for PICO 1 with included studies: Bell et al. 2008, Heskestad et al. 2010, Mittenberg et al. 1996. **Baseline risk/comparator** Control arm in reference used for the intervention.
10. **Risk of bias: Very serious.** inadequate allocation sequence generation, insufficiently hidden randomization, lack of blinding, insufficient/lack of blinding in the evaluation.; **Inconsistent results**: Serious. The statistical heterogeneity is high (77%); **Lack of transferability: Serious**. Patients aged 16 years, or more are included; **Inaccurate effect estimate: Serious**. Wide confidence intervals.
11. Meta-analysis for PICO 1 with included studies: Bell et al. 2008. **Baseline risk/comparator** Control arm in reference used for the intervention.
12. **Risk of bias: Serious.** Lack of blinding, insufficient/lack of blinding in the evaluation.; **Lack of transferability: Serious**. Patients aged 16 years, or more are included; **Inaccurate effect estimate: Serious**. Wide confidence intervals, only data from one study.
13. Meta-analysis for PICO 1 with included studies: Bell et al. 2008. **Baseline risk/comparator** Control arm in reference used for the intervention.
14. **Risk of bias: Serious.** Insufficient/lack of blinding in the evaluation, lack of blinding.; **Lack of transferability: Serious**. Patients aged 16 years, or more are included; **Inaccurate effect estimate: Serious.** Only data from one study, wide confidence intervals.
15. Meta-analysis for PICO 1 with included studies: Bell et al. 2008 **Baseline risk/comparator** Control arm in reference used for the intervention.
16. **Risk of bias: Serious.** Lack of blinding, insufficient/lack of blinding in the evaluation.; **Lack of transferability: Serious**. Patients aged 16 years, or more are included; **Inaccurate effect estimate: Serious**. Only data from one study.
17. Meta-analysis for PICO 1 with included studies: Bell et al. 2008. **Baseline risk/comparator** Control arm in reference used for the intervention.
18. **Risk of bias: Serious.** Lack of blinding, insufficient/lack of blinding in the evaluation.; **Lack of transferability: Serious**. Patients aged 16 years, or more are included; Inaccurate effect estimate: Serious; Only data from one study, Wide confidence intervals.
19. Meta-analysis for PICO 1 with included studies: Varner et al. 2017 **Baseline risk/comparator** Control arm in reference used for the intervention.
20. **Risk of bias: Serious.** Lack of blinding of the participants, insufficient/lack of blinding in the evaluation.; **Inaccurate effect estimate: Very serious,** Only data from one study, few patients included in the studies, wide confidence intervals.
21. Meta-analysis for PICO 1 with included studies: Mittenberg et al. 1996 **Baseline risk/comparator** Control arm in reference used for the intervention.

© 2021 Rytter HM et al. *JAMA Network Open.*

22. **Risk of bias: Very serious.** inadequate allocation sequence generation, insufficiently hidden randomization, lack of blinding, insufficient/lack of blinding in the evaluation; **Inaccurate effect estimate: Very Serious**. Wide confidence intervals, few patients included in the studies, only data from one study.

23. Meta-analysis for PICO 1 with included studies: Bell et al. 2008. **Baseline risk/comparator** Control arm in reference used for the intervention.

24. **Risk of bias: Serious.** Lack of blinding, insufficient/lack of blinding in the evaluation.; **Lack of transferability: Serious**. Patients aged 16 years, or more are included; **Inaccurate effect estimate: Serious**. Wide confidence intervals, only data from one study;

25. Meta-analysis for PICO 1 with included studies: Suffoletto et al. 2013. **Baseline risk/comparator** Control arm in reference used for the intervention.

26. **Risk of bias: Serious.** Lack of blinding, insufficient/lack of blinding in the evaluation.; **Inaccurate effect estimate: Very serious**. Wide confidence intervals, only data from one study, few patients (<100) /(100-300) included in the studies.

27. Meta-analysis for PICO 1 with included studies: Matuseviciene et al. 2013, Heskestad et al. 2010. **Baseline risk/comparator** Control arm in reference used for the intervention.

28. **Risk of bias: Very serious.** Incomplete data/or large dropout, selective reporting of outcome, inadequate allocation sequence generation, insufficiently hidden randomization, lack of blinding, insufficient/lack of blinding in the evaluation.; **Lack of transferability: Serious**. Patients aged 15 years or more are included.; Inaccurate effect estimate: Serious. Wide confidence intervals.

29. Meta-analysis for PICO 1 with included studies: Suffoletto et al. 2013 **Baseline risk/comparator** Control arm in reference used for the intervention.

30. **Risk of bias: Serious.** Lack of blinding, lack of blinding, insufficient/lack of blinding in the evaluation.; **Inaccurate effect estimate: Very serious**; few patients included in the studies, wide confidence intervals, only data from one study.

31. Meta-analysis for PICO 1 with included studies: Varner et al. 2017 **Baseline risk/comparator** Control arm in reference used for the intervention.

32. **Risk of bias: Serious.** Lack of blinding, insufficient/lack of blinding in the evaluation.; **Inaccurate effect estimate: Very serious**. Wide confidence intervals, few patients included in the studies, only data from one study.

References

Meta-analysis for PICO 1: Early information and advice

Bell KR, Hoffman JM, Temkin NR et al.: The effect of telephone counselling on reducing post-traumatic symptoms after mild traumatic brain injury: a randomised trial. Journal of Neurology, Neurosurgery & Psychiatry 2008;79(11):1275-1281.

Heskestad B, Waterloo K, Baardsen R, et al: No impact of early intervention on late outcome after minimal, mild and moderate head injury. Scandinavian Journal of Trauma, Resuscitation & Emergency Medicine 2010;18 10

Matuseviciene G, Borg J, Stalnacke B-M, Ulfarsson T, et al.: Early intervention for patients at risk for persisting disability after mild traumatic brain injury: a randomized, controlled study. Brain Injury 2013;27(3):318-324

Mittenberg W., Tremont G., Zielinski RE et al.: Cognitive-behavioral prevention of postconcussion syndrome. Archives of Clinical Neuropsychology 1996;11(2):139-145.

Ponsford J., Willmott C., Rothwell A., et al.: Impact of early intervention on outcome following mild head injury in adults. 2002;73(3):330-332

Suffoletto B, Wagner AK, Arenth PM et al.: Mobile phone text messaging to assess symptoms after mild traumatic brain injury and provide self-care support: a pilot study. Journal of Head Trauma Rehabilitation 2013;28(4):302-312.

© 2021 Rytter HM et al. *JAMA Network Open.*

Varner CE, McLeod S, Nahiddi N, et al.: Cognitive Rest and Graduated Return to Usual Activities Versus Usual Care for Mild Traumatic Brain Injury: A Randomized Controlled Trial of Emergency Department Discharge Instructions. Academic Emergency Medicine 2017;24(1):75-82

© 2021 Rytter HM et al. *JAMA Network Open.*

Graded physical exercise (PICO 2)

Outcome measures Time frame	Results and measurements	Effect estimates		Confidence in the estimates (that they reflect the true effect in the population)	Summary
		No graded physical exercise	Graded physical exercise		
The collective burden of post-concussion symptoms End of treatment	Measured with: Rivermead post-concussion symptoms questionnaire, total score Scale: 0-64 Lower better Based on data from 201 patients in 2 studies[1]	Difference: **MD 4.78 lower** (CI 95% 8.08 lower - 1.47 lower)		**Low** Due to serious risk of bias, due to serious absence of transferability[2]	Graded physical exercise in addition to other treatment may lead to an improvement of the collective burden of post-concussion symptoms after the end of treatment.
The collective burden of post-concussion symptoms Follow-up (3-6 months after the end of treatment)	Measured with: Rivermead post-concussion symptoms questionnaire, total score Scale: 0-64 Lower better Based on data from 201 patients in 2 studies[3]	Difference: **MD 6.33 lower** (CI 95% 9.78 lower - 2.88 lower)		**Low** Due to serious risk of bias, due to serious absence of transferability[4]	Graded physical exercise in addition to other treatment may lead to an improvement of the collective burden of post-concussion symptoms at follow-up (3-6 months after the end of treatment).
Physical functioning End of treatment	Measured with: SF-36, Physical functioning and physical component summary (subscale) Scale: - Higher better Based on data from 201 patients in 2 studies[5]	Difference: **SMD 0.31 higher** (CI 95% 0.03 higher - 0.59 higher)		**Very low** Due to serious risk of bias, due to serious absence of transferability, due to seriously inaccurate effect estimate[6]	Graded physical exercise in addition to other treatment may increase the level of physical function to some extent after the end of treatment
Physical functioning Follow-up (3-6 months after the end of treatment)	Measured with: SF-36, Physical functioning and physical component summary (subscale) Scale: - Higher better Based on data from 201 patients in 2 studies[7]	Difference: **SMD 0.49 higher** (CI 95% 0.21 higher - 0.77 higher)		**Low** Due to serious risk of bias, due to serious absence of transferability[8]	Graded physical exercise in addition to other treatment may increase the physical function level at follow-up (3-6 months after the end of treatment)
Emotional symptoms Follow-up (3-6 months after the end of treatment)	Measured with: Major depression inventory (total score) Scale: - Lower better Based on data from 201 patients in 2 studies[9]	Difference: **SMD 0.4 lower** (CI 95% 0.60 lower - 0.12 lower)		**Very low** Due to serious risk of bias, due to seriously inaccurate effect estimate, due to serious absence of transferability[10]	Graded physical exercise in addition to other treatment may improve the emotional symptoms to some extent at follow-up (3-6 months after the end of treatment)
Behavioral responses Follow-up (3 months after the end of treatment)	Measured with: Behavioral response to illness questionnaire, limiting behavior (subscale) Scale: 0-100 Lower better Based on data from 112 patients in 1 study[11]	Difference: **MD 11.5 lower** (CI 95% 19.96 lower - 3.04 lower)		**Low** Due to serious risk of bias, due to serious absence of transferability[12]	Graded physical exercise in addition to other treatment may reduce the behavioral responses to some extent at follow-up (3 months after the end of treatment)

© 2021 Rytter HM et al. *JAMA Network Open.*

Quality of life Follow-up (3-6 months after the end of treatment)	Measured with: General questionnaire, satisfaction with leisure life (subscale). The quality of life after brain injury (overall scale) Scale: - Higher better Based on data from 201 patients in 2 studies[13]	Difference: **SMD 0.48 higher** (CI 95% 0.02 higher - 0.94 higher)	**Very low** Due to serious risk of bias, due to serious absence of transferability, due to seriously inaccurate effect estimate[14]	Graded physical exercise in addition to other treatment may lead to an improvement of quality of life to some extent at follow-up (3-6 months after the end of treatment)
Pain (Headache) End of treatment	Measured with: Headache impact test-6 (total score) Scale: 0-78 Lower better Based on data from 89 patients in 1 study[15]	Difference: **MD 1.42 lower** (CI 95% 0.25 lower - 3.09 higher)	**Low** Due to serious risk of bias, due to seriously inaccurate effect estimate[16]	Graded physical exercise in addition to other treatment may not affect aches (headache) after the end of treatment
General satisfaction with current working conditions Follow-up (6 months after the end of treatment)	Measured with: General questionnaire, satisfaction with current working conditions (subscale) Scale: 0-0.9 Higher better Based on data from 89 patients in 1 study[17]	Difference: **MD 0.63 higher** (CI 95% 0.11 higher - 1.15 higher)	**Low** Due to serious risk of bias, due to seriously inaccurate effect estimate[18]	Graded physical exercise in addition to other treatment may lead to improvement of the general satisfaction with current working conditions at follow-up (6 months after the end of treatment)
Negative effect on prognosis End of treatment				We found no studies examining the negative effect on the prognosis after the end of treatment

1. Meta-analysis for PICO 2 with included studies: Rytter et al. 2019, Thastum et al. 2019 **Baseline risk/comparator** Control arm in reference used for the intervention.

2. **Risk of bias: Serious.** Lack of blinding of participants. Lack of blinding of those who assess outcome.; **Lack of transferability: Serious.** Patients >15 years included.

3. Meta-analysis for PICO 2 with included studies: Thastum et al., 2019, Rytter et al., 2019. **Baseline risk/comparator** Control arm in reference used for the intervention.

4. **Risk of bias: Serious.** Lack of blinding of participants, lack of blinding of those who assess outcome.; **Lack of transferability: Serious.** Patients >15 years included.

5. Meta-analysis for PICO 2 with included studies: Rytter et al., 2019, Thastum et al., 2019. **Baseline risk/comparator** Control arm in reference used for the intervention.

6. **Risk of bias: Serious.** Lack of blinding of participants, lack of blinding of those who assess outcome.; **Lack of transferability: Serious.** Patients > 15 years included; **Inaccurate effect estimate: Serious.** Wide confidence intervals.

7. Meta-analysis for PICO 2 with included studies: Rytter et al., 2019, Thastum et al., 2019. **Baseline risk/comparator** Control arm in reference used for the intervention.

8. **Risk of bias: Serious.** Lack of blinding of participants, lack of blinding of those who assess outcome.; **Lack of transferability: Serious.** Patients >15 years included.

9. Meta-analysis for PICO 2 with included studies: Rytter et al., 2019, Thastum et al., 2019. **Baseline risk/comparator** Control arm in reference used for the intervention.

10. **Risk for bias: Serious.** Lack of blinding of participants, lack of blinding of those who assess outcome.; **Lack of transferability: Serious. Inaccurate effect estimate: Serious.** Wide confidence intervals.

11. Meta-analysis for PICO 2 with included studies: Thastum et al., 2019. **Baseline risk/comparator** Control arm in reference used for the intervention.

12. **Risk for bias: Serious.** Lack of blinding of participants, lack of blinding of those who assess outcome.; **Lack of transferability: Serious.** Patients >15 years included.

13. Meta-analysis for PICO 2 with included studies: Rytter et al., 2019, Thastum et al., 2019. **Baseline risk/comparator** Control arm in reference used for the intervention.

14. **Risk of bias: Serious.** Lack of blinding of participants, lack of blinding of those who assess outcome.; **Lack of transferability: Serious.** Patients >15 years included; **Inaccurate effect estimate: Serious.** Wide confidence intervals.

15. Meta-analysis for PICO 2 with included studies: Rytter et al, 2019. **Baseline risk/comparator** Control arm in reference used for the intervention.

16. **Risk of bias: Serious.** Lack of blinding of participants, lack of blinding of those who assess outcome.; **Inaccurate effect estimate: Serious.** Wide confidence intervals.

17. Meta-analysis for PICO 2 with included studies: Rytter et al., 2019. **Baseline risk/comparator** Control arm in reference used for the intervention.

18. **Risk of bias: Serious.** Lack of blinding of participants, lack of blinding of those who assess outcome.; **Inaccurate effect estimate: Serious.** Wide confidence intervals.

References

Meta-analysis for PICO 2: graded exercise therapy

Rytter HM, Westenbaek K., Henriksen H. et al.: Specialized interdisciplinary rehabilitation reduces persistent post-concussive symptoms: a randomized clinical trial. Brain Injury 2019;33(3):266-281.

Thastum MM, Rask CU, Naess-Schmidt E et al.: Novel interdisciplinary intervention, GAIN, vs. enhanced usual care to reduce high levels of post-concussion symptoms in adolescents and young adults 2-6 months post-injury: A randomised trial. EClinicalMedicine 2019;17 100214.

Vestibular rehabilitation (PICO 3)

Outcome measures Time frame	Results and measurements	Effect estimates		Confidence in the estimates (that they reflect the true effect in the population)	Summary
		No vestibular training	Vestibular training		
Number of patients assessed ready to return to sport End of treatment	Relative risk: 10.27 (CI 95% 1.52 - 69.55) Based on data from 29 patients in 1 study[1]	**71** per 1.000	**729** per 1.000	**Very low** Due to serious absence of transferability and a very seriously inaccurate effect estimate[2]	Vestibular rehabilitation in addition to other treatment may increase the number of patients assessed ready to return to sports within 8 weeks
		Difference: **658 more per 1.000** (CI 95% 37 more - 4867 more)			
Dizziness End of treatment	Measured with: DHI Scale: 0-100 Lower better Based on data from 63 patients in 1 study[3]	Difference: **MD 3.50 lower** (CI 95% 14.38 lower - 7.38 higher)		**Very low** Due to serious risk of bias, serious absence of transferability, and due to seriously inaccurate effect estimate[4]	Vestibular rehabilitation in addition to other treatment may not affect dizziness measured with DHI after the end of treatment
Dizziness Follow-up (2	Measured with: DHI Scale: 0-100 Lower better			**Very low** Due to serious risk of bias, serious	Vestibular rehabilitation in addition to other

© 2021 Rytter HM et al. *JAMA Network Open.*

months after the end of treatment)	Based on data from 55 patients in 1 study[5]	Difference: **MD 2.10 higher** (CI 95% 9.82 lower - 14.02 higher)	absence of transferability, and due to seriously inaccurate effect estimate[6]	treatment may not affect dizziness measured with DHI measured at follow-up (2 months after the end of treatment
Physical functioning End of treatment	Measured with: HiMAT Scale: 0-54 Higher better Based on data from 52 patients in 1 study[7]	Difference: **MD 6.40 higher** (CI 95% 0.76 higher - 12.04 higher)	**Very low** Due to serious risk of bias, serious absence of transferability, and due to seriously inaccurate effect estimate[8]	Vestibular rehabilitation in addition to other treatment may increase the physical function level to some extent measured with HiMAT after the end of treatment
Physical functioning Follow-up (2 months after the end of treatment)	Measured with: HiMAT Scale: 0-54 Higher better Based on data from 51 patients in 1 study[9]	Difference: **MD 3 higher** (CI 95% 1.89 lower - 7.89 higher)	**Very low** Due to serious risk of bias, serious absence of transferability, and a seriously inaccurate effect estimate[10]	Vestibular rehabilitation in addition to other treatment may not affect the physical function level measured by HiMAT measured at follow-up (2 months after the end of treatment)
Emotional symptoms (anxiety) Follow-up (2 months after the end of treatment)	Measured with: HADS, anxiety subscale Scale: 0-21 Lower better Based on data from 55 patients in 1 study[11]	Difference: **MD 0.70 higher** (CI 95% 1.68 lower - 3.08 higher)	**Very low** Due to serious risk of bias, serious absence of transferability, and a seriously inaccurate effect estimate[12]	Vestibular rehabilitation in addition to other treatment may not affect anxiety measured with HADS, anxiety subscale measured at follow-up (2 months after the end of treatment)
Emotional symptoms (Depression) Follow-up (2 months after the end of treatment)	Measured with: HADS, depression subscale Scale: 0-21 Lower better Based on data from 55 patients in 1 study[13]	Difference: **MD 1.60 higher** (CI 95% 1.05 lower - 4.25 higher)	**Very low** Due to serious risk of bias, serious absence of transferability, and a seriously inaccurate effect estimate[14]	Vestibular rehabilitation in addition to other treatment may not affect depression measured with HADS, depression subscale measured at follow-up (2 months after the end of treatment)
The collective burden of post-concussion symptoms End of treatment				We found no studies that reported the collective burden of post-concussion symptoms after the end of treatment
The collective burden of post-concussion symptoms Follow-up (3 months after the end of treatment)				We found no studies that reported the collective burden of post-concussion symptoms at follow-up
Vestibulo-ocular reflex End of treatment				We found no studies that reported the vestibulo ocular reflex at the end of treatment
Behavioral response				We found no studies that reported the behavioral

Follow-up (14 days after the end of treatment)				responses at follow-up
Quality of life Follow-up (3 months after the end of treatment)				We found no studies that reported the quality of life at follow-up
Negative effect on prognosis End of treatment				We found no studies that reported the negative effect on the prognosis after the end of treatment

1. Meta-analysis for PICO 3 with included studies: Schneider et al., 2014. **Baseline risk/comparator** Control arm in reference used for the intervention.
2. **Risk of bias: No considerable.** Lack of blinding of participants and personnel.; **Lack of transferability: Serious.** Patients >12 years included; **Inaccurate effect estimate: Very serious.** Wide confidence intervals, based on 1 study with few patients.
3. Meta-analysis for PICO 3 with included studies: Kleffelgaard et al., 2019. **Baseline risk/comparator** Control arm in reference used for the intervention.
4. **Risk of bias: Serious.** Lack of blinding of participants and personnel.; **Inconsistent results: No considerable.** The direction of the effect is not consistent between the included studies; **Lack of transferability: Serious.** Patients >12 years included; **Inaccurate effect estimate: Serious.** Wide confidence intervals, Few patients included in the study, Only data from one study.
5. Meta-analysis for PICO 3 with included studies: Kleffelgaard et al., 2019. **Baseline risk/comparator** Control arm in reference used for the intervention.
6. **Risk of bias: Serious.** Lack of blinding of participants and personnel.; **Lack of transferability: Serious.** Patients >12 years included; **Inaccurate effect estimate: Serious.** Wide confidence intervals, Few patients included in the study, only data from one study.
7. Meta-analysis for PICO 3 with included studies: Kleffelgaard et al., 2019. **Baseline risk/comparator** Control arm in reference used for the intervention.
8. **Risk of bias: Serious.** Lack of blinding of participants.; **Lack of transferability: Serious.** Patients >12 years included; **Inaccurate effect estimate: Serious.** Wide confidence intervals, Few patients included in the study, only data from one study.
9. Meta-analysis for PICO 3 with included studies: Kleffelgaard et al., 2019. **Baseline risk/comparator** Control arm in reference used for the intervention.
10. **Risk of bias: Serious.** Lack of blinding of participants.; **Lack of transferability: Serious.** Patients >12 years included; **Inaccurate effect estimate: Serious.** Wide confidence intervals, Few patients included in the study, only data from one study.
11. Meta-analysis for PICO 3 with included studies: Kleffelgaard et al., 2019. **Baseline risk/comparator** Control arm in reference used for the intervention.
12. **Risk of bias: Serious.** Lack of blinding of participants.; **Lack of transferability: Serous.** Patients >12 years included; **Inaccurate effect estimate: Serious.** Wide confidence intervals, Few patients included in the study, only data from one study.
13. Meta-analysis for PICO 3 with included studies: Kleffelgaard et al., 2019. **Baseline risk/comparator** Control arm in reference used for the intervention.
14. **Risk of bias: Serious.** Lack of blinding of participants.; **Lack of transferability: Serious.** Patients >12 years included; **Inaccurate effect estimate: Serious.** Wide confidence intervals, Few patients included in the study, only data from one study.

References

Meta-analysis for PICO 3: Vestibular rehabilitation.

Kleffelgaard I, Soberg HL, Tamber A-L et al: The effects of vestibular rehabilitation on dizziness and balance problems in patients after traumatic brain injury: a randomized controlled trial. Clinical rehabilitation 2019;33(1):74-84.

© 2021 Rytter HM et al. *JAMA Network Open.*

Schneider KJ, Meeuwisse WH, Nettel-Aguirre A et al.: Cervicovestibular rehabilitation in sport-related concussion: a randomised controlled trial. British journal of sports medicine 2014;48(17):1294-1298.

© 2021 Rytter HM et al. *JAMA Network Open.*

Manual treatment of neck and back (PICO 4)

**Outcome measures** Time frame	**Results and measurements**	**Effect estimates**		**Confidence in the estimates** (that they reflect the true effect in the population)	**Summary**
		No mobilization and manipulation techniques	Mobilization and manipulation techniques		
Number of patients assessed ready to return to sport End of treatment	Relative risk: 10.27 (CI 95% 1.52 - 69.55) Based on data from 29 patients in 1 study[1]	**71** per 1.000	**729** per 1.000	**Very low** due to serious absence of transferability, due to a very seriously inaccurate effect estimate[2]	Manual treatment of neck and back in addition to other treatment may increase the number of patients who are assessed ready to return to sports within 8 weeks after the end of treatment
		Difference: **658 more per 1.000** (CI 95% 37 more - 4867 more)			
Physical functioning End of treatment					We found no studies that reported the physical function level after the end of treatment
The collective burden of post-concussion symptoms End of treatment					We found no studies that reported the collective burden of post-concussion symptoms after the end of treatment
The collective burden of post-concussion symptoms Follow-up (3 months after the end of treatment)					We found no studies that reported the collective burden of post-concussion symptoms at follow-up
Emotional symptoms Follow-up (3 months after the end of treatment)					We found no studies that reported the emotional symptoms at follow-up
Behavioral response Follow-up (14 days after the end of					We found no studies that reported the behavioral responses at follow-up

© 2021 Rytter HM et al. *JAMA Network Open.*

treatment)				
Quality of life Follow-up (3 months after the end of treatment)				We found no studies that reported the life quality at follow-up
Physical functioning Follow-up (3 months after the end of treatment)				We found no studies that reported the physical function level at follow-up
Negative effect on prognosis End of treatment				We found no studies that examined the negative affect on the prognosis after the end of treatment
Pain End of treatment	Based on data from 19 patients in 1 study[3]	The study of Jensen et al (1990) reports that manual treatment of neck and back compared to the control group (use of ice pack) reduced the degree of aches by 43% after the end of treatment ($p < 0.05$). Aches was measured by a mean pain index. It was not possible to include the results in an analysis, due to incomplete reporting of data in the study.	**Very low** Due to very serious risk of bias, due to very seriously inaccurate effect estimate[4]	Manual treatment of neck and back may reduce the degree of aches to some extent after the end of treatment

1. Meta-analysis for PICO 4 with included studies: Schneider et al. 2014. **Baseline risk/comparator** Control arm in reference used for the intervention.

2. **Risk of bias: No considerable.** Lack of blinding of participants.; **Lack of transferability: Serious.** Patients >12 years included; **inaccurate effect estimate: Very serious.** Wide confidence interval, only data from one study, few patients included in the study.

3. Jensen et al. 1990.

4. **Risk of bias: Very serious.** Inadequate allocation sequence generation, insufficiently hidden randomization, lack of blinding of participants, lack of blinding of those who assess outcome, incomplete data.; **Inaccurate effect estimate: Very serious.** Only data from one study, few patients included in the study.

References

Meta-analysis for PICO 4: Manual treatment of neck and back.

Jensen OK, Nielsen FF, Vosmar L.: An Open Study Comparing Manual Therapy with the Use of Cold Packs in the Treatment of Post-Traumatic Headache. Cephalalgia 1990;10(5):241-250.

Schneider KJ, Meeuwisse WH, Nettel-Aguirre A et al.: Cervicovestibular rehabilitation in sport-related concussion: a randomised controlled trial. British journal of sports medicine 2014;48(17):1294-1298.

© 2021 Rytter HM et al. *JAMA Network Open.*

Oculomotor vision therapy (PICO 5)

**Outcome measures** Time frame	**Results and measurements**	**Effect estimates**		**Confidence in the estimates** (that they reflect the true effect in the population)	**Summary**
		No oculomoto rvision therapy	Oculomotor vision therapy		
All outcomes					We found no studies to answer this clinical question.

© 2021 Rytter HM et al. *JAMA Network Open.*

Psychological treatment (PICO 6)

**Outcome measures** Time frame	**Results and measurements**	**Effect estimates**		**Confidence in the estimates** (that they reflect the true effect in the population)	**Summary**
		No psychological treatment	Psychological treatment		
Number of patients with persisting post-concussion symptoms End of treatment	Relative risk: 0.29 (CI 95% 0.06 - 1.46) Based on data from 104 patients in 2 studies[1]	**596** per 1.000	**173** per 1.000	**Low** Due to serious risk of bias, due to seriously inaccurate effect estimate[2]	Psychological treatment may reduce the number of patients with continued post-commotional symptoms to some extent after the end of treatment
		Difference: **423 fewer per 1.000** (CI 95% 560 fewer - 274 more)			
Number of patients returned to work Follow-up (7 months after the end of treatment)	Relative risk: 0.85 (CI 95% 0.67 - 1.07) Based on data from 151 patients in 1 studies[3]	**714** per 1.000	**607** per 1.000	**Low** Due to serious risk of bias, due to serious absence of transferability, due to seriously inaccurate effect estimate[4]	Psychological treatment may not affect the number of patients who returned to work to a considerable extent at follow-up (7 months after the end of treatment)
		Difference: **107 fewer per 1.000** (CI 95% 236 fewer - 50 more)			
The collective burden of post-concussion symptoms End of treatment	Measured with: Rivermead post-concussion symptoms questionnaire Scale: - Lower better Based on data from 270 patients in 4 studies[5]	Difference: **MD 4.66 lower** (CI 95% 7.59 lower - 1.74 lower)		**Low** Due to serious risk of bias, due to serious absence of transferability[6]	Psychological treatment may reduce the collective burden of post-concussion symptoms after the end of treatment
The collective burden of post-concussion symptoms Follow-up (3-7 months after the end of treatment)	Measured with: Rivermead post-concussion symptoms questionnaire Scale: - Lower better Based on data from 750 patients in 5 studies[7]	Difference: **SMD 0.31 lower** (CI 95% 0.47 lower - 0.14 lower)		**Very low** Due to serious risk of bias, due to serious absence of transferability, due to seriously inaccurate effect estimate[8]	Psychological treatment may reduce the collective burden of post-concussion symptoms to some extent at follow-up (3-7 months after the end of treatment)
Physical functioning Follow-up (3-12 months after the end of treatment)	Measured with: SF-36, physical functioning; physical component summary Scale: - Higher better Based on data from 600 patients in 4 studies[9]	Difference: **SMD 0.18 higher** (CI 95% 0.17 lower - 0.52 higher)		**Very low** Due to serious risk of bias, due to seriously inconsistent results, due to serious absence of transferability, due to seriously inaccurate effect estimate[10]	Psychological treatment may not affect the physical function level to a considerable extent at follow-up (3-12 months after the end of treatment)
Emotional symptoms Follow-up (3-7 months after	Measured with: Psychological distress, SCL-8; major depression inventory, total score; HADS, total score; Global severity index	Difference: **SMD 0.28 lower** (CI 95% 0.49 lower - 0.07 lower)		**Very low** Due to serious risk of bias, due to serious absence of transferability, due to seriously inaccurate effect estimate[12]	Psychological treatment may reduce the emotional symptoms to some extent at follow-up (3-9 months after the end of treatment)

© 2021 Rytter HM et al. *JAMA Network Open.*

the end of treatment)	Scale: - Lower better<br>Based on data from 422 patients in 4 studies[11]			
Behavioral responses End of treatment	Measured with: BRIQ limiting<br>Scale: - Lower better<br>Based on data from 112 patients in 1 studies[13]	Difference: **MD 4.10 lower**<br>(CI 95% 11.93 lower - 3.73 higher)	**Very low**<br>Due to very serious risk of bias, due to serious absence of transferability, due to seriously inaccurate effect estimate[14]	Psychological treatment may not affect the behavioral responses to a considerable extent after the end of treatment
Quality of life Follow-up (3-6 months after the end of treatment)	Measured with: SF-36, general health; The quality of life after brain injury; General questionnaire, satisfaction with leisure life<br>Scale: - Higher better<br>Based on data from 273 patients in 3 studies[15]	Difference: **SMD 0.37 higher**<br>(CI 95% 0.04 higher - 0.71 higher)	**Very low**<br>Due to serious risk of bias, due to serious absence of transferability, due to seriously inaccurate effect estimate[16]	Psychological treatment may increase the quality of life to some extent at follow-up (3-6 months after the end of treatment)
Return to usual activity levels Follow-up (3-7 months after the end of treatment)	Measured with: Community integration questionnaire, total; MFI-20; reduced activities<br>Scale: - Higher better<br>Based on data from 416 patients in 2 studies[17]	Difference: **SMD 0.31 higher**<br>(CI 95% 0.75 lower - 0.12 higher)	**Very low**<br>Due to serious risk of bias, due to seriously inconsistent results, due to serious absence of transferability, due to seriously inaccurate effect estimate[18]	Psychological treatment may not affect the return to usual activity level to a considerable extent at follow-up (3-7 months after the end of treatment)
Cognitive functioning End of treatment				We found no studies examining cognitive function after the end of treatment
Negative effect on prognosis End of treatment				We found no studies examining the negative effect on the prognosis after the end of treatment

1. Meta-analysis for PICO 6 with included studies: Caplain et al., 2019, Silverberg et al. 2013. **Baseline risk/comparator** Control arm in reference used for the intervention.

2. **Risk of bias: Serious.** Insufficiently hidden randomization, lack of blinding, insufficient/lack of blinding in the evaluation.; **Inaccurate effect estimate: Serious.** Only data from one study, Few patients (<100) /(100-300) included in the studies.

3. Meta-analysis for PICO 6 with included studies: Vikane et al., 2017 **Baseline risk/comparator** Control arm in reference used for the intervention.

4. **Risk of bias: Serious.** Insufficient/lack of blinding in the evaluation, lack of blinding, Insufficiently hidden randomization.; **Inaccurate effect estimate: Serious.** Wide confidence intervals, Only data from one study.

5. Meta-analysis for PICO 6 with included studies: Rytter et al., 2019, Thastum et al., 2019, Potter et al., 2016, Silverberg et al., 2013 **Baseline risk/comparator** Control arm in reference used for the intervention.

6. **Risk of bias: Serious.** Lack of blinding, insufficient/lack of blinding in the evaluation; **Lack of transferability: Serious.** Includes patients aged 16 years or older.

7. Meta-analysis for PICO 6 with included studies: Thastum et al., 2019, Rytter et al., 2019, Kjeldgaard et al., 2014, Vikane et al., 2017, ElgmarkAndersson et al., 2007. **Baseline risk/comparator** Control arm in reference used for the intervention.

8. **Risk of bias: Serious.** Lack of blinding, insufficient/lack of blinding in the evaluation; **Lack of transferability: Serious.** Includes patients aged 16 years or more; **Inaccurate effect estimate: Serious.** Wide confidence intervals.

9. Meta-analysis for PICO 6 with included studies: ElgmarkAndersson et al., 2007, Rytter et al., 2019, Kjeldgaard et al., 2014, Thastum et al., 2019. **Baseline risk/comparator** Control arm in reference used for the intervention.

10. **Risk of bias: Serious.** Lack of blinding, insufficient/lack of blinding in the evaluation.; **Inconsistent results: Serious.** The statistical heterogeneity is high I2=72%; **Lack of transferability: Serious.** Includes patients aged 16 years or more; **Inaccurate effect estimate: Serious.** Wide confidence intervals.

© 2021 Rytter HM et al. *JAMA Network Open.*

11. Meta-analysis for PICO 6 with included studies: Vikane et al., 2017, Thastum et al., 2019, Kjeldgaard et al., 2014, Rytter et al., 2019. **Baseline risk/comparator** Control arm in reference used for the intervention.

12. **Risk of bias: Serious.** Lack of blinding, insufficient/lack of blinding in the evaluation.; **Lack of transferability: Serious.** Includes patients aged 15 years or more; **Inaccurate effect estimate: Serious.** Wide confidence intervals.

13. Meta-analysis for PICO 6 with included studies: Thastum et al., 2019. **Baseline risk/comparator** Control arm in reference used for the intervention.

14. **Risk of bias: Very serious.** Inadequate allocation sequence generation, insufficiently hidden randomization, lack of blinding, insufficient/lack of blinding in the evaluation.; **Lack of transferability: Serious.** Includes patients aged 15 years or more; **Inaccurate effect estimate: Serious.** Wide confidence intervals, Only data from one study.

15. Meta-analysis for PICO 6 with included studies: Kjeldgaard et al., 2014, Thastum et al., 2019, Rytter et al., 2019. **Baseline risk/comparator** Control arm in reference used for the intervention.

16. **Risk of bias: Serious.** Lack of blinding, insufficient/lack of blinding in the evaluation.; **Inconsistent results: No considerable.** The statistical heterogeneity is high; **Lack of transferability: Serious.** Includes patients aged 15 years or more; **Inaccurate effect estimate: Serious.** Wide confidence intervals.

17. Meta-analysis for PICO 6 with included studies: ElgmarkAndersson et al., 2007, Rytter et al., 2019. **Baseline risk/comparator** Control arm in reference used for the intervention.

18. **Risk of bias: Serious.** Lack of blinding, lack of blinding, insufficient/lack of blinding in the evaluation; **Inconsistent results: Serious.** The statistical heterogeneity is high, I2=56%; **Lack of transferability: Serious.** Includes patients aged 16 years or more; **Inaccurate effect estimate: Serious.** Wide confidence intervals.

References

Meta-analysis for PICO 6: Psychological treatment.

Caplain S., Chenuc G., Blancho S., et al.: Efficacy of Psychoeducation and Cognitive Rehabilitation After Mild Traumatic Brain Injury for Preventing Post-concussional Syndrome in Individuals With High Risk of Poor Prognosis: A Randomized Clinical Trial. Frontiers in Neurology 2019;10 929

Elgmark Andersson E., Emanuelson I., Bjorklund R., et al.: Mild traumatic brain injuries: the impact of early intervention on late sequelae. A randomized controlled trial. Acta Neurochirurgica 2007;149(2):151-159

Kjeldgaard D., Forchhammer H.B., Teasdale T.W. et al.: Cognitive behavioural treatment for the chronic post-traumatic headache patient: a randomized controlled trial. Journal of Headache and Pain 2014;15(1):81.

Potter SDS, Brown RG, Fleminger S: Randomised, waiting list controlled trial of cognitive-behavioural therapy for persistent postconcussional symptoms after predominantly mild-moderate traumatic brain injury. Journal of Neurology, Neurosurgery & Psychiatry 2016;87(10):1075-1083

Rytter HM, Westenbaek K., Henriksen H. et al: Specialized interdisciplinary rehabilitation reduces persistent post-concussive symptoms: a randomized clinical trial. Brain Injury 2019;33(3):266-281.

Silverberg ND, Hallam BJ, Rose A, et al.: Cognitive-behavioral prevention of postconcussion syndrome in at-risk patients: a pilot randomized controlled trial. Journal of Head Trauma Rehabilitation 2013;28(4):313-322

© 2021 Rytter HM et al. *JAMA Network Open.*

Thastum MM, Rask CU, Naess-Schmidt E et al.: Novel interdisciplinary intervention, GAIN, vs. enhanced usual care to reduce high levels of post-concussion symptoms in adolescents and young adults 2-6 months post-injury: A randomised trial. EClinicalMedicine 2019;17 100214.

Tiersky LA, Anselmi V, Johnston MV, et al.: A trial of neuropsychologic rehabilitation in mild-spectrum traumatic brain injury. Archives of Physical Medicine & Rehabilitation 2005;86(8):1565-1574

Vikane E, Hellstrom T, Roe C et al: Multidisciplinary outpatient treatment in patients with mild traumatic brain injury: A randomised controlled intervention study. Brain Injury 2017;31(4):475-484.

© 2021 Rytter HM et al. *JAMA Network Open.*

Interdisciplinary coordinated rehabilitative treatment (PICO 7)

Outcome measures Time frame	Results and measurements	Effect estimates		Confidence in the estimates (that they reflect the true effect in the population)	Summary
		No interdisciplinary coordinated rehabilitative treatment	Interdisciplinary coordinated rehabilitative treatment		
Return to work Follow-up (6 months after the end of treatment)	Relative risk: 0.85 (CI 95% 0.67 - 1.07) Based on data from 151 patients in 1 study[1]	**714** per 1.000	**607** per 1.000	**Low** Due to seriously inaccurate effect estimate, due to serious risk of bias[2]	Interdisciplinary coordinated rehabilitative treatment may not affect the number of patients returning to work
		Difference: **107 fewer per 1.000** (CI 95% 236 fewer - 50 more)			
General satisfaction with current working conditions Follow-up (6 months after the end of treatment)	Measured with: General questionnaire, satisfaction with current working conditions (subscale) Scale: 0-0.9 Higher better Based on data from 89 patients in 1 study[3]	Difference: **MD 0.63 higher** (CI 95% 0.11 higher - 1.15 higher)		**Low** Due to serious risk of bias, due to seriously inaccurate effect estimate[4]	Interdisciplinary coordinated rehabilitative treatment may lead to an improvement of the general satisfaction with current working conditions
The collective burden of post-concussion symptoms End of treatment	Measured with: Rivermead post-concussion symptoms questionnaire, total score Scale: 0-64 Lower better Based on data from 201 patients in 2 studies[5]	Difference: **MD 4.78 lower** (CI 95% 8.08 lower - 1.47 lower)		**Low** Due to serious absence of transferability, due to serious risk of bias[6]	Interdisciplinary coordinated rehabilitative treatment may lead to a reduction of the total burden of post-commotional symptoms after the end of treatment
The collective burden of post-concussion symptoms Follow-up (3-9 months after the end of treatment)	Measured with: Rivermead post-concussion symptoms questionnaire, total score Scale: 0-64 Lower better Based on data from 352 patients in 3 studies[7]	Difference: **MD 6.42 lower** (CI 95% 9.73 lower - 3.11 lower)		**Low** Due to serious risk of bias, due to serious absence of transferability[8]	Interdisciplinary coordinated rehabilitative treatment may lead to a reduction of the collective burden of post-concussion symptoms at follow-up (3-9 months after the end of treatment)
Physical functioning End of treatment	Measured with: SF-36, Physical functioning and physical component summary (subscale) Scale: 0-100 Higher better Based on data from 201 patients in 2 studies[9]	Difference: **SMD 0.31 higher** (CI 95% 0.03 higher - 0.59 higher)		**Very low** Due to serious risk of bias, due to seriously inaccurate effect estimate, due to serious absence of transferability[10]	Interdisciplinary coordinated rehabilitative treatment may increase the physical function level to some extent after the end of treatment
Emotional symptoms	Measured with: Major depression inventory (total			**Very low** Due to serious risk of bias, due to	Interdisciplinary coordinated rehabilitative

© 2021 Rytter HM et al. *JAMA Network Open.*

Follow-up (3-9 months after the end of treatment)	score), Hospital Anxiety and Depression scale (total score), Symptom Checklist-8 (total score) Scale: - Lower better Based on data from 352 patients in 3 studies[11]	Difference: **SMD 0.27 lower** (CI 95% 0.54 lower - 0 higher)	serious absence of transferability, due to seriously inaccurate effect estimate[12]	treatment may improve the emotional symptoms to some extent at follow-up (3-9 months after the end of treatment)
Behavioral responses End of treatment	Measured with: Behavioral response to illness questionnaire (limiting behavior, subscale) Scale: 0-100 Lower better Based on data from 112 patients in 1 study[13]	Difference: **MD 4.10 lower** (CI 95% 11.93 lower - 3.73 higher)	**Very low** Due to serious risk of bias, due to serious absence of transferability, due to seriously inaccurate effect estimate[14]	Interdisciplinary coordinated rehabilitative treatment may not affect the behavioral responses after the end of treatment
Quality of life Follow-up (3-9 months after the end of treatment)	Measured with: General questionnaire, satisfaction with leisure life (subscale). The quality of life after brain injury (overall scale) Scale: - Higher better Based on data from 201 patients in 2 studies[15]	Difference: **SMD 0.48 higher** (CI 95% 0.02 higher - 0.94 higher)	**Very low** Due to serious risk of bias, due to seriously inconsistent results, due to serious absence of transferability, due to seriously inaccurate effect estimate[16]	Interdisciplinary coordinated rehabilitative treatment may lead to an increase in quality of life at follow-up to some extent (3-9 months after the end of treatment)
Pain (Headache) End of treatment	Measured with: Headache impact test-6 (total score) Scale: 0-78 Lower better Based on data from 89 patients in 1 study[17]	Difference: **MD 1.42 lower** (CI 95% 0.25 lower - 3.09 higher)	**Low** Due to serious risk of bias, due to seriously inaccurate effect estimate[18]	Interdisciplinary coordinated rehabilitative treatment may not affect aches (headache) after the end of treatment
Cognitive functioning End of treatment				We found no studies that reported cognitive function after the end of treatment
Negative effect on prognosis End of treatment				We found no studies that reported a potential negative effect on the prognosis after the end of treatment

1. Meta-analysis for PICO 7 with included studies: Vikane et al., 2017. **Baseline risk/comparator** Control arm in reference used for the intervention.
2. **Risk of bias: Serious.** Lack of blinding of participants, lack of blinding of those who assess outcome.; **Inaccurate effect estimate: Serious.** Only data from one study, Wide confidence intervals.
3. Meta-analysis for PICO 7 with included studies: Rytter et al., 2019. **Baseline risk/comparator** Control arm in reference used for the intervention.
4. **Risk of bias: Serious.** Lack of blinding of participants, lack of blinding of those who assess outcome.; **Inaccurate effect estimate: Serious.** Only data from one study.
5. Meta-analysis for PICO 7 with included studies: Thastum et al., 2019, Rytter et al., 2019. **Baseline risk/comparator** Control arm in reference used for the intervention.
6. **Risk of bias: Serious.** Lack of blinding of participants, lack of blinding of those who assess outcome.; **Lack of transferability: Serious.** Patients >15 years included.
7. Meta-analysis for PICO 7 with included studies: Rytter et al., 2019, Vikane et al., 2017, Thastum et al., 2019. **Baseline risk/comparator** Control arm in reference used for the intervention.
8. **Risk of bias: Serious.** Lack of blinding of participants, lack of blinding of those who assess outcome. **Lack of transferability: Serious.** Patients > 15 years included.
9. Meta-analysis for PICO 7 with included studies: Thastum et al., 2019, Rytter et al., 2019. **Baseline risk/comparator** Control arm in reference used for the intervention.

© 2021 Rytter HM et al. *JAMA Network Open.*

10. **Risk of bias: Serious.** Lack of blinding of participants, lack of blinding of those who assess outcome.; **Lack of transferability: Serious.** Patients >15 years included; **Inaccurate effect estimate: Serious.** Wide confidence intervals, Only data from one study.

11. Meta-analysis for PICO 7 with included studies: Thastum et al., 2019, Vikane et al., 2017, Rytter et al., 2019. **Baseline risk/comparator** Control arm in reference used for the intervention.

12. **Risk of bias: Serious.** Lack of blinding of participants, lack of blinding of those who assess outcome.; **Lack of transferability: Serious.** Patients > 15 years included; **Inaccurate effect estimate: Serious.** Wide confidence intervals.

13. Meta-analysis for PICO 7 with included studies: Thastum et al., 2019. **Baseline risk/comparator** Control arm in reference used for the intervention.

14. **Risk of bias: Serious.** Lack of blinding of the participants, lack of blinding of those who assess outcome.; **Lack of transferability: Serious.** Patients >16 years included; **Inaccurate effect estimate: Serious.** Wide confidence intervals, Only data from one study.

15. Meta-analysis for PICO 7 with included studies: Rytter et al., 2019, Thastum et al., 2019. **Baseline risk/comparator** Control arm in reference used for the intervention.

16. **Risk of bias: Serious.** Lack of blinding of participants, lack of blinding of those who assess outcome.; **Inconsistent results: Serious.** The statistical heterogeneity is high, I2=62%; **Lack of transferability: Serious.** Patients >15 years included; **Inaccurate effect estimate: Serious.** Wide confidence intervals.

17. Meta-analysis for PICO 7 with included studies: Rytter et al., 2019. **Baseline risk/comparator** Control arm in reference used for the intervention.

18. **Risk of bias: Serious.** Lack of blinding of participants, lack of blinding of those who assess outcome.; **Inaccurate effect estimate: Serious.** Wide confidence intervals, only data from one study.

References

Meta-analysis for PICO 7: Interdisciplinary coordinated rehabilitative treatment.

Rytter HM, Westenbaek K., Henriksen H. et al.: Specialized interdisciplinary rehabilitation reduces persistent post-concussive symptoms: a randomized clinical trial. Brain Injury 2019;33(3):266-281.

Thastum MM, Rask CU, Naess-Schmidt E et al.: Novel interdisciplinary intervention, GAIN, vs. enhanced usual care to reduce high levels of post-concussion symptoms in adolescents and young adults 2-6 months post-injury: A randomised trial. EClinicalMedicine 2019;17 100214.

Vikane E, Hellstrom T, Roe C et al.: Multidisciplinary outpatient treatment in patients with mild traumatic brain injury: A randomised controlled intervention study. Brain Injury 2017;31(4):475-484.

© 2021 Rytter HM et al. *JAMA Network Open.*

eTable 5. Characteristics of the Included Studies in the Systematic Review

Source	Study design	Setting	Diagnostic criteria	Age, mean or median (range),[a]	Male, No. (%)[b]	Female, No. (%)[b]	No. of participants		Intervention (IG) vs control group (CG)	Outcome measures[c]	Follow-up period	Summary of results
							IG	CG				
Mittenberg et al[48]	RCT	ED	GCS 13-15 points at the time of admission and without measurable period of PTA (GOAT ≥75 points)	43.35 (NS)	40 (69)	18 (31)	29	29	IG: A printed 10-page manual, and a meeting with a therapist.<br>CG: Routine hospital treatment and discharge instructions in written form.	A structured symptom checklist (frequency, intensity, and duration)	6-mo follow-up	IG had significantly shorter average symptom duration and fewer symptoms at 6-mo follow-up; significantly fewer symptomatic days and lower mean severity levels.
Bell et al[45]	RCT	ED	GCS 13-15 points, LOC ≤30 min, impaired consciousness, eg, confusion or PTA ≤24 h	33 (NS)	235 (75)	78 (25)	146	167	IG: Scheduled telephone contacts in the first 3 mo, standard patient instruction handout, a wallet card with a telephone number, and a booklet, in addition to usual care (n = 207).<br>CG: standard care with a patient instruction handout and standard outpatient treatment (n = 159).	Head Injury Symptom Checklist, SF-12, modified PQOL, PHQ-Depression and Panic/Anxiety, questions on change in role performance and participation in activities.	6-mo follow-up	IG showed a significantly better outcome regarding PCS and daily functioning (largest difference in work, leisure activities, memory and concentration, and financial independence) at follow-up but no difference in general health outcome.
Heksestad et al[46]	RCT	Universi-ty hospital	Classified as minimal, mild, moderate, or severe according to the Head Injury Severity Scale	40 (16-65)	32 (64)	8 (36)	22	28	IG: Cognitive oriented counseling, advice, additional information and reassurance, coping strategies.<br>CG: No treatment.	BDI. PCS examined as proportion of patients reporting headache and irritability.	3- and 12-mo follow-up	No statistically significant differences between IG and CG.

© 2021 Rytter HM et al. *JAMA Network Open.*

Matusevi-ciene et al[47]	RCT	ED	LOC ≤30 min and/or posttraumatic PTA ≤1 h and GCS 14-15 points on presentation in the ED.	41 (15-69)	38 (39)	59 (61)	48	49	IG: Consultation with a physician at 14-21 d postinjury and screening for anxiety and depression, including a detailed interview about symptoms, daily functioning, and comorbidities.<br>CG: Standard somatic examination and verbal and written information about mTBI and expected favorable outcomes.	HADS	3-mo follow-up	Anxiety and depression scores did not differ between the groups.
Varner et al[51]	RCT	ED	Patients presenting to the ED with complaints of "head injury" occurring within the last 24 h.	34.3 (NS)	43 (36)	75 (64)	60	58	IG: Cognitive rest and graduated return to usual activity discharge instructions.<br>CG: Usual care discharge instructions.	PCSS questionnaire and number of missed days of school or work.	4 wk after ED discharge	No difference in PCSS and number of missed days of school or work at 4 wk post-ED discharge between IG and CG.
Suffoletto et al[50]	RCT	ED	American Congress of Rehabilitation Medicine definition of mTBI: (LOC ≤30 min, GCS 13-15 points, PTA ≤24 h).	30 (NS)	19 (44)	24 (56)	18	25	IG: Symptom-specific education, reassurance and management guidance addition to standard care.<br>CG: Standard of care including patient instruction handout and outpatient referral, if needed.	RPQ; PHQ-4. Postinjury acute pain (head) measured by a visual analog scale (0-10 points).	15-d follow-up	IG had less severe PCS and anxiety than the CG, but results were not statistically significant. IG had lower mean scores for headaches.
Ponsford[d] et al[49]	Cohort study with	ED	LOC ≤30 min, PTA ≤24 h, GCS 13-15 points on	24.1 (NS)	NS	NS	79	123	IG: Information booklet outlining the symptoms associated with mild head injury	The postconcussion syndrome checklist, SCL-	3-mo follow-up	IG had fewer symptoms overall and was significantly less

© 2021 Rytter HM et al. *JAMA Network Open.*

	control group		presentation to the ED.						and suggested coping strategies.<br>CG: Standard treatment.	90-R, the Holmes Rahe survey of recent experiences, neuropsychological measures of premorbid intelligence quotient, attention, memory, and information processing speed.		stressed than CG at 3 mo postinjury.
Rytter et al[42]	RCT	Speciali-zed post-acute outpatient hospital	Documented concussion, persistent PCS for >6 mo postinjury according to the ICD-10 diagnostic criteria for postconcussion syndrome	NS (18-65)	30 (34)	59 (66)	45	44	IG: Interdisciplinary rehabilitation program with individual- and group-based interventions within neuropsychology, cognitive behavioral therapy, and physical therapy.<br>CG: Usual treatment offered by the public municipality services.	RPQ, the HIT-6, MDI, Health Survey (SF-36), MFI-20, Satisfaction with current working conditions and leisure life (questionnaire).	6-mo follow-up	IG showed a significantly greater reduction in RPQ, HIT-6, and improvements SF-36 (social functioning), MFI-20 (mental fatigue) and life satisfaction compared with CG.
Thastum et al[43]	RCT	Recruited from a cohort study on concu-ssion or referred by GPs	Concussion within 2-6 mo according to the World Health Organization's diagnostic criteria, high levels of PCS defined as ≥20 points on the RPQ	22.9 (NS)	23 (21)	89 (79)	57	55	IG: Interdisciplinary intervention program based on cognitive behavioral therapy and gradual return to activities, including group and individual sessions.<br>CG: Enhanced usual care including reassurance and individual psychoeducation on	RPQ, BRIQ (subscale "limiting behavior"), Quality of Life after Brain Injury, the SF-36 Health Survey (mental and physical health), Symptom Checklist-8	3 mo follow-up	IG had significantly larger reductions in postconcussion symptoms on RPQ at 3-mo follow-up, in symptom perpetuating illness perceptions and illness behaviors, and in overall physical health compared with CG.

© 2021 Rytter HM et al. *JAMA Network Open.*

									good prognosis and gradual resumption of premorbid activities.			
Kleffel-gaard et al[53]	RCT	Outpatient clinic at a university hospital	Reported mild, moderate, or severe feelings of dizziness on the RPQ (dizziness score ≥2 points) and/or had a positive Romberg test	37,6 (NS)	20 (31)	45 (69)	33	32	Both IG and CG received usual multidisciplinary outpatient rehabilitation comprising clinical examinations, assessments, and follow-ups. In addition, IG received a group-based vestibular rehabilitation with guidance, individually tailored exercises, a home exercise program, and an exercise diary.	DHI, High-Level Mobility Assessment Tool, HADS	2 mo after end of treatment	IG showed larger improvements in mean scores in dizziness-related disability and mobility than CG.
Schneider et al[52]	RCT	University of Calgary Sport Medicine Centre	Sport-related concussion and persistent symptoms (>10 d) of dizziness, neck pain and/or headaches reported on the Sport Concussion Assessment Tool 2	15 (12-27)	18 (58)	13 (42)	15	16	Both groups received weekly sessions with a physiotherapist, postural education, range-of-motion exercises, and cognitive and physical rest until asymptomatic followed by a protocol of graded exertion. In addition, IG received cervical spine treatment and vestibular rehabilitation.	Time to medical clearance to return to sport (d)	8-wk follow-up or until reaching medical clearance	A larger proportion of the included participants in IG were medically cleared to return to sport within 8 wk of initiating treatment compared with CG.
Jensen[e] et al[44]	RCT	County hospital	Diagnosis of cerebral concussion or	31.6 (19-48)	7 (37)	12 (63)	10	9	IG: Segmental spine examination of the neck and manual	Pain index (the intensity of the headache) was	Outcomes were assessed	IG had lower frequency of symptoms than CG,

© 2021 Rytter HM et al. *JAMA Network Open.*

			suspected cerebral concussion and posttraumatic headache						treatment (soft passive movements of the joint at the outer range of motion) of hypomobile segments.	measured on a visual analog scale	after end of treatment	but the differences were not statistically significant.
									CG: Cold packs on the neck.			
Caplain et al[54]	RCT	General hospital	The American Congress of Rehabilitation Medicine definition on mTBI, including LOC ≤30 min, an initial GCS 13-15 points after 30 min and PTA ≤24 h	38.14 (NS)	30 (38)	50 (62)	34	46	IG: psychoeducation, pain management, evaluation of mood disorders, and cognitive rehabilitation with computer-based exercises.	The presence of PCS according to the DSM-IV diagnosis of postconcussion syndrome	6-mo follow-up	Among the IG, 94% did not fulfill the criteria for postconcussion syndrome, whereas 52% in the CG fulfilled the criteria.
									CG: Information on the natural course of mTBI and pain management.			
Elgmark-Andersson et al[55]	RCT	County hospital	The American Congress of Rehabilitation Medicine definition on mTBI: LOC ≤30 min, an initial GCS 13-15 points after 30 min and PTA ≤24 h	32 (NS)	245 (62)	150 (38)	264	131	IG: Psychoeducation with oral information, counseling, encouragement, and if needed, pharmacotherapy for pain, depression, and sleep disturbances.	PCSQ, SF-36 (physical functioning), CIQ	3-mo follow-up	No statistically significant differences between the IG and CG.
									CG: Standard care.			
Kjeldgaard et al[56]	RCT	Multidisciplinary, tertiary headache center	Diagnosis of posttraumatic headache (CPTH) according to ICHD-II after mild head injury	34 (NS)	40 (44)	50 (56)	45	45	IG: Cognitive behavioral therapy group intervention including education, coping strategies, and relaxation techniques.	RPQ, SF-36 (physical functioning and general health), SCL-90-R	26 wk follow-up	IG experienced only minor effects on quality of life (physical functioning and general health), psychological distress (SCL-90-R), and the overall experience of
									CG: No active treatment (waiting-list patients).			

© 2021 Rytter HM et al. *JAMA Network Open.*

												symptoms (RPQ) compared with CG.
Potter et al[57]	RCT	Outpatient referrals to National Health Service secondary /tertiary care brain injury clinics	Documented mTBI at least 6 mo before and symptoms consistent with the ICD-10 criteria for postconcussion disorder	40.1 (NS)	25 (54)	21 (46)	26	20	IG: Individual cognitive behavioral therapy.<br>CG: No psychological intervention.	The Quality of Life Assessment Schedule and the Brain Injury Community Rehabilitation Outcome Scale, RPQ, measures of anxiety, depression, and fatigue	12 wk after end of treatment	IG had significant improvements in quality of life, PCS, anxiety, and fatigue, after covarying for treatment duration compared with CG.
Silverberg et al[58]	RCT	Tertiary rehabilitation center	Head trauma within 6 wk, American Congress of Rehabilitation Medicine criteria for mTBI	40.4 (NS)	11 (39)	17 (61)	15	13	IG: Cognitive behavioral therapy targeting maladaptive beliefs, coping behaviors, and perceived control associated with chronic PCS.<br>CG: Treatment as usual including written information and a 3-h session about symptoms, symptom management, and gradual return to preinjury activities.	RPQ	3-mo follow-up	IG had fewer PCS on the RPQ at 3-mo follow-up compared with CG.
Vikane et al[60]	RCT	Department of neurosurgery	ICD-10 diagnosis S06.0-S06.9, with sustained symptoms at 6 to 8 wk post-mTBI. GCS 13-15 points within 30 min or the lowest score during the first 24	31 (NS)	92 (61)	59 (39)	71	80	IG: Multidisciplinary outpatient follow-up program consisting of individual contacts and psychoeducational group intervention.<br>CG: regular unstandardized treatment at GP.	RPQ, HADS, No. of d to RTW, GOSE	12-mo follow-up	IG had significantly fewer PCS at RPQ at 12 mo follow-up compared with CG. There was no difference between the groups on RTW, HADS, and disability (GOSE).

© 2021 Rytter HM et al. *JAMA Network Open.*

			h, LOC ≤30 min and PTA ≤24 h									
Tiersky[f] et al[59]	RCT	Outpatient clinic	Mild or moderate head injury according to Congress of Rehabilitation Medicine (ACRM); DRS score between 1 and 5 points, complaints of attention and memory problems determined by the ACFI, complaints of emotional distress determined by the SCL-90R at study inclusion	47.55 (NS)	9 (45)	11 (55)	11	9	IG: Neuropsychologic rehabilitation program, consisting of cognitive remediation and cognitive behavioral therapy.<br>CG: Meeting with a professional in person or by telephone for 2 or 3 times over the course of 11 wk with discussion about patient-chosen topics.	GSI (SCL-90R), Depression (SCL-90R), Anxiety (SCL-90R), PASAT, problem-solving and emotional discharge Coping Response Inventory, attention questionnaire, somatization (SCL-90R), RAVLT, ACFI, CIQ	Immediately after treatment (11 wk after trauma), 1 mo and 3 mo after end of treatment	IG showed significant improvements in emotional functioning (anxiety and depression), emotional distress, and performance on a measure of divided auditory attention compared with CG. No significant changes were reported for community integration scores.

Abbreviations: ACFI, Assessment of Client Functioning Inventory; BDI, Beck Depression Inventory; BRIQ, Behavioral Responses to Illness Questionnaire; CG, control group; CIQ, The Community Integration Questionnaire; DHI, Dizziness Handicap Inventory; DRS, Disability Rating Scale; *DSM-IV*, *Diagnostic and Statistical Manual of Mental Disorders* (Fourth Edition); ED, emergency department; GCS, Glasgow Coma Scale score; GOAT, Galveston Orientation and Amnesia Test; GOSE, The Extended Glasgow Outcome Scale; GP, general practitioner; HADS, Hospital Anxiety and Depression Scale; HIT-6, Headache Impact Test-6; *ICD-10*, *International Statistical Classification of Diseases and Related Health Problems, Tenth Revision*; IG, intervention group; LOC, Loss of Consciousness; MDI, Major Depression Inventory; MFI-20, Multidimensional Fatigue Inventory-20 items; mTBI, mild traumatic brain injury; NS, not specified; PASAT, Paced Auditory Serial Addition Test; PCS, postconcussion symptoms; PCSS, Postconcussion Symptom Score; PCSQ, Postconcussion Symptoms Questionnaire; PHQ-4, Patient Health Questionnaire-4; PQOL, Perceived Quality of Life; PTA, posttraumatic amnesia; RAVLT, The Rey Auditory Verbal Learning Test; RCT, randomized clinical trial; RPQ, Rivermead Postconcussion Symptoms Questionnaire; RTW, return to work; SCL-90-R, The Symptom Checklist-90-Revised; SF-12, Short-Form Health Survey-12; SF-36, The Short-Form Health Survey.

[a]Age of the intervention group is reported as mean or median based on the data from the included studies.
[b]Sex distributions provided across both intervention group and control group.
[c]Only outcome measures included in the analysis are shown in the table.
[d]Ponsford et al[49] not included in the meta-analysis.
[e]Jensen et al[44] not included in the meta-analysis.
[f]Tiersky et al[59] not included in the meta-analysis.

© 2021 Rytter HM et al. *JAMA Network Open.*
